# Synopsis of Central Andean Orthalicoid land snails (Gastropoda, Stylommatophora), excluding Bulimulidae

**DOI:** 10.3897/zookeys.588.7906

**Published:** 2016-05-12

**Authors:** Abraham S.H. Breure, Valentín Mogollón Avila

**Affiliations:** 1 Naturalis Biodiversity Center, P.O. Box 9517, 2300 RA Leiden, the Netherlands; 2 Royal Belgian Institute of Natural Sciences, Vautierstraat 29, 1000 Brussels, Belgium; 3 Universidad Nacional Federico Villarreal, Facultad de Oceanografía, Pesquería y Ciencias Alimentarias, Roma 350, Lima 18, Peru

**Keywords:** Mollusca, Orthalicoidea, Bolivia, Ecuador, Peru, distribution, ecology, taxonomy

## Abstract

A faunal overview is presented of the molluscan families Amphibulimidae, Megaspiridae, Odontostomidae, Orthalicidae, Simpulopsidae in Bolivia, Ecuador, and Peru. These Central Andean countries are known for their biodiverse malacofauna, of which the superfamily Orthalicoidea takes relatively a large share. In this paper the five families containing 103 (sub)species, for which systematic information (original publication, type locality, type depository, summarizing literature) and distributional records are presented. All species are illustrated by photographs of the type material or, if this could not be located, by a reproduction of the original figure.

The following new taxon is introduced: Thaumastus (Thaumastus) sumaqwayqu **sp. n.** Junior subjective synonyms are established for: Plekocheilus (Sparnotion) Pilsbry, 1944 = Plekocheilus (Eudolichotis) Pilsbry, 1896; Scholvienia (Thomsenia) Strebel, 1910 = *Scholvienia* Strebel, 1910; Sultana (Trachyorthalicus) Strebel, 1909 = Sultana (Metorthalicus) Pilsbry, 1899; Plekocheilus (Eurytus) conspicuus Pilsbry, 1932 = Thaumastus (Thaumastus) hartwegi (Pfeiffer in Philippi, 1846); *Zebra gruneri* Strebel, 1909 = *Orthalicus maracaibensis* (Pfeiffer, 1856); *Scholvienia jaspidea minor* Strebel, 1910 = Scholvienia alutacea (Reeve, 1850); *Bulimus bifasciatus unicolor* Philippi, 1869 = Scholvienia brephoides (d’Orbigny, 1835). A new status is given to *Plekocheilus mcgintyi* ‘Pilsbry’ H.B. Baker, 1963 (subspecies of *Bulinus piperitus* Sowerby I, 1837); Strophocheilus superstriatus var. prodeflexus Pilsbry, 1895 (subspecies of *Bulinus piperitus* Sowerby I, 1837); Thaumastus (Quechua) salteri maximus Weyrauch, 1967 (subspecies of Thaumastus (Quechua) olmosensis Zilch, 1954); *Pseudoglandina agitata* Weyrauch, 1967 (nomen inquirendum). New combinations are: Clathrorthalicus corydon (Crosse, 1869), and Cyclodontina chuquisacana (Marshall, 1930). Lectotypes are now designated for *Bulimus incisus* Hupé, 1857 and *Bulinus piperitus* Sowerby I, 1837.

## Introduction

Faunal overviews are the keystones of modern biodiversity research, and while for many countries an overview is now available, this remains very fragmentary for most of the Neotropical realm.

As far as current knowledge goes, the land snail malacofauna of Peru is one of the richest in the Neotropical realm (Breure and Mogollón 2010: fig. 1), with 763 species (Ramírez et al. 2003). The land snail superfamily Orthalicoidea accounts for 58% and thus forms a dominant family in the fauna. Checklists or catalogues for several other countries or regions within the Neotropical realm have appeared only mostly recently: Argentina (Fernández 1973; Cuezzo et al. 2013), Bolivia (Zischka 1953), Brazil (Simone 2006), Central America (Thompson 2011), Colombia (Linares and Vera 2012), Cuba (González Guillén 2008, Espinosa and Ortea 2009), and French Guiana (Massemin et al. 2009); partial works have been published for Ecuador (Breure and Borrero 2008, Correoso 2008).

**Figure 1.**
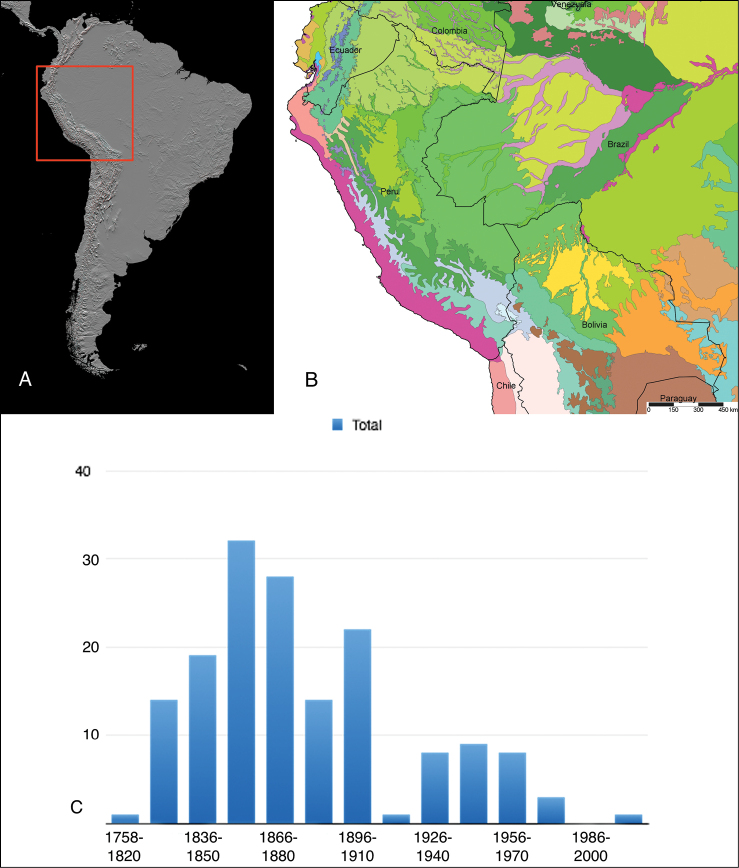
**A–B** Map of the study area, with ecoregions. For explanation, see Appendix 4 **C** Number of first descriptions of taxa mentioned in this paper, arranged into 15-years periods.

The study area for this paper comprises the countries from the Central Andean area, i.e. Ecuador, Peru and Bolivia (Figure 1A–B). Although some dispersed species descriptions appeared in publications during the early 19th century, the first major contribution was made by Alcide d’Orbigny (1834–1847, 1835) who travelled extensively in Brazil, Bolivia and Peru; details of his itinerary can be found in Papavero (1971), the localities have been transformed to modern geography by Breure (1973). The collections made by Hugh Cuming during his travels in the same era has been elaborated on elsewhere (Breure and Ablett 2011: 4–5). During the mid-19th century other expeditions followed, e.g., a French expedition during 1843–1847 (Hupé 1857) and a Spanish one during 1862–1865 (Hidalgo 1870, 1872, 1893a, 1893b, Breure and Araujo 2015). At the same time many individual travellers (e.g., Jørgensen 2015 for travellers in Ecuador) during this era brought smaller or larger collections of shells, from which new taxa were described by various European malacologists; e.g., Albers (1854a, 1854b) described new species on the basis of specimens collected by Warszewicz, Morelet (1860, 1863) used collections made by Léoncide Angrand, and Lubomirski (1880) based his descriptions on material from Jelski and Sztolcman. The 161 taxa mentioned in this paper are plotted against publication date in Fig. 1C. The period 1851–1865 stands out with 32 newly introduced taxa.

The aim of this paper is to compile data for part of the Orthalicoidea occurring in the study area, giving systematic information (original publication, type locality, type depository, summarizing literature) and distributional records. A photograph of a type specimen, if located, or an identified specimen by us are presented; if these were not available a copy of a picture from literature is provided.

## Methods

This compilation is based on literature (listed in Breure 1979, Breure and Schouten 1985, and recent papers; see Ramírez et al. 2003 for a name list of Peruvian Mollusca species), and distribution records with precise localities only from (unfortunately a limited number of) verified museum collections (Brussels, Leiden, London). These are listed for each taxon under distribution, the countries of the study area are indicated in bold. We have been reluctant in using unverified data (marked in the text with *) from online databases as misidentifications are possible for many taxa; only if relatively little doubt existed about (easily identifiable) species have these data been used as distribution records in the maps. Altitudes, if not given in the literature for the species, have been taken from Google Earth but should be treated as an estimate. Localities have been mapped with SimpleMappr (Shorthouse 2010), with terrestrial ecoregions layer (see also WWF 2015). The systematic part herein generally follows current understanding and is not to be considered a major revisionary work. Type localities are quoted from the original publication, unless stated otherwise. Diagnoses of supra-specific taxa are tailored to the study area; sizes mentioned (small/medium/large) are relative to the variation within the supra-specific taxon in this area.

Photographs are presented of at least the ventral view of a species and, only if they were available, of other views of the shell. In the legends the shell height (H) is given in millimeters. If available, living specimens are figured to facilitate recognition in the field, but generally pictures of living snails with verified identifications are very scarce.

The following abbreviations are used for depositories of material: ANSP, Academy of Natural Sciences, Philadelphia, U.S.A.; CMC, Cincinnati Museum Center, Cincinnati, U.S.A.; FML, Fundación Miguel Lillo, Tucumán, Argentina; MHNG, Muséum d’histoire naturelle, Genève, Switzerland; MIZW, Museum and Institute of Zoology, Polish Academy of Sciences, Warsaw, Poland; MNHN, Muséum national d’Histoire naturelle, Paris, France; NHMUK, Natural History Museum, London, U.K.; NMBE, Naturhistorisches Museum der Burgergemeinde Bern, Bern, Switzerland; NMW, National Museum of Wales, Cardiff, U.K.; RAMM, Royal Albert Memorial Museum, Exeter, U.K.; RBINS, Royal Belgian Institute of Natural Sciences, Brussels, Belgium; RMNH, Naturalis Biodiversity Center, Leiden, the Netherlands (formerly the Mollusca collection of Rijksmuseum van Natuurlijke Historie, Leiden); SMF, Senckenberg Natur-Museum, Frankfurt am Main, Germany; UF, Florida Museum of Natural History, Gainesville, U.S.A.; VMA, private collection V. Mogollón Avila, Lima, Peru; ZMA, Naturalis Biodiversity Center, Leiden, the Netherlands (formerly the Mollusca collection of Zoölogisch Museum, Amsterdam); ZMB, Zoologisches Museum, Humboldt Universität, Berlin, Germany; ZMUC, Statens Naturhistoriske Museum, Copenhagen, Denmark; ZSM, Zoologische Staatssammlung, München, Germany. The number of specimens (if known) is given after the registration number between brackets, unless it is a holotype or lectotype. In the sections on anatomy the following abbreviations are used between square brackets: d, digestive tract; g, genitalia; h, histology; k, kidney internal morphology; m, mandibula; n, nervous system; p, pallial organs; r, radula.

## Systematics

### Superfamily Orthalicoidea

Taxon classificationAnimaliaStylommatophora

#### Remarks.

The division into families follows the phylogenetic studies of Breure and Romero (2012). Within each family the genera and species are listed alphabetically.

#### Key to (sub)genera in the study area

**Table:** 

1	Shell thin	**2**
–	Shell rather solid	**3**
–	Shell solid	**4**
2	Base colour shell whitish or yellowish	**5**
–	Base colour shell tawny or brownish	**6**
3	Peristome thin	**7**
–	Peristome somewhat thick	**8**
–	Peristome thickened	**Porphyrobaphe (Porphyrobaphe)**
4	Peristome thin	**9**
–	Peristome somewhat thick	**10**
–	Peristome thickened	**11**
5	Suture ascending behind lip	**12**
–	Suture descending in front, ascending behind lip	**Plekocheilus (Eudolichotis)**
–	Suture not descending nor ascending in front	**13**
6	Apertural dentition absent	**14**
–	Apertural dentition present	***Cyclodontina***
7	Suture crenulated	15
–	Suture not crenulated	16
8	Suture crenulated	***Paeniscutalus***
–	Suture not crenulated	17
9	Suture crenulated	***Kara***
–	Suture not crenulated	***Corona***
10	Suture crenulated	**Thaumastus (Thaumastus)**
–	Suture not crenulated	**Sultana (Metorthalicus)**
11	Protoconch sculpture pit-reticulated	**Sultana (Trachyorthalicus)**
–	Protoconch sculpture with axial riblets, becoming more zigzag on the last part	**Sultana (Metorthalicus)**
12	Subsutural band present	***Clathrorthalicus***
–	Uniformly coloured	***Spixia***
13	Peristome whitish or light-coloured	**18**
–	Peristome dark coloured	**Sultana (Sultana)**
14	Shell sides straight	**Plekocheilus (Aeropictus)**
–	Shell sides slightly convex	**Plekocheilus (Eurytus)**
15	Basal margin of peristome regularly rounded	***Scholvienia***
–	Basal margin of peristome angled	***Quechua***
16	Groundcolour shell whitish or yellowish	**19**
–	Groundcolour shell tawny or brownish	**20**
17	Peristome not reflected	**Thaumastus (Thaumastiella)**
–	Peristome narrowly reflected	**Porphyrobaphe (Oxyorthalicus)**
18	Shell uniformly coloured	**Simpulopsis (Eudioptus)**
–	Shell colouration with spiral band(s) or waving or sinuous streaks	***Orthalicus***
19	Apertural dentition absent	***Corona***
–	Apertural dentition present	***Spixia***
20	Suture hardly impressed	**Plekocheilus (Plekocheilus)**
–	Suture well impressed	**Plekocheilus (Eurytus)**

### Family Amphibulimidae

Taxon classificationAnimaliaStylommatophoraAmphibulimidae

P. Fischer, 1873

Amphibulimidae P. Fischer 1873: 325.

### Genus Plekocheilus

Taxon classificationAnimaliaStylommatophoraAmphibulimidae

Guilding, 1828

Plekocheilus Guilding 1828: 532.

#### Type species.

*Caprella undulata* Guilding, 1824, by monotypy.

#### Diagnosis.

Shell (elongate-)globose to fusiform, rimate, rather thin to solid, height up to ca. 27–ca. 75 mm (study area). Colour light to darker (reddish-)brown, with dark axial zigzag streaks or oblique spiral series of spots. Surface smooth or malleate, in some species with cuticular cavities filled with air. Protoconch granulate or axially wrinkled. Whorls slightly convex, suture hardly to well impressed, descending in front. Aperture sub- to elongate-ovate. Peristome thickened, more or less expanded and reflexed. Columella in several species with a fold.

#### Distribution.

West Indies, Panama, Colombia, Ecuador, Peru, Bolivia, Brazil, French Guiana, Suriname, Guyana, Venezuela.

#### Anatomy.

Breure 1978: Plekocheilus (Aeropictus) calliostomus (Dohrn, 1882) [g, r], Plekocheilus (Aeropictus) delicatus (Pilsbry, 1935) [g, r], Plekocheilus (Aeropictus) dissimulans (Preston, 1909) [g, r], Plekocheilus (Aeropictus) veranyi (Pfeiffer, 1848) [g, r], Plekocheilus (Eudolichotis) aurissciuri (Guppy, 1866) [g, r], Plekocheilus (Eudolichotis) glaber (Gmelin, 1791) [g, r], Plekocheilus (Eurytus) ampullaroides (Mousson, 1873) [g, r], Plekocheilus (Eudolichotis) mundiperditi Haas, 1955 [g, r], Plekocheilus (Eurytus) piperitus (Sowerby I, 1837) [g, h, r], Plekocheilus (Eudolichotis) sophiae Breure, 2009 (as Plekocheilus (Plekocheilus) blainvilleanus linterae) [g]; Breure 2009: Plekocheilus (Eurytus) huberi Breure, 2009 [g], Plekocheilus (Eudolichotis) nebulosus Breure, 2009 [g], Plekocheilus (Eudolichotis) tatei Haas, 1955 [g]; Breure and Schlögl 2010: Plekocheilus (Eurytus) breweri Breure and Schlögl, 2010 [g], Plekocheilus (Plekocheilus) vlceki Breure and Schlögl, 2010 [g]; Breure 2013b: Plekocheilus (Eurytus) huberi Breure, 2009 [m, r], Plekocheilus (Eudolichotis) mundiperditi Haas, 1955 [m, r], Plekocheilus (Eudolichotis) nebulosus Breure, 2009 [m, r], Plekocheilus (Eudolichotis) tatei Haas, 1955 [m, r], Plekocheilus (Plekocheilus) vlceki Breure and Schlögl, 2010 [r]; Breure 2012b: Plekocheilus (Plekocheilus) philippei Breure, 2012 [g, p].

#### Phylogenetic data.

Breure et al. 2010: Plekocheilus (Plekocheilus) vlceki Breure and Schlögl, 2010; Breure 2013b: Plekocheilus (Aeropictus) succineoides (Petit de la Saussaye, 1840), Plekocheilus (Eudolichotis) glaber (Gmelin, 1791), Plekocheilus (Eurytus) breweri Breure and Schlögl, 2010, Plekocheilus (Eudolichotis) gibbonius (Lea, 1838), Plekocheilus (Eudolichotis) piperatoides Pilsbry, 1901; Breure and Romero 2012: Plekocheilus (Eurytus) breweri Breure and Schlögl, 2010.

#### Key to subgenera of Plekocheilus in the study area

**Table:** 

1	Shell surface with malleation or granulation	**2**
–	Shell surface with cuticular cavities filled with air	**Plekocheilus (Aeropictus)**
2	Shell elongate-ovate to fusiform, surface smooth, granulate or with spiral series of puckered bands	**3**
–	Shell (elongate-)globose, surface malleate and/or with axial riblets	**Plekocheilus (Plekocheilus)**
3	Aperture subovate, its basal margin rounded; columella simple or with a crescent-shaped channel	**Plekocheilus (Eurytus)**
–	Aperture narrowly-ovate, its basal margin rounded or produced, columella with a fold at the basal-parietal margin	**Plekocheilus (Eudolichotis)**

### Subgenus Plekocheilus (Aeropictus)

Taxon classificationAnimaliaStylommatophoraAmphibulimidae

Weyrauch, 1967

Plekocheilus (Aeropictus) Weyrauch 1967: 465.

#### Type species.

*Bulimus veranyi* Pfeiffer, 1848, by original designation.

#### Diagnosis.

Shell rather thin, spire short, surface with cuticular cavities filled with air, protonch finely granulated, aperture with well expanded lip.

#### Distribution.

Colombia, Ecuador, ?Peru, ?Brazil, Venezuela.

#### Habitat.

May be found in montane and cloud forest, and in páramos; occasionally in pockets of arid vegetation (e.g., *Opuntia* sp.). The vertical distribution is 1000–4000 m, with an emphasis on 2500–3000 m.

### Plekocheilus (Aeropictus) tenuissimus

Taxon classificationAnimaliaStylommatophoraAmphibulimidae

Weyrauch, 1967

Figs 2A–C , 14

Plekocheilus (Orcesiellus) tenuissimus Weyrauch 1967: 469, figs 23, 50.
Plekocheilus tenuissimus ; Richardson 1995: 323 (references).
Plekocheilus (Aeropictus) tenuissimus ; Breure and Borrero 2008: 7; Borrero and Breure 2011: 15, figs 5A–C; Breure 2012a: 12.

#### Type locality.

“Ecuador, Tandayapa, en la vertiente oriental del cerro Pichincha, approximadamento 2500 m”.

#### Type material.

FML 3364, holotype.

#### Diagnosis.

Shell relatively small, with hardly convex whorls, the height of the aperture 0.72 total shell height, suture descending in front, but sharply ascending behind the lip, parietal callus pale greenish-brown.

#### Dimensions.

Shell height 27.8, diameter 17.4 mm.

#### Distribution.

**Ecuador**, Prov. Pichincha, Tandayapa; ?Prov. Carchi, El Laurel (Breure and Borrero 2008).

#### Ecoregion.

Northwestern Andean montane forests [NT0145].

#### Remarks.

This species occurs on the western slope of the Andes in cloud forest. Fig. 85C–D is possibly a living specimen of this species, for which no voucher could be studied.

### Subgenus Plekocheilus (Eudolichotis)

Taxon classificationAnimaliaStylommatophoraAmphibulimidae

Pilsbry, 1896

Auris (Eudolichotis) Pilsbry 1896 [1895–1896]: 108.
Plekocheilus (Sparnotion) Pilsbry 1944c: 30 (**syn. n.**).

#### Type species.

*Bulimus distortus* Bruguière, 1789, by original designation.

#### Diagnosis.

Shell relatively medium-sized, fusiform, a papillose-granulose sculpture on the last whorl, the aperture elongate, with a produced pinkish lip, the columellar margin with a slight fold entering the aperture.

#### Distribution.

West Indies, Panama, Colombia, Peru, Brazil, French Guiana, Suriname, Guyana, Venezuela.

#### Habitat.

The species live mainly in arid conditions in the leaf litter layer of xerophytic shrub vegetation and in deciduous forests. The following, Peruvian species is an exception, living in rainforest.

#### Anatomy.

Araujo 1975b: Plekocheilus (Eudolichotis) lacertus (Pfeiffer, 1855) [g, m, p, r]; Breure 1978: Plekocheilus (Eudolichotis) aurissciuri (Guppy, 1866) [g, r], Plekocheilus (Eudolichotis) distortus (Bruguière, 1789) [r], Plekocheilus (Eudolichotis) glaber glaber (Gmelin, 1791) [g, r], Plekocheilus (Eudolichotis) glaber grenadensis (Guppy, 1868) [g, r].

#### Phylogenetic data.

Breure 2013b: Plekocheilus (Eudolichotis) glaber glaber (Gmelin, 1791), Plekocheilus (Eudolichotis) glaber grenadensis (Guppy, 1868).

#### Remarks.

The reasons for considering Plekocheilus (Sparnotion) Pilsbry, 1944 a junior subjective synonym are given below.

### Plekocheilus (Eudolichotis) hauxwelli

Taxon classificationAnimaliaStylommatophoraAmphibulimidae

(Crosse, 1872)

Figs 12A–F , 16

Bulimus hauxwelli Crosse 1872: 211; Crosse 1873: 252, pl. 11 fig. 2.

#### Type locality.

“in vicinio fluminis Ambiyacu, ad locum Pebas, Peruviae”.

#### Type material.

MCZ 202073 (1), paratype.

#### Diagnosis.

See above.

#### Dimensions.

Shell height 50.6, diameter 18.5 mm.

#### Distribution.

**Peru**, Dept. Loreto, Pebas, banks of río Ampiyacu.

#### Ecoregion.

Iquitos varzea [NT0128].

#### Remarks.

Crosse did not state on how many specimens his description was based, but said his material was based on “(Coll. Orton)”, and collected by John Hauxwell. The record of Breure (1979: 32) “HT MCZ” refers to specimen MCZ 202073 (“banks of Ambiyacu River near Pebas, Peru / Vassar College / James Orton”; see http://bit.ly/1fFP7xF), which was erroneous as Turner (1962) explained that in 1874 the type material—returned to Orton after the description by Crosse—has been transferred to the MCZ collection. Unfortunately, after Pilsbry used the holotype for his re-description, it “has since been misplaced or lost”; the MCZ specimen is thus a paratype, and was correctly mentioned as such by Breure (1978: 22). Pilsbry classified the species with his subgenus Plekocheilus (Eudolichotis) Pilsbry, 1896 and singled Plekocheilus (Eudolichotis) hauxwelli out in the key for the subgenus (Pilsbry 1896 [1895–1896]: 109), distinguishing it from Plekocheilus (Eudolichotis) distortus (Bruguière, 1789) and Plekocheilus (Eudolichotis) aurissciuri (Guppy, 1866) by having (1) a “minutely, densely but irregularly scattered, papillose” sculpture on the last whorl; (2) “longitudinal groups of crowded, finely zigzag hydrophanous lines” on the dorsal side of the last whorl (Pilsbry 1896 [1895-1896]: pl. 44 fig. 78); (3) a narrow, “not calloused” lip. Many years later, Pilsbry (1944c) referred to this characteristics presented in this key to define his new subgenus Plekocheilus (Sparnotion), with its sole species Plekocheilus (Simpulopsis) hauxwelli. This subgenus has been recognised by Zilch (1960: 476, fig. 1674), and Breure (1979: 32); Schileyko (1999: 277: fig. 334) expressed some doubt about its status by placing a question mark, but did not explicitly comment on this in his text.

The loss of the holotype of *Bulimus hauxwelli* makes it necessary to judge this taxon—and the subgenus *Sparnotion*—largely on the basis of the figures provided by Pilsbry and the remaining paratype in MCZ. As far as we know there is no material with proven locality data present in other museum collections. However, we recently had the opportunity to re-study the specimen in MCZ on the basis of high resolution pictures supplied by Adam Baldinger. As noted earlier (Breure 1978: 22), the paratype does not show the “longitudinal groups of crowded, finely zigzag hydrophanous lines” very clearly and this could hardly be compared to the subcuticular cavities filled with air characteristic for Plekocheilus (Aeropictus); see also Borrero and Breure 2011: fig. 6 for shell sculptures of several Plekocheilus species. While the paratype shell shows a papillose sculpture of the last whorl (unfortunately not clearly shown on the picture), we think this sculpture is not atypical compared to the known species of Plekocheilus (Eudolichotis). The sprout in the basal lip seems stronger than in Crosse’s or Pilsbry’s figures; this may be a sign for intra-specific variation. Finally, the narrow and ‘not calloused’ lip reminds of several Plekocheilus (Eurytus) species and we hardly doubt if this characteristic alone may be sufficient for a subgeneric separation of this species.

When this manuscript was being finalized, we received information about a specimen with locality “Peru” in the RAMM collection. This specimen originates from the collection of Miss J.E. Linter (1844–1909) and is the sole specimen we have been able to trace apart from the type material. This specimen (Figs 12D–F) does show the characteristics that Pilsbry mentioned for the lost holotype. And although there seems some mixing in of a characteristic—zigzag hydrophanous lines—from Plekocheilus (Aeropictus), based on the shell morphology alone we conclude that this species may be best classified as Plekocheilus (Eudolichotis) hauxwelli untill more material, hopefully allowing for anatomical and molecular studies, becomes available.

### Subgenus Plekocheilus (Eurytus)

Taxon classificationAnimaliaStylommatophoraAmphibulimidae

Albers, 1850

Eurytus Albers 1850: 169.

#### Type species.

*Helix pentadina* d’Orbigny, 1835, by subsequent designation (Albers 1860: 195).

#### Diagnosis.

Shell (elongate-)ovate, height up to ca. 35–ca. 75 mm (study area), colour brownish, usually with darker spots, arranged in axial streaks or oblique series, zigzags or irregularly spaced, whorls slightly convex, suture well impressed, aperture (elongate- or sub-) ovate, columellar margin usually entering with a slight fold above, peristome simple or slightly expanded and reflexed.

#### Distribution.

West Indies, Panama, Venezuela, Brazil, Bolivia, Peru, Ecuador, Colombia.

#### Habitat.

Species classified with this taxon fall into two groups: (a) occurring in lowland (rain)forests at altitudes up to ca. 1000 m, or (b) living in montane forests at ca. 1250–3500 m. The species may be found in leaf litter or on shrubs.

#### Remarks.

This subgenus is not mentioned by Schileyko (1999). See Figs 85A–B and 86G–H for unidentified living specimens from Ecuador.

#### Key to species in the study area

**Table:** 

1	Last whorl regularly rounded	**2**
–	Last whorl inflated or ‘hump-back’ shaped	**3**
2	Shell height / diameter ratio less than 2.0	**4**
–	Shell height / diameter ratio 2.0 or more	**5**
3	Shell height / diameter ratio less than 1.6	**6**
–	Shell height / diameter ratio 1.61–1.99	**7**
–	Shell height / diameter ratio 2.0 or more	***aristaceus***
4	Aperture shape ovate	**8**
–	Aperture shape elongate-ovate	**9**
5	Ratio aperture height / shell height less than 0.55	**10**
–	Ratio aperture height / shell height more than 0.55	**11**
6	Shell height less than 50 mm	***cardinalis***
–	Shell height 51–60 mm	***doliarius***
–	Shell height 60 mm or more	***jimenezi***
7	Aperture shape ovate	***taylorianus***
–	Aperture shape broadly ovate	***nocturnus***
8	Suture regularly descending in front	***lynciculus***
–	Suture rapidly descending in front	***piperitus***
–	Suture descending in front, ascending behind lip	***roseolabrum***
9	Ratio aperture height / shell height less than 0.65	***tricolor***
–	Ratio aperture height / shell height 0.66 or more	***eros***
10	Teleoconch sculptured with granulation	***onca***
–	Teleconch sculptured with spiral elements	***bruggeni***
11	Aperture shape ovate	***aureonitens***
–	Aperture shape elongate-ovate	***floccosus*, *superstriatus***

### Plekocheilus (Eurytus) aristaceus

Taxon classificationAnimaliaStylommatophoraAmphibulimidae

(Crosse, 1869)

Figs 8A–C , 14

Bulimus aristaceus Crosse 1869: 185; Crosse 1870: 105, pl. 6 fig. 5.
Plekocheilus aristaceus ; Richardson 1995: 302 (references).
Plekocheilus (Eurytus) aristaceus ; Breure and Borrero 2008: 5; Breure and Araujo 2015: 87, fig. 1.

#### Type locality.

“Quito, republica Aequatoris”.

#### Type material.

MNCN 15.05/7180, lectotype (Breure and Araujo 2015).

#### Diagnosis.

Shell relatively medium-sized, moderately solid, sculptured with granulation, a faint pattern of spiral bands on the last whorl, the interstices about as wide as the bands, last whorl inflated, suture deeply descending in front, peristome hardly expanded and reflexed.

#### Dimensions.

Shell height 48.3, diameter 22.7 mm.

#### Distribution.

**Ecuador**, Prov. Chimborazo, Bucay; Prov. Cotopaxi, Páramo de Sighos; Prov. Pichincha, Rio Pilaton (all Breure and Borrero 2008).

#### Remarks.

In the MNCN there are three lots labelled ‘*Bulimus aristaceus* Crosse’ which were previously considered as syntypes. These lots appeared not to be conspecific, and only lot MNCN 15.05/7180 is considered as type material of this taxon. This species was recently re-described and re-figured by Breure and Araujo (2015). The figure of Crosse (1870) does not entirely adequately represent the current state of the shell as the colour marks largely have faded away.

### Plekocheilus (Eurytus) aureonitens

Taxon classificationAnimaliaStylommatophoraAmphibulimidae

(Miller, 1878)

Fig. 9A

Eurytus aureonitens Miller 1878: 181; Miller 1879: pl. 6 fig. 2.
Plekocheilus aureonitens ; Richardson 1995: 303 (references).
Plekocheilus (Eurytus) aureonitens ; Breure and Borrero 2008: 5.

#### Type locality.

[Ecuador] “Valli Pilatonensi”.

#### Type material.

Not located.

#### Diagnosis.

Shell relatively medium-sized, rather thin, sculptured with fine granulation, the last whorl nearly smooth, suture deeply descending in front, columella twisted (Pilsbry 1895 [1895–1896]).

#### Dimensions.

Shell height 53, diameter 25 mm.

#### Distribution.

**Ecuador**, Prov. Pichincha, Río Pilatón valley.

#### Ecoregion.

Northwestern Andean montane forests [NT0145].

#### Remarks.

This species is only known from the type locality at an altitude of 1000 m, and may prove to be a synonym of Plekocheilus (Eurytus) taylorianus (Reeve, 1849).

### Plekocheilus (Eurytus) bruggeni

Taxon classificationAnimaliaStylommatophoraAmphibulimidae

Breure, 1978

Figs 2E–G , 14

Plekocheilus (Eurytus) bruggeni Breure 1978: 9, pl. 6 figs 5–7; Richardson 1995: 306 (references); Ramírez et al. 2003: 281; Breure and Ablett 2011: 17, figs 18D–F, 18ii.

#### Type locality.

“Peru, Dept. Pasco, Huancabamba”.

#### Type material.

NHMUK 1911.11.2.88, holotype.

#### Additional material.

Paratypes NHMUK 1911.11.2.89–90 (2), RMNH 55122 (1).

#### Diagnosis.

Shell relatively medium-sized, rather solid, colour light brown with irregular reddish-brown dots, surface with numerous cutical spiral striae, suture somewhat descending in front, aperture elongate-ovate, peristome thin and simple.

#### Dimensions.

Shell height 39.0, diameter 19.5 mm.

#### Distribution.

**Peru**, Dept. Pasco, Huancabamba.

#### Remarks.

This taxon is only known from the type locality.

### Plekocheilus (Eurytus) cardinalis

Taxon classificationAnimaliaStylommatophoraAmphibulimidae

(Pfeiffer, 1853)

Figs 2D , 14

Bulimus cardinalis Pfeiffer 1853: 316.
Plekocheilus cardinalis ; Richardson 1995: 306 (references).
Plekocheilus (Plekocheilus) cardinalis ; Köhler 2007: 127, fig. 2; Breure and Borrero 2008: 5.
Plekocheilus (Eurytus) cardinalis ; Borrero and Breure 2011: 44, figs 14A, 15E–F.

#### Type locality.

[Ecuador] “Quito”.

#### Type material.

ZMB 112721 (1), syntype.

#### Diagnosis.

Shell relatively medium-sized, rather solid, last whorl inflated, surface sculptured with strong, axial and oblique criss-crossing sections, suture somewhat descending in front and slightly ascending behind lip, aperture round-ovate.

#### Dimensions.

Shell height 46, diameter 32 mm.

#### Distribution.

Colombia (Borrero and Breure 2011). **Ecuador**, Prov. Napo, Nachiyacu; ibid., Topo; Prov. Pastaza, Mera; ibid., Puyo; Prov. Pichincha, Milpe; ibid., near Mindo; ibid., Nanegal (Breure and Borrero 2008, Borrero and Breure 2011).

#### Ecoregion.

Northwestern Andean montane forests [NT0145].

#### Remarks.

This species has been found at ca. 1000–1250 m altitude. The reference of Borrero and Breure (2011) to a lectotype designation by Köhler is erroneous.

### Plekocheilus (Eurytus) doliarius

Taxon classificationAnimaliaStylommatophoraAmphibulimidae

(da Costa, 1898)

Figs 3A–B , 14

Strophocheilus (Eurytus) doliarius da Costa 1898: 84, fig. 1; Neubert and Janssen 2004: 208, pl. 1 fig. 1; Breure and Ablett 2011: 19, figs 16D–E, 16ii.
Plekocheilus doliarius ; Richardson 1995: 310 (references).
Plekocheilus (Eurytus) doliarius ; Breure and Borrero 2008: 5; Borrero and Breure 2011: 45, figs 15I–J.

#### Type locality.

“Paramba, Ecuador”.

#### Type material.

NHMUK 1907.11.21.110, lectotype (Breure 1979: 30).

#### Additional material.

SMF 9513 (1), paralectotype.

#### Diagnosis.

Shell relatively medium-sized, solid, last whorl very inflated, sculpture malleated, suture somewhat descending in front and slightly ascending behind lip, aperture ear-shaped (broadly ovate), peristome expanded and slightly reflexed.

#### Dimensions.

Shell height 58.0, diameter 41.5 mm.

#### Distribution.

Colombia (Borrero and Breure 2011). **Ecuador**, Prov. Carchi, Hacienda Paramba.

#### Ecoregion.

Northwestern Andean montane forests [NT0145].

### Plekocheilus (Eurytus) eros

Taxon classificationAnimaliaStylommatophoraAmphibulimidae

(Angas, 1878)

Fig. 2H–J

Bulimus (Eurytus) eros Angas 1878: 312, pl. 18 figs 6–7; Breure and Ablett 2011: 20, figs 20D–F, 20ii.
Plekocheilus eros ; Richardson 1995: 310 (references).
Plekocheilus (Eurytus) eros ; Breure and Borrero 2008: 5.

#### Type locality.

“Ecuador”.

#### Type material.

NHMUK 1879.1.21.2, lectotype (Breure 1979: 30).

#### Diagnosis.

Shell relatively medium-sized, rather thin, surface densely and evenly granulate, suture somewhat descending in front and sharply ascending behind lip, aperture ovate, peristome expanded and narrowly reflexed.

#### Dimensions.

Shell height 35.5, diameter 18.5 mm.

#### Distribution.

**Ecuador**, Prov. Loja, ? Chaguarpamba (MIZW).

#### Remarks.

A full re-description, based on the lectotype, was given by Breure (1978: 11). The first precise locality for this species is based on two specimens found in the MIZW collection; the material is accompanied by an original label “Chaguarpata (5800´) [1768 m]”, and was collected in 1883. Modern gazetteers do not provide any exact name like this for Ecuador; the closest match is Chaguarpamba in Prov. Loja, where altitudes in that range do occur. The (more recent, second) label “Chaguaspata, Peru” seems to be in error as there is no such place in Peru. The specimens have a white lip instead of the pink one in the type specimen.

### Plekocheilus (Eurytus) floccosus

Taxon classificationAnimaliaStylommatophoraAmphibulimidae

(Spix in Wagner, 1827)

Figs 3C–E , 13A–B , 14 , 88B

Achatina floccosa Spix in Wagner 1827: 10, pl. 9 figs 3–4.
Helix pentadina d’Orbigny 1835: 8.
Bulimus lacrimosus Heimburg 1884: 92; Heimburg 1887: 1, pl. 1 fig. 1.
Plecocheilus pentadinus ; Zischka 1953: 78.
Plekocheilus floccosus ; Richardson 1995: 311 (references, synonymy).
Plekocheilus (Eurytus) floccosus ; Ramírez et al. 2003: 281.
Plekocheilus (Eurytus) lacrimosus ; Ramírez et al. 2003: 281.

#### Type locality.

“sylvis Provinciarum septemtrionalium Brasiliae”.

#### Type material.

ZSM 20020116 (1), syntype.

#### Additional material.

MNHN 28258, holotype of *Helix pentadina* d’Orbigny.

#### Diagnosis.

Shell relatively large, sculptured with closely and coarsely, partly bifurcating plicae, and dense granulation; suture hardly descending in front, aperture elongate-ovate, peristome narrowly expanded below.

#### Dimensions.

Shell height 60.0, diameter 27.4 mm.

#### Distribution.

**Ecuador**, Prov. Napo, 120 km SE Quito (Weyrauch 1967). **Peru**, Dept. San Martin, Tingo Maria; ibid., Pucallpa; ibid., Yarinacocha (Breure 1978). **Bolivia**, Dept. Cochabamba, Prov. Chaparé. Brazil (Simone 2006).

#### Ecoregion.

Eastern Cordillera real montane forests [NT0121], Southwest Amazon moist forests [NT0166].

#### Remarks.

The apex of the syntype is damaged, so the total shell height is slightly over 60 mm. The type locality is very imprecise and covers a large area. This taxon has been synonymised with *Helix pentadina* d’Orbigny, 1835 (described from central Bolivia) and *Bulimus lacrimosus* Heimburg, 1884 (from Peru, Dept. Loreto) by Weyrauch (1967: 462) without further comments. We concur with his opinion on the former, with the notice that *Helix pentadina* was described by d’Orbigny on the basis of one damaged shell from Bolivia, Prov. Chaparé. The type material of Heimburg’s taxon has not been located; therefore it remains difficult to fully asses this species, but his figure leaves little doubt. Richardson (1995) agreed with both synonymizations of Weyrauch, and we provisionally follow this conclusion. However, this species deserves further studies given the very large distribution range.

### Plekocheilus (Eurytus) jimenezi

Taxon classificationAnimaliaStylommatophoraAmphibulimidae

(Hidalgo, 1872)

Figs 10C–F , 14

Bulimus gibbonius Hidalgo 1870: 54. Not Bulimus gibbonius Lea, 1838.
Bulimus jimenezi Hidalgo 1872: 93, pl. 5 figs 2–3.
Plekocheilus jimenezi ; Richardson 1995: 315 (references, synonymy [partly]).
Plekocheilus (Eurytus) jimenezi ; Breure and Borrero 2008: 6; Borrero and Breure 2011: 43, figs 15A–B.

#### Type locality.

[Ecuador] “San José”.

#### Type material.

MNCN 15.05/1066 (2), MNCN 15.05/3158 (2), syntypes.

#### Diagnosis.

Shell relatively large, solid, with oblique series of more or less spirally arranged reddish-brown spots, evenly and dense granulation, suture somewhat descending in front and slightly ascending behind lip, aperture broadly ovate, peristome narrowly expanded and reflexed.

#### Dimensions.

Shell height 74.9, diameter 48.4 mm.

#### Distribution.

**Ecuador**, Prov. Napo, Nachiyacu; ibid., Sarayacu (see Weyrauch 1967: 463); ibid., valley of Río Quijos; Prov. Orellana, San José de Suno; Prov. Pastaza, Puyo; ibid., Mera (Breure and Borrero 2008).

#### Ecoregion.

Eastern Cordillera real montane forests [NT0121].

#### Remarks.

The type locality is—according to Weyrauch (1967: 463)—located 50 km E Baeza; this points to Prov. Orellana, San José de Suno, also known as San José Viejo. *Bulimus gibbonius* Hidalgo, 1870 has the same type locality and is, following the opinion of Pilsbry (Pilsbry 1895 [1895–1896]: 87), a subjective synonym of *Bulimus jimenezi* Hidalgo, 1872.

### Plekocheilus (Eurytus) lynciculus

Taxon classificationAnimaliaStylommatophoraAmphibulimidae

(Deville & Hupé, 1850)

Figs 11A–C , 15

Bulimus lynciculus Deville and Hupé 1850: 640, pl. 15 fig. 1.
Plekocheilus (Eurytus) jacksoni Pilsbry 1939: 1, fig. 2.
Plekocheilus lynciculus ; Richardson 1995: 316 (references, synonymy).
Plekocheilus (Eurytus) lynciculus ; Breure and Borrero 2008: 6; Borrero and Breure 2011: 30, figs 14C, 17C–D.

#### Type locality.

“Mission de Sarayacu, sur les bords de la rivière de l’Ucuyali, Pérou”.

#### Type material.

Not located.

#### Additional material.

ANSP 170694, holotype of Plekocheilus (Eurytus) jacksoni Pilsbry.

#### Diagnosis.

Shell relatively medium-sized, with dots and longitudinal streaks of (reddish-)brown, sculptured with impressed spiral grooves crossing the growth striae, suture descending in front, aperture ovate, peristome slightly expanded.

#### Dimensions.

Not given; (*jacksoni* Pilsbry) shell height 45.4, diameter 25.7 mm.

#### Distribution.

Colombia (Borrero and Breure 2011). **Ecuador**, Prov. Napo, Nachiyacu; Prov. Tungurahua, Rio Pastaza watershed. **Peru**, Dept. Loreto, Sarayacu.

#### Ecoregion.

Eastern Cordillera real montane forests [NT0121], Iquitos varzea [NT0128].

#### Remarks.

The sentence “sur les bords de la rivière de l’Ucuyali” leaves little doubt about the type locality, although there is also a locality named Sarayacu in Ecuador, Prov. Pastaza. We have been unable to locate the type specimens of Deville and Hupé, but close examination of their original figure leads us to believe that the shell exhibits the same longitudinal plication as seen on the holotype of *jacksoni* Pilsbry. We now tentatively consider the specimens figured by Borrero and Breure (2011: figs 17G–J, as Plekocheilus (Eurytus) piperitus) to be conspecific with Deville and Hupé’s species.

### Plekocheilus (Eurytus) nocturnus

Taxon classificationAnimaliaStylommatophoraAmphibulimidae

Pilsbry, 1939

Figs 11D–F , 15

Plekocheilus nocturnus Pilsbry 1939: 3, fig. 5; H.B. Baker 1963: 229; Richardson 1995: 317 (references).
Plekocheilus (Eurytus) nocturnus ; Breure and Borrero 2008: 6; Borrero and Breure 2011: 30, figs 16A–F.

#### Type locality.

“Ecuador, Puyo”.

#### Type material.

ANSP 170695, lectotype (Baker 1963: 229).

#### Diagnosis.

Shell rather solid, last whorl inflated, sculptured with growth wrinkles and very minute, low granulation, suture descending in front but flattened behind the lip, aperture ovate, peristome expanded and narrowly reflexed.

#### Dimensions.

Shell height 51.0, diameter 30.6 mm.

#### Distribution.

**Ecuador**, Prov. Imbabura, Ibarra; Prov. Napo, Topo; Prov. Pastaza, Mera; ibid., Puyo.

#### Ecoregion.

Eastern Cordillera real montane forests [NT0121], Northwestern Andean montane forests [NT0145].

### Plekocheilus (Eurytus) oligostylus

Taxon classificationAnimaliaStylommatophoraAmphibulimidae

Pilsbry, 1939

Figs 10A–B , 14

Plekocheilus oligostylus Pilsbry 1939: 3, fig. 6.
Plekocheilus jimenezi ; Richardson 1995: 315 (references, excl. synonymy).
Plekocheilus (Eurytus) jimenezi oligostylus ; Breure and Borrero 2008: 6.

#### Type locality.

“Colombia”, see remarks.

#### Type material.

ANSP 170696, lectotype (Baker, 1963).

#### Diagnosis.

Shell relatively large, solid, with oblique series of more or less spirally arranged reddish-brown spots, evenly and dense granulation, suture somewhat descending in front, aperture ovate.

#### Dimensions.

Shell height 71.0, diameter 47.0 mm.

#### Distribution.

Ecuador, Prov. Napo, Nachiyacu; ibid., Sarayacu; ibid., valley Río Quijos; Prov. Pastaza, Puyo; ibid., Mera (all Breure and Borrero 2008).

#### Ecoregion.

Eastern Cordillera real montane forests [NT0121].

#### Remarks.

Clench and Turner (1962: 109) have pointed out that the original locality was erroneous; the type locality should be Nachiyacu. This taxon has been synonymized by Richardson (1995) with Plekocheilus (Eurytus) jimenezi, but without any comments. The shape of the aperture in the type specimen may not be entirely typical; it does not show the ascending suture behind the lip typical of Plekocheilus (Eurytus) jimenezi, and the aperture is different in shape. Tentatively we have retained this taxon as a separate species, as it may be sympatric with Plekocheilus (Eurytus) jimenezi, awaiting further studies in the area.

### Plekocheilus (Eurytus) onca

Taxon classificationAnimaliaStylommatophoraAmphibulimidae

(d’Orbigny, 1835)

Figs 6A–C , 15

Helix onca d’Orbigny 1835: 8; Breure and Ablett 2011: 25, figs 19A–C, 19i.
Plecocheilus onca ; Zischka 1953: 78.
Plekocheilus onca ; Richardson 1995: 317 (references).

#### Type locality.

[Bolivia] “non loin…de Tutulima” (d’Orbigny 1837 [1834–1847]: 295).

#### Type material.

NHMUK 1854.12.4.120, lectotype (Breure and Ablett 2011: 26).

#### Additional material.

NHMUK 1854.12.4.120 (3), paralectotypes.

#### Diagnosis.

Shell relatively large, slender, with irregularly spaced reddish-brown spots, sculptured with dense and fine granulation, suture descending in front, aperture oblique elongate-ovate, peristome simple.

#### Dimensions.

Shell height 66.5, diameter 25.9 mm.

#### Distribution.

**Bolivia**, Dept. Cochabamba, near Totolima.

#### Ecoregion.

Bolivian Yungas [NT0105].

#### Remarks.

This species is very similar to Plekocheilus (Eurytus) floccosus (Spix in Wagner, 1827), but is decidedly more slender. The reference to non-Bolivian localities (Cousin 1887: 207) needs to be viewed with much suspicion as likely a misidentification may be involved.

### Plekocheilus (Eurytus) piperitus piperitus

Taxon classificationAnimaliaStylommatophoraAmphibulimidae

(Sowerby I, 1837)

Figs 4A–F , 5A , 15

Bulinus piperitus Sowerby I 1837 [1832–1841]: 8, fig. 93; Reeve 1848 [1848–1850]: pl. 16 fig. 96; Breure and Ablett 2011: 28, 20A–C, 20i.
Bulimus pseudopiperatus J. Moricand 1858: 451, pl. 14 fig. 2.
Plekocheilus piperitus ; Richardson 1995: 318 (references, synonymy).
Plekocheilus (Eurytus) piperitus ; Köhler 2007: 127, fig. 4; Borrero and Breure 2011: 48, figs 17G–J [partim].

#### Type locality.

[Peru] “Huallaga”.

#### Type material.

NHMUK 1975329, lectotype (**design. n.**) and paralectotype.

#### Additional material.

ZMB 112724 (1), paralectotype; MHNG-INVE-55493 (1), syntype of *Bulimus pseudopiperatus* J. Moricand.

#### Diagnosis.

Shell relatively medium-sized, with irregularly spaced reddish-brown dots, sometimes forming longitudal streaks, sculptured with a regular pattern of granulation (Fig. 5A), suture descending in front, rapidly descending behind the lip, aperture ovate, peristome simple.

#### Dimensions.

Shell height 55.8, diameter 31.3 mm.

#### Distribution.

**Peru**, Dept. San Martin, along Río Huallaga; ibid., Moyobamba; Dept. Ucayali, Pucallpa (Weyrauch 1967: 464).

#### Ecoregion.

Ucayali moist forests [NT0174].

#### Remarks.

Borrero and Breure (2011) identified Ecuadorian material as this species, without having seen the type material. However, the type material of Sowerby is somewhat tapering at base and has the suture descending in front (Fig. 4B–C); also the shells figured by Borrero and Breure seem slightly smaller and slenderer. Therefore we are of the opinion that this taxon is best restricted to Peruvian material, and therefore the specimen figured by Breure and Ablett (2011: fig. 20A–C) is now designated lectotype (**design. n.**). *Bulimus pseudopiperatus* J. Moricand, 1858 is considered a junior subjective synonym of *Bulinus piperitus* Sowerby I, 1837. This was also the opinion of Weyrauch (1967), who provided Pucallpa as the first precise locality for this species.

### Plekocheilus (Eurytus) piperitus mcgintyi

Taxon classificationAnimaliaStylommatophoraAmphibulimidae

‘Pilsbry’ H.B. Baker, 1963 stat. n.

Figs 5B–E , 15

Plekocheilus mcgintyi Pilsbry 1944a: pl. 9 fig. 6. Nomen nudum.
Plekocheilus mcgintyi ‘Pilsbry’ H.B. Baker 1963: 229; Richardson 1995: 316 (references).
Plekocheilus (Eurytus) mcgintyi ; Breure and Borrero 2008: 6.
Plekocheilus (Eurytus) piperitus ; Borrero and Breure 2011: 48, figs 17G–J [partim].

#### Type locality.

“Rio Napo, northeastern boundary of Ecuador”.

#### Type material.

ANSP 227455 (1), possible syntype.

#### Diagnosis.

Shell relatively medium-sized, with longitudinal streaks reddish-brown dots, some dots irregularly spaced in between, sculptured with a regular pattern of granulation (Fig. 5B), suture somewhat descending in front, rapidly descending behind the lip, aperture ovate, peristome hardly expanded and reflexed.

#### Dimensions.

Shell height 56.8, diameter 29.5 mm.

#### Distribution.

**Ecuador**, Prov. Napo, Río Jatunyacu [= Río Napo].

#### Ecoregion.

Eastern Cordillera real montane forests [NT0121].

#### Remarks.

This taxon has been considered as a separate species. Upon comparing the type with material of Plekocheilus (Eurytus) piperitus (Sowerby I, 1837), we conclude that *mcgyntyi* is very similar and consider it herein as subspecies of Sowerby’s taxon (**stat. n.**). The differences seem to consist mainly in Plekocheilus (Eurytus) piperitus mcgyntyi having a more expanding lip, and a somewhat slenderer shell. The Ecuadorian material mentioned by Borrero and Breure (2011) is now tentatively considered to be this subspecies.

### Plekocheilus (Eurytus) piperitus prodeflexus

Taxon classificationAnimaliaStylommatophoraAmphibulimidae

Pilsbry, 1895 stat. n.

Figs 7E–H , 16

Strophocheilus superstriatus var. prodeflexus Pilsbry 1895 [1895–1896]: 91, pl. 36 fig. 81; H.B. Baker 1963: 230.
Plekocheilus (Eurytus) superstriatus prodeflexus ; Ramírez et al. 2003: 281.

#### Type locality.

“Balsas, valley of Maranon R., Peru”.

#### Type material.

ANSP 66439, lectotype (Baker 1963: 230).

#### Diagnosis.

Shell relatively medium-sized, with irregularly spaced reddish-brown dots, sometimes forming longitudal streaks, sculptured with a regular pattern of granules (Fig. 7H), suture descending in front, rapidly descending behind the lip, aperture ovate, peristome simple.

#### Dimensions.

Shell height 52.0, diameter 30.0 mm.

#### Distribution.

**Peru**, Dept. Amazonas, Balsas.

#### Ecoregion.

Marañon dry forests [NT0223].

#### Remarks.

This taxon was described as a variety of Plekocheilus (Eurytus) superstriatus (Sowerby III, 1890). Upon comparing the type specimens we see differences in the shell shape, the sculpture of the last whorl at dorsal side (recognizing that Pilsbry’s shell is somewhat worn), and the dimensions. Moreover *prodeflexus* Pilsbry has a descending suture in front. This taxon appears related to both Plekocheilus (Eurytus) piperitus piperitus (Sowerby I, 1837) and to Plekocheilus (Eurytus) piperitus mcgintyi ‘Pilsbry’ H.B. Baker, 1963, sharing characteristics with both; the differences are but slight and seem to lie mainly in the sculpture of the last whorl. However, since the type specimen is worn, additional material from that area should clarify the possible variation. Tentatively we give it a subspecific status as Plekocheilus (Eurytus) piperitus prodeflexus (Pilsbry, 1895) (**stat. n.**).

### Plekocheilus (Eurytus) roseolabrum

Taxon classificationAnimaliaStylommatophoraAmphibulimidae

(E.A. Smith, 1877)

Figs 6G–I , 16

Bulimus roseolabrum E.A. Smith 1877: 362, pl. 39 fig. 8.
Plekocheilus roseolabrus [sic]; Richardson 1995: 320 (references).
Plekocheilus (Eurytus) roseolabrum ; Breure and Borrero 2008: 6; Borrero and Breure 2011: 44, figs 13G–I; Breure and Ablett 2011: 36, figs 22D–F, 22ii.

#### Type locality.

“Malacatos, South Ecuador”.

#### Type material.

NHMUK 1975135, lectotype (Breure 1978: 16).

#### Additional material.

NHMUK 1877.3.28.2 (1), paralectotype.

#### Diagnosis.

Shell relatively medium-sized, sculptured with granulose striae, suture somewhat descending in front, aperture ovate, peristome narrowly expanded and decidedly reflexed.

#### Dimensions.

Shell height 42.0, diameter 22.5 mm.

#### Distribution.

**Ecuador**, Prov. Loja, Malacatos.

#### Ecoregion.

Eastern Cordillera real montane forests [NT0121].

#### Remarks.

So far this species has not been re-found after its original description. The record from Prov. Zamora-Chinchipe, Tapichalaca (Breure and Borrero 2008) refers to a similar but as yet undescribed species.

### Plekocheilus (Eurytus) superstriatus

Taxon classificationAnimaliaStylommatophoraAmphibulimidae

(Sowerby III, 1890)

Figs 7A–D , 16

Bulimus superstriatus Sowerby III 1890: 578, pl. 56 fig. 9; Breure and Ablett 2011: 40, figs 23A–C, 23i.
Plekocheilus superstriatus ; Richardson 1995: 322 (references, synonymy).
Plekocheilus (Eurytus) superstriatus superstriatus ; Ramírez et al. 2003: 281.

#### Type locality.

[Peru] “Yquitos, Peruviae”.

#### Type material.

NHMUK 1889.11.19.1, lectotype (Breure 1978: 16).

#### Diagnosis.

Shell relatively large, with longitudinally plicae and granulation, the latter especially on the last whorl, suture hardly descending in front, aperture elongate-ovate, peristome narrowly expanded below.

#### Dimensions.

Shell height 64.5, diameter 31.0 mm.

#### Distribution.

**Peru**, Dept. Loreto, Iquitos.

#### Ecoregion.

Iquitos varzea [NT0128].

#### Remarks.

This species shows the same colour pattern and general shell shape as Plekocheilus (Eurytus) floccosus (Spix in Wagner, 1827), and is evidently closely allied to this species. Further studies should clarify the relationships between these species.

### Plekocheilus (Eurytus) taylorianus

Taxon classificationAnimaliaStylommatophoraAmphibulimidae

(Reeve, 1849)

Figs 5F , 6D–F , 9B , 15

Bulimus taylorianus Reeve 1849 [1848–1850]: pl. 81 fig. 602; Breure and Ablett 2011: 42, figs 24A–C, 24i.
Eurytus taylorioides minor Miller 1878: 181, pl. 4 fig. 1; Miller 1879: pl. 7 fig. 1.
Plekocheilus taylorianus ; Richardson 1995: 322 (references, synonymy).
Plekocheilus (Eurytus) taylorianus ; Köhler 2007: 127, fig. 5; Breure and Borrero 2008: 7; Borrero and Breure 2011: 42, figs 15C–D.

#### Type locality.

[Ecuador] “Environs of Quito”.

#### Type material.

NHMUK 1874.12.11.271, lectotype (Breure 1978: 16).

#### Diagnosis.

Shell relatively large, with reddish-brown oblique zigzags on the penultimate whorl, becoming irregularly spaced dots on the last whorl which is sculptured with fine granulation (Fig. 6F), suture descending in front, rapidly descending behind lip, aperture ovate, peristome simple.

#### Dimensions.

Shell height 58.5, diameter 31.0 mm.

#### Distribution.

**Ecuador**, Prov. Chimborazo, Mt. Chimborazo; Prov. Cotopaxi, Sigchos; Prov. Imbabura, Ibarra; Prov. Napo, Nachiyacu; Prov. Pastaza, Mera; ibid., Puyo; Prov. Pichincha, Nanegal; ibid., Pacto; ibid., Pintag; ibid., Gualea; Prov. Tungurahua, Topo (all Breure and Borrero 2008).

#### Ecoregion.

Northwestern Andean montane forests [NT0145].

#### Remarks.

Pilsbry 1895 [1895–1896]: 88 regarded this species as closely resembling Plekocheilus (Eurytus) piperitus (Sowerby I, 1837), with which we concur. Also Plekocheilus (Eurytus) roseolabrum (E.A. Smith, 1877) may be added to this group. Plekocheilus (Eurytus) taylorianus differs mainly in its larger size and the fine granulation on the last whorl (Fig. 5F).

### Plekocheilus (Eurytus) tricolor

Taxon classificationAnimaliaStylommatophoraAmphibulimidae

(Pfeiffer, 1853)

**Figure 2.**
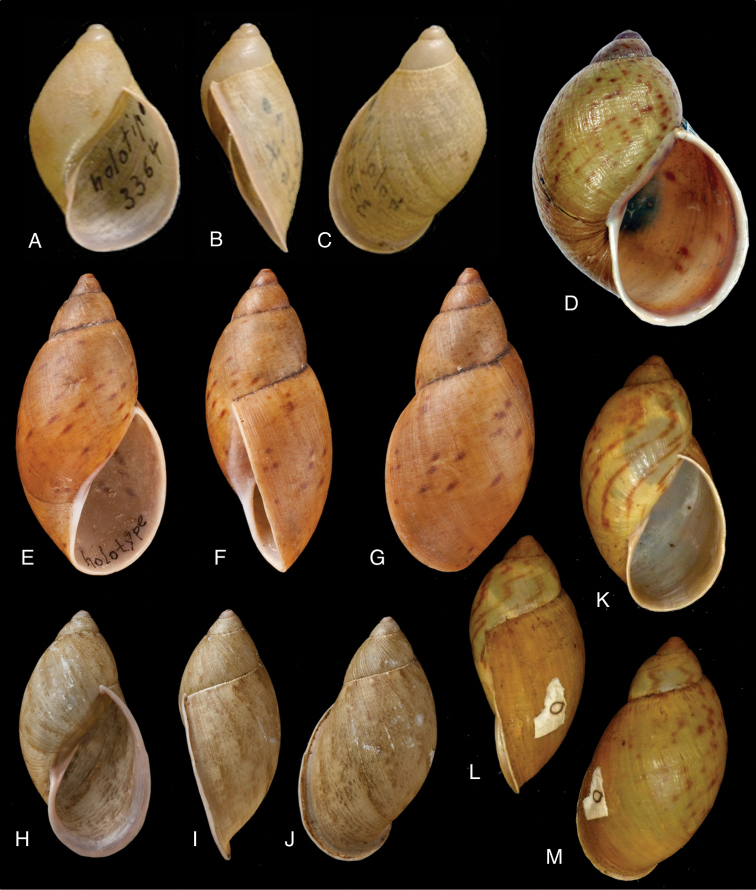
Plekocheilus species. **A–C** Plekocheilus (Aeropictus) tenuissimus Weyrauch, 1967, holotype FML 3364 (H = 27.8) **D** Plekocheilus (Eurytus) cardinalis (Pfeiffer, 1853), syntype ZMB 112721 (H = 46.0) **E–G** Plekocheilus (Eurytus) bruggeni Breure, 1978, holotype NHMUK 1911.11.2.88 (H = 39.0) **H–J** Plekocheilus (Eurytus) eros (Angas, 1878), lectotype NHMUK 1879.1.21.2 (H = 35.5) **K–M** Plekocheilus (Eurytus) tricolor (Pfeiffer, 1853), lectotype of *Bulimus semipictus* Hidalgo MNHN 28113 (H = 37.7).

Figs 2K–M , 13C–D , 16

Bulimus tricolor Pfeiffer 1853: 325; Pfeiffer 1853 in Küster and Pfeiffer 1840–1865: 95, pl. 32 figs 17–18.
Bulimus semipictus Hidalgo 1869: 188.
Plekocheilus tricolor ; Richardson 1995: 323 (references, synonymy).
Plekocheilus (Eurytus) tricolor ; Breure and Borrero 2008: 7; Borrero and Breure 2011: 43, figs 17A–B.

#### Type locality.

“Gualea, Neu Granada”.

#### Type material.

Not located.

#### Additional material.

MHNH 28113, lectotype (Breure 1975: 1139); MNCN 15.05/3161 (2) and 15.05/6943 (6), paralectotypes of *Bulimus semipictus* Hidalgo.

#### Diagnosis.

Shell relatively medium-sized, with reddish-brown oblique zigzags on the penultimate and last whorl, becoming irregularly spaced dots on the dorsal side of last whorl which is sculptured with longitudinal striae and finely impressed spiral lines, resulting in coarse, oblong granules; suture regularly descending in front, peristome narrowly expanded and reflexed.

#### Dimensions.

Shell height 37.7, diameter 21.6 mm (*semipictus* Hidalgo).

#### Distribution.

**Ecuador**, Prov. Bolivar, N of Bucay; Prov. Cotopaxi, Sigchos; Prov. Imbabura, Ibarra; Prov. Los Rios, Cerro Samana; Prov. Napo, Beaza; Prov. Pichincha, Santo Domingo de las Colorados; Prov. Tungurahua, Topo (all Breure and Borrero 2008).

#### Ecoregion.

Eastern Cordillera real montane forests [NT0121], Napo moist forests [NT0142], Northwestern Andean montane forests [NT0145].

#### Remarks.

Of the two taxa mentioned only type material of the junior subjective synonym *Bulimus semipictus* Hidalgo has been located.

### Subgenus Plekocheilus (Plekocheilus)

Taxon classificationAnimaliaStylommatophoraAmphibulimidae

#### Diagnosis.

Shell relatively medium-sized, with axial colour streaks of reddish-brown, partly oblique and zigzag, sculptured with axial riblets, becoming malleated on the last whorl and with a dense pattern of oblong granules behind the lip; aperture ovate, peristome expanded and reflexed.

#### Distribution.

West Indies, Colombia, Ecuador, Guyana, Venezuela.

#### Habitat.

The species live in montane and cloud forest in leaf litter, at altitudes of ca. 900–3350 m; the ecology within the study area is unknown.

### Plekocheilus (Plekocheilus) cecepeus

Taxon classificationAnimaliaStylommatophoraAmphibulimidae

Breure & Araujo, 2015

Figs 8D–F

Plekocheilus (Plekocheilus) cecepeus Breure and Araujo 2015: 89, fig. 2.

#### Type locality.

“Ecuador, Quito”.

#### Type material.

MNCN 15.05/60013H, holotype.

#### Additional material.

MNCN 15.05/60013P (5), MNCN 15.05/7477P (3), paratypes.

#### Diagnosis.

See above.

#### Dimensions.

Shell height 44.8, diameter 25.3 mm.

#### Distribution.

**Ecuador**, without precise locality.

#### Remarks.

This species was described on the basis of material collected by the Comisión Científica del Pacífico with an imprecise locality. While more precise records are awaited, it is suggested that the eastern Cordillera could be a possible location where this species might occur.

### Family Megaspiridae

Taxon classificationAnimaliaStylommatophoraMegaspiridae

Pilsbry, 1904

Megaspiridae Pilsbry 1904 [1903–1904]: 175.

#### Remarks.

During ongoing phylogenetic research Breure and Romero (2012) showed that species attributed to Thaumastus belonged to different monophyletic clades. Consequently they have been classified accordingly. The Brazilian species Thaumastus (Thaumastus) achilles (Pfeiffer, 1853) and Thaumastus (Thaumastus) largillierti (Philippi, 1845) grouped with *Megaspira* species and are thus without much doubt placed in the Megaspiridae. As material of the type species Thaumastus (Thaumastus) hartwegi (Pfeiffer in Philippi, 1846)—occurring in Ecuador—has not been sequenced, the classification of the Andean species of this group remains tentative. Also for Thaumastus (Thaumastiella) no material could be analysed as yet, and its classification with this family is only provisional. The genus Paeniscutalus is also provisionally arranged under this family, awaiting a further clarification of the findings of Breure and Romero (2012). They found that the sole species classified with this genus, which they suggested to be a relict of an older group, appeared at the very basis of the phylogenetic tree of the Orthalicoidea.

### Genus Paeniscutalus

Taxon classificationAnimaliaStylommatophoraMegaspiridae

Wurtz, 1947

Bulimulus (Paeniscutalus) Wurtz 1947: 12.

#### Type species.

Megalobulimus (Microborus) incarum Pilsbry, 1944, by monotypy.

#### Diagnosis.

Shell ovate, rimate, rather solid, suture crenulate, surface smooth with more or less incrassate growth striae, aperture (sub-)ovate, peristome slightly thickened and hardly expanded.

#### Distribution.

Peru.

#### Habitat.

Living under stones and buried in the ground at elevations of 1850–3300 m.

#### Anatomy.

Wurtz 1947: Paeniscutalus crenellus (Philippi, 1867) [g, m, p, r]; Breure 1978: *Paeniscutalus crenellus* [g, h, r].

#### Phylogenetic data.

Breure and Romero 2012: Paeniscutalus crenellus (Philippi, 1867).

### Paeniscutalus crenellus

Taxon classificationAnimaliaStylommatophoraMegaspiridae

(Philippi, 1867)

Figs 17A–F , 18

Bulimus crenellus Philippi 1867: 67.
Megalobulimus (Microborus) incarum Pilsbry 1944c: 29, pl. 1 figs 8–9.
Strophocheilus (Microborus) tenuis Haas 1955b: 330, fig. 70.
Thaumastus crenellus ; Richardson 1995: 374 (references, synonymy).
Thaumastus (Paeniscutalus) incarum ; Schileyko 1999: 281, fig. 338.
Thaumastus (Paeniscutalus) crenellus ; Ramírez et al. 2003: 282; Breure and Mogollón 2010: 16.

#### Type locality.

“Peru, hacienda de Unigambal”.

#### Type material.

Not located.

#### Additional material.

ANSP 180677, holotype, and ANSP 411182 (1), paratype of Megalobulimus (Microborus) incarum Pilsbry; FMNH 51925, holotype of Strophocheilus (Microborus) tenuis Haas.

#### Diagnosis.

See above.

#### Dimensions.

Shell height 35, diameter 21 mm (*incarum* Pilsbry), respectively 30.1 and 18.8 mm (*tenuis* Haas).

#### Distribution.

**Peru**, Dept. Ancash, Shaurama near Huaraz (Pilsbry 1944); ibid., Yungay (Haas 1955); ibid., Carhuáz; ibid., hacienda Llaguén, Potrero Nuevo; ibid., between Huaráz and Caráz; ibid., near Colcabamba; ibid., hacienda Damián, Paja; ibid., Pacap; ibid., Tapacocha; ibid, near Cajacay; ibid., N of Chiquián, Aquia (all Breure 1978); ibid., 3 km S Macará (Breure, unpublished data); Dept. La Libertad, Unigambal (Philippi 1867); Dept. Lima, Autisha; ibid., near Yánac; ibid., near Matucana; ibid., near San Bartolomé (all Breure 1978); ibid., Magdalena (Breure and Mogollón 2010).

#### Ecoregion.

Sechura desert [NT1315].

#### Remarks.

This species has been considered as a member of the Strophocheilidae due to its general shell shape, with a relatively low spire (Pilsbry 1944c, Haas 1955b). The anatomy and phylogenetic data, however, clearly shows it belongs to the superfamily Orthalicoidea. The mentioning by Schileyko (1999: 282) of “2 spp.” within this genus is erroneous as all described taxa are synonymous.

### Genus Thaumastus

Taxon classificationAnimaliaStylommatophoraMegaspiridae

Martens in Albers, 1860

Bulimulus (Thaumastus) Martens in Albers 1860: 215.

#### Type species.

*Bulimus hartwegi* Pfeiffer in Philippi, 1846, by original designation.

#### Diagnosis.

Shell elongate-ovate to ovate conical, imperforate to rimate, solid, with rather blunt apex. Colour whitish to (mostly) brownish, generally with axial streaks or spiral band(s). Protoconch with axial sculpture. Whorls hardly to slightly convex, aperture generally subovate, peristome simple or hardly expanded.

#### Distribution.

Ecuador, Peru, Bolivia, Brazil.

#### Habitat.

Occurring generally in evergreen forest up to ca. 3000 m, where the species live in the leaf litter layer.

#### Anatomy.

Pilsbry 1902 [1901–1902]: Thaumastus (Thaumastus) taunaisii (Férussac, 1822) [g, m, r]; Zilch 1953: Thaumastus (Thaumastiella) koepckei (Zilch, 1953) [g, m, r]; Breure 1978: Thaumastus (Thaumastus) foveolatus (Reeve, 1849) [g, h, r], Thaumastus (Thaumastus) insolitus (Preston, 1909) [g, r], Thaumastus (Thaumastus) sangoae (Tschudi, 1852) [g].

#### Phylogenetic data.

Breure and Romero 2012: Thaumastus (Thaumastus) achilles (Pfeiffer, 1853), Thaumastus (Thaumastus) largillierti (Philippi, 1845).

#### Key to the subgenera of Thaumastus in the study area

**Table:** 

1	Protoconch with fine, close axial wrinkles; shell height generally above 48 mm	**Thaumastus (Thaumastus)**
–	Protoconch with axial riblets, which become wavy, anastomosing and irregularly broken up into bead-like to oblong granules on the second whorl; shell height up to 48 mm	**Thaumastus (Thaumastiella)**

### Subgenus Thaumastus (Thaumastiella)

Taxon classificationAnimaliaStylommatophoraMegaspiridae

Weyrauch, 1956

Thaumastus (Thaumastiella) Weyrauch 1956: 11.

#### Type species.

*Bulimulus sarcochrous* Pilsbry, 1897, by original designation.

#### Diagnosis.

Shell ovate conical, narrowly perforate, height up to ca. 30–ca. 47 mm. Colour whitish to brownish, uniformly coloured or with a light coloured spiral band at the periphery. Surface with incrassate growth striae or, additionally, with incised spiral lines or malleation. Protoconch with axial riblets, which become wavy, anastomosing and irregularly broken up into bead-like to oblong granules on the second whorl. Whorls hardly convex, suture crenulate, hardly to well impressed. Aperture (elongate-)ovate. Peristome thickened, simple or hardly expanded below.

#### Distribution.

Peru.

#### Habitat.

Species have been found under stones in ‘savannah forest’ at 1200–2750 m.

### Thaumastus (Thaumastiella) glyptocephalus

Taxon classificationAnimaliaStylommatophoraMegaspiridae

(Pilsbry, 1897)

Figs 19D–G , 20

Bulimulus glyptocephalus Pilsbry 1897: 21; Pilsbry 1897 [1897–1898]: 93, pl. 5 figs 62–64.
Thaumastus glyptocephalus ; Richardson 1995: 376 (references).
Thaumastus (Thaumastiellus) glyptocephalus ; Ramírez et al. 2003: 282.

#### Type locality.

“Peru”.

#### Type material.

ANSP 25675 (1), syntype.

#### Diagnosis.

Shell relatively small, whitish, surface coarsely wrinkle-striate and conspicuously malleated on the last whorl, apex very obtuse, peristome simple, slightly sinuous in side view.

#### Dimensions.

Shell height 31, diameter 17 mm.

#### Distribution.

**Peru**, Dept. Arequipa, SW Arequipa.

#### Ecoregion.

Sechura desert [NT1315].

#### Remarks.

Additional material (ANSP 321960, not seen) suggests the region of occurrence in Dept. Arequipa; the material was collected by W.F. Jenko at 6 km SSW Tiabaya, near Arequipa. This species was considered by Pilsbry as belonging to his group *Protoglyptus* on account of the axial riblets in the protoconch sculpture; however, this genus is currently understood as distributed in the West Indies and eastern South America. Pilsbry considered this taxon closely related to *Bulimulus sarcochrous* Pilsbry, 1897. Weywauch (1956b) placed *Bulimulus glyptocephalus* and *Bulimulus sarcochrous* Pilsbry, 1897 in his Thaumastus (Thaumastiella). It should be noted that the habitat at the above mentioned locality is different (i.e., not forested) from the other species.

### Thaumastus (Thaumastiella) koepckei

Taxon classificationAnimaliaStylommatophoraMegaspiridae

Zilch, 1953

Figs 19H , 20

Thaumastus (Scholvienia) koepckei Zilch 1953: 53, pl. 14 fig. 3; Neubert and Janssen 2004: 214, pl. 2 fig. 25.
Thaumastus koepckei ; Richardson 1995: 378 (references).
Thaumastus (Thaumastiella) koepckei ; Ramírez et al. 2003: 282.

#### Type locality.

“Peru, Hacienda Monteseco (ca. 6°50'S 79°10'W)”.

#### Type material.

SMF 111487, holotype.

#### Additional material.

SMF 111468 (3), 111488 (24), paratypes.

#### Diagnosis.

Shell relatively large, reddish-brown with a yellow peripheral band, surface with a fine spiral sculpture, which is dissolved in the finest elongated marked tubercles, as the basis for fine flat small bristles, peristome thickened.

#### Dimensions.

Shell height 46.6, diameter 21.4 mm.

#### Distribution.

**Peru**, Dept. Cajamarca, Hacienda Monteseco (ca. 6°50'S 79°10'W).

#### Ecoregion.

Tumbes-Piura dry forests [NT0232].

#### Remarks.

The above diagnosis is based on the original description for this species, for which we know no other material than the types.

### Thaumastus (Thaumastiella) occidentalis occidentalis

Taxon classificationAnimaliaStylommatophoraMegaspiridae

Weyrauch, 1960

Figs 19I , 20

Thaumastus (Thaumastiella) occidentalis Weyrauch 1960: 28, pl. 3 figs 13-14; Ramírez et al. 2003: 282; Neubert and Janssen 2004: 220, pl. 2 fig. 24; Köhler 2007: 129, fig. 16; Barbosa et al. 2008: 273; Breure 2012a: 10.
Thaumastus occidentalis ; Richardson 1995: 380 (references, synonymy).

#### Type locality.

“N-Peru am Westhang der westlichen Anden: in der Umgebung von Contumazá, 110 km nö Trujillo”.

#### Type material.

SMF 162026, holotype.

#### Additional material.

ANSP 204515 (2), FMNH 53991, FMNH 216808, MCZ 211967, SMF 162027 (1), SMF 162028 (4), SMF 208392 (4), ZMB 101463 (1), paratypes.

#### Diagnosis.

Shell relatively large, brown, with sculpture of incised spiral lines crossing the growth striae (Weyrauch 1960: fig. 13a), aperture elongate-ovate, peristome hardly expanded at basal margin.

#### Dimensions.

Shell height 45.7, diameter 20.9 mm.

#### Distribution.

**Peru**, Dept. Cajamarca, near Contumazá.

#### Ecoregion.

Tumbes-Piura dry forests [NT0232].

#### Remarks.

The above diagnosis is based on the original description for this species, for which we know no other material than the types.

### Thaumastus (Thaumastiella) occidentalis debilisculptus

Taxon classificationAnimaliaStylommatophoraMegaspiridae

Weyrauch, 1960

Figs 19J , 20

Thaumastus (Thaumastiella) occidentalis debilisculptus Weyrauch 1960: 30, pl. 3 fig. 15; Ramírez et al. 2003: 282; Neubert and Janssen 2004: 207, pl. 2 fig. 26; Barbosa et al. 2008: 270; Breure 2012a: 7.

#### Type locality.

“N-Peru, am Westhang der westlichen Anden: bei Llama (2000–2250 m), an der Autostrasse von Chiclayo nach Cutervo, ca. 80 km nö Chiclayo”.

#### Type material.

SMF 162029, holotype.

#### Additional material.

FML 1630, FMNH 107841, FMNH 216807, FMNH 216880, MCZ 233545, SMF 162082 (8), paratypes.

#### Diagnosis.

Shell relatively medium-sized, brown, with weak sculpture of incised spiral lines crossing the growth striae (Weyrauch 1960: fig. 15a), aperture elongate-ovate, peristome hardly expanded at basal margin.

#### Dimensions.

Shell height 40.0, diameter 17.2 mm.

#### Distribution.

**Peru**, Dept. Cajamarca, near Llama.

#### Ecoregion.

Tumbes-Piura dry forests [NT0232].

#### Remarks.

The above diagnosis is based on the original description for this species, for which we know no other material than the types.

### Thaumastus (Thaumastiella) sarcochrous

Taxon classificationAnimaliaStylommatophoraMegaspiridae

(Pilsbry, 1897)

Figs 19A–C , 20

Bulimulus sarcochrous Pilsbry 1897: 21; Pilsbry 1897 [1897–1898]: 93, pl. 5 figs 65–66.
Thaumastus sarcochrous ; Richardson 1995: 381 (references).
Thaumastus (Thaumastiella) sarcochrous ; Ramírez et al. 2003: 282.

#### Type locality.

“Peru”.

#### Type material.

ANSP 4705, holotype.

#### Diagnosis.

Shell relatively small, whitish to pinkish-brownish, surface weakly striate, faintly malleated on the last whorl, peristome simple.

#### Dimensions.

Shell height 29, diameter 16 mm.

#### Distribution.

**Peru**, Dept. La Libertad, Rio Chusgon valley; ibid., Hacienda Marcabal (USNM 601792).

#### Ecoregion.

Marañon dry forests [NT0223].

#### Remarks.

Weyrauch (1956: 10) provided the first, more precise locality after the original description, viz. Rio Chusgon valley, ca. 50 km NE Huamachuco, at 1600–2150 m.

### Subgenus Thaumastus (Thaumastus)

Taxon classificationAnimaliaStylommatophoraMegaspiridae

Albers, 1860

#### Diagnosis.

Shell elongate-ovate, imperforate, solid, height up to ca. 49–100 mm (study area). Colour light to dark brown, mostly with darker axial streaks or light coloured spiral band(s). Surface with incrassate growth striae. Protoconch with fine, close axial wrinkles. Whorls hardly to slightly convex, suture well impressed, more or less crenulate. Aperture relatively small, subovate. Peristome slightly expanded.

#### Distribution.

Brazil, Bolivia, Peru, Ecuador.

#### Habitat.

As far as ecological data are available, the species live in cloud and montane forest, mainly near rocky outcrop. The altitudinal distribution is 0–2300 m, but likely the species are mainly restricted to the upper half of this range in the area treated.

### Thaumastus (Thaumastus) blanfordianus

Taxon classificationAnimaliaStylommatophoraMegaspiridae

(Ancey, 1903)

Figs 21A–B , 35

Bulimulus blanfordianus Ancey 1903: 90; Wood and Gallichan 2008: 29; Breure 2011: 16, figs 4C–D.
Thaumastus blanfordianus ; Richardson 1995: 373 (references).

#### Type locality.

“Iquico, Bolivia, 3500 m”.

#### Type material.

RBINS/MT1865, lectotype (Breure 2011: 16).

#### Diagnosis.

Shell relatively small, uniformly dark brown coloured on the last whorl, the spire paler, whorls rather convex, suture crenulate, surface sculptured with spirally incised lines, strongest on the penultimate whorl, crossing the incrassate growth lines, columellar margin broadly dilated above.

#### Dimensions.

Shell height 52.5, diameter 25.1 mm.

#### Distribution.

**Bolivia**, Dept. La Paz, Ikiko.

#### Ecoregion.

Bolivian montane dry forests [NT0206].

#### Remarks.

This species is known from the type material only.

### Thaumastus (Thaumastus) buckleyi

Taxon classificationAnimaliaStylommatophoraMegaspiridae

(Higgins, 1872)

Figs 28C–D , 33

Orthalicus (Porphyrobaphe) buckleyi Higgins 1872: 685, pl. 56 fig. 3; Breure and Ablett 2015: 25, figs 3iv–v, L3iii.
Thaumastus buckleyi ; Richardson 1995: 374 (references);
Thaumastus (Thaumastus) buckleyi ; Breure and Borrero 2008: 8.

#### Type locality.

[Ecuador, Prov. Loja] “San Lucas”.

#### Type material.

NHMUK 1872.5.22.6, two syntypes.

#### Diagnosis.

Shell relatively large, slender and elongate, apex obtuse, colour tawny-yellow, whorls slightly convex, suture well impressed, sculptured with incrassate growth lines and malleation, especially on the last whorl, peristome expanded and narrowly reflexed.

#### Dimensions.

Shell height 93, diam. 36 mm.

#### Distribution.

**Ecuador**, Loja, San Lucas (NHMUK, USNM 317381).

#### Ecoregion.

Northwestern Andean montane forests [NT0145].

#### Remarks.

This species is only known from the type locality and is possibly a short-range endemic. The material referred to by Strebel (1909: 138) must be considered lost.

### Thaumastus (Thaumastus) flori

Taxon classificationAnimaliaStylommatophoraMegaspiridae

(Jousseaume, 1897)

Figs 22D–F , 33

Dryptus flori Jousseaume 1897: 265.
Thaumastus flori ; Richardson 1995: 375 (references).
Thaumastus (Thaumastus) flori ; Breure and Mogollón 2010: 17, figs 15-20.

#### Type locality.

[Ecuador] “Machala Équateur”.

#### Type material.

MNHN 22474, lectotype (Breure 1975: 1139).

#### Additional material.

MNHN 22475 (2), paralectotypes.

#### Diagnosis.

Shell relatively large, coloured with axial streaks of yellow to dark chestnut, sculptured with growth striae, thickened at irregular distances, aperture truncate-ovate, columellar margin twisted, peristome slightly expanded below.

#### Dimensions.

Shell height 85.3, diameter 42.8 mm.

#### Distribution.

**Ecuador**, Prov. El Oro, Machala; Prov. Pichincha, Nanegal (Weyrauch 1967: 467).

#### Ecoregion.

Northwestern Andean montane forests [NT0145].

#### Remarks.

This is a quite variable species which Breure and Mogollón (2010) considered identical with Plekocheilus (Eurytus) conspicuus Pilsbry, 1932. They also suggested Thaumastus (Thaumastus) flori (Jousseaume, 1897) to be closely related to Thaumastus (Thaumastus) hartwegi (Pfeiffer, 1846), which occurs in the same general area. Upon comparison of the type specimens, however, we are now of the opinion that Pilsbry’s taxon is a junior subjective synonym of Thaumastus (Thaumastus) hartwegi, and Jousseaume’s taxon is a related but distinct species. The record from Nanegal needs further confirmation.

### Thaumastus (Thaumastus) foveolatus

Taxon classificationAnimaliaStylommatophoraMegaspiridae

(Reeve, 1849)

Figs 25A–C , 30A–B , 34

Bulimus mahogani Pfeiffer 1841: 42; Pfeiffer 1844 in Küster and Pfeiffer 1840–1865: 40, pl. 13 figs 1–2; Pfeiffer 1848: 24. Not Bulinus mahogani Sowerby, 1838. See remarks.
Bulimus foveolatus Reeve 1849 [1848–1850]: pl. 73 fig. 526; Breure and Ablett 2015: 30, figs 1v–vi, L7i.
Bulimus impressus Tschudi in Troschel 1852: 188.
Thaumastus foveolatus ; Richardson 1995: 375 (references, synonymy).
Thaumastus (Thaumastus) foveolatus ; Ramírez et al. 2003: 282.
Thaumastus (Thaumastus) impressus ; Ramírez et al. 2003: 282.

#### Type locality.

“Vitoe, near Sarma [sic, Tarma], Alto-Peru”.

#### Type material.

NHMUK 1975275, lectotype (Breure 1979: 44).

#### Additional material.

NHMUK 1975276 (1), paralectotype.

#### Dimensions.

Shell height 71.5, diameter 37.0 mm.

#### Diagnosis.

Shell relatively medium-sized, uniformly brownish with a slightly darker spiral band at the periphery and a yellowish one below the suture, sculptured with spiral rows of oblong granules, suture crenulate, ascending in front, aperture subovate, columellar margin curved and dilated above, peristome white, hardly expanded below, and very narrowly reflexed.

#### Distribution.

**Peru**, Dept. Junín, near Tarma, Mito; ibid., 19.5 km WNW San Ramón (Breure 1978).

#### Ecoregion.

Peruvian Yungas [NT0153].

#### Remarks.

Pfeiffer (1844 in Küster and Pfeiffer 1840–1865) figured a species clearly unlike the original figure by Sowerby, which he considered a Chilean species; Pfeiffer said his figured specimen was from “Chili und Peru”, only the latter locality seems plausible for this species. Reeve (1849 [1848–1850]) considered his taxon identical to the species figured by Pfeiffer. The name “Vitoe” might be a misspelling for Mito, which is ca. 70 km SE Tarma at ca. 3450 m elevation.

### Thaumastus (Thaumastus) granocinctus

Taxon classificationAnimaliaStylommatophoraMegaspiridae

(Pilsbry, 1901)

Figs 25F , 35

Bulimus (Dryptus) filocinctus Rolle 1901: 93.
Strophocheilus (Thaumastus) granocinctus Pilsbry 1901 [1901–1902]: 126 (new name for Bulimus filocinctus Rolle, 1901 not Reuss, 1861); Neubert and Janssen 2004: 211, pl. 2 fig. 17.
Thaumastus (Thaumastus) granocinctus ; Ramírez et al. 2003: 282.

#### Type locality.

[Peru] “Chanchamayo Peruviae”.

#### Type material.

SMF 208383 (1), syntype.

#### Diagnosis.

Shell relatively large, dark-brown coloured with yellowish subsutural and peripheral bands, sculptured with incrassate growth striae and spiral, incised lines, suture descending in front but slightly ascending behind lip, aperture subovate, peristome hardly expanded.

#### Dimensions.

Shell height 80.5, diameter 42.3 mm.

#### Distribution.

**Peru**, Dept. Junín, Chanchamayo (Rolle 1901); ibid., Perené; Prov. Pasco, Huancabamba.

#### Ecoregion.

Peruvian Yungas [NT0153].

#### Remarks.

This species was described but not figured by Rolle. Neubert and Janssen (2004) found a syntype, smaller than the original dimensions (shell height 94, diameter 50 mm) given by Rolle, in the S.H. Jaeckel collection; their figure is the sole that exists of this taxon. Richardson (1995) put Rolle’s taxon in the synonymy of Thaumastus (Thaumastus) melanocheilus (Nyst, 1845), but comparison of type material shows that this is not warranted. Simroth (1911) reported on an aberrant shell which showed “Riezenwuchs” [growth which leads to abnormal shell height]; in his case the shell was 88 mm high, with locality Chanchamayo, and seems to fit within the variation. It should be noted, however, that Neubert and Janssen (2004: pl. 2 fig. 18) figured a specimen of Bulimus achilles var. nehringi Martens, 1889 from Piracicaba, Edo. Sao Paulo, Brazil, which is very similar to Pilsbry’s taxon, except being stouter. This observation certainly deserves further study.

### Thaumastus (Thaumastus) hartwegi

Taxon classificationAnimaliaStylommatophoraMegaspiridae

(Pfeiffer in Philippi, 1846)

Figs 21E–G , 22A–C , 31C , 33

Bulimus hartwegi Pfeiffer in Philippi 1846 [1845–1847]: 111, pl. 4 fig. 1; Breure and Ablett 2015: 33, figs 3i–iii, L8i.
Zebra loxensis Miller 1879: 119, pl. 12 fig. 2.
Plekocheilus (Eurytus) conspicuus Pilsbry 1932: 390, pl. 27 figs 4 (**syn. n.**); Ramírez et al. 2003: 281.
Thaumastus hartwegi ; Richardson 1995: 376 (references, synonymy).
Thaumastus (Thaumastus) hartwegi ; Ramírez et al. 2003: 282; Breure and Borrero 2008: 9.
Thaumastus (Thaumastus) flori ; Breure and Mogollón 2010: 17, figs 15-20.

#### Type locality.

“respublica [sic] Aequatoris, ubi ad ‘El Catamaija’ prope Loxa”.

#### Type material.

NHMUK 1975126 (1), syntype.

#### Additional type material.

ANSP 141959, holotype, and ANSP 460589, paratypes of Plekocheilus (Eurytus) conspicuus Pilsbry, 1932.

#### Diagnosis.

Shell relatively small to medium-sized, irregularly streaked with white and chestnut-brown, sculptured with incrassate growth striae and spirally incised lines, suture slightly ascending behind lip, aperture truncate-ovate, columellar margin twisted, peristome slightly expanded below.

#### Dimensions.

Shell height 57.0, diameter 30.0 mm (64.5 respectively 33.5 mm, *conspicuus* Pilsbry).

#### Distribution.

**Ecuador**, Prov. Loja, near Catamayo. **Peru**, Dept. Piura, Inia (Breure and Mogollón 2010); near Huasimal (Pilsbry 1932).

#### Ecoregion.

Eastern Cordillera real montane forests [NT0121], Tumbes-Piura dry forests [NT0232].

#### Remarks.

As mentioned above,Plekocheilus (Eurytus) conspicuus Pilsbry is now considered a junior subjective synonym of *Bulimus hartwegi* Pfeiffer (**syn. n.**), after having compared the type specimens.

### Thaumastus (Thaumastus) inca

Taxon classificationAnimaliaStylommatophoraMegaspiridae

(d’Orbigny, 1835)

Figs 26D–F , 30C , 35

Helix inca d’Orbigny 1835: 16; Breure and Ablett 2015: 34, figs 4iv–vi, L8iii.
Thaumastus (Atahualpa) brunneus Strebel 1910: 19, pl. 2 fig. 25.
Thaumastus inca ; Richardson 1995: 376 (references).

#### Type locality.

[Bolivia] “Tutulima, reipublica Boliviana”.

#### Type material.

NHMUK 1854.12.4.116, lectotype (Breure and Ablett 2015: 34).

#### Additional material.

NHMUK 1854.12.4.116 (3), paralectotypes; MNHN 28070 (3), paralectotypes.

#### Diagnosis.

Shell relatively large, elongate, uniformly brownish, suture slightly ascending in front, aperture relatively small, subovate, peristome thickened, sinuous, somewhat expanded, narrowly reflexed.

#### Dimensions.

Shell height 75.4, diameter 32.2 mm.

#### Distribution.

**Bolivia**, Dept. Cochabamba, Totolima.

#### Ecoregion.

Bolivian Yungas [NT0105].

#### Remarks.

This species has only been recorded from the type locality, for which Totolima is now the current name. This is a high-altitude locality (4500 m), which makes it more likely that the species may occur 20-40 km (N)NE where elevations of 2000–2500 m occur; this is the Parque Nacional Isiboro Secure. The synonymization of the Ecuadorian Thaumastus (Atahualpa) brunneus Strebel, 1910 by Richardson (1995) is evidently based upon the opinion of Pilsbry (1932: 391); as Strebel’s material was destroyed during World War 2 there is no longer an opportunity for comparison.

### Thaumastus (Thaumastus) insolitus

Taxon classificationAnimaliaStylommatophoraMegaspiridae

(Preston, 1909)

Figs 25D–E , 35

Bulimus (Thaumastus) insolitus Preston 1909: 509, pl. 10 fig. 9; Breure and Ablett 2015: 35, 4i–iii, L9ii.
Thaumastus insolitus ; Richardson 1995: 377 (references).
Thaumastus (Thaumastus) insolitus ; Ramírez et al. 2003: 282.

#### Type locality.

“Chanchamayo, Peru”.

#### Type material.

NHMUK 1947.3.11.1, holotype.

#### Diagnosis.

Shell relatively medium-sized, blackish-brown coloured, coarsly sculptured with transverse ridges crossed by fine, spiral grooves, giving the last whorls a finely beaded appearance, suture somewhat descending in front, peristome thickened, reflexed below, parietal callus polished.

#### Dimensions.

Shell height 70.4, diameter 31.2 mm.

#### Distribution.

**Peru**, Dept. Junín, Chanchamayo valley; ibid., near Campanillayoc (Zilch 1954: 76); near Carpapata (Breure 1978).

#### Ecoregion.

Peruvian Yungas [NT0153].

### Thaumastus (Thaumastus) integer

Taxon classificationAnimaliaStylommatophoraMegaspiridae

(Pfeiffer, 1855)

Figs 27A–B , 31A–B

Bulimus integer Pfeiffer 1855: 114; Breure and Ablett 2015: 35, figs 5i–iii, L10i.
Pachytholus pseudoiostomus Strebel 1909: 139, pl. 21 fig. 338, pl. 26 figs 397–398.
Thaumastus integer ; Richardson 1995: 377 (references, synonymy).
Thaumastus (Thaumastus) integer ; Breure and Borrero 2008: 8.

#### Type locality.

“Quito, Ecuador”.

#### Type material.

NHMUK 1975244, lectotype (Breure 1978: 31).

#### Additional material.

NHMUK 1975245 (1), paralectotype.

#### Diagnosis.

Shell relatively large, irregularly streaked with white and chestnut-brown, sculptured with incrassate growth striae and spirally incised lines, giving the shell a puckered appearance, aperture truncate-ovate, columellar margin twisted, peristome slightly expanded below.

#### Dimensions.

Shell height 81.5, diameter 42.0 mm.

#### Distribution.

**Ecuador**, without precise locality.

#### Remarks.

The material described by Pfeiffer originated possibly from southern Ecuador. The figured specimen by Strebel (1909) was based on material without locality data. This species is related to Thaumastus (Thaumastus) hartwegi (Pfeiffer in Philippi, 1846), Thaumastus (Thaumastus) flori (Jousseaume, 1897), and Thaumastus (Thaumastus) orcesi Weyrauch, 1967.

### Thaumastus (Thaumastus) loxostomus

Taxon classificationAnimaliaStylommatophoraMegaspiridae

(Pfeiffer, 1855)

Figs 26A–C

Bulimus loxostomus Pfeiffer 1855: 114; Breure and Ablett 2015: 38, figs 5iv–vi, L11iii.
Thaumastus loxostomus ; Richardson 1995: 378 (references).
Thaumastus (Thaumastus) loxostomus ; Breure and Borrero 2008: 8; Linares and Vera 2012: 206.

#### Type locality.

“in Andibus Novae Granadae”.

#### Type material.

NHMUK 1975125, one syntype.

#### Diagnosis.

Shell relatively medium-sized, with creamy ground colour and brownish axial streaks and blotches at irregular distances, suture crenulate, descending in front, ascending at the insertion of the peristome, which is thickened, hardly expanded below and hardly reflexed.

#### Dimensions.

Shell height 71.3, diameter 37.3 mm.

#### Distribution.

**Ecuador**, ?Prov. Loja.

#### Remarks.

This species has not been found since its original publication. Breure and Borrero (2008) assumed this species to be distributed in southern Ecuador, while Linares and Vera (2012) attributed it to the Colombian malacofauna without further evidence.

### Thaumastus (Thaumastus) magnificus

Taxon classificationAnimaliaStylommatophoraMegaspiridae

(Grateloup, 1839)

Figs 27C–E

Bulimus magnificus Grateloup 1839a: 165; Grateloup 1839b: 419, pl. 4 fig. 1; Breure and Ablett 2015: 39, 6i–iii, L12i.
Thaumastus magnificus ; Richardson 1995: 379 (references); Simone 2006: 153, fig. 521.
Thaumastus (Thaumastus) magnificus ; Ramírez et al. 2003: 282.

#### Type locality.

“Pérou”.

#### Type material.

NHMUK 1907.11.22.24, lectotype (Breure 1978: 31).

#### Diagnosis.

Shell relatively large, brownish with a small, somewhat lighter girdle at the periphery, sculptured with incrassate growth striae and spiral striation, most noteable on the upper whorls, suture slightly ascending in front, peristome thin, sinuous, simple.

#### Dimensions.

Shell height 78.0, diameter 36.0 mm.

#### Distribution.

?**Peru** (see remarks). Brazil (Simone 2006).

#### Remarks.

This species has been recorded from eastern Brazil by Simone (2006), and its presence in Peru, for which we have not seen any verified material or record, remains doubtful at best.

### Thaumastus (Thaumastus) melanocheilus

Taxon classificationAnimaliaStylommatophoraMegaspiridae

(Nyst, 1845)

Figs 21C–D , 34

Bulimus melanocheilus Nyst 1845: 149, pl. 2 fig. 3; Breure 2011: 34, figs 4A–B, 4i.
Thaumastus melanocheilus ; Richardson 1995: 379 (references, synonymy).
Thaumastus (Thaumastus) melanocheilus ; Ramírez et al. 2003: 282.

#### Type locality.

“l’Amérique meriodionale, au Pampas”.

#### Type material.

RBINS/MT2361, lectotype (Breure 2011: 34).

#### Diagnosis.

Shell relatively large, brownish with a somewhat lighter girdle at the periphery, sculptured with incrassate growth striae and an indistinct spiral striation, suture plicated below, descending in front, aperture elongate-ovate, peristome thickened, hardly expanded below.

#### Dimensions.

Shell height 78.5, diameter 36.6 mm.

#### Distribution.

**Peru**, Dept. Huánuco, Pampayacu (Breure 2011).

#### Ecoregion.

Peruvian Yungas [NT0153], Southwest Amazon moist forests [NT0166].

#### Remarks.

We have found specimens that are intermediate between Thaumastus (Thaumastus) melanocheilus and Thaumastus (Thaumastus) sangoae (Tschudi in Troschel, 1852) on one hand, and between Thaumastus (Thaumastus) melanocheilus and Thaumastus (Thaumastus) robertsi Pilsbry, 1932 on the other hand. The variation and distribution records of these three taxa need more study; they might prove synonyms but molecular studies could help to clarify the systematic position of these species.

### Thaumastus (Thaumastus) orcesi

Taxon classificationAnimaliaStylommatophoraMegaspiridae

Weyrauch, 1967

Figs 24C–F , 33

Thaumastus (Thaumastus) orcesi Weyrauch 1967: 473, fig. 2; Breure and Borrero 2008: 9; Breure 2012a: 11, pl. 6 figs 59–61.

#### Type locality.

“Ecuador, cuenca del río Esmeraldas, 35 km al noroeste de Quito, region de Nanegal, 1500 m”.

#### Type material.

FML 3165, holotype.

#### Additional material.

SMF 156325 (1), paratype.

#### Diagnosis.

Shell relatively small, irregularly streaked with white and chestnut-brown, sculptured with incrassate growth striae and spirally incised lines, giving the shell a puckered appearance, aperture truncate-ovate, columellar margin twisted, peristome slightly expanded below.

#### Dimensions.

Shell height 49.4, diameter 23.8 mm.

#### Distribution.

**Ecuador**, Prov. Pichincha, Nanegal.

#### Ecoregion.

Northwestern Andean montane forests [NT0145].

#### Remarks.

This species is evidently related to Thaumastus (Thaumastus) hartwegi (Pfeiffer in Philippi, 1846), Thaumastus (Thaumastus) integer (Pfeiffer, 1855), and Thaumastus (Thaumastus) flori (Jousseaume, 1897).

### Thaumastus (Thaumastus) orobaenus

Taxon classificationAnimaliaStylommatophoraMegaspiridae

(d’Orbigny, 1835)

Figs 29A–C , 35

Helix orobaena d’Orbigny 1835: 17; d’Orbigny 1837 [1834–1847]: 293.
Thaumastus orobaenus ; Richardson 1995: 380 (references).

#### Type locality.

“provincia Yungacensi, republica Boliviana”.

#### Type material.

MNHN 28091, lectotype (Breure 1975b).

#### Diagnosis.

Shell relatively small, rimate, brownish with small yellowish blotches, the apex paler, suture slightly crenulate, ascending in front, sculptured with incrassate growth striae and spirally incised lines, forming oblong granules, aperture relatively small, columellar margin narrowly dilated above, entering the aperture with a small twist, peristome whitish, simple, parietal callus thin, whitish.

#### Dimensions.

Shell height 38.8, diameter 16.8 mm.

#### Distribution.

**Bolivia**, Dept. La Paz, Circuata.

#### Ecoregion.

Bolivian montane dry forests [NT0206].

#### Remarks.

d’Orbigny (1837 [1834–1847]: 293–294) precised the type locality as “au milieu d’un bois très-humide, au sommet de la montagne dite du Biscachal, près du village de Carcuata”.

### Thaumastus (Thaumastus) robertsi robertsi

Taxon classificationAnimaliaStylommatophoraMegaspiridae

Pilsbry, 1932

Figs 23A–D , 34

Thaumastus robertsi Pilsbry 1932: 390, pl. 27 figs 3, 6; Richardson 1995: 387 (references).

#### Type locality.

“Rio Jelashte, at about 4500 ft., Dept. of San Martin, Peru”.

#### Type material.

ANSP 159920, holotype.

#### Diagnosis.

Shell relatively medium-sized, brownish, with lighter subsutural and peripheral bands, sculptured with fine, irregular wrinkles and spaced spiral series of little granules, suture crenulate, aperture with a brown coloured band behind the peristome, which is thickened and slightly expanded.

#### Dimensions.

Shell height 63.7, diameter 31.6 mm.

#### Distribution.

**Peru**, Dept. Amazonas, Chachapoyas (NHMUK 1896.6.23.3–4); Dept. San Martin, Rio Jelashte [E of Leymebamba], ca. 1500 m.

#### Ecoregion.

Peruvian Yungas [NT0153].

#### Remarks.

Upon further collecting and careful studies, preferably in conjunction with molecular research, this species may prove to be closely related to Thaumastus (Thaumastus) melanocheilus (Nyst, 1845).

### Thaumastus (Thaumastus) robertsi satipoensis

Taxon classificationAnimaliaStylommatophoraMegaspiridae

Pilsbry 1944

Figs 23E–G , 34

Thaumastus robertsi satipoensis Pilsbry 1944b: 121, pl. 11 fig. 1.
Thaumastus robertsi ; Richardson 1995: 387 (references, synonymy).
Thaumastus satipoensis ; Ramírez et al. 2003: 282.

#### Type locality.

“Satipo, near Huancayo, Peru, at 600 m”.

#### Type material.

ANSP 179990, holotype.

#### Diagnosis.

Shell as in the nominate taxon, but more slender and the spire forming a higher, narrower cone.

#### Dimensions.

Shell height 74.4, diameter 34.0 mm.

#### Distribution.

**Peru**, Dept. Junín, Satipo (ANSP, USNM 601810).

#### Ecoregion.

Peruvian Yungas [NT0153].

#### Remarks.

See under the nominate taxon.

### Thaumastus (Thaumastus) sangoae

Taxon classificationAnimaliaStylommatophoraMegaspiridae

(Tschudi in Troschel, 1852)

Figs 24A–B , 34

Bulimus sangoae Tschudi in Troschel 1852: 189, pl. 6 fig. 1.
Thaumastus sangoae ; Richardson 1995: 381 (references).
Thaumastus (Thaumastus) sangoae ; Ramírez et al. 2003: 282.

#### Type locality.

“Urwäldern von Sangoa in Peru”.

#### Type material.

Not located.

#### Diagnosis.

Shell relatively large, brownish, with lighter subsutural and peripheral bands, sculptured with fine, irregular growth striae, the last whorl subcancellated and somewhat beaded, aperture subovate, with a brown coloured band behind the lip.

#### Dimensions.

Shell height 81, diameter 40 mm.

#### Distribution.

**Peru**, Dept. Junin, Río Pangoa valley; ibid., 16.8 km WNW San Ramón (Breure 1978).

#### Ecoregion.

Southwest Amazon moist forests [NT0166].

#### Remarks.

As Morelet (1863: 155) has pointed out, the name *sangoae* was probably an error and refers to Río Pangoa in Dept. Junín, “qui prend sa source sur les hauteurs d’Andamarca et qui donne son nom à la vallée qu’elle arrose dans la partie inférieure de son cours”. The colour pattern of Troschel’s figure suggests that this species may be close to Thaumastus (Thaumastus) robertsi Pilsbry, 1932 and Thaumastus (Thaumastus) melanocheilus (Nyst, 1845).

### Thaumastus (Thaumastus) sumaqwayqu sp. n.

Taxon classificationAnimaliaStylommatophoraMegaspiridae

http://zoobank.org/669169B5-A3C3-4803-9891-38980E11E1A9

Figs 32A–F , 35 , 87B

#### Diagnosis.

A relatively small species of Thaumastus (Thaumastus), characterized, when freshly collected, by the deep brown colour on the last whorl, with a golden hue, with two small brown spiral bands on the upper whorls, one of which is subsutural, the lower one becomes peripheral on last whorls, which have on the upper side a zone of axial bands, the interstices twice as broad.

#### Description.

Shell up to 52.5 mm, 2.0 times as long as wide, imperforate, rather thin, elongate-ovate, with hardly convex sides, with (when fresh) a deep brown colour on the last whorl, with a golden hue, with two small brown spiral bands on the upper whorls, one of which is subsutural, the lower one becomes peripheral on last whorls, which have on the upper side a zone of axial bands, the interstices twice as broad. Protoconch sculptured with fine axial wrinkles, partly bifurcating or anostomsing on lower part of whorl, on the second whorl partly broken up in oblong granules; teleoconch sculptured with incrassate growth striae and very shallow, more or less interrupted, spiral depressions. Whorls up to 5, hardly convex, suture slightly impressed, somewhat crenulate. Aperture narrowly elongate-ovate, pale brown with a whitish lustre inside, 1.6 times longer than wide, 0.5 times the total height, peristome thin and simple, columellar margin slightly curved, receding above, threadlike entering the aperture, parietal callus transparent and thin.

#### Dimensions in mm.

H 40.5–52.5, D 21.0–25.2, HA 22.2–25.2, WA 14.2–15.7, LW 32.7–40.8, 4.5–5.0 whorls. Holotype H 52.5, D 25.2, HA 25.2, WA 15.7, LW 40.8, 5.0 whorls.

#### Type locality.

Peru, Dept. Cuzco, 1.6 km W of Aguas Calientes, slope along river, on the ground between plants near rocks, 1985 m (Fig. 87A).

#### Ecoregion.

Peruvian Yungas [NT0153].

#### Type material.

RMNH 201636, holotype. RMNH 201637 (1), VMA (3), paratypes. All material S.J. Breure-Dorsman & A.S.H. Breure leg., 12 March 2012.

#### Additional material.

Peru, Dept. Cuzco, W of Aguas Calientes, FML (5). M.G. Cuezzo & E. Dominguez leg., 7 March 2007.

#### Comparison with other species.

This new species resembles Thaumastus (Thaumastus) inca (d’Orbigny, 1835) but differs in being smaller, having the apex more blunt, the peristome not thickened, nor sinuous.

#### Remarks.

The holotype has lost the outer shell layer on the last whorls during conservation. Also some of the other specimens in the material examined have partially lost this layer.

#### Etymology.

The specific epithet is formed from the Quechua words *sumaq* (good, beautiful) and *wayqu* (ravine), referring to the type locality, which is along the river at the basis of Machu Picchu. The epithet is used as a noun in apposition.

### Thaumastus (Thaumastus) tatutor

Taxon classificationAnimaliaStylommatophoraMegaspiridae

(Jousseaume, 1887)

Figs 29D–E

Tatutor tatutor Jousseaume 1887: 6, fig. 1.
Thaumastus tatutor ; Richardson 1995: 383 (references).

#### Type locality.

“Nouvelle Grenada”.

#### Type material.

MNHN 28122, holotype.

#### Diagnosis.

Shell relatively large, brownish, the upper whorls paler, sculptured with incrassate growth striae, suture crenulate, hardly ascending in front, aperture elongate-subovate, with a brownish colour band behind the lip, peristome somewhat thickened, hardly expanded.

#### Dimensions.

Shell height 99.9, diameter 52.5 mm.

#### Distribution.

?Colombia. ?**Ecuador**. ?Venezuela.

#### Remarks.

This species has not been found since its description. Given the political boundaries of the former ‘Nouvelle Grenada’, it may be expected in Colombia, Ecuador or Venezuela.

### Thaumastus (Thaumastus) taunaisii

Taxon classificationAnimaliaStylommatophoraMegaspiridae

(Férussac, 1822)

Figs 28A–B

Helix (Cochlostyla) taunaisii Férussac 1822 [1821–1822]: 48.
Bulimus achilles Pfeiffer 1853b: 378.
Thaumastus (Thaumastus) taunaisii ; Richardson 1995: 383 (references, synonymy).
Thaumastus (Thaumastus) achilles ; Ramírez et al. 2003: 282; Simone 2006: 152, fig. 514.

#### Type locality.

[Brazil] “in ripis fluvii Amazonum”.

#### Type material.

Not located.

#### Additional material.

NHMUK 1975268, lectotype of *Bulimus achilles* Pfeiffer (Breure 1978: 32); NHMUK 1975269 (2), paralectotypes.

#### Diagnosis.

Shell relatively medium-sized, tawny coloured with some axial streaks of (purplish- to reddish-)brown, a light girdle at the periphery, sculptured with growth striae and fine, somewhat undulating, spiral, incised lines, aperture subovate, peristome somewhat thickened and hardly expanded at basal margin.

#### Dimensions.

Shell height 58.0, diameter 25.5 mm.

#### Distribution.

Brazil (Simone 2006).

#### Remarks.

The record for Peru by Ramírez et al. (2003) of this eastern Brazilian species may be due to a misidentification and needs further confirmation.

### Family Odontostomidae

Taxon classificationAnimaliaStylommatophoraOdontostomidae

Pilsbry & Vanatta, 1898

Odontostomidae Pilsbry and Vanatta 1898: 283.

### Genus Cyclodontina

Taxon classificationAnimaliaStylommatophoraOdontostomidae

Beck, 1837

Pupa (Cyclodontina) Beck 1837: 88.

#### Type species.

*Pupa inflata* Wagner, 1827, by subsequent designation (Pilsbry 1901 [1901–1902]: 58).

#### Diagnosis.

Shell elongate-ovate to subfusiform, rimate, thin to rather solid, glossy, height up to ca. 22 mm (study area), groundcolour whitish to tawny, whorls slightly convex, protoconch with delicately radially costulae, later with fine, irregular, radial wrinkles and wavy spiral striae, aperture irregularly ovate, only slightly oblique, with 4–5 teeth, parietal lamella thin, rather short, columellar lamella spirally ascending, baso-palatal wall with 2–3 short plicae, upper sometimes absent, peristome thin, a little reflexed (modified after Schileyko 1999).

#### Distribution.

Bolivia, Paraguay, Argentina, ?Uruguay, Brazil.

#### Habitat.

Insufficient data available.

#### Anatomy.

Breure and Schouten 1985: *Cyclodontina tudiculata* (Martens, 1868) [g].

#### Phylogenetic data.

Breure and Romero 2012: *Cyclodontina guarani* (d’Orbigny, 1835).

### Cyclodontina chuquisacana

Taxon classificationAnimaliaStylommatophoraOdontostomidae

(Marshall, 1930) comb. n.

Figs 36C–E , 38

Odontostomus (Spixia) chuquisacana Marshall 1930: 3, pl. 1 fig. 2; Zischka 1953: 82.
Spixia chuquisacana ; Richardson 1993: 57 (references).

#### Type locality.

“Province of Chuquizaca, Bolivia”.

#### Type material.

USNM 380700, holotype.

#### Diagnosis.

Shell thin, rimate, chestnut to grayish-tawny coloured, sculptured with numerous low, irregular, wavy, sometimes interrupted, longitudinal folds, and a faint indication of spiral striae, last whorl contracted at base, angulate around umbilicus, a deep pit just behind the outer lip, aperture subtriangular, with a prominent palatal lamella, a weak callus as basal lamellae, a strong, platelike, twisted columellar lamella, peristome thin, rounded below attachment to body whorl (modified after Marshall 1930).

#### Dimensions.

Shell height 17.5, diameter 4.75 mm.

#### Distribution.

**Bolivia**, Dept. Chuquisaca.

#### Remarks.

In his description Marshall mentioned the protoconch sculpture as “apical and first three whorls are confusedly vertically costulate, malleate and spirally striate”. The latter description hints to a protoconch sculpture which is classified by Schileyko (1999) as *Cyclodontina* Beck, 1837. Also other characteristics place this species in the vicinity of *Cyclodontina lemoinei* (Ancey, 1892). This taxon needs further anatomical and molecular studies to clarify its systematic position.

### Cyclodontina lemoinei

Taxon classificationAnimaliaStylommatophoraOdontostomidae

(Ancey, 1892)

Figs 36A–B , 38 , 84D–F

Odontostomus lemoinei Ancey 1892a: 178; Ancey 1892b: 93, fig. 1; Richardson 1993: 47 (references, synonymy); Wood and Gallichan 2008: 58, pl. 10 fig. 4, iv.

#### Type locality.

“Santa Cruz de la Sierra, Bolivia”.

#### Type material.

NMW 1955.158.24077 (1), possible syntype.

#### Diagnosis.

Shell tawny with whitish, oblique riblets, on lower whorls vermiculate or wrinkled, last whorl tapering, angular around the umbilicus, a deep pit just behind the outer lip, aperture oblique, oblong, with four teeth (moderate parietal lamella, prominent columellar lamella, indistinct basal lamella, large palatal lamella), peristome angular above and at base, expanded (modified after Ancey 1892).

#### Dimensions.

Shell height 22, diameter 6.25 mm.

#### Distribution.

**Bolivia**, Dept. Santa Cruz.

#### Ecoregion.

Chiquitano dry forests [NT0212].

#### Remarks.

There is material in the UF collection (not seen), which is supposedly this species according to their datebase; this material was collected in Dept. Santa Cruz, Prov. Nuflo de Chavez, 32 km W Santa Rosa de la Roca at 545 m elevation (UF 212848). This is the only precise record known to us for this taxon.

### Genus Spixia

Taxon classificationAnimaliaStylommatophoraOdontostomidae

Pilsbry & Vanatta, 1898

Odontostomus (Spixia) Pilsbry and Vanatta in Pilsbry 1898: 57.

#### Type species.

*Pupa striata* Wagner, 1827, by original designation.

#### Diagnosis.

Shell high-conic to subcylindrical, rimate, moderately solid, height up to ca. 35 mm (study area), groundcolour whitish to corneous, sometimes with reddish streaks, whorls slightly convex, protoconch finely regularly striated, then striae becoming obsolete, teleoconch sometimes with radially riblets, aperture irregularly ovate, with four teeth (parietal lamella short, columellar lamella very oblique, long, entering, basal lamella tubercular, palatal lamella short, triangular), peristome angular above and at base, expanded (modified after Schileyko 1999).

#### Distribution.

Bolivia, Paraguay, Argentina, Uruguay, Brazil.

#### Habitat.

Found under rocks and among roots and basal portions of small shrubs.

#### Anatomy.

Breure and Schouten 1985: *Spixia aconjigastana* (Döring, 1876) [g, r], *Spixia doellojuradoi* (Parodiz, 1941) [g, h, m, r], *Spixia pyrgula* (Hylton Scott, 1952) [g, r]; *Spixia striata* (Spix in Wagner,1827) [g, r]; Schileyko 1999: Spixia striata (Wagner, 1827) [g, m]; Salas Oroño 2007: *Spixia doellojuradoi* (Parodiz, 1941) [g, m, r, p], *Spixia martensii* (Döring, 1874) [g, m, r], *Spixia pyriformis* (Pilsbry, 1901) [g, m], *Spixia tucumanensis* (Parodiz, 1941) [g, m]; Salas Oroño 2010: *Spixia cuezzae* Salas Oroño, 2010 [g, m, r, p].

#### Phylogenetic data.

Breure et al. 2010: *Spixia popana* (Döring, 1874); Breure and Romero 2012: *Spixia pervarians* Haas, 1936, *Spixia philippii* (Döring, 1874), *Spixia tucumanensis* (Parodiz, 1941).

### Spixia minor

Taxon classificationAnimaliaStylommatophoraOdontostomidae

(d’Orbigny, 1837)

Figs 37A–E , 38

Helix spixii var. minor d’Orbigny 1835: 21. Nomen nudum.
Pupa spixii var. β minor d’Orbigny 1837 [1834–1847]: pl. 41*bis* fig. 11; d’Orbigny 1838 [1834–1847]: 320; Breure and Ablett 2012: 26, figs 21A–F, 21i.
Spixia minor ; Cuezzo et al. 2013: 178 (references, synonymy).

#### Type locality.

[Bolivia] “province de Chiquitos, entre Santo-Corazon et San-Juan”; see Breure 1973: 123.

#### Type material.

NHMUK 1854.12.4.231, lectotype (Breure and Ablett 2012), and NHMUK 1854.12.4.231 (7), paralectotypes.

#### Diagnosis.

Shell slender and elongate, rather thin, broadly perforate, grayish-tawny coloured, sculptured with growth striae and a faint indication of spiral lines, suture abruptly ascending behind the lip, aperture oblique-ovate, with five teeth (small suprapalatal lamella, large palatal lamella, small basal lamella, prominent columellar lamella entering the aperture, large but relatively thin parietal lamella), peristome thickened, expanded.

#### Dimensions.

Shell height 29.2, diameter 7.46 mm.

#### Distribution.

**Bolivia**, Dept. Santa Cruz, between San Juan de Chiquitos and Ruinas de Santo Corazón.

#### Ecoregion.

Dry Chaco [NT0210].

#### Remarks.

Breure and Ablett (2012) clarified the confusion about d’Orbigny’s varietal names for *Pupa spixii* by selecting lectotypes for each variety and giving *minor* specific status; the expert opinion of Cuezzo et al. (2013) is here adopted for the current systematic position.

### Spixia striata

Taxon classificationAnimaliaStylommatophoraOdontostomidae

(Wagner, 1827)

Figs 37F–I , 38

Pupa striata Wagner 1827: 19.
Helix spixii var. major d’Orbigny 1835: 21 [nomen nudum].
Pupa spixii var. α major d’Orbigny 1838 [1834–1847]: 320; Breure and Ablett 2012: 25, figs 22A–E, 22i.
Spixia striata ; Cuezzo et al. 2013: 182 (references, synonymy).

#### Type locality.

[Brazil] “in Provinciis S. Pauli et Sebastianopolitana”.

#### Type material.

Not located.

#### Additional material.

NHMUK 1854.12.4.232, lectotype (Breure and Ablett 2012), and NHMUK 1854.12.4.232 (6), paralectotypes of *Pupa spixii major* d’Orbigny.

#### Diagnosis.

Shell elongate-ovate, rather solid, broadly perforate, whitish with tawny blotches, sculptured with incrassate growth striae, suture slightly ascending behind the lip, aperture squarish oblique-ovate, with four teeth (small palatal lamella, indistinct basal lamella, concave columellar lamella entering the aperture, rectangular parietal lamella), peristome thickened, well expanded, narrowly reflexed.

#### Dimensions.

Shell height 34.8, diameter 11.0 mm.

#### Distribution.

**Bolivia**, Dept. Santa Cruz, Prov. Chiquitos (d’Orbigny 1838 [1834–1847]). Paraguay. Argentina (Cuezzo et al. 2013). Brazil (Simone 2006).

#### Ecoregion.

Chiquitano dry forests [NT0212].

#### Remarks.

Breure (2013: 14) discussed the need for an in-depth study of the variation of this wide-ranging species; preferably with anatomical and molecular research. The Bolivian record based on d’Orbigny (1838) “frontières nord de la province de Chiquitos” needs further confirmation. The Bolivian material which is found as Spixia striata in museum collections may need re-identification in the light of the recent split of the two varieties of d’Orbigny.

### Family Orthalicidae

Taxon classificationAnimaliaStylommatophoraOrthalicidae

Martens in Albers, 1860

Orthalicidae Martens in Albers 1860: 209.

### Genus Clathrorthalicus

Taxon classificationAnimaliaStylommatophoraOrthalicidae

Strebel, 1909

Orthalicus (Clathrorthalicus) Strebel 1909: 150.

#### Type species.

*Orthalicus wallisi* Strebel, 1909, by original designation (Strebel 1909: 102).

#### Diagnosis.

Shell ovate-conic, thin, whorls slightly convex, apex rather blunt, height up to ca. 30–45 mm (study area), colour of early whorls uniformly pink, yellowish or greyish-brown, the last whorls with dark radial streaks interrupted by 2–3 light bands, typically on the penultimate whorl with a subsutural band on a lighter ground colour, protoconch pitted, teleoconch with growth striae and delicate spiral lines, aperture ovate, peristome expanded, parietal wall brown (modified after Schileyko 1999).

#### Distribution.

Colombia, Ecuador.

#### Habitat.

Probably living in trees, as far as known in cloud forests (Figs 85E–F, 86D–F).

#### Remarks.

This genus is scarcely represented in (historical) collections, and only recently some of its taxa were transferred to it and Strebel’s taxon given generic status (Breure and Ablett 2015). Further morphological and molecular studies should clarify its systematic position.

#### Key to species in the study area

**Table:** 

1	Spiral band above the periphery on last whorl absent	**2**
–	Spiral band above the periphery on last whorl present	***magnificus***
2	Shell height up to ca. 32 mm	***phoebus***
–	Shell height larger than 35 mm	***corydon***

### Clathrorthalicus corydon

Taxon classificationAnimaliaStylommatophoraOrthalicidae

(Crosse, 1869) comb. n.

Figs 39D–G

Bulimus corydon Crosse 1869: 185; Crosse 1870: 104, pl. 6 fig. 6.
Plekocheilus corydon ; Richardson 1995: 308 (references).
Plekocheilus (Eurytus) corydon ; Breure and Borrero 2008: 5; Borrero and Breure 2011: 55.

#### Type locality.

“Quito”.

#### Type material.

MNCN 15.05/8077 (1), MNCN 15.05/13683 (1), MNCN 15.05/21868 (1), syntypes.

#### Diagnosis.

Shell (elongate-)ovate, creamy ground colour with a nubelous pattern of streaks and spots of russet-brown, indistinctly sculptured with growth striae, suture hardly ascending behind the lip, aperture with well expanded and reflexed peristome.

#### Dimensions.

Shell height 32, diameter 23.5 mm.

#### Distribution.

**Ecuador**, Mindo (Borrero and Breure 2011).

#### Ecoregion.

Northwestern Andean montane forests [NT0145].

#### Remarks.

Crosse did not state on how many specimens his description was based. None of the syntypes found in MNCN correspond exactly with the measurements given by Crosse. However, since no specimens have been located in the MNHN collection, it is assumed that all material was returned by Crosse and is now preserved in Madrid. Breure and Ablett (2015: 39, 45) suggested that this taxon belongs to *Clatrorthalicus*, and inspection of the MNCN material corroborates this point of view. It may be noted that this species strongly resembles *Clathrorthalicus phoebus* (Pfeiffer, 1863), and further studies of the variation and distribution of both species are needed to fully assess their taxonomic positions as a synonymy might be involved.

### Clathrorthalicus magnificus

Taxon classificationAnimaliaStylommatophoraOrthalicidae

(Pfeiffer, 1848)

Figs 40A–B

Achatina magnifica Pfeiffer 1848a: 232; Breure and Ablett 2015: 38, figs 7i–ii, L11iv.
Hemibulimus magnificus ; Richardson 1993: 71 (references).
Hemibulimus (Hemibulimus) magnificus ; Breure and Borrero 2008: 29.

#### Type locality.

“Quito, Ecuador”.

#### Type material.

NHMUK 20100508, two syntypes.

#### Diagnosis.

Shell very thin, ground colour creamy-pink with somewhat undulating, axial streaks of brown and on the last whorl two spiral bands with arrow-like (<<) blotches, aperture elongate-ovate, with truncate-sprouted base, very thin and simple peristome.

#### Dimensions.

Shell height 46.6, diameter 23.0 mm.

#### Distribution.

**Ecuador**, without precise locality.

#### Remarks.

This species is only known by the type material, which may prove to be subadult as the aperture is not rounded and the peristome not expanded like in the other two species.

### Clathrorthalicus phoebus

Taxon classificationAnimaliaStylommatophoraOrthalicidae

(Pfeiffer, 1863)

Figs 39A–C

Bulimus phoebus Pfeiffer 1863: 274; Breure and Ablett 2015: 44, figs 7iii–v, L15iv.
Plekocheilus phoebus ; Richardson 1995: 318 (references).
Plekocheilus (Eurytus) phoebus ; Breure and Borrero 2008: 6.

#### Type locality.

“Ecuador”.

#### Type material.

NHMUK 1975143, lectotype (Breure 1979: 30).

#### Diagnosis.

Shell ovate, creamy ground colour with few axial streaks and spots of russet-brown, on the last whorls a lighter subsutural band is visible, indistinctly sculptured with growth striae, suture hardly ascending behind the lip, aperture with well expanded and reflexed peristome.

#### Dimensions.

Shell height 30.5, diameter 17.5 mm.

#### Distribution.

**Ecuador**, without precise locality.

#### Remarks.

This species strongly resembles Clathrorthalicus corydon (Crosse, 1869), being only slightly smaller. Upon further studies both taxa may prove to be synonyms.

### Genus Corona

Taxon classificationAnimaliaStylommatophoraOrthalicidae

Albers, 1850

Achatina (Corona) Albers 1850: 193.

#### Type species.

Helix (Cochlitoma) regina Férussac, 1821, by subsequent designation (Martens in Albers, 1860).

#### Diagnosis.

Shell dextral or sinistral (eniantomorphy), elongate-ovate, solid, shining, height up to ca. 80 mm (study area), corneous or pinkish ground colour, uniformly or (usually) with a dark or light peripheral band (mostly with arrow shaped markings) and axial streaks of reddish-brown, sculptured with growth striae, aperture (narrowly) subovate, peristome simple, parietal and columellar walls dark-brown to blackish.

#### Distribution.

Colombia, Ecuador, Peru, Bolivia, Brazil, French Guiana, Suriname, Guyana, ?Venezuela.

#### Habitat.

Species of this genus live supposedly most of the time at canopy level in lowland tropical rainforest (W.J.M. Maassen, unpublished data); at occasions they descent downwards and may be found on tree stems or near the ground.

#### Anatomy.

Schileyko 1999: *Corona perversa* (Swainson, 1821) [g, m, as *Laeiorthalicus reginaeformis* (Strebel, 1909)]; Breure and Mogollón 2010: Corona pfeifferi (Hidalgo, 1869) [g].

#### Phylogenetic data.

Breure et al. 2010: Corona pfeifferi (Hidalgo, 1869).

#### Remarks.

Species of this genus show quite some variation and their distinction is, with some exceptions, difficult as they are often found in low numbers (one or a few shells at most) at a specific locality. Moreover, several species show enantiomorphy, which may add to taxonomic confusion. Distributional records for species in this group thus need to be viewed in this context. Due to their hidden habitat at the canopy level their distribution records probably do not reflect their true occurrence.

The taxonomy of this group is hampered by the fact that a) most species described are morphologically very similar; b) the type material of some species has either not been located or is worn, thus making comparative research difficult; c) intraspecific variation is insufficiently known, and anatomical and molecular data is rare; and d) many records in museum collections often have imprecise localities. Moreover, the distribution of these species over the larger part of the vast continent of South America, with the same species in unverified museum collections reportedly occurring at locations ca. 2500 km apart (e.g., central Bolivia and French Guiana), is puzzling. We regard it as suspicious for two species to occur sympatrically at such distances without distinct differences. For the time being, as many lots in museum collections may have been misidentified, it is here suggested that 1) Corona incisa (Hupé, 1857) is used for occurrences in the southern distribution range (Bolivia, adjacent areas of Peru and Brazil), 2) Corona regalis (Hupé, 1857) for specimens from western Brazil, central and northern Peru, Ecuador and southeastern Colombia, and 3) *Corona regina* (Férussac, 1823) for records from the northwestern distribution range (Brazil, French Guiana, Suriname). Unverified records from these areas have been plotted as *Corona* sp. in the distribution maps. Corona pfeifferi (Hidalgo, 1869) is a species that, within the study area, may be unambiguously recognized. The taxonomy of this group thus urgently needs further revision, preferably with molecular research from samples throughout the distribution range.

### Corona incisa

Taxon classificationAnimaliaStylommatophoraOrthalicidae

(Hupé, 1857)

Figs 40C–D , 42D–E , 43 , 84A–B , 89B

Bulimus incisus Hupé 1857: 36, pl. 9 fig. 1.
Corona incisa var. machadoensis Strebel 1909: 131, pl. 27 figs 412–413.
Corona incisa ; Richardson 1993: 67 (references, synonymy); Simone 2006: 159, fig. 543.
Corona machadoensis Simone 2006: 159, fig. 545.

#### Type locality.

“Bolivie”; see remarks.

#### Type material.

MNHN 28242, lectotype (**design.n**.); MNHN 28068 (4), paralectotypes.

#### Diagnosis.

Shell sinistral or dextral, conic-ovate, solid, changing in ground colour from creamy (top) to tawny (last whorl) with a narrow girdle at the periphery of yellowish, arrow-like markings (>>) and darker sections in between, numerous narrow axial streaks, overlying few broader ones in the basic pattern.

#### Dimensions.

“Alt., 62; diam., 33 mill.”; figured specimen herein shell height 73.8, diameter 33.4 mm.

#### Distribution.

**Peru**, Dept. Madre de Dios, Reserva Los Amigos, Boca Amigo (FML 14940); **Bolivia**, Dept. Beni, Covendo (USNM 361134*, 362864*); ibid., Reyes, Hacienda Shatarona (ANSP 165233*); Dept. La Paz, Chiñiri (ANSP 165232*); ibid., Santa Ana (ANSP 165234*); Dept. Santa Cruz, Amboró (FML 1121). Brasil (Simone 2006).

#### Ecoregion.

Bolivian Yungas [NT0105], Southwest Amazon moist forests [NT0166], Dry Chaco [NT0210], Beni savanna [NT0702].

#### Remarks.

Hupé did not state on how many specimens his description was based; he referred to d’Orbigny 1837 [1834–1847]: pl. 29 figs 4–5. The specimens corresponding to this plate, however, do not match the dimensions given by Hupé. It may be that Hupé made an error when stating the shell height as “Alt., 62”, or that he had both d’Orbigny’s and his own specimens at his disposal during the description; the latter, if present, have not been found. The specimen matching d’Orbigny’s (1837 [1834–1847]: pl. 29) figure 4 has been located in the MNHN collection; it corresponds to the figure of Hupé (1857: pl. 9 fig. 1), and is now designated lectotype (**design.n**.). According to d’Orbigny 1837 [1834–1847]: 258 his material was found “entre cette province [Chiquitos] et celle de Moxos [i.e., northern part of Dept. Cochabamba and southern part of Dept. Beni], dans les forêts inondées une partie de l’année, et qu’habitent les sauvages Guarayos, à la saison des pluies, elle est assez commune”. The variety described by Strebel (1909) was based on material from the Dohrn collection and labelled ‘Rio Machado’. This is both the name for a river in Edo. Minas Gerais and the local name for Río Ji-Paraná in Edo. Rondônia in Brazil. The provenance of Dohrn’s material is unknown and the specimens have not been located. The type material of both *Bulimus incisus* Hupé, 1857 and Corona incisa var. machadoensis Strebel, 1909 is sinistral; however, this is an eniantomorphous species as shown by Simone (2006: fig. 543). The record from Peru is tentatively identified as this species.

### Corona pfeifferi

Taxon classificationAnimaliaStylommatophoraOrthalicidae

(Hidalgo, 1869)

Figs 41A–E , 43 , 89A

Orthalicus pfeifferi Hidalgo 1869b: 412; Hidalgo 1870: 65, pl. 6 fig. 8.
Corona pfeifferi cincta Strebel 1909: 135, pl. 21 fig. 337, pl. 22 figs 356–357; Breure 2013a: 16, figs 18A–B, 18i.
Corona pfeifferi ; Richardson 1993: 68 (references); Breure and Mogollón 2010: 27, figs 2–4, 14, 37–38.

#### Type locality.

[Ecuador, Prov. Pastaza] “Canelos, reipublicae Aequatoris”.

#### Type material.

MACN 15.05/3280 (1), syntype.

#### Additional material.

ZMB 101836 (1), syntype of Corona pfeifferi cincta Strebel, 1909.

#### Dimensions.

Shell height 56.3, diameter 25.0 mm.

#### Diagnosis.

Shell dextral, elongate-ovate, rather solid, creamy ground colour with numerous small axial, partly waving, brown streaks, a few broader and intense brown, peripheral band hardly noticeable or light with few brown markings (<<).

#### Distribution.

**Ecuador**, Prov. Napo, Tena (RBINS); ibid., Tiputini (RBINS); Prov. Pastaza, Canelos; Prov. Tungurahua, Topo (Breure and Borrero 2008). **Peru**, Dept. Loreto, near río Curaray (Breure and Mogollón 2010).

#### Ecoregion.

Eastern Cordillera real montane forests [NT0121], Northwestern Andean montane forests [NT0145].

#### Remarks.

Hitherto this is the only published record of this Ecuadorian species from Peru.

### Corona regalis

Taxon classificationAnimaliaStylommatophoraOrthalicidae

(Hupé, 1857)

Figs 42A–C , 43 , 89B

Bulimus regalis Hupé 1857: 34, pl. 10 fig. 3.
Bulimus loroisianus Hupé 1857: 35, pl. 2 fig. 4.
Corona regalis ; Richardson 1993: 68 (synonymy, references); Simone 2006: 160, fig. 547.
Corona regalis regalis ; Ramírez et al. 2003: 282.
Corona regalis loroisiana ; Ramírez et al. 2003: 282.
Corona loroisiana ; Simone 2006: 159, fig. 544.

#### Type locality.

“le Brésil”.

#### Type material.

Not located.

#### Diagnosis.

Shell sinistral or dextral, solid, ground colour brownish to whitish, the upper whorls gradually turning into pinkish, a dark peripheral band may be present, columellar margin bordered by a dark band, extending in the dark parietal callus.

#### Dimensions.

Shell height 70, diameter 34 mm (*regalis* Hupé), resp. 64 and 30 mm (*loroisianus* Hupé).

#### Distribution.

Colombia (Linares and Vera 2012). **Ecuador**, Prov. Tungurahua, Baños (Breure and Borrero 2008). **Peru**, Dept. Loreto, Pebas (MCZ 156697*); ibid., Santa Clara (USNM *); ibid., Yurimaguas (ANSP 189244*); Dept. San Martín, Moyobamba (ANSP 26166); ibid., Saposoa (ANSP 165231*); ibid., Shapaja (ANSP 165230*); ibid., near Tingo Maria (MCZ 179600*); ibid., near Yarina (MCZ 272904*, 272906*, 272918*); Dept. Huánuco, Aguas Calientes (MCZ 225651*); Dept. Ucayali, río Aguaytia (ANSP 331978*; MCZ 159190*). Brazil (Simone 2006).

#### Ecoregion.

Iquitos varzea [NT0128], Ucayalí moist forests [NT0174].

#### Remarks.

This species shows enantiomorphy and its geographic variation needs more study. The morphological differences with *Corona regina* (Férussac, 1823) seem but marginal, and only a thorough revision may shed further light on the taxonomy of this group.

### Genus Kara

Taxon classificationAnimaliaStylommatophoraOrthalicidae

Strebel, 1910

Thaumastus (Kara) Strebel 1910: 16.

#### Type species.

*Bulimus thompsonii* Pfeiffer, 1845, by monotypy.

#### Diagnosis.

Shell elongate-ovate, imperforate, solid, whorls slightly convex, apex blunt, height up to ca. 70 mm, colour yellowish to (pale) brown, usually with darker axial streaks, protoconch pit-reticulated, teleoconch sculptured with growth striae, sometimes with indistinct spiral impressions, aperture subovate, peristome thin and simple, columellar margin hardly dilated, parietal wall with a thin callus.

#### Distribution.

Ecuador, Peru.

#### Habitat.

Presumably living in leaf litter in (secondary) forests.

#### Phylogenetic data.

Breure and Romero 2012: Kara thompsonii (Pfeiffer, 1845).

#### Remarks.

This taxon was given generic status by Breure (2011).

### Kara cadwaladeri

Taxon classificationAnimaliaStylommatophoraOrthalicidae

(Pilsbry, 1930)

Figs 46A–C , 47

Thaumastus cadwaladeri Pilsbry 1930: 355, pl. 31 fig. 10; Richardson 1995: (references).
Thaumastus (Thaumastus) cadwaladeri ; Ramírez et al. 2003: 282.

#### Type locality.

“Huacapistana, Prov. Junin, Peru”.

#### Type material.

Holotype ANSP 151812.

#### Additional material.

ANSP 453097 (1), paratype.

#### Diagnosis.

Shell elongate, uniformly dark brown coloured on the last whorl, upper whorls somewhat paler, a small white girdle below the crenulate suture, aperture relatively small, columellar margin relatively dilated above.

#### Dimensions.

Shell height 70.2, diameter 27.5 mm.

#### Distribution.

**Peru**, Dept. Junín, Huacapistana; ibid., near Campanillayoc (Zilch 1954: 76).

#### Ecoregion.

Peruvian Yungas [NT0153].

### Kara indentatus

Taxon classificationAnimaliaStylommatophoraOrthalicidae

(da Costa, 1901)

Figs 44C–D

Strophocheilus (Dryptus) indentatus da Costa 1901: 239, pl. 24 fig. 8; Breure and Ablett 2015: 34, 8iii–iv, L9i.
Dryptus indentatus ; Richardson 1995: 200.
Thaumastus (Thaumastus) indentatus ; Breure and Borrero 2008: 8.

#### Type locality.

“Ecuador”.

#### Type material.

Lectotype NHMUK 1907.11.21.115 (Breure and Ablett 2015).

#### Additional material.

NHMUK 1907.11.21.116 (1), paralectotype.

#### Dimensions.

Shell height 44.0, diameter 24.0 mm.

#### Distribution.

**Ecuador**, no precise locality known.

#### Remarks.

As Breure and Ablett (2015) remarked, this species may be closely allied to Kara thompsonii (Pfeiffer, 1845) and *Kara yanamensis* (Morelet, 1863), and upon further studies may prove to be a synonym of either of these species.

### Kara ortiziana

Taxon classificationAnimaliaStylommatophoraOrthalicidae

(Haas, 1955)

Figs 46D–F , 47

Plecocheilus (Eurytus) ortizianus Haas 1955a: 366, fig. 73.
Thaumastus ortizianus ; Richardson 1995: 380 (references).
Thaumastus (Kara) ortizianus ; Ramírez et al. 2003: 282.

#### Type locality.

“near Chancay, between La Colmena and La Esperanza, Peru”.

#### Type material.

FMNH 47083, holotype.

#### Diagnosis.

Shell elongate-ovate, coloured with olive buff with darker brown, axial striae, suture crenulate, height of aperture 0.57 times total shell height, ovate, pointed above, widely rounded below, peristome simple, parietal wall covered by a transparent callus (modified after Haas 1955).

#### Dimensions.

Shell height 60.0, diameter 28.7 mm.

#### Distribution.

**Peru**, Dept. Cajamarca, near Chancay.

#### Ecoregion.

Peruvian Yungas [NT0153].

#### Remarks.

Haas (1955) characterized this species “by the gloss of its shell, which is without any trace of bands or spots”.

### Kara thompsonii

Taxon classificationAnimaliaStylommatophoraOrthalicidae

(Pfeiffer, 1845)

Figs 44A–B , 45A–D , 47 , 88A

Bulimus thompsonii Pfeiffer 1845: 74; Breure and Ablett 2015: 51, figs 8i–ii, L17iii.
Orphnus thompsoni var. lutea Cousin 1887: 212; Breure 2011: 35, figs 7A, 7i.
Orphnus thompsoni var. nigricans Cousin 1887: 212; Breure 2011: 35, figs 7B, 7ii.
Orphnus thompsoni var. olivaceus Cousin 1887: 212; Breure 2011: 36, figs 7C, 7iii.
Orphnus thompsoni var. zebra Cousin 1887: 212; Breure 2011: 42, figs 7D, 7iv.
Thaumastus (Kara) thompsoni [sic]; Breure and Borrero 2008: 7.

#### Type locality.

[Ecuador] “Quito”.

#### Type material.

NHMUK 1975464, lectotype (Breure 1978: 34).

#### Additional material.

NHMUK 1975465 (2), paralectotypes. RBINS/MT2358, lectotype of Orphnus thompsoni var. lutea Cousin; RBINS/MT2363, lectotype of Orphnus thompsoni var. nigricans Cousin; RBINS/MT2366, lectotype of Orphnus thompsoni var. olivaceus Cousin; RBINS/MT2375, lectotype of Orphnus thompsoni var. zebra Cousin.

#### Diagnosis.

Shell (rather) elongate, coloured with yellowish to brown, with light to darker brown, axial streaks, the upper whorls paler, a white girdle below the crenulate suture, aperture height less than half the shell height, peristome simple, whitish, a darker band inside the aperture behind the lip.

#### Dimensions.

Shell height 71.0, diameter 32.0 mm.

#### Distribution.

**Ecuador**, Prov. Azuay, Cuenca, Azogues (Cousin 1887); ibid., San Francisco (RMNH 114279); prov. El Oro, Zaruma (USNM 515471).

#### Ecoregion.

Eastern Cordillera real montane forests [NT0121].

### Kara viriata

Taxon classificationAnimaliaStylommatophoraOrthalicidae

(Morelet, 1863)

Fig. 45F

Bulimus viriatus Morelet 1863: 170, pl. 7 fig. 4.
Thaumastus viriatus ; Richardson 1995: 385 (references).
Thaumastus (Kara) viriatus ; Ramírez et al. 2003: 282.

#### Type locality.

[Peru] “Niguapata (…) la vallée de Santa-Anna”.

#### Type material.

MHNG-INVE-78772 (2), syntypes.

#### Diagnosis.

Shell ovate-conic, colour yellowish [with brownish axial streaks], upper whorls pale, aperture height slightly over half the shell height, peristome simple, whitish, parietal callus thin and translucent-whitish.

#### Dimensions.

Shell height 58.7, diameter 31.8 mm.

#### Distribution.

**Peru**, Dept. Cuzco, ‘Niguapata’.

#### Remarks.

The type locality is likely in the Dept. Cuzco, given the addition of “la vallée de Santa-Anna”; however, it is not mentioned in modern gazetteers. The species was described from specimens denuded of the periostracum. The figured specimen has a trace of it remaining, which suggests the colour pattern described above. Size and shape are very similar to Kara yanamensis (Morelet, 1863), and the variation and distribution of these two taxa need further study.

### Kara yanamensis

Taxon classificationAnimaliaStylommatophoraOrthalicidae

(Morelet, 1863)

Figs 45E , 47

Bulimus yanamensis Morelet 1863: 171, pl. 8 fig. 3; Breure and Ablett 2015: 53, figs 8v–vi, L18ii.
Thaumastus yanamensis ; Richardson 1995: 386 (references).
Thaumastus (Kara) yanamensis ; Ramírez et al. 2003: 282.

#### Type locality.

[Peru] “Yanama”.

#### Type material.

MHNG-INVE-60202, lectotype (Breure 1978: 34).

#### Additional material.

MHNG-INVE-60202 (1), paralectotype; NHMUK 1893.2.4.167–168 (2), paralectotypes.

#### Diagnosis.

Shell ovate-conic, colour yellowish with brownish axial streaks, upper whorls pale, aperture height slightly over half the shell height, peristome simple, whitish, parietal callus thin and translucent-whitish.

#### Dimensions.

Shell height 55.4, diameter 29.5 mm.

#### Distribution.

**Peru**, Dept. Apurimac, Yanama (see remarks).

#### Ecoregion.

Peruvian Yungas [NT0153].

#### Remarks.

There are different places called Yanama in Peru, but probably the type locality is found between Abancay and Andahaylas, as L. Angrand, the collector, travelled along this route. The relationship of this species with Kara viriata (Morelet, 1863) needs more study as the differences are but slight.

### Genus Orthalicus

Taxon classificationAnimaliaStylommatophoraOrthalicidae

Beck, 1837

Orthalicus Beck 1837: 59.

#### Type species.

*Buccinum zebra* Müller, 1774, by subsequent designation (Herrmannsen 1847 [1847–1849]: 159).

#### Description.

Shell ovate-conical, imperforate, rather thin, shell height up to ca. 45–75 mm (study area), colour whitish with usually longitudinal or zigzag stripes, and more or less modified by three equidistant spiral bands, surface with incrassate growth lines, sometimes with spiral lines or rarely with weak malleation, protoconch smooth, whorls hardly convex, suture well impressed, aperture (elongate-)ovate, skewed in side view, peristome thin and simple.

#### Distribution.

U.S.A. (Florida), Mexico, Belize, Guatemala, Honduras, El Salvador, Costa Rica, Panama, Colombia, Ecuador, Peru, Bolivia, Brazil, French Guiana, Surinam, Guyana, Venezuela, Trinidad and Tobago.

#### Habitat.

Species are occurring in dry to humid forests at elevations up to ca. 1500 m.

#### Anatomy.

Lehmann 1864: *Orthalicus undatus* (Bruguière, 1789) [m]; Fischer and Crosse 1870–1878: *Orthalicus longus* Pfeiffer, 1856 [g, m, r]; Binney and Bland 1871: *Orthalicus undatus* [r]; Martens 1873: *Orthalicus obductus* Shuttleworth, 1856 [m, r]; Strebel and Pfeffer 1882: *Orthalicus ferussaci* Martens, 1864 [r], *Orthalicus princeps* (Broderip in Sowerby I and II, 1833 [1832–1841]) [g, m, r], *Orthalicus zoniferus* Strebel and Pfeffer, 1882 [g, m, r]; Pilsbry 1902 [1901–1902]: *Orthalicus undatus jamaicensis* Pilsbry, 1901 [g, p, r], *Orthalicus longus* [m], *Orthalicus princeps* (Broderip in Sowerby I and II, 1833 [1832–1841]) [g], *Orthalicus pulchellus* (Spix in Wagner, 1827) [g]; Breure and Schouten 1985: *Orthalicus ferussaci*, *Orthalicus melanocheilus* (Valenciennes, 1833), *Orthalicus maracaibensis* Pfeiffer, 1856, *Orthalicus princeps*, *Orthalicus undatus*, *Orthalicus zoniferus* Strebel and Pfeffer, 1882) [all g, r].

#### Phylogenetic data.

Breure et al. 2010: *Orthalicus ponderosus* Strebel and Pfeffer 1882.

#### Remarks.

Rehder (1945: 29–31) has elucidated the status of *Buccinum zebra* Müller, which had obscured the taxonomy of this group due to the high variation and many contradicting interpretations in literature. Nevertheless this genus urgently needs a thorough revision using morphological, anatomical and molecular data from specimens throughout the vaste distribution range. Additional to the species listed below, in some museum collections unidentified material of this genus has been listed. Some of these unverified records, collected from precise localities, are plotted as *Orthalicus* sp. in Figure 52.

### Orthalicus bensoni

Taxon classificationAnimaliaStylommatophoraOrthalicidae

(Reeve, 1849)

Figs 48A–E , 52

Bulimus bensoni Reeve 1849 [1848–1850]: pl. 78 fig. 571; Breure and Ablett 2015: 23, figs 12i–ii, L2ii [not 11v–vii].
Orthalicus isabellinus Martens 1873: 190, pl. 1 fig. 8; Breure 2013: 23, fig. 22G–H, 22iv.
Orthalicus bensoni ; Richardson 1995: 98 (references); Ramírez et al. 2003: 282; Simone 2006: 156, fig. 530; Breure and Borrero 2008: 26; Massemin et al. 2009: 406, pl. 5E; Linares and Vera 2012: 151.

#### Type locality.

“Banks of the Amazon”.

#### Type material.

NHMUK 1975582 (1), syntype.

#### Additional material.

ZMB 8876 (2), syntypes of *Orthalicus isabellinus* Martens.

#### Diagnosis.

Shell sculptured with dense spiral lines, colour pattern predominantly with three small spiral bands of dark reddish-brown interrupted with white << marks, aperture with a small dark band behind the lip, around the columellar margin, and on the parietal wall.

#### Dimensions.

Shell height 66.6, diameter 35.0 mm.

#### Distribution.

Colombia. **Ecuador**, Prov. Napo, Sarayacu (Pilsbry 1899: 148); Río Napo (CMC C10806). **Peru**, no specific locality (Martens 1873). Brazil (Simone 2006). French Guiana (Massemin et al. 2009). Suriname (Altena 1975).

#### Ecoregion.

Eastern Cordillera real montane forests [NT0121].

#### Remarks.

The figures by Breure and Ablett 2015: figs 11v–vii are not of the syntype; this error is corrected herein. This species seems only slightly different from the Colombian Orthalicus bifulguratus (Reeve, 1849). The Peruvian record is based on the specimens described by Martens as *Orthalicus isabellinus*, which were collected by Tschudi at an unspecified locality (Breure 2013). Strebel (1909: 29) remarked that these subadult shells were not quite typical; however, we are of the opinion that Martens’ and Reeve’s taxa may be synonymous, although some doubt remains. The species is evidently widely distributed within the Amazon river basin, although molecular research may show that the Peruvian population is distinct from the eastern forms; in that case Martens’ taxon should be resurrected.

### Orthalicus bifulguratus

Taxon classificationAnimaliaStylommatophoraOrthalicidae

(Reeve, 1849)

Figs 50A–C , 51A–B , 52

Bulimus bifulguratus Reeve 1849 [1848–1850]: pl. 82 fig. 606; Breure and Ablett 2015: 24, fig. L2iii [not L2i–ii].
Zebra fulgur Miller 1878: 186; Miller 1879: pl. 6 figs 1a–b.
Orthalicus bifulguratus ; Breure and Schouten 1985: 29 (lectotype designation); Richardson 1993: 98 (references); Linares and Vera 2012: 151.

#### Type locality.

[Colombia] “Andes of Columbia”.

#### Type material.

NHMUK 20140082, lectotype (Breure and Schouten 1985: 29) (Cuming coll.).

#### Diagnosis.

Shell sculptured with a dense pattern of fine spiral lines, coloured with pairs of yellow, irregularly waving and zigzag, longitudinal bands bordered with dark brown on the right side.

#### Dimensions.

Shell height 56.9, diameter 32.8 mm.

#### Distribution.

Colombia. **Ecuador**, Prov. El Oro, 10.2 km W Pinas (UF 26616); Prov. Pichincha, Milpe (ANSP 170727); ibid., San Nicolás (RBINS); Prov. Tungurahua, Topo (FMNH 86649).

#### Ecoregion.

Eastern Cordillera real montane forests [NT0121], Northwestern Andean montane forests [NT0145].

#### Remarks.

Breure and Ablett (2015) noted that the lectotype, for which the dimensions are given above, is probably not full-grown. Their figures L2i–ii illustrate Orthalicus bensoni (Reeve, 1849); this error is now redressed by figuring the correct shell. Also the specimen of *Zebra fulgur* Miller, 1878 was subadult as Miller gave the shell height as 50 mm. Cousin (1887) mentioned a specimen from San Nicolás, which was located in the RBINS collection. In the same collection a specimen was found with locality “Cabiloña, montaña / près Ambato”; we have been unable to find this locality in modern gazetteers.

### Orthalicus mars

Taxon classificationAnimaliaStylommatophoraOrthalicidae

Pfeiffer, 1861

Figs 49D–F

Orthalicus mars Pfeiffer 1861: 25, pl. 2 fig. 8; Simone 2006: 156, fig. 533; Breure and Borrero 2008: 8; Breure and Ablett 2015: 40, figs 13v–vi, L13i.

#### Type locality.

“republica Aequatoris”.

#### Type material.

NHMUK 20100504 (3), syntypes.

#### Diagnosis.

Shell ovate-conical, upper whorls with a colour pattern of longitudinal, dark brown streaks, broad on the lower half, forked on the upper half of whorl, separated by whitish ‘3-like’ shapes, pattern fading on the last whorls, aperture dark brown bordered inside, columellar margin and parietal callus also dark brown.

#### Dimensions.

Shell height 76.6, diameter 38.4 mm.

#### Distribution.

**Ecuador**, without precise locality. Brazil (Simone 2006).

#### Remarks.

This species, mentioned from Edo. Amazonas in Brazil by Simone (2006), may occur in the easternmost part of Ecuador. Compared to Orthalicus phlogerus (d’Orbigny, 1835) this species is relatively stout, the aperture bordered by dark colours.

### Orthalicus phlogerus

Taxon classificationAnimaliaStylommatophoraOrthalicidae

(d’Orbigny, 1835)

Figs 49A–C , 52

Helix phlogera d’Orbigny 1835: 8; Breure and Ablett 2015: 44, figs 13iii–iv, L15iii.
Bulimus phlogerus ; d’Orbigny 1837 [1834–1847]: pl. 29 figs 6–8; d’Orbigny 1838 [1834–1847]: 259.
Orthalicus phlogerus ; Richardson 1993: 108 (references).

#### Type locality.

“provincia Chiquitensi (republica Boliviana)”.

#### Type material.

NHMUK 1854.12.4.86 (6), syntypes.

#### Diagnosis.

Shell elongate-conical, upper whorls with a colour pattern of longitudinal, dark brown streaks, broad on the lower half, forked on the upper half of whorl, separated by whitish ‘3-like’ shapes, pattern fading on the last whorl, aperture whitish.

#### Dimensions.

Shell height 59.8, diameter 26.8 mm.

#### Distribution.

**Bolivia**, Dept. Santa Cruz, between San Javier and Concepción.

#### Ecoregion.

Chiquitano dry forests [NT0212].

#### Remarks.

Compared to Orthalicus mars Pfeiffer, 1861 this species is smaller, slenderer, and has the apeture in lighter colours.

### Orthalicus pulchellus

Taxon classificationAnimaliaStylommatophoraOrthalicidae

(Spix in Wagner, 1827)

Figs 50C–D , 52

Achatina pulchella Spix in Wagner 1827: 9, pl. 9 fig. 2.
Orthalicus puchellus ; Richardson 1993: 111 (references); Simone 2006: 157, figs 536, 537 (as Orthalicus undatus); Massemin et al. 2009: 408, pl. 5D.

#### Type locality.

[Brazil, Para] “in sylvis Provinciae Paraënsis”.

#### Type material.

ZSM 20020203, syntype (1).

#### Diagnosis.

Shell marked with narrow, dark brown longitudinal stripes, spaced at equal distances, bent a little below the suture, and at the position of three spiral bands of dark brown interrupted by yellowish-whitish << marks, aperture with a small, dark brown band behind the lip, parietal callus also dark brown (modified after Pilsbry 1899: 135–136).

#### Dimensions.

Shell height 47.9, diameter 29.1 mm.

#### Distribution.

?Colombia (Linares and Vera 2012: 155). **Bolivia**, Dept. Santa Cruz, near Santiago de Chiquitos (UF 40539, 40541, 40556). Paraguay (Simone 2006). Brazil (Simone 2006). French Guiana (Massemin et al. 2009). Suriname (Pilsbry 1899: 136; not in Altena 1975). ?Venezuela (Simone 2006).

#### Ecoregion.

Chiquitano dry forests [NT0212].

#### Remarks.

This Brazilian species has an enormous distribution range given the records mentioned above. Some of these, especially of Venezuela and Colombia, need to be viewed with much suspicion and further evidence is needed as misidentifications are likely. The shell figured by Massemin et al. 2009: pl. 5 fig. D has the stripes more waving and partly confluenced.

### Genus Porphyrobaphe

Taxon classificationAnimaliaStylommatophoraOrthalicidae

Shuttleworth, 1856

Porphyrobaphe Shuttleworth 1856: 70.

#### Type species.

*Bulimus iostomus* Sowerby I, 1824, by subsequent designation (Martens in Albers, 1860: 227).

#### Description.

Shell ovate-conical, imperforate, rather solid, up to ca. 60–80 mm (study area), groundcolour yellowish to tawny, with longitudinal streaks (or with an irregular zigzag pattern or with irregular spots), protoconch smooth, teleoconch sculptured with growth striae and spiral impressions, which may be either closely set or at larger intervals, aperture ovate, columellar margin straight, peristome expanded and narrowly reflexed.

#### Distribution.

Colombia, Ecuador, Peru.

#### Habitat.

As far as known mainly found in leaf litter, but some species are (also) tree-inhabiting.

#### Anatomy.

Fischer and Crosse 1870–1878: Porphyrobaphe (Porphyrobaphe) iostoma (Sowerby I, 1824) [m, r]; Breure and Schouten 1985: Porphyrobaphe (Porphyrobaphe) iostoma [g, r], Porphyrobaphe (Oxyorthalicus) iris (Pfeiffer, 1853) [g, h, r], Porphyrobaphe (Oxyorthalicus) irrorata (Reeve, 1849) [g, r].

#### Phylogenetic data.

Breure et al. 2010: Porphyrobaphe (Porphyrobaphe) iostoma (Sowerby I, 1824).

### Subgenus Porphyrobaphe (Oxyorthalicus)

Taxon classificationAnimaliaStylommatophoraOrthalicidae

Strebel, 1909

Porphyrobaphe (Oxyorthalicus) Strebel 1909: 117.

#### Type species.

*Bulimus irrorata* Reeve, 1849, by original designation (Strebel 1909: 102).

#### Diagnosis.

Shell upper whorls pointed, spiral sculpture rather strong, cutting the growth striae into oblong granules, height of last whorl ca. 0.8 shell height, aperture without columellar fold.

#### Distribution.

Colombia, Ecuador.

### Porphyrobaphe (Oxyorthalicus) irrorata

Taxon classificationAnimaliaStylommatophoraOrthalicidae

(Reeve, 1849)

Figs 53A–B , 55A–B , 56

Bulimus irrorata Reeve 1849 [1848–1850]: pl. 62 fig. 427; Breure and Ablett 2015: 36, figs 15i–ii, L10iii.
Dryptus irroratus var. β elongata Miller 1878: 179; Miller 1879: pl. 2 fig. 2a.
Dryptus irroratus var. γ minor Miller 1878: 180; Miller 1879: pl. 2 fig. 2b.
Porphyrobaphe irroratus ; Richardson 1993: 119 (references, synonymy).
Porphyrobaphe (Oxyorthalicus) irrorata ; Breure and Borrero 2008: 28.

#### Type locality.

“Brazil? New Granada?”.

#### Type material.

NHMUK 1975248 (3), syntypes.

#### Diagnosis.

Shell yellowish with an irregular pattern of tawny spots and longitudinal streaks. Aperture white.

#### Dimensions.

Shell height 77.0, diameter 44.0 mm.

#### Distribution.

Colombia (Linares and Vera 2012). **Ecuador**, Prov. Napo, “Oriente”; Prov. Pastaza, Puyo; ibid., Mera; Prov. Pichincha, 71.7 km SW Quito, road to Santo Domingo; ibid., Santo Domingo; ibid., Mindo; ibid., near Nanegal; ibid., Gualea; ibid., Rio Cinto; Río Pilaton valley; Prov. Tungurahua, Topo (all Breure and Borrero 2008).

#### Ecoregion.

Eastern Cordillera real montane forests [NT0121], Northwestern Andean montane forests [NT0145].

#### Remarks.

The Colombian records by Linares and Vera (2012) are based on unverified material and need confirmation as far as they are not adjacent to the distribution range in Ecuador (i.e. their record from Antioquia is likely a misidentification).

### Porphyrobaphe (Oxyorthalicus) subirrorata

Taxon classificationAnimaliaStylommatophoraOrthalicidae

(da Costa, 1898)

Figs 54A–C , 56

Strophocheilus (Eurytus) subirroratus da Costa 1898: 83, fig. II.
Porphyrobaphe subirroratus ; Richardson 1993: 120 (references).
Porphyrobaphe (Oxyorthalicus) subirroratus ; Breure and Borrero 2008: 29.

#### Type locality.

“Paramba, Ecuador”.

#### Type material.

NHMUK 1907.11.21.114, lectotype (Breure and Schouten 1985: 54).

#### Additional material.

ANSP 220422 (1), paralectotype.

#### Diagnosis.

Shell yellowish-brown with a close pattern of longitudinal stripes, partly forked, upper whorls uniformly brown, spiral sculpture dense on last whorl, peristome whitish, parietal callus dark-whitish.

#### Dimensions.

Shell height 62.6, diameter 36.6 mm.

#### Distribution.

**Ecuador**, Prov. Carchi, Hacienda Paramba; Prov. Pastaza, Mera (Breure and Borrero 2008).

#### Ecoregion.

Northwestern Andean montane forests [NT0145].

### Subgenus Porphyrobaphe (Porphyrobaphe)

Taxon classificationAnimaliaStylommatophoraOrthalicidae

Shuttleworth, 1856

#### Diagnosis.

Shell sculpture with weak or without spiral striation, height of last whorl ca. 0.7 shell height, aperture with (weak) columellar fold.

### Porphyrobaphe (Porphyrobaphe) iostoma

Taxon classificationAnimaliaStylommatophoraOrthalicidae

(Sowerby I, 1824)

Figs 55D–F , 56 , 84C , 86A–C

Bulimus iostoma Sowerby I 1824: 58, pl. 5 fig. 1.
Bulimus grevillei Pfeiffer 1875 [1870–1876]: 143, pl. 133 figs 4–5.
Porphyrobaphe iostomus ; Richardson 1993: 118 (references, synonymy); Ramírez et al. 2003: 282.
Porphyrobaphe (Porphyrobaphe) iostoma ; Breure and Borrero 2008: 28.

#### Type locality.

No type locality given.

#### Type material.

Not located.

#### Diagnosis.

Shell with a colour pattern of irregularly spaced spots, partly forming longitudinal streaks, especially on upper whorls, aperture broadly ovate, peristome thick, typically purple but may be whitish, expanded and reflexed.

#### Dimensions.

Shell height 60.3, diameter 31.7 mm.

#### Distribution.

Colombia (Linares and Vera 2012). **Ecuador**, Prov. El Oro, near Machala; ibid., Chacras; ibid., Santa Rosa; Prov. Esmeraldas, various localities; Prov. Guayas, 5 km N Santa Elena (Breure and Borrero 2008); Prov. Manabí, Jama (NHMUK 20150529). **Peru**, Dept. Piura, La Laja (Schileyko 1999); Dept. Tumbes, Lechugal (Sztolcman leg., Pilsbry 1899: 151); ibid., Matapalo (USNM 666039*).

#### Ecoregion.

Western Ecuador moist forests [NT0178], Ecuadorian dry forests [NT0214], Tumbes-Piura dry forests [NT0232], South American Pacific mangroves [NT1405].

#### Remarks.

This is a very characteristic species which can hardly be mistaken. However, the record for Colombia from Linares and Vera (2012), based on unverified material, needs confirmation. The colour pattern may be faded away in living specimens (Fig. 86A–C).

### Porphyrobaphe (Porphyrobaphe) saturnus

Taxon classificationAnimaliaStylommatophoraOrthalicidae

(Pfeiffer, 1860)

Figs 53C–E , 56

Bulimus saturanus Pfeiffer 1860: 136 [*lapsus calami*, see Breure and Ablett 2015: 49].
Bulimus saturnus Pfeiffer 1860: pl. 51 fig. 6; Breure and Ablett 2015: 49, figs 15iii–v, L17v.
Porphyrobaphe (Porphyrobaphe) saturnus ; Breure and Borrero 2008: 28.

#### Type locality.

“Pallatanga, Republic of Ecuador”.

#### Type material.

NHMUK 20140080 (3), syntypes.

#### Diagnosis.

Shell with longitudinal pattern of stripes and some spots, peristome and parietal wall dark brown.

#### Dimensions.

Shell height 75.8, diameter 38.4 mm.

#### Distribution.

**Ecuador**, Prov. Chimborazo, Pallatanga; ibid., Riobamba; Prov. El Oro, 10 km S Piñas; ibid., 6 km N Zaruma; Prov. Loja, Malacatos (all Breure and Borrero 2008).

#### Ecoregion.

Eastern Cordillera real montane forests [NT0121], Northwestern Andean montane forests [NT0145].

### Genus Quechua

Taxon classificationAnimaliaStylommatophoraOrthalicidae

Strebel, 1910

Thaumastus (Quechua) Strebel 1910: 17.

#### Type species.

*Bulimus salteri* Sowerby III, 1890, by original designation.

**Description.** Shell elongate-ovate, imperforate or rimate, rather solid, up to ca. 50–100 mm, groundcolour flesh-coloured to yellowish with dark brown longitudinal streaks, upper whorls pale, apex sunken, protoconch with axial riblets and wrinkles, more or less anastomosing, teleoconch with growth striae and (usually light) spiral impressions, aperture elongate-ovate, peristome thin and simple.

#### Distribution.

Peru.

#### Habitat.

The species live in montane forests at 800–ca. 3000 m.

#### Anatomy.

Zilch 1953: Quechua salteri (Sowerby III, 1890) [g, m, r]; Breure 1978: Quechua taulisensis (Zilch, 1953) [g, h, r].

#### Remarks.

Breure and Ablett (2015: 20) elevated this group as separate genus and tentatively placed it in the family Orthalicidae. Further studies are needed to corroborate this position.

### Quechua olmosensis olmosensis

Taxon classificationAnimaliaStylommatophoraOrthalicidae

(Zilch, 1954)

Figs 58A , 59

Thaumastus (Quechua) olmosensis Zilch 1954: 76, pl. 6 figs 10–11; Ramírez et al. 2003: 282; Neubert and Janssen 2004: 220, pl. 2 fig. 15.
Thaumastus olmosensis ; Richardson 1995: 380 (references).

#### Type locality.

“Peru, am Weg von Olmos nach Jaén, der den nur 2144 m hohe Pass Abra Porculla überschreitet”.

#### Type material.

SMF 123653, holotype.

#### Additional material.

SMF 123654 (20), SMF 123655 (5), SMF 123656 (3), paratypes.

#### Diagnosis.

Shell with an irregular pattern of lighter and darker brown stripes, aperture ear-shaped, light coloured inside, parietal callus whitish-transparent.

#### Dimensions.

Shell height 91.5, diameter 42.0 mm.

#### Distribution.

**Peru**, Dept. Lambayeque, east of Olmos.

#### Ecoregion.

Tumbes-Piura dry forests [NT0232].

#### Remarks.

The species was collected in “Lichter Bergwald in 840 m Höhe” by Koepcke. The pass Abra de Poculla is located at 5°50'S, 79°30'W (contrary to the data given by Zilch 1954), and an elevation of 840 m on the road to Jaén, east of Olmos, is reached at ca. 5°55'30"S, 79°32'23"W.

### Quechua olmosensis maxima

Taxon classificationAnimaliaStylommatophoraOrthalicidae

(Weyrauch, 1967) stat. n.

Figs 58B , 59

Thaumastus (Quechua) salteri maximus Weyrauch 1967: 347, fig. 135; Ramírez et al. 2003: 282; Barbosa et al. 2008: 272; Breure 2012a: 10.
Thaumastus (Quechua) maximus ; Neubert and Janssen 2004: 217, pl. 2 fig. 13.

#### Type locality.

“Norte de Perú interandino, Peña Blanca, en el camino de herradura de Sócota a San Andrés, 25 km NE Cutervo, 2600 m”.

#### Type material.

SMF 156381, holotype.

#### Additional type material.

FML 3202 (1), paratype.

#### Diagnosis.

Differs from the nominal taxon by the larger size and the darker aperture.

#### Dimensions.

Shell height 99.4, diameter 47.3 mm.

#### Distribution.

**Peru**, Dept. Cajamarca, Peña Blanca.

#### Ecoregion.

Peruvian Yungas [NT0153], Marañon dry forests [NT0223].

#### Remarks.

This taxon was described as a subspecies of Quechua salteri (Sowerby III, 1890); Neubert and Janssen (2004) considered it as a distinct species. Upon comparison with both the types from Quechua salteri and *Quechua olmosensis* (Zilch, 1954), it appears very similar in its external morphology to the latter, and quite distinct to the former. Given the difference in size and the dislocation of the type locality, Weyrauch’s taxon is now considered as Quechua olmosensis maxima **(stat. n.)**.

### Quechua salteri

Taxon classificationAnimaliaStylommatophoraOrthalicidae

(Sowerby III, 1890)

Figs 57B , 59

Bulimus salteri Sowerby III 1890: 578, pl. 50 fig. 4; Breure and Ablett 2015: 47, figs 13i–ii, L17i.
Thaumastus salteri ; Richardson 1995: 381 (references [partim]).
Thaumastus (Quechua) salteri salteri ; Ramírez et al. 2003: 282.

#### Type locality.

“Catamarca, Andes Peruviae”.

#### Type material.

NHMUK 1907.11.21.118, lectotype (Breure 1979: 45).

#### Diagnosis.

Shell sculptured with irregular longitudinal and spiral striation, giving a malleated appearance, with brown markings and a few longitudinal streaks, aperture pale-purple inside, parietal callus transparent.

#### Dimensions.

Shell height 69.9, diameter 35.2 mm.

#### Distribution.

**Peru**, Dept. Cajamarca, Chota (ANSP 183257).

#### Ecoregion.

Peruvian Yungas [NT0153].

#### Remarks.

Sowerby also mentioned a variety which was hardly malleated and reached a larger size. The variation and distribution of this species needs further studies.

### Quechua taulisensis

Taxon classificationAnimaliaStylommatophoraOrthalicidae

(Zilch, 1953)

Figs 57A , 59

Thaumastus (Quechua) taulisensis Zilch 1953: 52, pl. 14 fig. 2; Ramírez et al. 2003: 282; Neubert and Janssen 2004: 231, pl. 2 fig. 14.
Thaumastus taulisensis ; Richardson 1995: 383 (references).

#### Type locality.

[Peru] “Bergurwald der Hacienda Taulis”.

#### Type material.

SMF 111465, holotype.

#### Additional material.

SMF 111466 (22), paratypes.

#### Diagnosis.

Shell rather thin, with inconspicuous sculpture of spiral striation, parietal callus transparent.

#### Dimensions.

Shell height 60.0, diameter 27.4 mm.

#### Distribution.

**Peru**, Dept. Cajamarca, Hacienda Taulis (ca. 6°50'S 79°10'W).

#### Ecoregion.

Peruvian Yungas [NT0153].

#### Remarks.

Zilch compared this species with Thaumastus (Quechua) salteri, but said it differs by having a smaller and slenderer shell, which is less sculptured and with a relatively smaller aperture.

### Genus Scholvienia

Taxon classificationAnimaliaStylommatophoraOrthalicidae

Strebel, 1910

Scholvienia Strebel 1910: 20.
Scholvienia (Thomsenia) Strebel 1910: 26, **syn. n.**

#### Type species.

Bulimus bitaeniatus Nyst, 1845, by subsequent designation (Pilsbry 1932: 391).

#### Description.

Shell elongate-ovate, rimate, rather solid, shell height up to ca. 35–62 mm, colour uniformly (chestnut-)brown, in some species with few spiral bands, protoconch with axial, waving riblets, on the lower part becoming split or broken up in wrinkles, teleoconch with incrassate growth striae, in some species becoming thickened at irregular distances, in some species (additionally) crossed by spiral striation, aperture ovate, relatively small, peristome thin and simple, slightly sinuate in side view.

#### Distribution.

Peru.

#### Habitat.

In the leaf litter layer of open montane forest and steppe vegetation.

#### Anatomy.

Breure 1978: Scholvienia alutacea(Reeve, 1850) [g], Scholvienia bifasciata (Philippi, 1845) [g, h, r], Scholvienia gittenbergerorum (Breure, 1978) [g].

#### Remarks.

See the remarks under Scholvienia claritaeStrebel, 1910 for the synonymization of Thomsenia Strebel, 1910. The occurrence of several morphologically similar species in the Tarma and Chanchamayo regions deserves further study, including anatomical and molecular research.

### Scholvienia alutacea

Taxon classificationAnimaliaStylommatophoraOrthalicidae

(Reeve, 1850)

Figs 60A–D , 64A–D , 66

Bulimus alutaceus Reeve 1849 [1848–1850]: pl. 72 fig. 522; Breure and Ablett 2015: 22, figs 10i–iv, L1iii.
Bulimus tarmensis Philippi 1867: 70.
Scholvienia jaspidea minor Strebel 1910: 24, pl. 3 figs 31–32, 36. **syn. n.**
Bulimulus (Protoglyptus) weeksi Pilsbry 1930: 357, pl. 31 fig. 9.
Thaumastus alutaceus ; Richardson 1995: 371 (references, synonymy).
Thaumastus (Scholvienia) alutaceus ; Ramírez et al. 2003: 282.
Thaumastus (Scholvienia) tarmensis tarmensis ; Ramírez et al. 2003: 282.
Thaumastus (Scholvienia) tarmensis weeksi ; Ramírez et al. 2003: 282.

#### Type locality.

[Peru] “Cuzco, Bolivia”.

#### Type material.

NHMUK 1975148, lectotype (Breure 1978).

#### Additional material.

NHMUK 1975149 (1), paralectotype.

#### Diagnosis.

Shell with brownish groundcolour and one, white peripheral band, sculptured with narrow, longitudinal, irregularly thickened, densely placed rib-like striae (Fig. 59D).

#### Dimensions.

Shell height 35.5, diameter 16.5 mm.

#### Distribution.

**Peru**, Dept. Amazonas, Yambrasbamba (NHMUK 1928.12.6.77–93); Dept. Junín, near Tarma and La Oroya.

#### Remarks.

Weyrauch (1964: 46) argued that the type locality is probably in error; this species has not been re-found in the Cuzco area nor in Bolivia, and neither has any similar species. The taxa from Philippi and Pilsbry were described from “ad Oroya haud procul ab oppido Tarma”, respectively [La] Oroya. The specimens on which Strebel based his Scholvienia jaspidea forma minor were collected at “Quimia”, which might be a misspelling for Quenua at ca. 4000 m in the same general area. The shells figured fit into the variation shown by Scholvienia alutacea (Reeve). We found a lot from Yambrasbamba, north of Chachapoyas, which we tentatively refer to this species. Further studies should clarify the distribution and systematic position of Reeve’s taxon.

### Scholvienia bambamarcaensis

Taxon classificationAnimaliaStylommatophoraOrthalicidae

(Breure, 1978)

Figs 61D–F , 66

Thaumastus (Scholvienia) bambamarcaensis Breure 1978: 41, pl. 6 fig. 8; Ramírez et al. 2003: 282.
Thaumastus bambamarcaensis ; Richardson 1995: 372 (references).

#### Type locality.

“Peru, Dept. Cajamarca, 7 km SW Bambamarca, 2920 m”.

#### Type material.

UF 22752, holotype.

#### Additional material.

RMNH 55188 (9), paratypes; UF 22778 (14), paratypes.

#### Diagnosis.

Shell with rather convex sides, height/diameter ratio 2.2, russet-brown with a yellowish subsutural band, sculptured with fine spiral striation, height of aperture more than 0.4 times shell height.

#### Dimensions.

Shell height 44.0, diameter 21.5 mm.

#### Distribution.

**Peru**, Dept. Cajamarca, near Bambamarca.

#### Ecoregion.

Peruvian Yungas [NT0153].

### Scholvienia bifasciata

Taxon classificationAnimaliaStylommatophoraOrthalicidae

(Philippi, 1845)

Figs 57C–E , 65C–F , 66

Bulimus bifasciatus Philippi 1845a [1845–1847]: 10, pl. 3 fig. 5.
Bulimus bitaeniatus Nyst 1845: 153. New name for Bulimus bivittatus Philippi, 1845 not Bulinus bivittatus Sowerby I, 1833.
Bulimus bivittatus Philippi 1845 [1845–1847]: 62.
Thaumastus (Scholvienia) bitaeniatus pallida Strebel 1910: 22, pl. 3 figs 29–30; Ramírez et al. 2003: 282.
Thaumastus (Quechua) tetricus Haas 1951: 523, fig. 110; Ramírez et al. 2003: 282. **syn. n.**
Thaumastus bitaeniatus ; Richardson 1995: 372 (references, synonymy).
Thaumastus tetricus ; Richardson 1995: 385 (references).
Thaumastus (Scholvienia) bifasciatus bifasciatus ; Ramírez et al. 2003: 282.
Thaumastus (Scholvienia) bitaeniatus bitaeniatus ; Ramírez et al. 2003: 282.

#### Type locality.

[Peru] “sylvae peruanae”.

#### Type material.

Not located.

#### Additional material.

FMNH 30920, holotype of Thaumastus (Quechua) tetricus Haas, 1951.

#### Diagnosis.

Shell with straight sides, russet-brown with two yellowish bands (on the last whorl one subsutural, one peripheral), height of aperture 0.4 times shell height or less.

#### Dimensions.

Shell height 50.1, diameter 24.0 mm.

#### Distribution.

**Peru**, Dept. Junín, Chanchamayo valley (Strebel 1910); ibid., Huacapistana (Haas 1951).

#### Ecoregion.

Peruvian Yungas [NT0153].

#### Remarks.

Philippi wrote a paper describing several new species, one of which was *Bulimus bivittatus*, which he sent to the Archiv für Naturgeschichte; the publication date of this journal is not stated and, although it was likely earlier, following Art. 21.3 ICZN has to be assumed as 31 December 1845 (Philippi 1845b). A little later he used the same text, supplemented with a correction—“*Bulimus bivittatus* (ein Schreibfehler für *bifasciatus*)”, thus intended as an author’s emendation—, a commentary and figures, for a part of his ‘Abbildungen’ (Philippi 1845a [1845–1847]: 10); according to Coan and Kabat (2015) this part was published in March 1845. About the same time Nyst published a replacement name for *Bulimus bivittatus*, viz. *Bulimus bitaeniatus* (Nyst 1845: 153); Menke (1845: 95) wrote “Unter dem Postzeichen Löwen (Louvain), 17. April 1845 ist mir eine schätzbare kleine Abhandlung “*Description de deux Bulimes nouveaux de la Colombie*, *par* H. Nyst, *membre de l’Académie* (*royale de Bruxelles*), als *Extrait du tom. XII nr. 3 des Bulletins*, von dem verehrlichen Herrn Verf[asser] freundlichst zugesendet worden”. From this statement it may be deduced that Nyst’s paper was published early April, and thus Philippi’s *Bulimus bifasciatus* has priority. We conclude that this name is the first published available name, which has predominantly been used by later authors (Pfeiffer 1846: 53, Pfeiffer 1848: 199, Albers 1850: 161, Troschel 1852: 1992, Pfeiffer 1853: 425, Adams and Adams 1855 [1854–1858]: 158, Pfeiffer 1856: 148, Hupé 1857: 30, Pfeiffer 1859: 487, Martens in Albers 1860: 193, Martens 1867: 141, Pfeiffer 1868: 132, Hidalgo 1870: 46, Hidalgo 1872: 68, Pfeiffer 1877: 169, Lubomirski 1880: 722, Paetel 1889 [1888–1890]: 208, Ramírez et al. 2003: 282). Nyst’s name has been used by Pilsbry 1895 [1895–1896]: 59, who stated “From present information, it appears that Nyst was the first to change the preoccupied name originally proposed by Philippi”; as we have shown above, this was erroneous. Subsequent authors have followed Pilsbry (viz. E.A. Smith 1904: 4, Strebel 1910: 22, Pilsbry 1932: 391, Haas 1955b: 309, Zilch 1960: 477, Breure 1978: 41, Richardson 1995: 372, Ramírez et al. 2003: 282). Consequently, we are using Philippi’s original name again. Strebel’s forma *pallida* is a slightly smaller shell than Philippi’s type, but seems to fall within the variation.Thaumastus (Quechua) tetricus Haas, 1951 was described from the same region (“Huacapistana on Rio Tarma, Junin Prov., Peru”), is slightly larger but falls within the variation of Philippi’s taxon. It is now considered a junior subjective synonym **(syn. n.)**.

This species is similar in its external morphology to Scholvienia alutacea (Reeve, 1850), *Scholvienia iserni* (Philippi, 1867), *Scholvienia jelskii* (Lubomirski, 1880), and *Scholvienia weyrauchi* (Pilsbry, 1944). The variation and distribution of these taxa in the wider area around Tarma needs further study, including anatomical and molecular research.

### Scholvienia brephoides

Taxon classificationAnimaliaStylommatophoraOrthalicidae

(d’Orbigny, 1835)

Figs 60E–G , 66

Helix brephoides d’Orbigny 1835: 17; Breure and Ablett 2015: 25, figs 10v–vii, L3ii.
Bulimus bifasciatus unicolor Philippi 1869: 36 (**syn. n.**).
Thaumastus brephoides ; Richardson 1995: 373 (references).
Thaumastus (Scholvienia) brephoides ; Ramírez et al. 2003: 282.
Thaumastus (Scholvienia) bifasciatus unicolor ; Ramírez et al. 2003: 282.

#### Type locality.

[Peru] “republica Peruviana”.

#### Type material.

NHMUK 1854.12.4.117, lectotype (Breure and Ablett 2015: 25).

#### Diagnosis.

Shell with rather convex sides, height/diameter ratio 2.0, unicoloured light brown with a paler zone below the suture, peristome rather thick, simple.

#### Dimensions.

Shell height 51.9, diameter 25.1 mm.

#### Distribution.

**Peru**, Dept. Junín, “Prov. Huancayo” (Pilsbry 1895 [1895–1896]: 57); Dept. Huancavelica [?], Huaribamba (Philippi 1869).

#### Ecoregion.

Peruvian Yungas [NT0153].

#### Remarks.

Phillipi’s variety *unicolor* was described as an unbanded form from Huaribamba, which is found at ca. 3100 m elevation in Dept. Huancavelica or at ca. 4900 m in Dept. Junín. The former is adjacent to Prov. Huancayo mentioned by Pilsbry (1895 [1895–1896]). Philippi compared his taxon to Scholvienia brephoides (d’Orbigny), which is also unicoloured. Philippi’s unfigured form—for which no dimensions were given and of which the type has not been located—has been treated as subspecies of the nominate form, but is herein considered as junior subjective synonym of d’Orbigny’s species.

### Scholvienia claritae

Taxon classificationAnimaliaStylommatophoraOrthalicidae

(Strebel, 1910)

Figs 65G , 67

Thomsenia claritae Strebel 1910: 27, pl. 2 fig. 16.
Thaumastus claritae ; Richardson 1995: (references).
Thaumastus (Scholvienia) claritae ; Ramírez et al. 2003: 282.

#### Type locality.

“Chanchamayo, Peru”.

#### Type material.

Not located, see remarks.

#### Diagnosis.

Shell relatively large, and slender (height/diameter ratio 2.3), uniformly “kaffee-braun”.

#### Dimensions.

Shell height 61.2, diameter 28.0 mm.

#### Distribution.

**Peru**, Dept. Junín, Chanchamayo valley.

#### Ecoregion.

Peruvian Yungas [NT0153].

#### Remarks.

This species, described from a single, (supposedly subadult) shell in the O. Semper collection, was used by Strebel to erect a monotypic subgenus *Thomsenia*. Breure (1979: 46) pointed out that the type material was probably lost during World War 2, and treated this taxon as *nomen inquirendum*. He suggested it might belong to *Scholvienia*. Strebel (1910: 26) said both protoconch and teleoconch sculpture were the same as in *Scholvienia porphyria* (Pfeiffer, 1847), *Scholvienia jaspidea* (Morelet, 1863), *Scholvienia jelskii* (Lubomirski, 1880), *Scholvienia iserni* (Philippi, 1867), and *Scholvienia huancabambensis* Strebel, 1910. Therefore. we fail to see the need for a separate subgenus, and *Thomsenia* is now considered a junior subjective synonym of Scholvienia Strebel, 1910 **(syn. n.)**.

### Scholvienia gittenbergerorum

Taxon classificationAnimaliaStylommatophoraOrthalicidae

(Breure, 1978)

Figs 61A–C , 67

Thaumastus (Scholvienia) gittenbergerorum Breure 1978: 44, fig. 57, pl. 6 figs 1–4; Ramírez et al. 2003: 282.
Thaumastus gittenbergerorum ; Richardson 1995: 376 (references).

#### Type locality.

“Peru, Dept. Huánuco, 10.8 km W Huancapallac, 2950 m”.

#### Type material.

UF 22119, holotype.

#### Additional material.

RMNH 55191 (3), RMNH 55189 (4), RMNH 55190 (3), UF 22119a (3), 22119b (3), 22751a (3), 22751b (5), 22751c(3), 22753 (16), paratypes.

#### Diagnosis.

Shell tawny coloured, on the last whorl indistinct, darker coloured spiral bands are present, teleoconch sculptured with incrassate growth striae, thickened at irregular distances forming peculiar whitish longitudinal stripes, partly fading away at lower side of whorl, and crossed by shallow spiral lines.

#### Dimensions.

Shell height 41.0, diameter 18.0 mm.

#### Distribution.

**Peru**, Dept. Amazonas, 21 km ENE Balsas; Dept. Huánuco, west of Huancapallac; ibid., 9.2 km S of Tingo Maria.

#### Ecoregion.

Peruvian Yungas [NT0153].

### Scholvienia huancabambensis

Taxon classificationAnimaliaStylommatophoraOrthalicidae

Strebel, 1910

Figs 65H–I , 67

Scholvienia huancabambensis Strebel 1910: 26, pl. 2 figs 15, 19a.
Thaumastus huancabambensis ; Richardson 1995: 376 (references).
Thaumastus (Scholvienia) huancabambensis ; Ramírez et al. 2003: 282.

#### Type locality.

“Huancabamba, Peru”.

#### Type material.

Not located.

#### Diagnosis.

Shell dark brown with a small, yellowish subsutural band, aperture with a dark brown band behind the lip

#### Dimensions.

Shell height 58.4, diameter 26.5 mm.

#### Distribution.

**Peru**, Dept. Pasco, Huancabamba.

#### Ecoregion.

Peruvian Yungas [NT0153].

#### Remarks.

Strebel described his species on the basis of material supplied by Rolle. There are several places with the name Huancabamba throughout Peru, but Rolle supplied more often material from the Chanchamayo region. Therefore it is assumed this material originated from (Tingo de) Huanacabamba in Dept. Pasco, which is at ca. 1870 m altitude in the Chanchamayo region. Strebel (1910: 26) remarked that, when the shells were held against bright backlight, one sees one, or more often two, spiral bands that are lighter than the groundcolour. This hints at a possible close relationship of this taxon with Scholvienia bifasciata (Philippi, 1845) or *Scholvienia iserni* (Philippi, 1867). The spiral banding visible in Strebel’s original figure (Fig. 65I) may be due to a growth anomaly.

### Scholvienia iserni

Taxon classificationAnimaliaStylommatophoraOrthalicidae

(Philippi, 1867)

Figs 65A–B , 67

Bulimus iserni Philippi 1867: 75; Pfeiffer 1867 [1866–1869]: 338, pl. 80 figs 16–18.
Thaumastus iserni ; Richardson 1995: 377 (references).
Thaumastus (Scholvienia) iserni ; Ramírez et al. 2003: 282.

#### Type locality.

[Peru] “prope La Oroya”.

#### Type material.

Not located.

#### Diagnosis.

Shell slender (height/diameter ratio 2.4), dark brown with two yellowish spiral bands, one subsutural, the other around the umbilical area on the last whorl.

#### Dimensions.

Shell height 53.0, diameter 22.0 mm.

#### Distribution.

**Peru**, Dept. Junín, near La Oroya.

#### Ecoregion.

Peruvian Yungas [NT0153].

#### Remarks.

The species in the La Oroya–Chanchamayo region need further studies to untangle distributions and relationships.

### Scholvienia jaspidea

Taxon classificationAnimaliaStylommatophoraOrthalicidae

(Morelet, 1863)

Figs 62D , 63A–C , 66

Bulimus jaspideus Morelet 1863: 180, pl. 7 fig. 7.
Thaumastus jaspideus ; Richardson 1995: 377 (references; excl. synonymy).
Thaumastus (Scholvienia) jaspideus ; Ramírez et al. 2003: 282.

#### Type locality.

[Peru] “de la vallée tempérée de Yucaï”, and “sur les murs des jardins, aux environs de Huancabelica”.

#### Type material.

MHNG-INVE-60211 (2), syntypes; MHNG-INVE-60210 (4), syntypes.

#### Diagnosis.

Shell with hardly convex sides, tawny coloured with some darker patches, sculptured with incrassate growth striae, especially on the last whorl thickened at irregular distances, crossed by spiral lines resulting in oblong granulation, aperture with columellar margin well dilated above.

#### Dimensions.

Shell height 47.2, diameter 21.4 mm.

#### Distribution.

**Peru**, Dept. Huancavelica.

#### Ecoregion.

Peruvian Yungas [NT0153].

### Scholvienia jelskii

Taxon classificationAnimaliaStylommatophoraOrthalicidae

(Lubomirski, 1880)

Figs 62E–F , 66

Bulimus (Orphnus) jelskii Lubomirski 1880: 722, pl. 56 figs 1–2.
Thaumastus jelskii ; Richardson 1995: 378 (references).
Thaumastus (Scholvienia) jelskii ; Ramírez et al. 2003: 282.

#### Type locality.

[Peru] “Amable Maria, près de Tarma”.

#### Type material.

MZIW, holotype.

#### Diagnosis.

Shell with straight sides, reddish-brown coloured with three spiral band, two small (subsutural, peripheral) and one broader (around the umbilical area on the lower part of last whorl).

#### Dimensions.

Shell height 35.0, diameter 15.0 mm.

#### Distribution.

**Peru**, Dept. Junín, San Ramón.

#### Ecoregion.

Peruvian Yungas [NT0153].

#### Remarks.

The species in the La Oroya–Chanchamayo region need further studies to untangle distributions and relationships.

### Scholvienia porphyria

Taxon classificationAnimaliaStylommatophoraOrthalicidae

(Pfeiffer, 1847)

Figs 62A–C , 67

Bulimus porphyrius Pfeiffer 1847: 114; Reeve 1848 [1848–1850]: pl. 15 fig. 89; Breure and Ablett 2015: 46, figs 11i–iv, L16iii.
Thaumastus porphyrius ; Richardson 1995: 380 (references).
Thaumastus (Scholvienia) porphyrius ; Ramírez et al. 2003: 282; Köhler 2007: 129, fig. 15.

#### Type locality.

“Bolivia”.

#### Type material.

NHMUK 1975277, lectotype (Breure 1978: 46).

#### Additional material.

NHMUK 1975278 (2), ZMB 112727 (2), paralectotypes.

#### Diagnosis.

Shell brownish coloured, on the last whorl a small, lighter coloured peripheral band is present, teleoconch sculptured with incrassate growth striae, thickened at irregular distances forming peculiar whitish longitudinal stripes, partly fading away at lower side of whorl, and crossed by shallow spiral lines.

#### Dimensions.

Shell height 51.5, diameter 22.0 mm.

#### Distribution.

**Peru**, Dept. Apurimac, Andahuaylas (Morelet 1863); ibid., Abancay; Prov. Ayacucho, Ccarapa (Breure 1978). ?**Bolivia**.

#### Ecoregion.

Peruvian Yungas [NT0153].

#### Remarks.

Breure (1978: 46) has argued why the type locality might be based on a labelling error. Morelet (1863: 173) attributed juvenile specimens from Andahuaylas to this species. This species has not been recognised in material from southern Peru; the presumed occurrence in Bolivia remains problematic as we have not seen any trusted material from that country that could be assigned to this species.

### Scholvienia weyrauchi

Taxon classificationAnimaliaStylommatophoraOrthalicidae

(Pilsbry, 1944)

Figs 64E–G , 66

Thaumastus (Scholvienia) weyrauch[i] Pilsbry 1944b: 121, pl. 11 fig. (emendation by Parodiz 1957: 134); Ramírez et al. 2003: 282.
Thaumastus weyrauchi ; Richardson 1995: 385 (references).

#### Type locality.

“Carpapata on the Rio Tarma, near Palca, Peru, at 2300 meters”.

#### Type material.

Holotype ANSP 179992.

#### Diagnosis.

Shell with straight sides, reddish-brown coloured with three narrow spiral band, one subsutural and two slightly broader (above and below the periphery of last whorl).

#### Dimensions.

Shell height 39.5, diameter 15 mm.

#### Distribution.

**Peru**, Dept. Junín, Carpapata.

#### Ecoregion.

Peruvian Yungas [NT0153].

#### Remarks.

Pilsbry (1944) also mentioned a paratype, with larger dimensions (shell height 46.5, diameter 16 mm).

### Genus Sultana

Taxon classificationAnimaliaStylommatophoraOrthalicidae

Shuttleworth, 1856

#### Type species.

*Helix sultana* Dillwyn, 1817, by tautonomy.

#### Description.

Shell (elongate-)ovate, imperforate, thin to solid, shell height up to ca. 60–90 mm (study area), colour pattern generally with <<-shaped spots or sinuous streaks, protoconch pitted or radially wrinkled, teleoconch with or without spiral elements, last whorl usually inflated.

#### Distribution.

?Panama, Colombia, Ecuador, Peru, Bolivia, Brazil, French Guiana, Suriname, Guyana.

#### Habitat.

Only partly known; mostly living in humid forests from 0–ca. 2000 m.

#### Anatomy.

Troschel 1849: Sultana (Sultana) sultana (Dillwyn, 1817) [r]; Strebel and Pfeffer 1882: Sultana (Metorthalicus) atramentaria (Pfeiffer, 1855) [g, m, r], Sultana (Sultana) sultana [g].

#### Remarks.

We follow herein the classification of Schileyko (1999), awaiting further morphological and molecular studies to ascertain the systematic position of this genus. The record for Panama is based on unverified museum collections. Contrary to Schileyko (1999: 362) we are not aware of any sinistral Sultana species.

### Subgenus Sultana (Metorthalicus)

Taxon classificationAnimaliaStylommatophoraOrthalicidae

Pilsbry, 1899

Orthalicus (Metorthalicus) Pilsbry 1899: 187.
Sultana (Trachyorthalicus) Strebel 1909: 151 (**syn. n.**).

#### Type species.

*Bulimus yatesi* Pfeiffer, 1855, by original designation.

#### Diagnosis.

Shell conic-ovate, solid, yellowish coloured with a pattern of brown, sinuous streaks, protoconch sculptured with axial riblets, becoming more zigzag on the last part, suture sharply ascending in front, aperture ovate, peristome thickened, columellar margin with a (indistinct) fold entering the aperture.

#### Distribution.

Colombia, Ecuador, Peru.

#### Habitat.

Not known.

#### Remarks.

The main distinction between Sultana (Metorthalicus) and Sultana (Trachyorthalicus)—type species *Bulimus fraseri* Pfeiffer, 1858, by original designation (Strebel 1909: 103)—is a slight difference in the protoconch scultpture, which in the latter subgenus consists of “schräge sich kreuzenden Reihen von Grübchen” (Strebel 1909: 151). The two subgenera are here synonymized after examination of the protoconch sculpture in the type specimens of the two type species; this sculpture proved to be nearly identical.

Most species in this group are represented in museum collections by a low number of specimens, which hampers an in-depth study of their variation. Also the lack of anatomical and phylogenetical data is currently a bottle-neck to fully understand their systematic position.

### Sultana (Metorthalicus) atramentaria

Taxon classificationAnimaliaStylommatophoraOrthalicidae

(Pfeiffer, 1855)

Figs 71A–C , 80

Bulimus atramentaria Pfeiffer 1855: 116.
Orthalicus iodes Shuttleworth 1856: 68, pl. 4 fig. 8; Neubert and Gosteli 2003: 30, pl. 6 fig. 2.
Bulimus boussingaultii Hupé 1857: 37, pl. 9 fig. 2.
Sultana atramentaria ; Richardson 1993: 121 (references, synonymy); Ramírez et al. 2003: 282.

#### Type locality.

“New Grenada”.

#### Type material.

Not located.

#### Additional material.

NMBE 19045 (3), syntypes of *Orthalicus iodes* Shuttleworth.

#### Diagnosis.

Shell light coloured with brownish sinuous streaks, which merge on the last whorl, aperture with a dark brown band inside behind the lip, columellar magin and parietal callus also dark brown.

#### Dimensions.

Shell height 81, diameter 35 mm.

#### Distribution.

Colombia (Shuttleworth 1856). **Ecuador**, Prov. Pastaza, Canelos (Martens 1885: 156). **Peru**, Dept. San Martin, Yuracyacu (NHMUK 1928.12.6.15).

#### Ecoregion.

Napo moist forests [NT0142], Peruvian Yungas [NT0153].

#### Remarks.

Pfeiffer based his description on material from the Cuming collection; Shuttleworth described his material, which he received from Cuming, from “in Andibus Columbiae”. At the time of collection of the material, both type localities extended beyond the present-day administrative boundaries of Colombia. The Ecuadorian record is on authority of Martens, and needs confirmation of the material to be conspecific with the Colombian specimens. We found a lot in NHMUK corresponding to this species, with a precise locality in Peru.

### Sultana (Metorthalicus) augusti

Taxon classificationAnimaliaStylommatophoraOrthalicidae

(Jousseaume, 1887)

Figs 71D–E , 80

Porphyrobaphe augusti Jousseaume 1887: 165, pl. 3 fig. 10.
Sultana augusti ; Richardson 1993: 122 (references).
Sultana (Metorthalicus) augusti ; Breure and Borrero 2008: 25.

#### Type locality.

[Ecuador] “l’Équateur”.

#### Type material.

MNHN 28014, holotype.

#### Diagnosis.

Shell yellowish, upper whorls with irregular pattern of brown, sinuous streaks, on last whorls streaks narrow and fading away, aperture flaring, peristome white, columellar margin vertical.

#### Dimensions.

Shell height 68.4, diameter 38.4 mm.

#### Distribution.

**Ecuador**, Prov. Azuay, Quebrada Machai (Pilsbry, 1899: 195); ?Prov. El Oro, Mirador (RBINS); Prov. Pastaza, Mera; ibid., Puyo; Prov. Tungurahua, Topo (Breure and Borrero 2008).

#### Ecoregion.

Eastern Cordillera real montane forests [NT0121].

#### Remarks.

As already noticed by Ancey (1890), his *Porphyrobaphe galactostoma* is evidently allied to the species (see also *Sultana yatesi* below). The specimen in RBINS is from “Mirador, Ecuador” without further indication of the Province; it is here tentatively assigned to El Oro.

### Sultana (Metorthalicus) deburghiae

Taxon classificationAnimaliaStylommatophoraOrthalicidae

(Reeve, 1859)

Figs 55C , 68A–D , 80

Bulimus deburghiae Reeve 1859: 123; Breure and Ablett 2015: 28, figs 18i–ii, L5iii.
Bulimus gloriosus Pfeiffer 1862: 387, pl. 37 fig. 4; Breure and Ablett 2015: 31, figs 18iii–iv, L7iv.
Porphyrobaphe gloriosus var. β elongatus Miller, 1878: 185; Miller 1879: pl. 5 fig. 1.
Sultana deburghiae ; Richardson 1993: 122 (references); Ramírez et al. 2003: 282; Breure and Borrero 2008: 25.

#### Type locality.

“Peruvian side of the Amazon”.

#### Type material.

NHMUK 19601622, lectotype (Breure and Schouten 1985: 27).

#### Additional material.

NHMUK 1975243, lectotype of *Bulimus gloriosus* Pfeiffer (Breure and Schouten 1985: 27).

#### Diagnosis.

Shell with broad dark stripes separated by light coloured, narrow zigzag stripes, a wide umbilical zone paler, two narrow dark bands at the periphery and around the umbilical area, both interrupted by light spots.

#### Dimensions.

Shell height 64.7, diameter 33.6 mm.

#### Distribution.

**Ecuador**, Prov. Napo, 6.5 km SSE Baeza; ibid., Nachiyacu; Prov. Pastaza, Cerros de Abitagua; ibid., Mera; ibid., Porvenir; Prov. Pichincha, Nanegal; Prov. Tungurahua, Topo; ibid., Baños; ibid., Rio Negro (all Breure and Borrero 2008); ?Prov. El Oro, Mirador (RBINS). **Peru** (?, see remarks).

#### Ecoregion.

Eastern Cordillera real montane forests [NT0121], Napo moist forests [NT0142], Northwestern Andean montane forests [NT0145].

#### Remarks.

Pfeiffer’s taxon was described from Ecuador, without specific locality. The record for Peru seems to be only based on Reeve’s locality and needs further confirmation.

### Sultana (Metorthalicus) fraseri

Taxon classificationAnimaliaStylommatophoraOrthalicidae

(Pfeiffer, 1858)

Figs 72A–B , 76A–B , 80

Bulimus fraseri Pfeiffer 1858: 239; Pfeiffer 1860: 137, pl. 51 fig. 5; Breure and Ablett 2015: 31, figs 19i–ii, L7iii.
Orthalicus fraseri brevispira Pilsbry 1899: 194, pl. 46 figs 34–35.
Sultana fraseri ; Richardson 1993: 123 (references, synonymy).
Sultana (Trachyorthalicus) fraseri ; Breure and Borrero 2008: 26.

#### Type locality.

“in provincia Cuenca reipublicae Aequatoris”.

#### Type material.

NHMUK 20140083, lectotype (Breure and Schouten 1985: 28).

#### Additional material.

ANSP 78573, holotype of *Orthalicus fraseri brevispira* Pilsbry.

#### Diagnosis.

Shell with yellowish ground colour and very narrow, interrupted, longitudinal dark brown stripes, especially on last whorl, and up to five spiral bands crossed by sinuous markings, peristome white, parietal callus and upper part of columellar margin lilac-whitish.

#### Dimensions.

Shell height 88.9, diameter 45.0 mm.

#### Distribution.

**Ecuador**, Prov. Azuay, near Cuenca; Prov. Loja (Strebel 1909: 154, as forma *brevispira*; no specific locality mentioned); ibid., Malacatos (USNM 316083); Prov. Morona-Santiago, Gualaquiza (Breure and Borrero 2008).

#### Ecoregion.

Eastern Cordillera real montane forests [NT0121].

#### Remarks.

Evidently related to *Sultana augusti* (Jousseaume, 1887) and *Sultana yatesi* (Pfeiffer, 1855).

### Sultana (Metorthalicus) kellettii

Taxon classificationAnimaliaStylommatophoraOrthalicidae

(Reeve, 1850)

Figs 73A , 79A–B , 80

Bulimus kellettii Reeve 1850 [1848–1850]: pl. 89 fig. 661; Breure and Ablett 2015: 37, figs 19iii–iv, L11ii.
Bulimus jatesi ‘Shuttleworth’ Hupé 1857: 31, pl. 8 figs 1–1a.
Bulimus fungairinoi Hidalgo 1867: 72, pl. 4 fig. 4.
Sultana kellettii ; Richardson 1993: 123 (synonymy, references); Ramírez et al. 2003: 282; Breure and Borrero 2008: 26.

#### Type locality.

“Ecuador?”.

#### Type material.

NHMUK 1975241, lectotype (Breure and Schouten 1985: 28).

#### Additional material.

MNCN 15.05/3159 (2), syntypes of *Bulimus fungairinoi* Hidalgo.

#### Diagnosis.

Shell tawny coloured, the upper whorls paler, last whorl with three spiral bands of dark brown, interrupted by sinuous streaks, aperture whitish inside with a darker band behind lip, parietal callus dark.

#### Dimensions.

Shell height 61.2, diameter 33.2 mm.

#### Distribution.

**Ecuador**, Prov. Azuay, Cuenca; ibid., Nabón (Strebel 1909: 160); Prov. Loja, Malacatos (Strebel 1909: 159); Prov. Pastaza, Mera; ibid., Cerros de Abitagua; Prov. Tungurahua, Rio Negro (all Breure and Borrero 2008). **Peru** (?, see remarks).

#### Ecoregion.

Eastern Cordillera real montane forests [NT0121].

#### Remarks.

The Peruvian record is based on Hupé (1857: 32, “le Pérou”). Dohrn (1882: 112–114) and Pilsbry (1899: 204–205) have discussed the relationship between the different forms of this species, which is only known with certainty from Ecuador.

### Sultana (Metorthalicus) labeo

Taxon classificationAnimaliaStylommatophoraOrthalicidae

(Broderip, 1828)

Figs 77A–B , 78A–D , 80

Bulinus labeo Broderip 1828: 222, suppl. pl. 31.
Sultana labeo ; Richardson 1993: 124 (references); Ramírez et al. 2003: 282.

#### Type locality.

“sylvis Peruvianis”.

#### Type material.

Not located (see Pain 1959).

#### Diagnosis.

Shell light to dark brown, with three (indistinct) spiral bands on the last whorl, interrupted by a few, oblique, light-coloured streaks, apex blunt, aperture with a calloused peristome, flesh- to dark-brown coloured on the front side, dirty whitish on the dorsal side.

#### Dimensions.

Shell height 76.2, diameter 44.5 mm.

#### Distribution.

**Peru**, Amazonas, east of Chachapoyas.

#### Ecoregion.

Peruvian Yungas [NT0153].

#### Remarks.

Broderip (1828: 223) mentioned in his text that the type material originated from Lieut. Maw, who obtained it at “Toulea, about nine leagues to the eastward of Chachapoyas”; this is evidently the locality presently known as Taulia [-06.1200 S, -077.3700 W].

### Sultana (Metorthalicus) macandrewi

Taxon classificationAnimaliaStylommatophoraOrthalicidae

Sowerby III, 1889 comb. n.

Figs 77C

Orthalicus macandrewi Sowerby III 1889: 398, pl. 25 fig. 18; Richardson 1993: 104 (references); Ramírez et al. 2003: 282.

#### Type locality.

“Santiago de Cou, Peru”.

#### Type material.

Not located.

#### Diagnosis.

Shell coloured with a grayish-fulvous zone below the suture, and two light brown zones at the periphery and on the lower part of last whorl, aperture lilac within, peristome black-edged (after Sowerby III 1889).

#### Dimensions.

Shell height 70, diameter 30 mm.

#### Distribution.

**Peru**, Dept. La Libertad, Prov. Santiago de Chuco (?, see remarks).

#### Remarks.

The type locality mentioned by Sowerby could not been found in modern gazetteers. Since he was a shell dealer and obtained his specimens through third persons, there might have been a mistake in labeling. In case this assumption is correct, the province of Santiago de Chuco might have been meant; this province is located west of and adjacent to the Marañon river. This species was regarded so far as *Orthalicus macandrewi*, but is unlike other *Orthalicus* species in shell shape and colouration. It is now tentatively placed in Sultana (Metorthalicus), but future studies are needed to confirm this.

### Sultana (Metorthalicus) maranhonensis

Taxon classificationAnimaliaStylommatophoraOrthalicidae

(Albers, 1854)

Fig. 73C–E

Bulimus maranhonensis Albers 1854: 216; Breure 2013: 31, fig 28C–E, 28ii.
Sultana maranhonensis ; Richardson 1993: 124 (references); Ramírez et al. 2003: 282.

#### Type locality.

“in Columbia ad fluvium Maranhon”.

#### Type material.

ZMB 101825, lectotype (Breure 2013: 31).

#### Additional material.

ZMB 111927 (1), paralectotype.

#### Diagnosis.

Shell tawny, with livid clouds and irregular blackish streaks and spots, height of aperture less than half the shell height.

#### Dimensions.

Shell height 75.6, diameter 36.8 mm.

#### Distribution.

**Peru**, ?Dept. Loreto; see remarks.

#### Remarks.

The río Marañon nowadays runs totally through Peruvian territory, although at the time the material was collected by Warszewicz some areas were part of Colombia.

### Sultana (Metorthalicus) shuttleworthi

Taxon classificationAnimaliaStylommatophoraOrthalicidae

(Albers, 1854)

Figs 75A–C

Bulimus shuttleworthi Albers 1854: 216; Breure 2013: 45, figs 29A–B, 29i.
Sultana shuttleworthi ; Richardson 1993: 125 (references); Ramírez et al. 2003: 283.

#### Type locality.

“in Columbia ad fluvium Maranhon”.

#### Type material.

ZMB 101827 (2), syntypes.

#### Diagnosis.

Shell with broad, irregular, dark brown streaks, height of aperture less than half the shell height.

#### Dimensions.

Shell height 70.5, diameter 34.4 mm.

#### Distribution.

**Peru**, ?Dept. Loreto; see remarks.

#### Remarks.

See also above under *maranhonensis* (Albers).

### Sultana (Metorthalicus) vicaria

Taxon classificationAnimaliaStylommatophoraOrthalicidae

(Fulton, 1896)

Fig. 69A–C

Porphyrobaphe vicaria Fulton 1896: 103; Breure and Ablett 2015: 51, figs 20i–ii, L18i.
Sultana yatesi (Pfeiffer); Richardson 1993: 128 (references), see remarks.
Sultana yatesi vicaria ; Ramírez et al. 2003: 283.

#### Type locality.

“Leimabamba, Peru, 8000 feet”.

#### Type material.

NHMUK 20100507, holotype.

#### Diagnosis.

Shell ovate-conical, with a uniform colour pattern of faint, narrow, longitudinal, reddish-brown stripes on a yellow-whitish ground colour, peristome pinkish, parietal callus dark brown.

#### Dimensions.

Shell height 82.2, diameter 46.7 mm.

#### Distribution.

Peru, Dept. Amazonas, Leimebamba.

#### Ecoregion.

Peruvian Yungas [NT0153].

#### Remarks.

This taxon has been synonymized with Sultana (Metorthalicus) yatesi (Pfeiffer, 1855) by Richardson (1993) without further comments. Breure and Ablett (2015) followed this opinion, but doubt remained. In the context of this study we prefer to treat Fulton’s taxon tentatively as a separate species as it seems to differ by the stouter shell, the more inflated last whorl, and the uniform colour pattern.

### Sultana (Metorthalicus) wrzesniowskii

Taxon classificationAnimaliaStylommatophoraOrthalicidae

(Lubomirski, 1880)

Figs 75D–F , 80

Bulimus (Porphyrobaphe) wrzesniowskii Lubomirski 1880: 721, pl. 55 figs 7–8.
Sultana wrzesniowskii ; Richardson 1993: 127 (references); Ramírez et al. 2003: 283.

#### Type locality.

[Peru] “Tambillo”.

#### Type material.

MIZW, holotype.

#### Diagnosis.

Shell flesh-coloured with longitudinal, narrow brownish streaks and some irregularly spaced spots, height of aperture more than half the shell height.

#### Dimensions.

Shell height 78.0, diameter 37.0 mm.

#### Distribution.

**Peru**, Dept. Ayacucho, [Prov. Huamanga, Distr.] Tambillo (MZIW).

#### Ecoregion.

Peruvian Yungas [NT0153].

#### Remarks.

The type material was located in the Lubomirski collection (D. Mierzwa-Szymkowiak, pers. commun., 2012). This species has not been re-collected after its description.

### Sultana (Metorthalicus) yatesi yatesi

Taxon classificationAnimaliaStylommatophoraOrthalicidae

(Pfeiffer, 1855)

Figs 69C–D , 70A–C , 80

Bulimus labeo Reeve 1848 [1848–1850]: pl. 71 fig. 207b, pl. 72 fig. 207c. Not Bulimus labeo Broderip, 1828.
Bulimus yatesi Pfeiffer 1855: 93, pl. 31 fig. 5; Breure and Ablett 2015: 54, figs 20iii–iv, L19ii.
Porphyrobaphe latevittata Shuttleworth 1856: 71, pl. 5 figs 2–3; Neubert and Gosteli 2003: 32, pl. 6 fig. 1.
Porphyrobaphe sublabeo ‘Dohrn’ Ancey 1890: 153; Wood and Gallichan 2008: 86, pl. 10 figs 2, ii.
Porphyrobaphe grandis Rolle 1902: 211.
Porphyrobaphe sarcostoma Ancey 1903: 83; Wood and Gallichan 2008: 82, pl. 10 figs 1, i.
Sultana yatesi ; Richardson 1993: 127 (references, synonymy) [partial].
Sultana yatesi yatesi ; Ramírez et al. 2003: 283.

#### Type locality.

[Peru] “Meobamba”.

#### Type material.

NHMUK 1975239, lectotype (Breure and Schouten 1985).

#### Additional material.

NMBE 18965 (3), syntypes of *Porphyrobaphe latevittata* Shuttleworth; NMW 1955.158.24080 (1), syntype of *Porphyrobaphe sublabeo* Ancey; NMW 1955.158.24078 (1), syntype of *Porphyrobaphe sarcostoma* Ancey.

#### Diagnosis.

Shell elongate-ovate, ground colour varying from yellowish to reddish-brown and purplish, with more or less conspicuous longitudinal sinuous streaks and crossed by up to four spiral bands, peristome and parietal callus whitish or pinkish.

#### Dimensions.

Shell height 84.3, diameter 39.7 mm.

#### Distribution.

**Peru**, Dept. Amazonas, Leimebamba; ibid., Goncha [Asunción] (NHMUK 1928.12.6.14); ibid., Puca Tambo (NHMUK 1928.12.6.6–7); Dept. Junín, Chanchamayo [valley], 1000 m (NHMUK); Dept. San Martín, Moyobamba (NHMUK 1975239); ibid., Tarapoto (NMBE); ?, “Reipublica Peruvianae, regione Amazonica” (NMW, syntype of *Porphyrobaphe sublabeo*).

#### Ecoregion.

Peruvian Yungas [NT0153].

#### Remarks.

The taxon described by Rolle (1902) was figured by Strebel (1909: 168, pl. 33 fig. 476), but the specimen has not been located in the ZMB collection. No type locality has been supplied by Rolle, and Richardson (1993: 127) has synonymized this taxon with Pfeiffer's species.

### Sultana (Metorthalicus) yatesi galactostoma

Taxon classificationAnimaliaStylommatophoraOrthalicidae

(Ancey, 1890)

Fig. 70D

Porphyrobaphe galactostoma Ancey 1890: 153; Wood and Gallichan 2008: 46, pl. 10 figs 3, iii.
Sultana yatesi ; Richardson 1993: 127 (references).
Sultana yatesi galactostoma ; Ramírez et al. 2003: 283.

#### Type locality.

“Republica Aequatoris”.

#### Type material.

NMW 1955.158.24079 (1), syntype.

#### Diagnosis.

As nominate species, but colour whitish yellow.

#### Distribution.

**Ecuador**, without precise locality. **Peru**, without precise locality (?, see remarks).

#### Remarks.

This taxon is known by the type material only. According to Wood and Gallichan (2008: 46), the subsequent record from Peru by Ancey (1903: 89) was a mistake; there is no verified material to prove this record.

### Subgenus Sultana (Sultana)

Taxon classificationAnimaliaStylommatophoraOrthalicidae

Shuttleworth, 1856

#### Habitat.

May be found on leaves and trunks of trees, especially after rains, buried at base of trees during dry season (Gargominy in Massemin et al. 2009). All localities known are from low altitudes (< 500 m).

### Sultana (Sultana) meobambensis

Taxon classificationAnimaliaStylommatophoraOrthalicidae

(Pfeiffer, 1855) comb. n.

Figs 73B , 74A–B , 81 , 90A–C

Bulimus meobambensis Pfeiffer 1855: 96; Breure and Ablett 2015: 41, figs 17iii–iv, L13i.
Orthalicus meobambensis carnea Strebel 1909: 149, pl. 19 fig. 428; Breure 2013: 15, figs 28A–B, 28i.
Sultana meobambensis ; Ramírez et al. 2003: 282.
Sultana meobambensis carnea ; Ramírez et al. 2003: 282.
Sultana sultana (Dillwyn); Richardson 1993: 127 (references, synonymy) [partial].

#### Type locality.

“Meobamba, Eastern Peru”.

#### Type material.

NHMUK 20100505 (2), syntypes.

#### Additional material.

ZMB 101823, holotype of *Orthalicus meobambensis carnea* Strebel.

#### Dimensions.

Shell height 84.9, diameter 52.8 mm.

#### Distribution.

**Peru**, San Martín, Moyobamba.

#### Ecoregion.

Peruvian Yungas [NT0153].

#### Remarks.

Cuming’s material might have originated in the Province of Moyobamba rather than near the locality of the same name. This taxon was regarded by Richardson (1993) as a junior subjective synonym of *Sultana sultana* Dillwyn, 1817, possibly reflecting the opinions of Parodiz (1962: 456), who wrote “I am inclined to think that *Orthalicus meobambensis* Pfeiffer is a synonym”, and of Pilsbry (1899: 191) stating “from the description (…) I would think *meobambensis* identical with the upper Amazonian variety of *Orthalicus sultana*”. We agree that both species are closely related, but refrain from this conclusion until the variation of both taxa is better documented.

### Sultana (Sultana) sultana

Taxon classificationAnimaliaStylommatophoraOrthalicidae

(Dillwyn, 1817)

Figs 76C , 81

Helix sultana Dillwyn 1817: 920.
Orthalicus trullisatus Shuttleworth 1856: 58, pl. 5 fig. 1; Neubert and Gosteli 2003: 54, pl. 6 fig. 4.
Orthalicus sultana angustior Preston 1914: 524.
Sultana sultana ; Richardson 1993: 125 (references, synonymy); Ramírez et al. 2003: 283; Simone 2006: 158, fig. 541; Massamin et al. 2009: 410, pl. 5A; Linares and Vera 2012: 160.
Sultana sultana angustior ; Ramírez et al. 2003: 283.

#### Type locality.

“New Zealand” [sic].

#### Type material.

Not located.

#### Additional material.

NMBE 18962 (2), syntypes of *Orthalicus trullisatus* Shuttleworth.

#### Dimensions.

Shell height 87.4, diameter 54.5 mm.

#### Distribution.

Panama (ANSP). Colombia (Linares and Vera 2012). **Ecuador**, Prov. Los Rios, Palenque Science Center (UF 181509); Prov. Morona-Santiago, 59 km SSE Patuca (UF 139123); Prov. Napo, Nachiyacu (ANSP 170700); Prov. Orellana, Loreto (ANSP 195216); ibid., Tiputini (RBINS); Prov. Tungurahua, Topo (ANSP 306772). **Peru**, Dept. Amazonas, San Antonio (UF 24931); ibid., Caterpiza (UF 28050); ibid., Galilea (UF 28048); ibid., Huampami (UF 24932); Dept. Huánuco, near Tingo María (UF 21275); Dept. Loreto, Orellana (USNM 601366); Dept. Madre de Dios, ca. 30 km SSW Puerto Maldonado (UF 26618); Dept. San Martín, Tarapoto (see remarks). **Bolivia**, Dept. Beni, Rurrenabaque (USNM 361144); Dept. La Paz, Chiñiri (ANSP 165196); Dept. Santa Cruz, Todos Santos (ANSP 170682). Brazil (Simone 2006). French Guiana (Massemin et al. 2009). Suriname (Altena 1975).

#### Ecoregion.

Bolivian Yungas [NT0105], Eastern Cordillera real montane forests [NT0121], Iquitos varzea [NT0128], Napo moist forests [NT0142], Northwestern Andean montane forests [NT0145], Peruvian Yungas [NT0153], Southwest Amazon moist forests [NT0166].

#### Remarks.

This easily recognizable species has been reported from widely disjunct areas on the entire continent, but always from low altitude moist forests. Shuttleworth described his taxon from “ab oriente Andium prope Tarapoto”, thus Peru. The dimensions given above are after Neubert and Gosteli 2003: 54. Richardson (1993: 127) arranged Shuttleworth’s taxon under *Sultana meobambensis* Pfeiffer, 1855. Preston’s taxon, which was described from “Eastern Peru”, is also arranged under this wide-spread species. Further morphological and molecular studies on its variation may clarify the systematic position of this taxon.

### Family Simpulopsidae

Taxon classificationAnimaliaStylommatophoraSimpulopsidae

Schileyko, 1999

Simpulopsidae Schileyko 1999: 324.

### Genus Simpulopsis

Taxon classificationAnimaliaStylommatophoraSimpulopsidae

Beck, 1837

Simpulopsis Beck 1837: 100.

#### Type species.

Helix (Cochlohydra) sulculosa Férussac, 1821, by subsequent designation (Martens in Albers 1860: 309).

#### Description.

Shell elongate-ovate to globose, rimate or imperforate, thin, protoconch with fine spiral lines that more or less cut the low, oblique riblets or wrinkles into granules, teleoconch smooth or corrugate, last whorl prominent, suture well impressed, aperture oblique to ovate, peristome thin and simple.

#### Distribution.

Mexico, Guatemala, West Indies, Colombia, Ecuador, Peru, Argentina, Paraguay, Brazil, French Guiana, Suriname, Venezuela.

#### Habitat.

Arboreal living in humid forests up to ca. 2500 m.

#### Anatomy.

Heynemann 1868: Simpulopsis (Simpulopsis) sulculosa (Férussac, 1821) [r]; Hylton Scott 1967: Simpulopsis (Eudioptus) citrinovitrea (S. Moricand, 1836) [m, r]; Araujo 1971: Simpulopsis (Eudioptus) citrinovitrea (S. Moricand, 1836) [g, m, p, r]; Araujo 1975a: Simpulopsis (Simpulopsis) ovata (Sowerby I, 1822) [g, m, p, r]; Breure 1975a: Simpulopsis (Simpulopsis) pseudosulculosa Breure, 1975 [g], Simpulopsis (Simpulopsis) sulculosa [g], Simpulopsis (Simpulopsis) wiebesi Breure, 1975 [g], Simpulopsis (Eudioptus) araujoi Breure, 1975 [g], Simpulopsis (Eudioptus) citrinovitrea (S. Moricand, 1836) [g]; Breure and Ploeger 1977: Simpulopsis (Simpulopsis) pseudosulculosa Breure, 1975 [r], Simpulopsis (Simpulopsis) wiebesi Breure, 1975 [r], Simpulopsis (Eudioptus) araujoi Breure, 1975 [r], Simpulopsis (Eudioptus) citrinovitrea (S. Moricand, 1836) [r]; Araujo and Breure 1977: Simpulopsis (Simpulopsis) miersi Pfeiffer, 1856 [g, h, p, r]; Tillier 1989: Simpulopsis (Simpulopsis) miersi Pfeiffer, 1856 [d, k, n, p]; da Silva and Thomé 2005: Simpulopsis (Eudioptus) citrinovitrea (S. Moricand, 1836) [g, m, p, r]; da Silva and Thomé 2006: Simpulopsis (Simpulopsis) gomesae da Silva and Thomé, 2006 [g, m, p, r], Simpulopsis (Simpulopsis) prometensis da Silva and Thomé, 2006 [g, m, p, r]; da Silva and Thomé 2007: Simpulopsis (Simpulopsis) decussata Pfeiffer, 1856 [g, m, p, r].

#### Phylogenetic data.

Breure and Romero 2012: *Simpulopsis decussata* Pfeiffer, 1856, *Simpulopsis rufovirens* (S. Moricand, 1846).

#### Remarks.

This genus is concentrated in eastern and southern Brazil, with one species—Simpulopsis (Eudioptus) citrinovitrea (S. Moricand, 1836)—extending in several Andean countries with a remarkable disjunct distribution. Cuezzo et al. (2013) also mentioned the occurrence in “Chile” for Simpulopsis (Eudioptus) eudioptus (Ihering in Pilsbry, 1897), however, without further evidence.

### Subgenus Simpulopsis (Eudioptus)

Taxon classificationAnimaliaStylommatophoraSimpulopsidae

Martens in Albers, 1860

Bulimulus (Eudioptus) Martens in Albers 1860: 223.

#### Type species.

Helix (Cochlogena) pseudosuccinea S. Moricand, 1836, by original designation.

#### Diagnosis.

Shell elongate-ovate, colour uniformly yellowish to brownish, protoconch with spiral lines and (indistinct) axial wrinkles, teleoconch surface smooth or with delicate spiral striae, aperture (sub- to elongate-)ovate.

#### Distribution.

Colombia, Ecuador, Peru, Argentina, Paraguay, Brazil.

### Simpulopsis (Eudioptus) citrinovitrea

Taxon classificationAnimaliaStylommatophoraSimpulopsidae

(S. Moricand, 1836)

Figs 79C–D , 82A–B , 83

Helix (Cochlogena) citrinovitrea S. Moricand 1836: 436, pl. 2 fig. 19; Neubert and Janssen, 2004: 205, pl. 17 fig. 208.
Bulimus fulguratus Miller 1878: 187; Miller 1879: pl. 6 fig. 6; Borrero and Breure 2011: 44 (synonymy).
Bulimulus (Paracochlea) willineri Hylton Scott 1967: 90.
Simpulopsis citrinovitrea ; Richardson 1995: 362 (references, partial synonymy).
Simpulopsis (Eudioptus) willineri ; Miquel 1998: 186.
Simpulopsis (Eudioptus) citrinovitrea ; Cuezzo et al. 2013: 184.

#### Type locality.

“[Brazil] aux environs de Bahia”.

#### Type material.

MHNG-INVE-64617 (19), syntypes.

#### Additional material.

MCZ 26194 (2), syntypes; MHNG-INVE 64616 (4), probable syntypes; SMF 302256 (2), syntypes.

#### Diagnosis.

Shell thin, uniformly yellowish, sculptured with spiral elements, aperture ovate, peristome thin and simple.

#### Dimensions.

Shell height 16.0, diameter 11.7 mm.

#### Distribution.

Colombia (Breure 1978). **Ecuador**, Prov. Pichincha, 59 km W Machachi (Breure 1978: 235). Argentina (Cuezzo et al. 2013). Paraguay (Hylton Scott 1967). Brazil (Simone 2006).

#### Ecoregion.

Northwestern Andean montane forests [NT0145].

#### Remarks.

This taxon has been reported from disjunct localities that are widely separate, at altitudes ranging ca. 700–1500 m. The external morphology is, however, very similar. Miquel (1998: 186) remarked that Simpulopsis (Eudioptus) willineri (Hylton Scott, 1967)—known from Paraguay and northern Argentina—strongly resembles this species. Cuezzo et al. (2013: 184) had it as synonym of Moricand’s taxon. The species might also be expected in suitable habitats in Bolivia. Breure (1978: 235) already pointed out that this disjunct distribution needs further investigation and molecular research may show either convergent evolution or a species complex.

### Nomen inquirendum

#### ‘Pseudoglandina’ agitata

Taxon classificationAnimaliaStylommatophoraSimpulopsidae

Weyrauch, 1967 stat. n.

Figs 82C , 83

Pseudoglandina agitata Weyrauch, 1967: 486, fig. 53; Neubert and Janssen 2004: 197, pl. 17 fig. 212; Barbosa et al. 2008: 268; Breure 2012a: 5.

##### Type locality.

“Perú central, vertiente oriental de la Cordillera Oriental, valle de Chanchamyo entre La Merced y San Ramón, 1100 m”.

##### Type material.

Holotype FML 1066.

##### Additional material.

FML 1066 (1), 10609 (1), paratypes; SMF 162138 (1), paratype.

##### Dimensions.

Shell height 16.8, diameter 10.4 mm.

##### Distribution.

**Peru**, Dept. Cajamarca, Miraflores; Dept. Junín, between La Merced and San Ramón; near Tarma, Pan de Azúcar (all Weyrauch 1967).

##### Ecoregion.

Peruvian Yungas [NT0153].

##### Remarks.

This species has been synonymized with Simpulopsis (Eudioptus) citrinovitrea by Breure (1978: 235). Upon re-study of the type specimens of Weyrauch’s taxon we notice that the holotype seems to be the most adult shell and is different from Moricand’s species. The paratypes in SMF (Fig. 82C) and FML are subadult and similar to Simpulopsis (Eudioptus) citrinovitrea. It is possible that this taxon may prove to be a related species, for which anatomical and molecular studies are needed. Weyrauch’s taxon is now re-surrected and considered as *nomen inquirendum* until further studies have clarified its systematic position.

### Doubtful and excluded taxa

#### Taxa doubtfully referred to the Ecuadorian malacofauna

*Bulimus maracaibensis* Pfeiffer 1856: 186; Breure and Borrero 2008: 26; Linares and Vera 2012: 152.

**Remarks.** The Ecuadorian record is based on material collected by Riess (Martens 1893 [1890–1901]: 184). This record needs further confirmation as they are well outside the main distribution range of the species. We have been unable to locate the material from this source.

*Orthalicus obductus* Shuttleworth 1856: 61, pl. 3 figs 1–3; Linares and Vera 2012: 154.

**Remarks.** This species was described from “Barquimeseto [sic, Barquisimeto] in Columbia [Venezuela]” (Neubert and Gosteli 2003: 40, pl. 6 fig. 3). The record for Ecuador is probably erroneous and seems to be copied from Mousson 1869: 179. This record was based on material from Wallis collected “in 8000' (!) [~ 2440 m] Höhe bei Nabon” (Prov. Azuay, Nabón). We have not located Mousson’s material, but assume a misidentification.

*Bulimus pulicarius* Reeve 1848 [1848–1850]: pl. 42 fig. 267; Breure and Borrero 2008: 6.

**Remarks.** This species has only been positively identified among Colombian material; see Borrero and Breure 2011: 46, figs 14B, 16G–M; Breure and Ablett 2011: 31, figs 22A–C, 22i.

Helix (Cochlitoma) regina Férussac 1821 [1821–1822]: 49 [nomen nudum]; Férussac 1823 in Férussac and Deshayes 1820–1851: pl. 119 figs 3–5; Breure and Borrero 2008: 27.

**Remarks.** Férussac did not mention a type locality for this species, which is generally considered to occur in northeastern South America. Gargominy in Massemin et al. (2009: 404) reported it from “Guyane et Brésil (?)”, and noticed that it has not been collected alive for more than a century in French Guiana. The lectotype, designated by Tillier (1980: 71, pl. 4 fig. 13), is MNHN 21881 (Figs 40E–G). Six specimens (MNHN 21883), labelled as “var. B com. Howe / Cayenne, haut de Raouza”, are labeled as paralectotypes; one specimen proved to be Orthalicus bensoni (Reeve, 1849). The presence of this species in Colombia (Linares and Vera 2012: 156) and Ecuador (Breure and Borrero 2008) are based on museum records which need further confirmation. See below for a record from Peru, and also the remarks above at the genus level.

#### Taxa doubtfully referred to the Peruvian malacofauna

*Bulimus maracaibensis* Pfeiffer 1856: 186; ; Linares and Vera 2012: 152.

**Remarks.** The Peruvian record from the imprecise locality “Río Marañon” is based on material collected by Warscewicz (Martens 1893 [1890–1901]: 185). This locality is very doubtful and may be based on misidentification, as this is a species with a coastal distribution. We have been unable to locate the material on which Martens based his record. We now consider *Zebra gruneri* Strebel, 1909 as a junior subjective synonym of Pfeiffer's species (**syn. n.**). See also Breure 2013a: 21.

*Zebra pilsbryi* Strebel 1909: 46, pl. 6 figs 85–86; Ramírez et al. 2003: 282 [species 664]; Linares and Vera 2012: 154.

**Remarks.** This record is based on specimens from Strebel’s collection in the Hamburg museum (now considered to be lost, B. Hausdorf pers. commun., 2008), from “Chonchomayo” [sic, Chanchamayo]. Strebel used this name also for shells from Costa Rica and Colombia; the Peruvian record remains very doubtful.

*Bulinus princeps* Broderip in Sowerby I and II, 1833 [1832–1841]: 6, fig. 18; Linares and Vera 2012: 155.

**Remarks.** This species is generally considered as Central American (Thompson 2011). It remains unclear on what evidence the Peruvian record is based of Linares and Vera (2012).

*Achatina pulchella* Spix in Wagner, 1827: pl. 9 fig. 2; Ramírez et al. 2003: 282 [species 664].

**Remarks.** The record of this Brazilian species (Simone 2006: 157, fig. 536) is likely based on the synonymy with *Zebra pilsbryi* Strebel, 1909 (see above).

Helix (Cochlitoma) regina Férussac 1821 [1821–1822]: 49 [nomen nudum]; Férussac 1823 in Férussac and Deshayes 1820–1851: pl. 119 figs 3–5; Ramírez et al. 2003: 282 [species 660].

**Remarks.** See above. The record from Peru is very doubtful as it remains unclear on what evidence it was based.

*Bulimus taeniolus* Nyst 1845: 151, pl. 2 fig. 4a, b; Ramírez et al. 2003: 282 [species 632].

**Remarks.** This species was described from “l’Amérique méridionale” and subsequent authors have never been able to pinpoint a more precise locality. Pilsbry (1895 [1895–1896]: 57) wrote “Compare Strophocheilus brephoides Orb.; *Strophocheilus spixii* Reeve; *Strophocheilus spixii* Wagner, etc.”. As the type material has not been located, this taxon is best treated as nomen inquirendum.

#### Taxa excluded from the Ecuadorian malacofauna

Thaumastus (Thaumastus) brunneus (Strebel 1910) (type locality: “Ecuador”; type material not located); considered a synonym of the Bolivian Thaumastus (Thaumastus) inca (d’Orbigny, 1835) by Pilsbry (1932: 391), who regarded Strebel’s locality data erroneous.

Hemibulimus (Hemibulimus) excisus (Martens, 1885) (type locality: “Columbiae (Novae Granadae) prope Popayan”; type material ZMB 101837). The Ecuadorian record is based on Strebel (1909: 108), who had one specimen from “Maccas”; this locality was tentatively attributed to Prov. Azuay by Breure and Borrero (2008). Re-studying of the figure of Strebel (1909: figs 361) has led us to the conclusion that this shell represents Clathrorthalicus magnificus (Pfeiffer, 1848) and Strebel’s record has to be regarded as misidentification.

#### Taxa excluded from the Peruvian malacofauna

The following taxa, arranged alphabetically by species name, are now excluded from the land snail fauna of Peru:

*Cyclodontina angulata* (Wagner, 1827) [Ramiréz et al. 2003: 282, species 658] (type locality “in sylvis ad Solimoès et Purù fluvios”; type material not located); no evidence found for Peruvian material. This Brazilian species is listed as *Moricandia angulata* in Simone (2006: 171).

*Plectostylus granulosus* (Broderip, 1832) [Ramírez et al. 2003: 281, species 555, with wrong authorship]; no evidence found for Peruvian records of this Chilean species which is regarded a junior subjective synonym of *Plectostylus chilensis* (Lesson, 1831) by Richardson (1995: 294).

Plekocheilus (Eurytus) manco Pilsbry, 1930 [Ramírez et al. 2003: 281, species 545] (type locality: “Peru”, ANSP 152287); considered to be a synonym of the Venezuelan species Plekocheilus (Aeropictus) veranyi (Pfeiffer, 1847) by Weyrauch, 1967b: 457).

*Orthalicus ponderosus* (Strebel in Strebel and Pfeffer, 1882) [Ramírez et al. 2003: 282, species 663] (no type locality given; type material not located); considered to be a Mexican taxon (Thompson 2011: 103).

*Orthalicus zebra* (Müller, 1774) [Ramírez et al. 2003: 282, species 665] (no type locality given; type material: ZMUC); evidence unclear on which the Peruvian record is based.

## Discussion

The frequent papers by Simone and collaborators on the relatively well-known malacofauna of Brazil, regularly describing new species (e.g. Simone 2012, Salvador and Cavallari 2014, Salvador and Simone 2014, Simone 2015), demonstrates that the Neotropical malacofauna is still insufficiently known. This is especially alarming in the light of the ongoing biodiversity loss due to habitat destruction which hampers conservation efforts. In this context the poor knowledge of the malacofauna of Bolivia as shown in this paper makes it relatively a ‘white spot’ in South America; this calls for an urgent prioritisation for field work being undertaken throughout this country, and subsequent taxonomical studies to bring our knowledge of this fauna up to date. The same applies, although this cannot be detailed in the present paper, for the malacofauna of Paraguay.

Although the data for Ecuador and Peru are seemingly more substantial, it may be noted that many taxa are still poorly known, with (very) limited verified distribution records, and their anatomical morphology unknown in most cases. This compilation of data on these families thus can only be seen as a necessarily incomplete attempt to give an overview of this malacofauna, but hopefully will also act as a stimulus for (local) malacologists to further our knowledge.

Given the limited verified distribution records presented in this paper, we refrain from an analysis of ecoregions and endemism comparable to Breure and Borrero (2008: 30–32). It is hoped that the data in this paper will stimulate curators to have their collections checked for additional distribution records, as well as Neotropical malacologists to gather new records, which together will allow a future analysis.

From the data presented it may be clear that the ecology of nearly all species listed in this paper is still hardly known or very poorly understood. Meyer III et al. (2013) have shown that terrestrial gastropods can play a major role in litter decomposition in tropical habitats and the presence of gastropods increased litter decomposition rates; the highest decomposition rates were those with the greatest gastropod biomass. Furthermore, although there were differences in the rates of release of some nutrients among treatments, the different gastropod species appeared to influence nutrient release in a similar way. Since all species listed herein are relatively of considerable size, their contribution to maintain the soil quality in their habitats may not be negligible and an additional argument for conservation measures. Insufficient data may prevent specific measures for species treated above, but it is already alarming to note that a substantial number of species have not been re-found after their original description in the 19th century or are hardly represented in collections made during the 20th century.

## Plates

**Figure 3.**
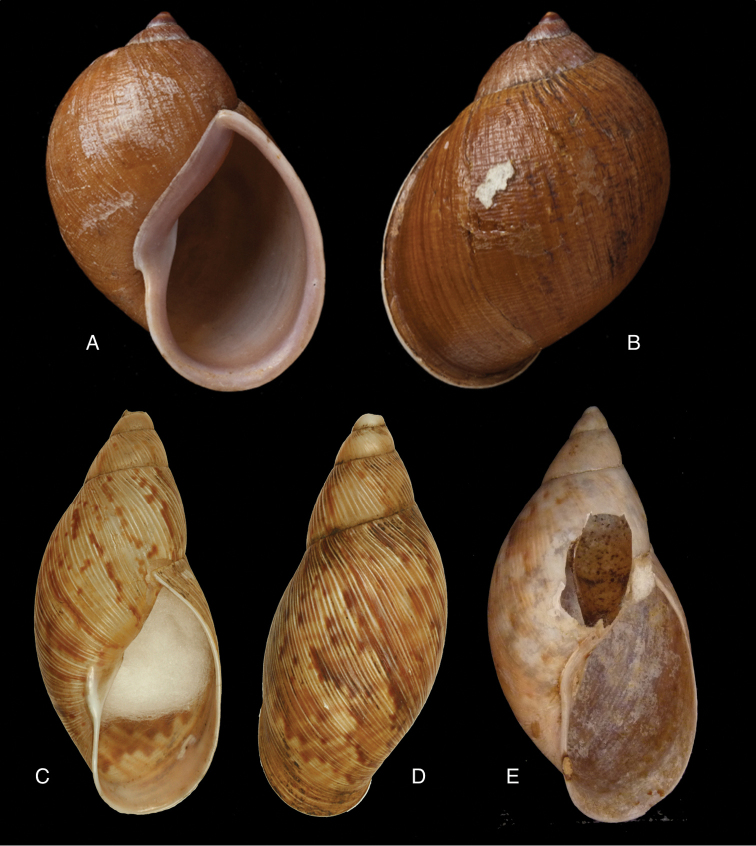
Plekocheilus species. **A–B** Plekocheilus (Eurytus) doliarius (Da Costa, 1898), lectotype NHMUK 1907.11.21.110 (H = 58.0) **C–E** Plekocheilus (Eurytus) floccosus (Spix in Wagner, 1827) **C–D** syntype ZSM 20020116 (H = 60.0) **E** holotype of *Helix pentadina* d’Orbigny MNHN 28258 (H = 61.1).

**Figure 4.**
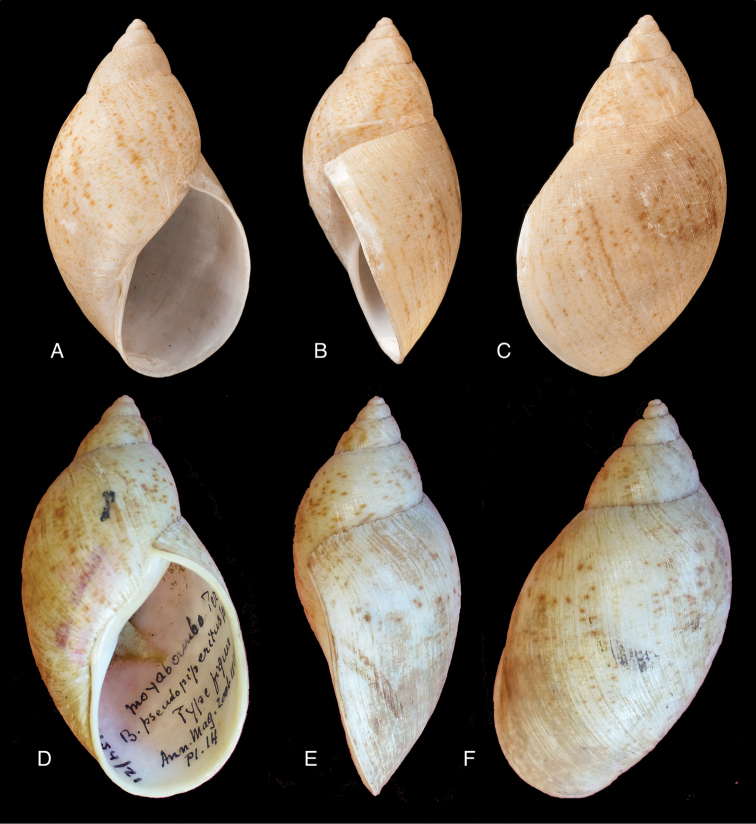
Plekocheilus species. **A–F** Plekocheilus (Eurytus) piperitus piperitus (Sowerby I, 1837) **A–C** syntype NHMUK 1975239 (H = 55.8) **D–F** syntype of *Bulimus pseudopiperatus* J. Moricand, 1858, MHNG-INVE-55493 (H = 59.1).

**Figure 5.**
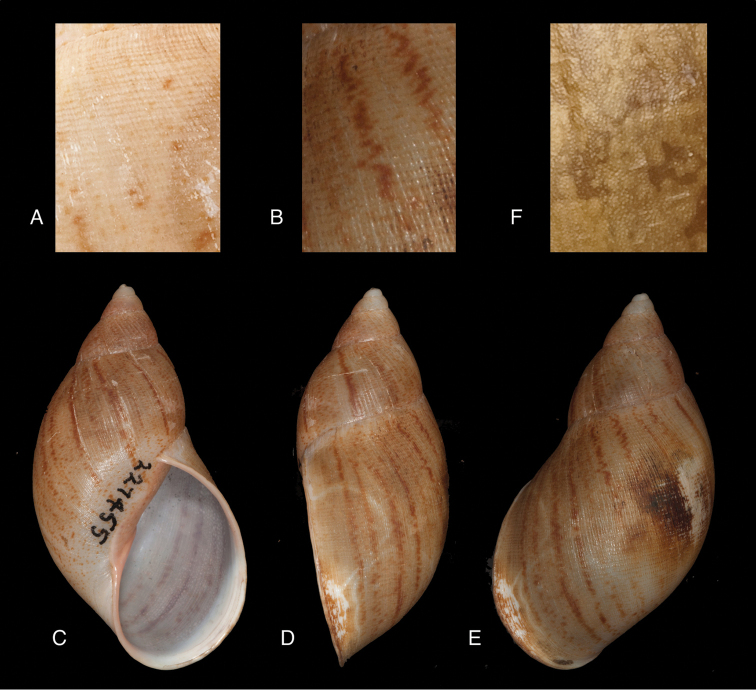
Plekocheilus species. **A** Plekocheilus (Eurytus) piperitus piperitus (Sowerby I, 1837), detail of sculpture on dorsal side of last whorl **B–E** Plekocheilus (Eurytus) piperitus mcgintyi ‘Pilsbry’ H.B. Baker, 1963; **B** detail of sculpture on dorsal side of last whorl **C–E** possible syntype ANSP 227455 (H = 56.8) **F** Plekocheilus (Eurytus) taylorianus (Reeve, 1849), detail of sculpture on dorsal side of last whorl.

**Figure 6.**
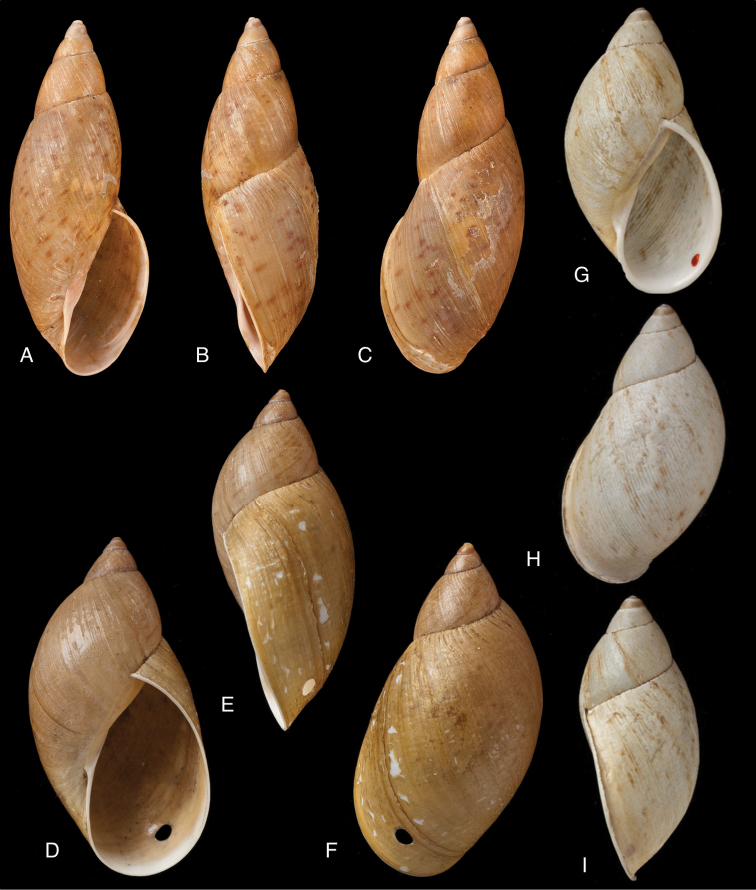
Plekocheilus species. **A–C** Plekocheilus (Eurytus) onca (d’Orbigny, 1835), lectotype NHMUK 1854.12.4.120 (H = 66.5) **D–F** Plekocheilus (Eurytus) taylorianus (Reeve, 1849), lectotype NHMUK 1874.12.11.271 (H = 58.5) **G–I** Plekocheilus (Eurytus) roseolabrum (E.A. Smith, 1877), lectotype NHMUK 1975135 (H = 42.0).

**Figure 7.**
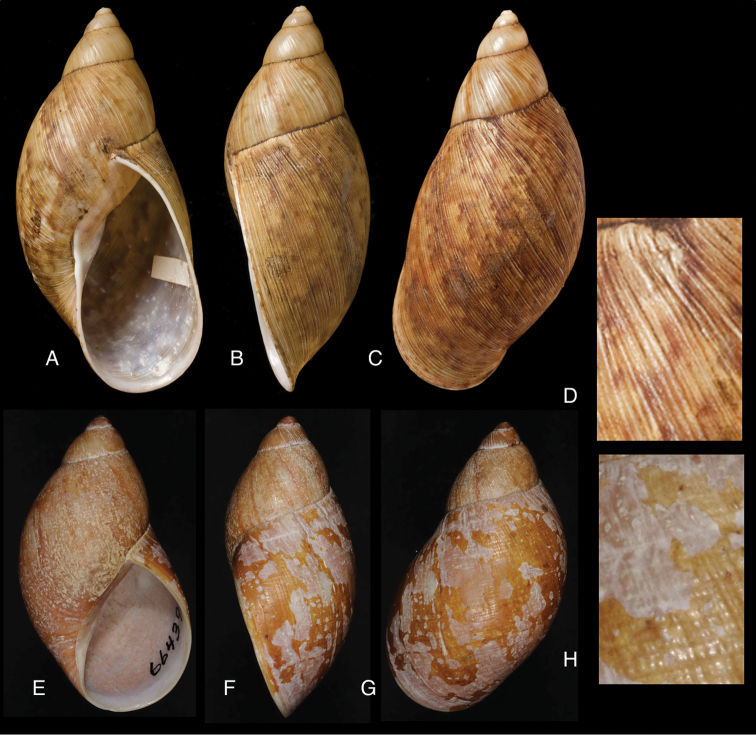
Plekocheilus species. **A–D** Plekocheilus (Eurytus) superstriatus (Sowerby III, 1890), lectotype NHMUK 1889.11.19.1 (H = 64.5) **D** detail of sculpture on dorsal side of last whorl **E–H** Plekocheilus (Eurytus) piperitus prodeflexus (Pilsbry, 1895), lectotype ANSP 66439 (H = 52.0) **H** detail of sculpture on dorsal side of last whorl.

**Figure 8.**
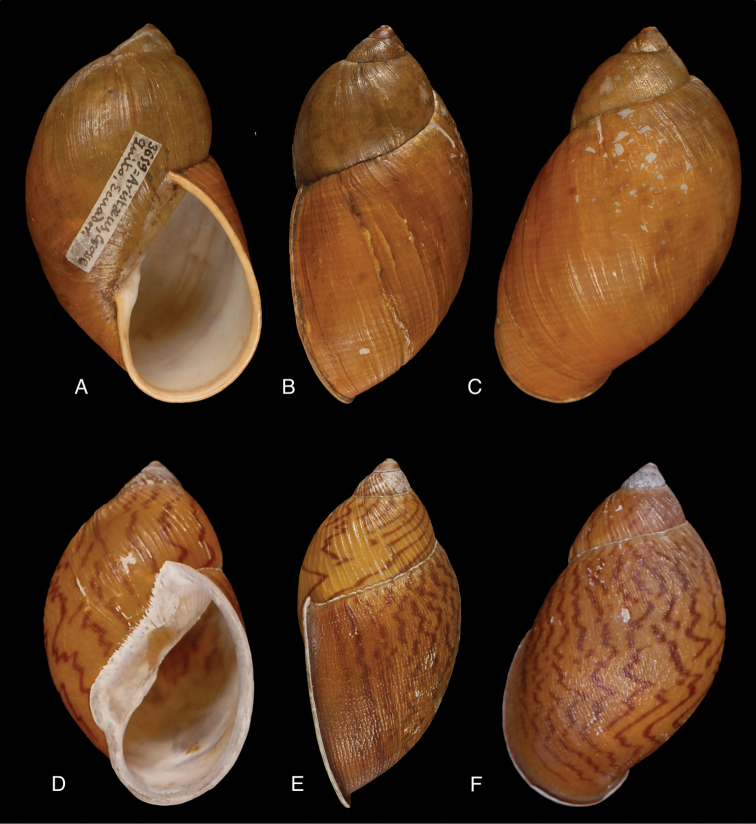
Plekocheilus species. **A–C** Plekocheilus (Eurytus) aristaceus (Crosse, 1869), lectotype MNCN 15.05/7180 (H = 48.3) **D–F** Plekocheilus (Plekocheilus) cecepeus Breure and Araujo, 2015, holotype MNCN 15.05/60013H (H = 44.8).

**Figure 9.**
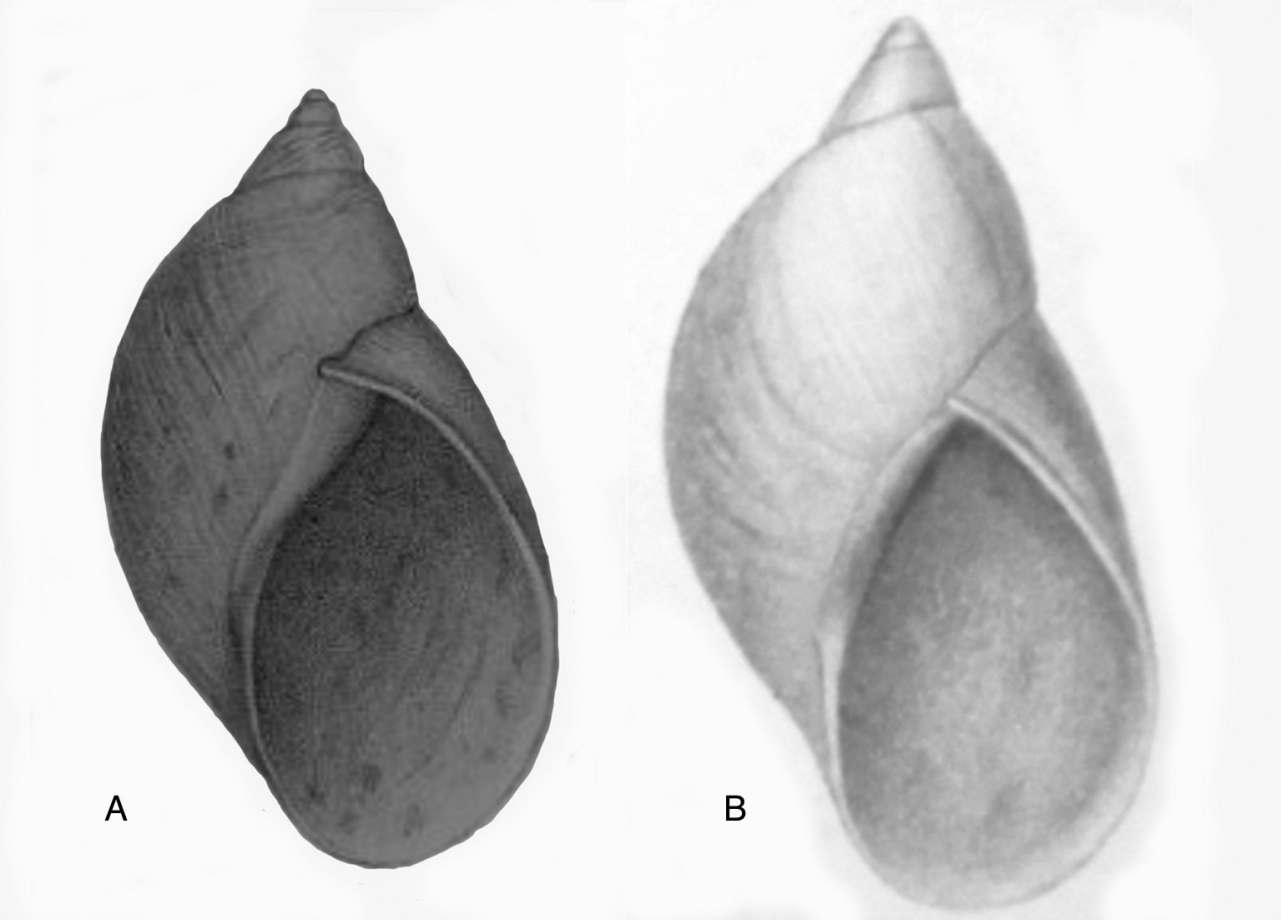
Plekocheilus species. **A** Plekocheilus (Eurytus) aureonitens (Miller, 1878), original figure Miller 1879: pl. 6 fig. 2 (H = 53.0) **B** Plekocheilus (Eurytus) taylorianus (Reeve, 1849), original figure of *Eurytus taylorioides minor* Miller, 1878, Miller 1879: pl. 7 fig. 1 (H = 59.0).

**Figure 10.**
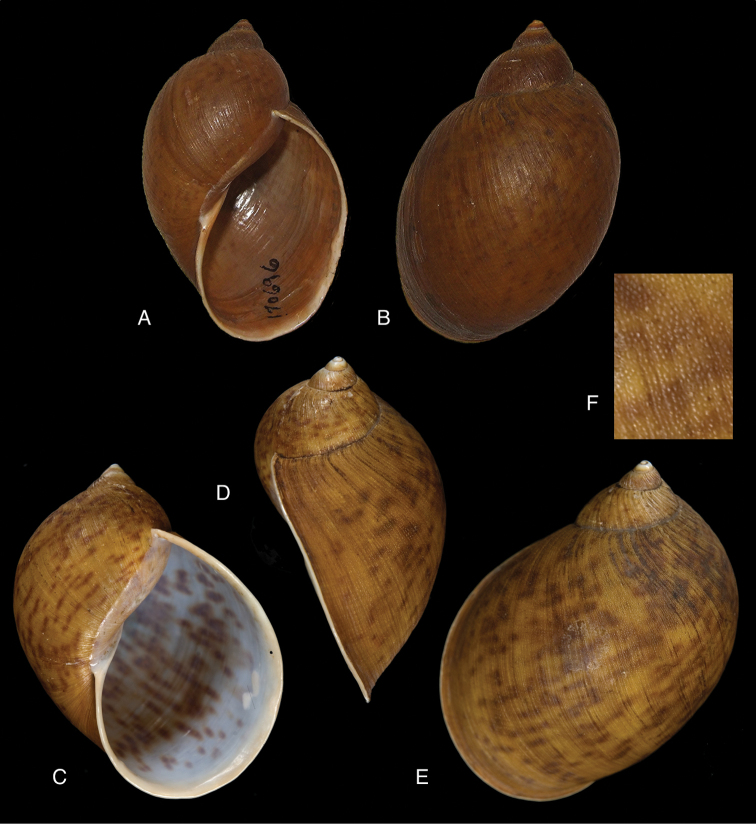
Plekocheilus species. **A–B** Plekocheilus (Eurytus) oligostylus Pilsbry, 1939, lectotype ANSP 170696 (H = 71.0) **C–F** Plekocheilus (Eurytus) jimenezi (Hidalgo, 1872) **C–E** syntype of *Bulimus jimenezi* Hidalgo 1872, MNCN 15.05/1066 (H = 69.0) **F** detail of sculpture on dorsal side of last whorl.

**Figure 11.**
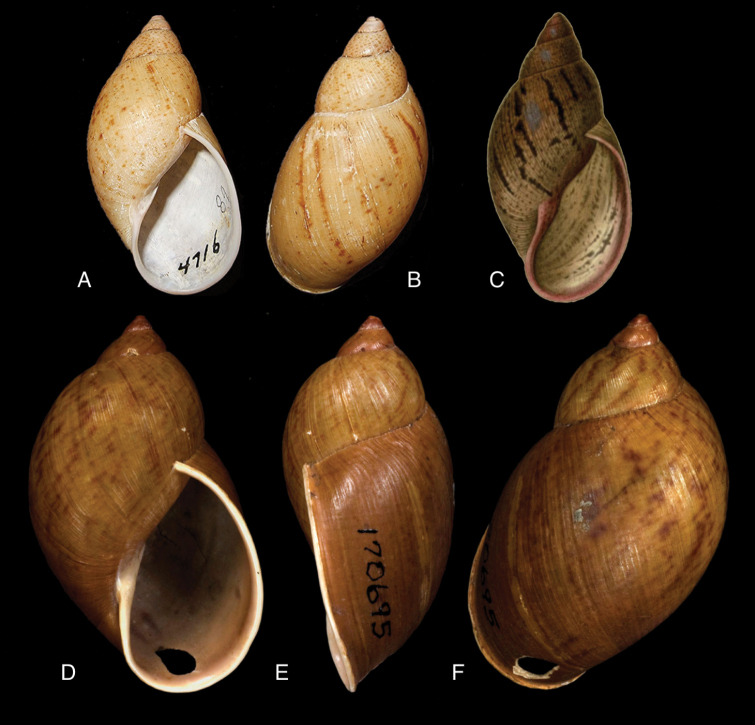
Plekocheilus species. **A–C** Plekocheilus (Eurytus) lynciculus (Deville and Hupé, 1850); **A–B** holotype of Plekocheilus (Eurytus) jacksoni Pilsbry, 1939, ANSP 170694 (H = 45.5) **C** original figure Deville and Hupé, 1850: pl. 15 fig. 1 **D–F** Plekocheilus (Eurytus) nocturnus Pilsbry, 1939, lectotype ANSP 170695 (H = 51.0).

**Figure 12.**
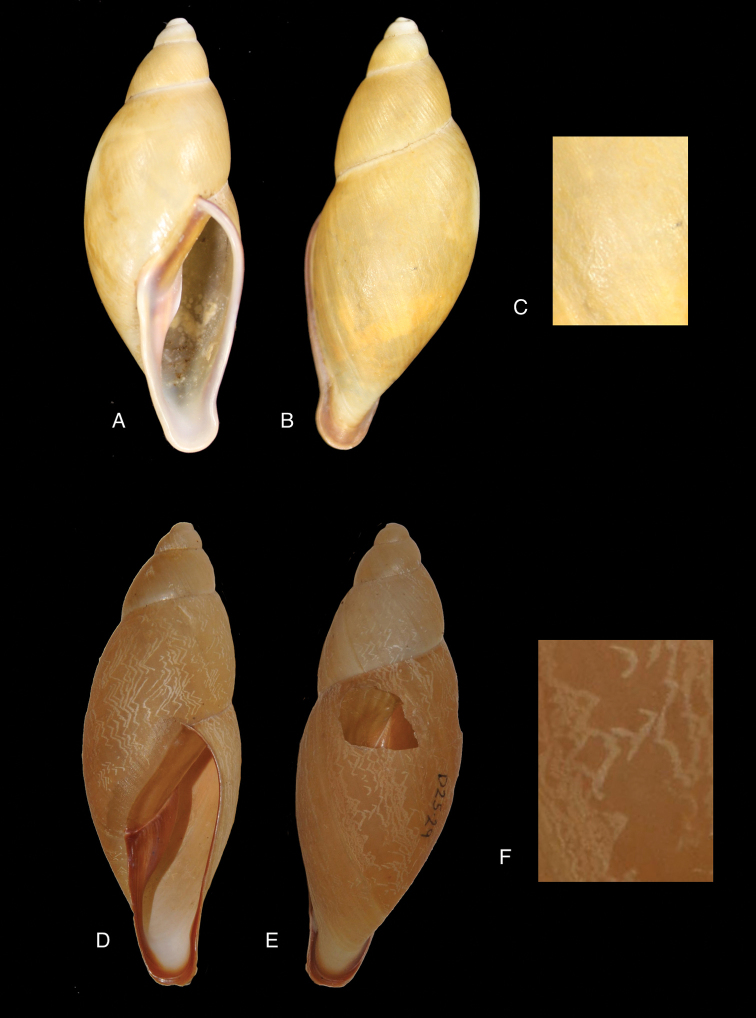
Plekocheilus species. **A–F** Plekocheilus (Eudolichotis) hauxwelli (Crosse, 1872) **A–C** paratype MCZ 202073 (H = 50.6) **C** detail of sculpture on dorsal side of last whorl **D–F** RAMM 1720/1909/D25/29 (H = 51.1) **F** detail of sculpture on dorsal side of last whorl.

**Figure 13.**
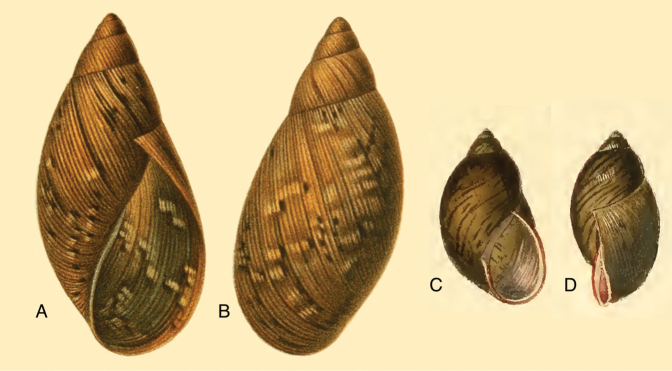
Plekocheilus species. **A–B** Plekocheilus (Eurytus) floccosus (Spix in Wagner, 1827); original figure of *Bulimus lacrimosus* Heimburg, 1884 [Heimburg 1887: pl. 11 fig. 1] (H = 62) **C–D** Plekocheilus (Eurytus) tricolor (Pfeiffer, 1853) [Küster and Pfeiffer 1853 (1840–1865): pl. 32 figs 17–18] (H = 45.8).

**Figure 14.**
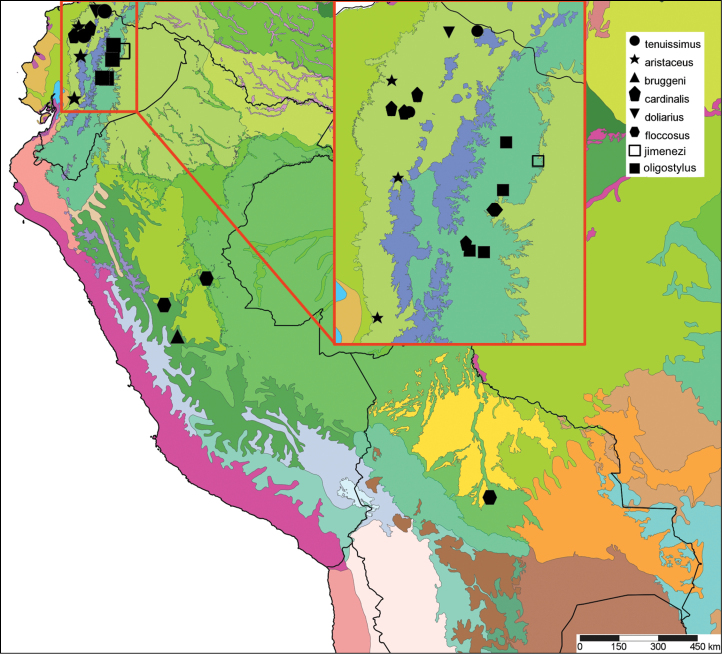
Distribution map of Plekocheilus (Eurytus) species. See Figure 91 and Appendix 4 for explanation of ecoregions.

**Figure 15.**
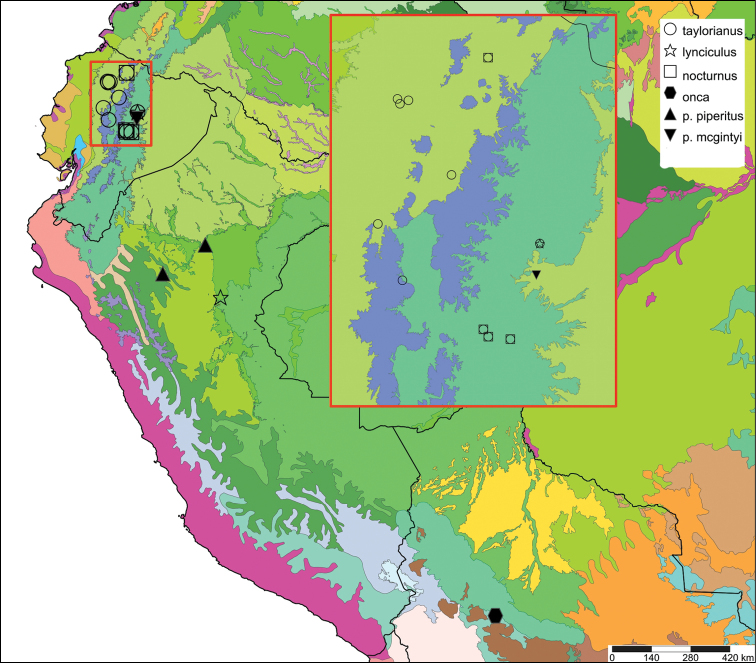
Distribution map of Plekocheilus (Eurytus) species. See Figure 91 and Appendix 4 for explanation of ecoregions.

**Figure 16.**
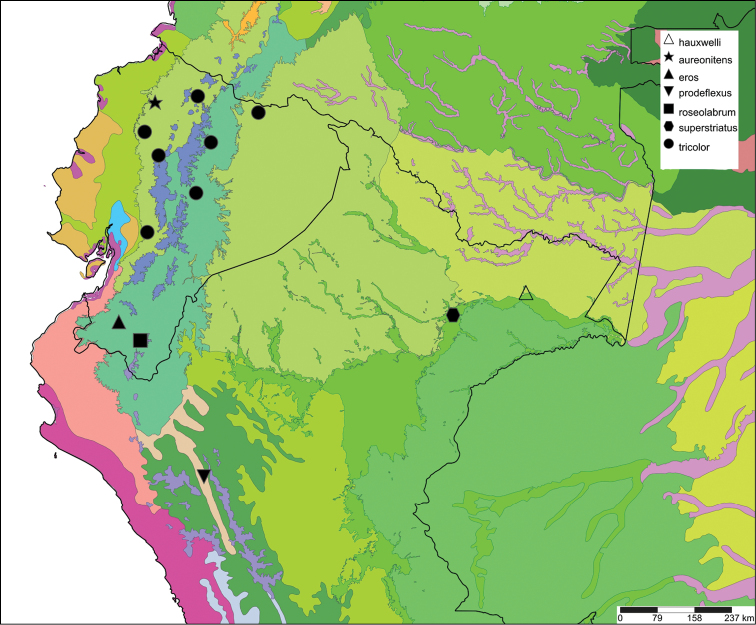
Distribution map of Plekocheilus (Eurytus) and Plekocheilus (Eudolichotis) species. See Figure 91 and Appendix 4 for explanation of ecoregions.

**Figure 17.**
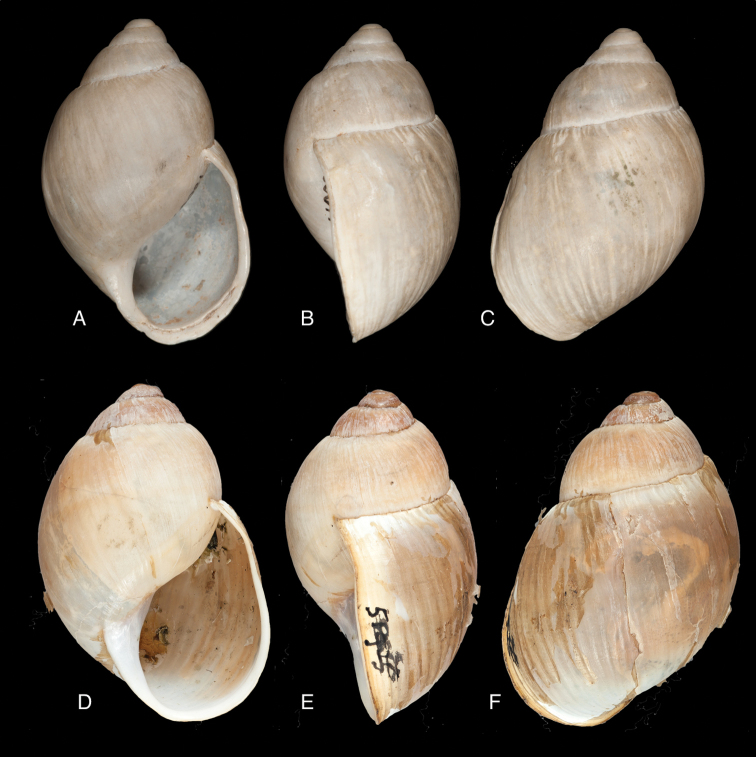
*Paeniscutalus* species. **A–E** *Paeniscutalus crenellus* (Philippi, 1867) **A–C** holotype of Megalobulimus (Microborus) incarum Pilsbry, 1944, ANSP 180677 (H = 35) **D–E** holotype of Strophocheilus (Microborus) tenuis Haas, 1955, FMNH 51925 (H = 30.1).

**Figure 18.**
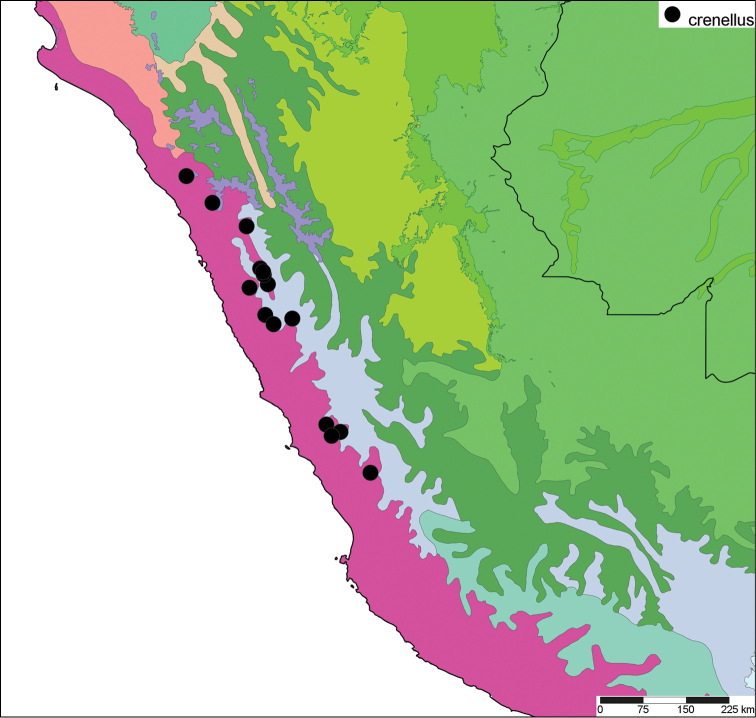
Distribution map of Paeniscutalus crenellus (Philippi, 1867). See Figure 91 and Appendix 4 for explanation of ecoregions.

**Figure 19.**
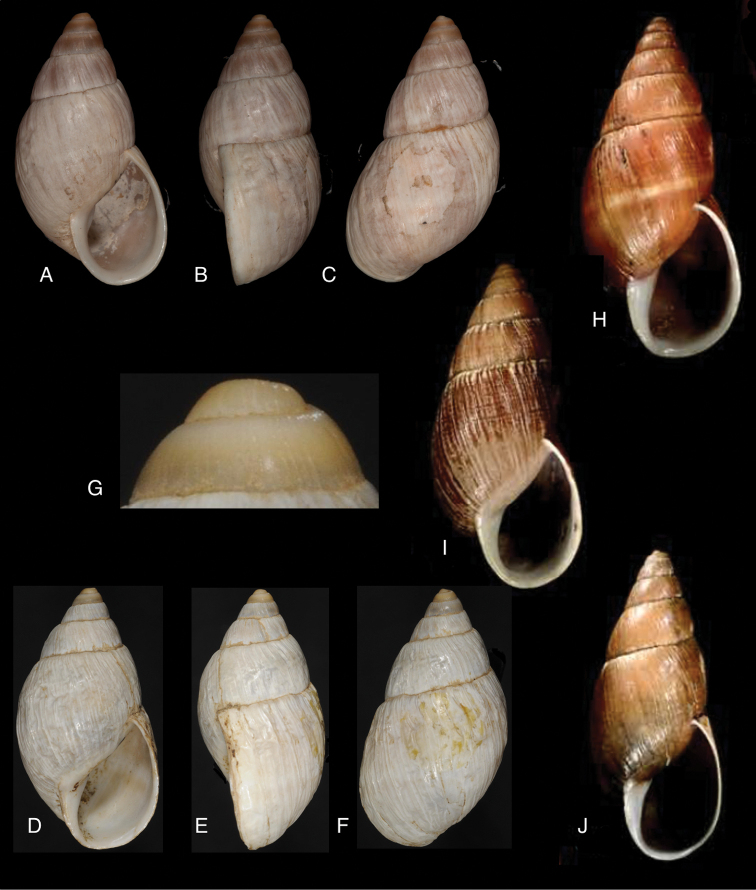
Thaumastus species. **A–C** Thaumastus (Thaumastiella) sarcochrous (Pilsbry, 1897), holotype ANSP 4705 (H = 29.0) **D–G** Thaumastus (Thaumastiella) glyptocephalus (Pilsbry, 1897), syntype ANSP 25675 (H = 31.0) **G** detail of protoconch sculpture **H** Thaumastus (Thaumastiella) koepckei Zilch, 1953, holotype SMF 111487(H = 46.6) **I** Thaumastus (Thaumastiella) occidentalis occidentalis Weyrauch, 1960, holotype SMF 162026 (H = 45.7) **J** Thaumastus (Thaumastiella) occidentalis debilisculptus Weyrauch, 1960, holotype SMF 162029 (H = 40.0).

**Figure 20.**
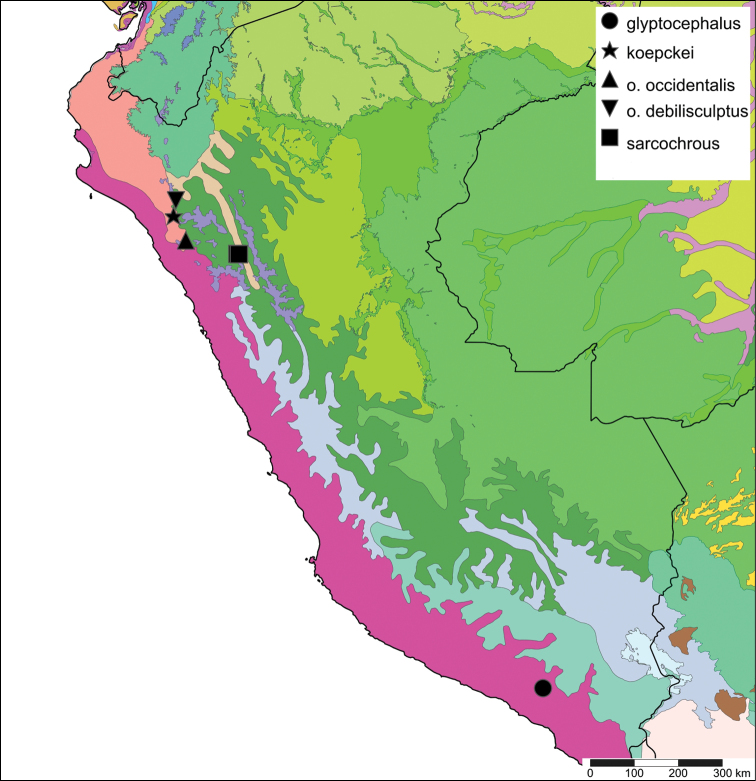
Distribution map of Thaumastus (Thaumastiella) species. See Figure 91 and Appendix 4 for explanation of ecoregions.

**Figure 21.**
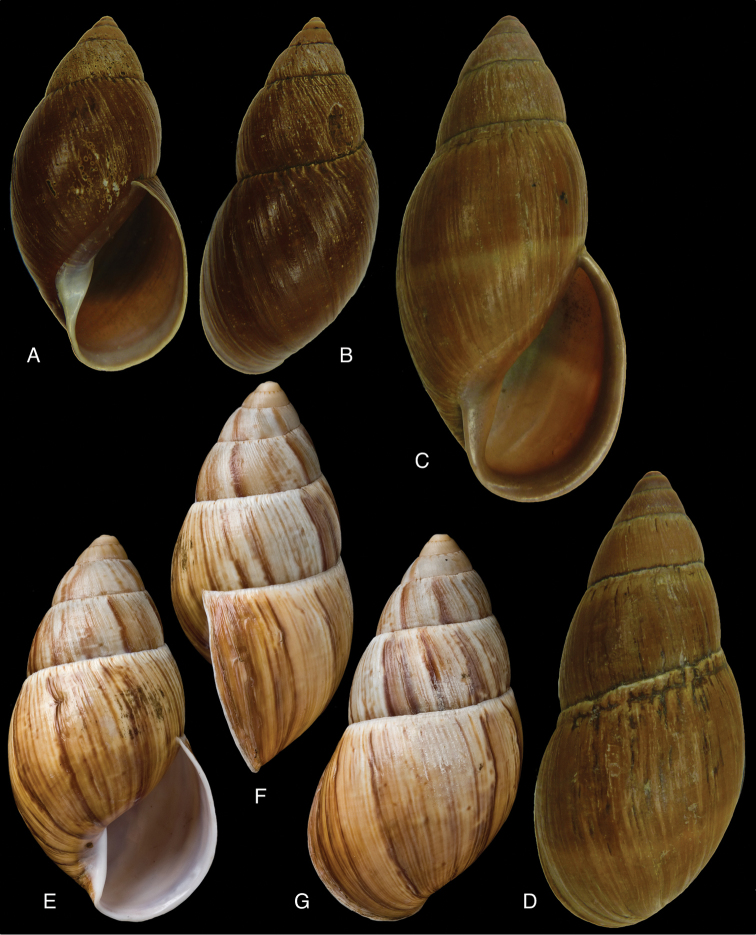
Thaumastus species. **A–B** Thaumastus (Thaumastus) blanfordianus (Ancey, 1903), holotype RBINS/MT (H = 52.5) **C–D** Thaumastus (Thaumastus) melanocheilus (Nyst, 1845), lectotype RBINS/MT2361 (H = 78.5) **E–G** Thaumastus (Thaumastus) hartwegi (Pfeiffer in Philippi, 1846), syntype NHMUK 1975126 (H = 57.0).

**Figure 22.**
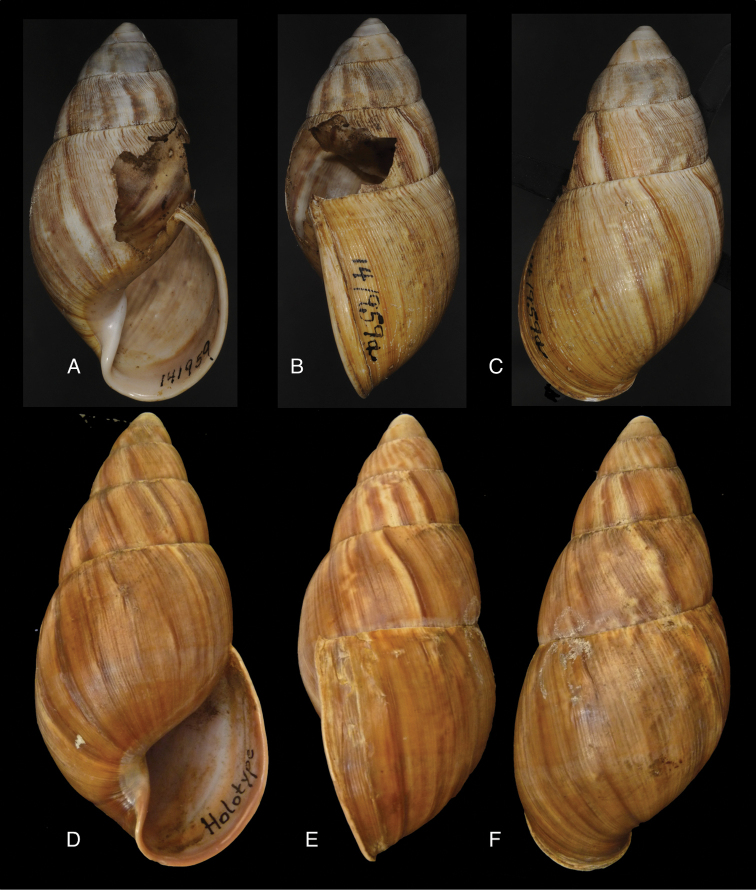
Thaumastus species. **A–C** Thaumastus (Thaumastus) hartwegi (Pfeiffer in Philippi, 1846), holotype of Plekocheilus (Eurytus) conspicuus Pilsbry, 1932 ANSP 141959 (H = 64.5) **D–F** Thaumastus (Thaumastus) flori (Jousseaume, 1897), holotype MNHN 22474 (H = 85.3).

**Figure 23.**
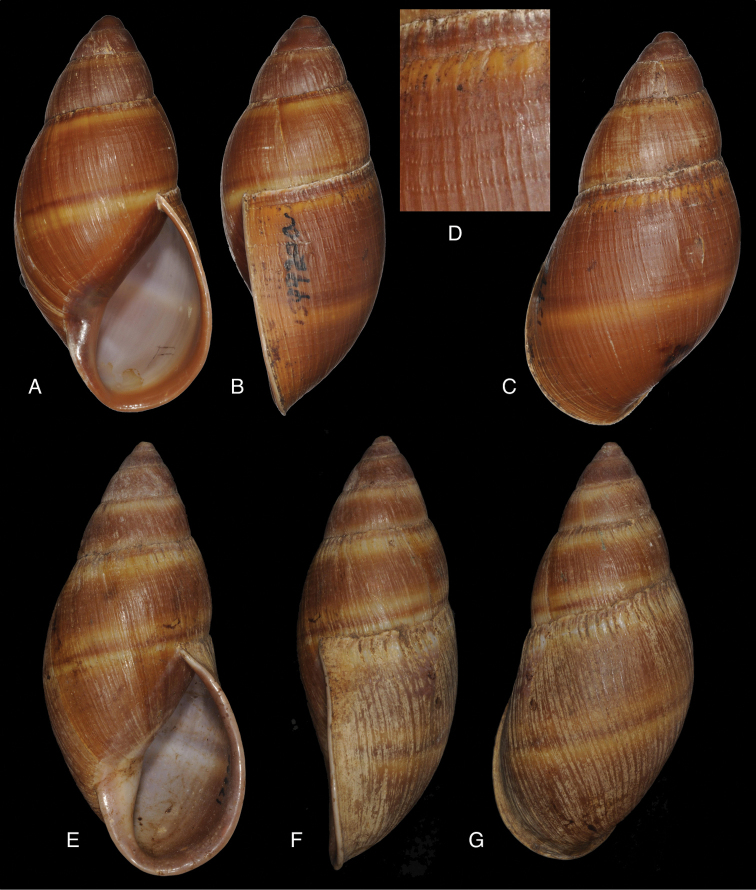
Thaumastus species. **A–D** Thaumastus (Thaumastus) robertsi robertsi Pilsbry, 1932, holotype ANSP 159920 (H = 63.7) **D** sculpture on dorsal side of last whorl **E–G** Thaumastus (Thaumastus) robertsi satipoensis Pilsbry, 1944, holotype ANSP 179990 (H = 74.4).

**Figure 24.**
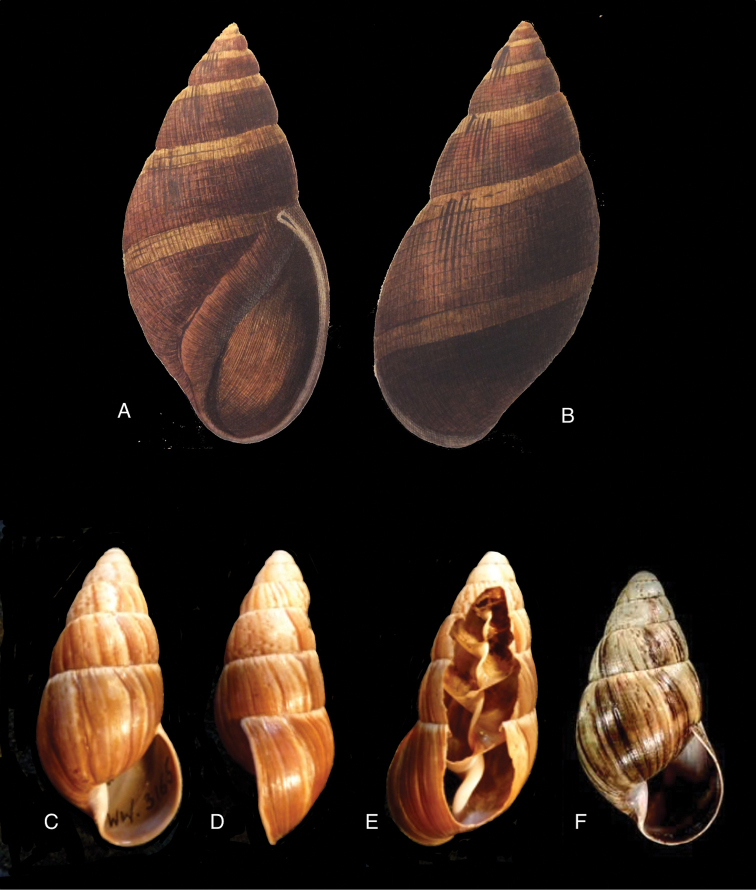
Thaumastus species. **A–B**Thaumastus (Thaumastus) sangoae (Tschudi in Troschel, 1852), Troschel 1852: pl. 6 figs 1a–b (H = 81) **C–F** Thaumastus (Thaumastus) orcesi Weyrauch, 1967 **C–E** holotype FML 3165 (H = 49.4) **F** paratype SMF 156325 (H = 45.9).

**Figure 25.**
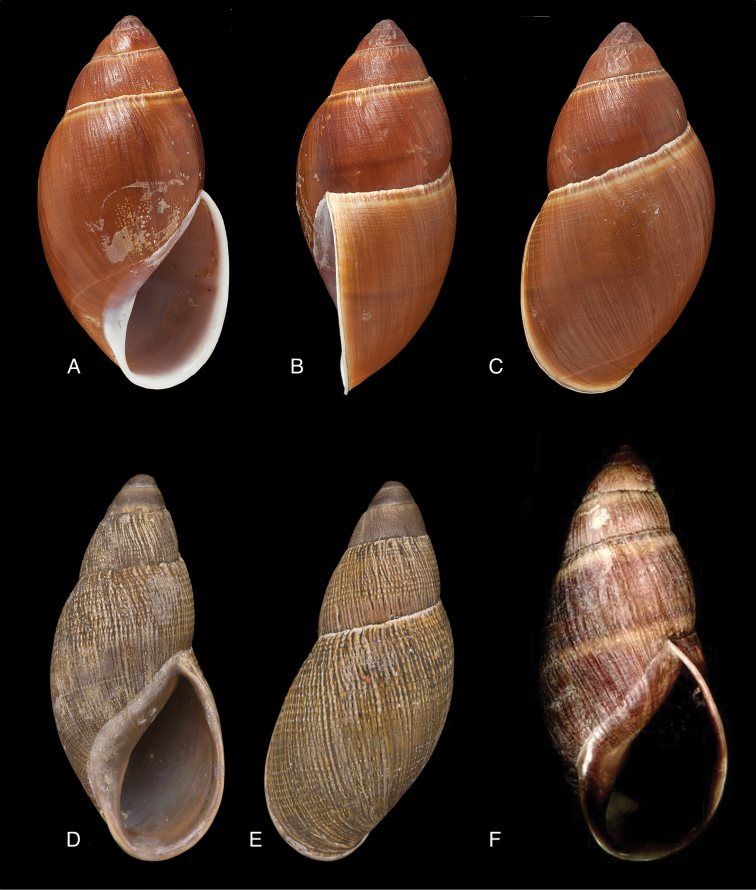
Thaumastus species. **A–C** Thaumastus (Thaumastus) foveolatus (Reeve, 1849), lectotype NHMUK 1975275 (H = 71.5) **D–E** Thaumastus (Thaumastus) insolitus (Preston, 1909), holotype NHMUK 1947.3.11.1 (H = 70.4) **F** Thaumastus (Thaumastus) granocinctus Pilsbry, 1901, syntype SMF 208383 (H = 80.5).

**Figure 26.**
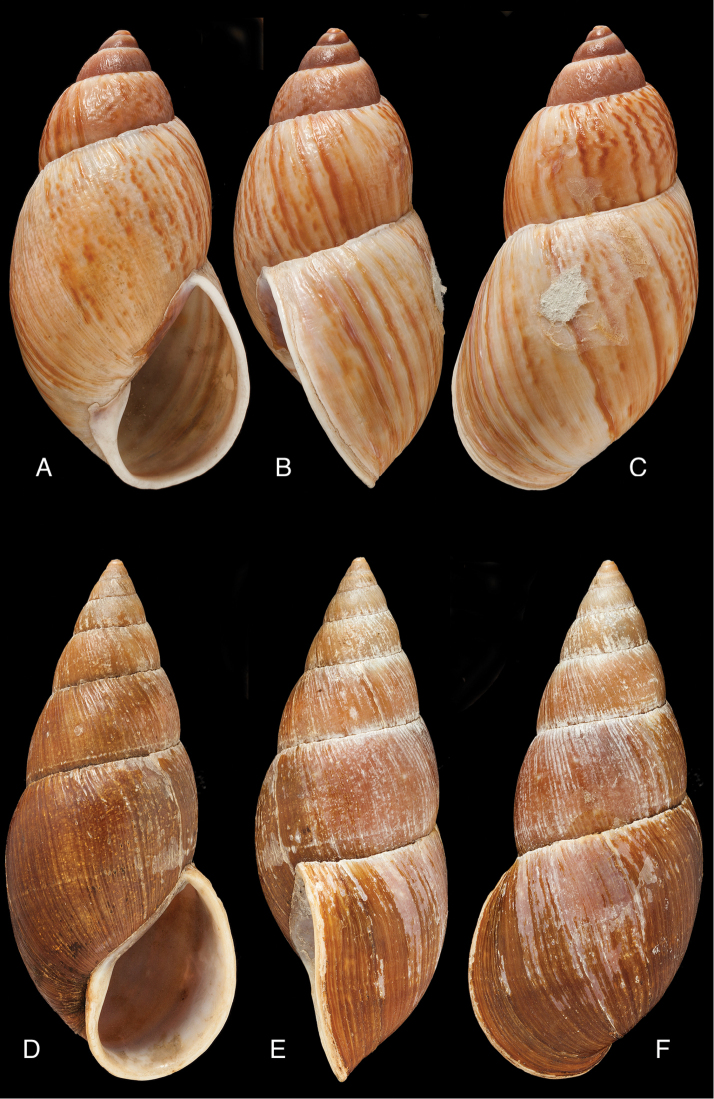
Thaumastus species. **A–C** Thaumastus (Thaumastus) loxostomus (Pfeiffer, 1853), syntype NHMUK 1975125 (H = 71.3) **D–F** Thaumastus (Thaumastus) inca (d’Orbigny, 1835), lectotype NHMUK 1854.12.4.116 (H = 75.4).

**Figure 27.**
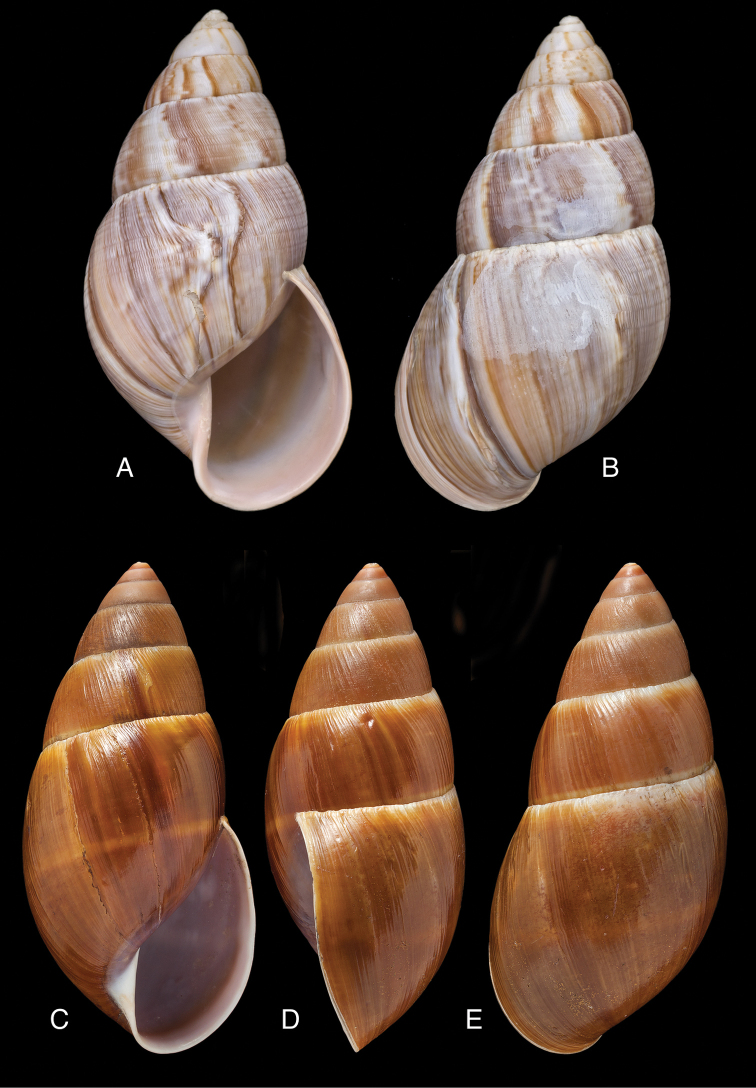
Thaumastus species. **A–B** Thaumastus (Thaumastus) integer (Pfeiffer, 1855), lectotype NHMUK 1975244 (H = 81.5) **C–E** Thaumastus (Thaumastus) magnificus (Grateloup, 1839), lectotype NHMUK 1907.11.22.24 (H = 78.0).

**Figure 28.**
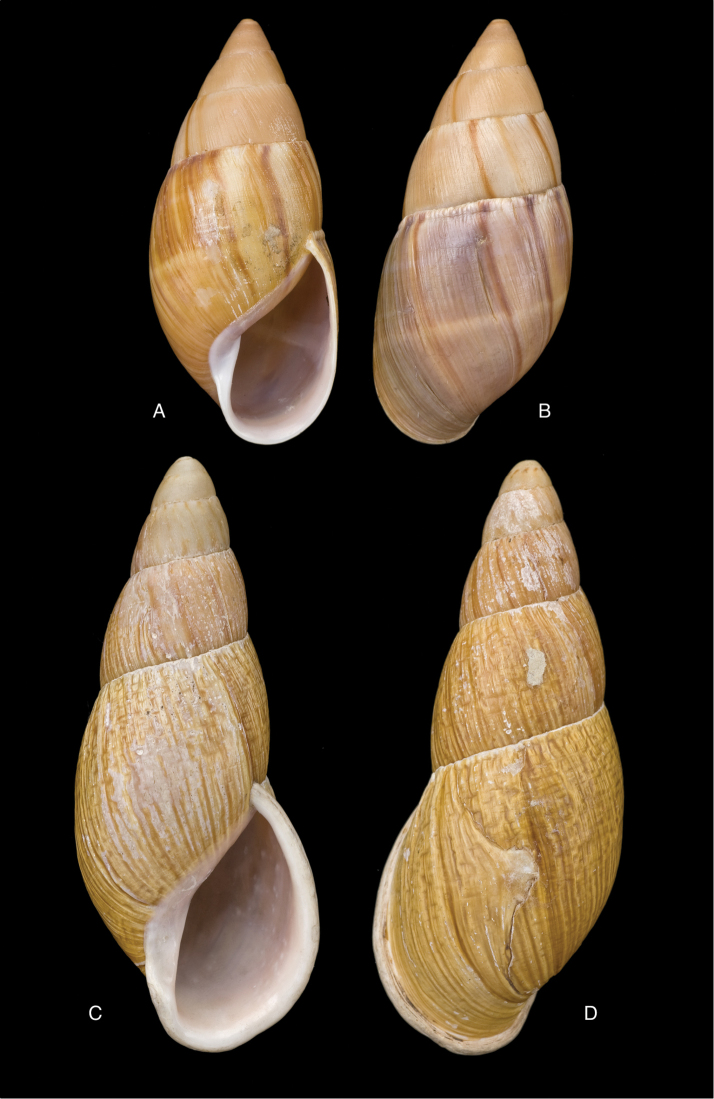
Thaumastus species. **A–B** Thaumastus (Thaumastus) taunaisii (Férussac, 1822), lectotype of *Bulimus achilles* Pfeiffer, 1853, NHMUK 1975268 (H = 58.0) **C–D** Thaumastus (Thaumastus) buckleyi (Higgins, 1872), syntype NHMUK 1875.5.2.6 (H = 92.0).

**Figure 29.**
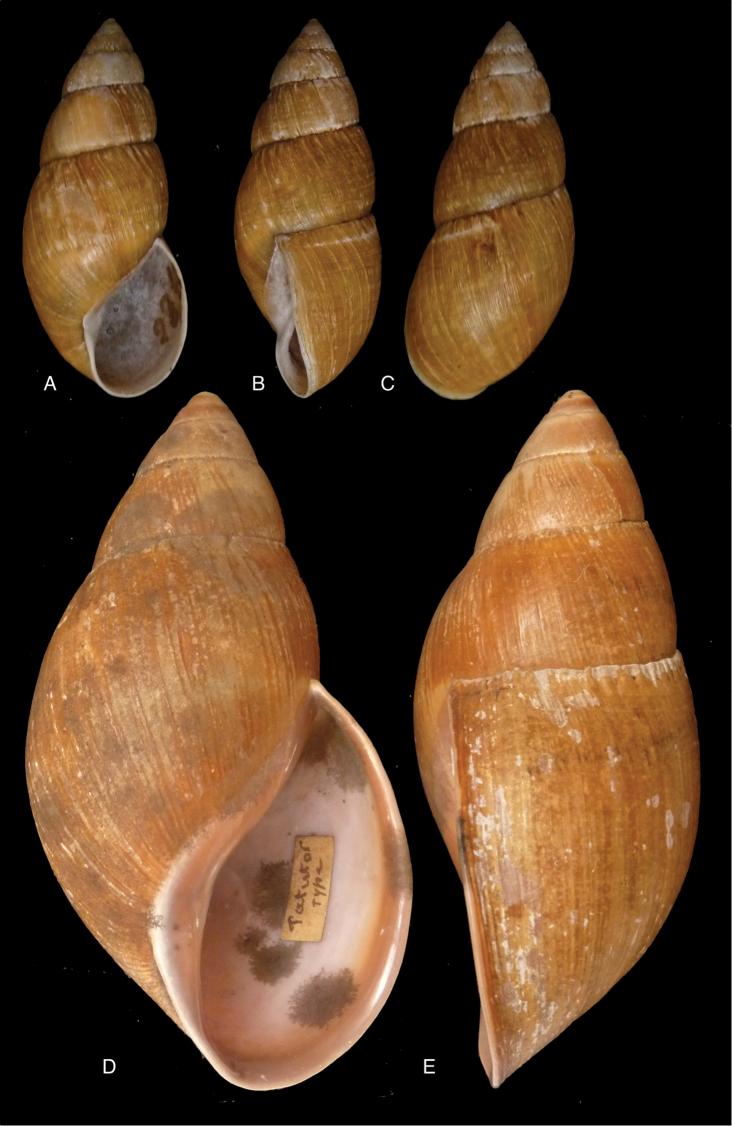
Thaumastus species. **A–C** Thaumastus (Thaumastus) orobaenus (d’Orbigny, 1835), lectotype MNHN 28091 (H = 38.8) **D–E** Thaumastus (Thaumastus) tatutor (Jousseaume, 1887), holotype MNHN 28122 (H = 100.0).

**Figure 30.**
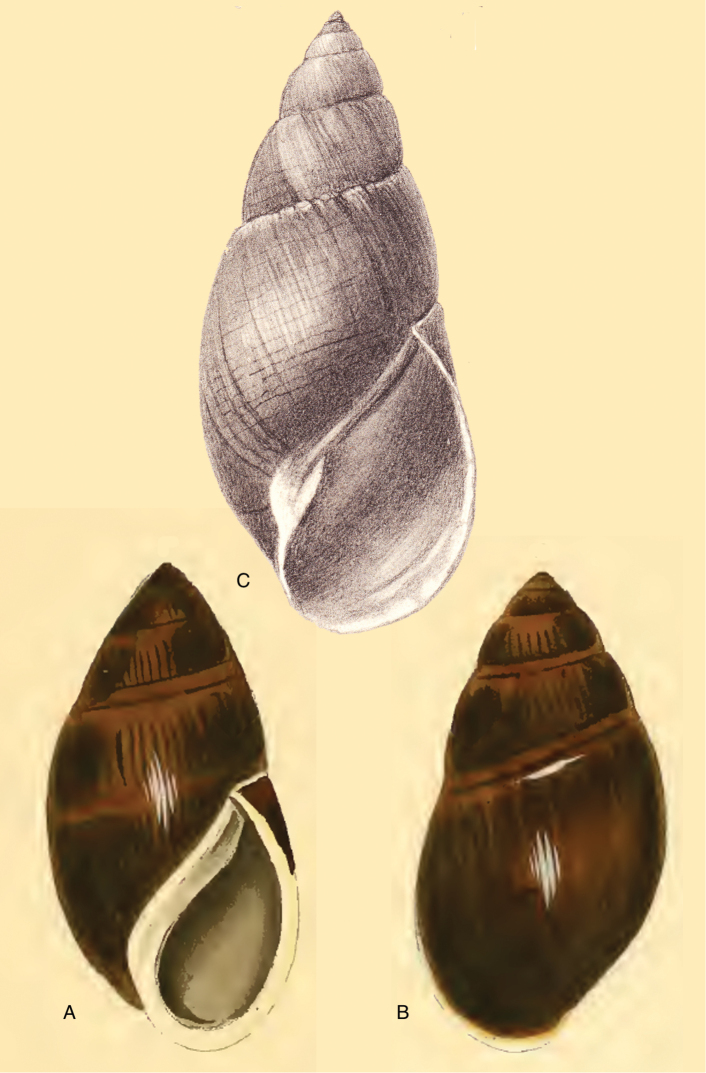
Thaumastus species. **A–B** Thaumastus (Thaumastus) foveolatus (Reeve, 1849); original figure of *Bulimus mahogani* Pfeiffer, 1841 [Küster and Pfeiffer 1844 (1840–1865): pl. 13 figs 1–2] (H = [65.4]) **C** Thaumastus (Thaumastus) inca (d’Orbigny, 1835); original figure Thaumastus (Atahualpa) brunneus Strebel 1910 [pl. 2 fig. 25] (H = 79.2).

**Figure 31.**
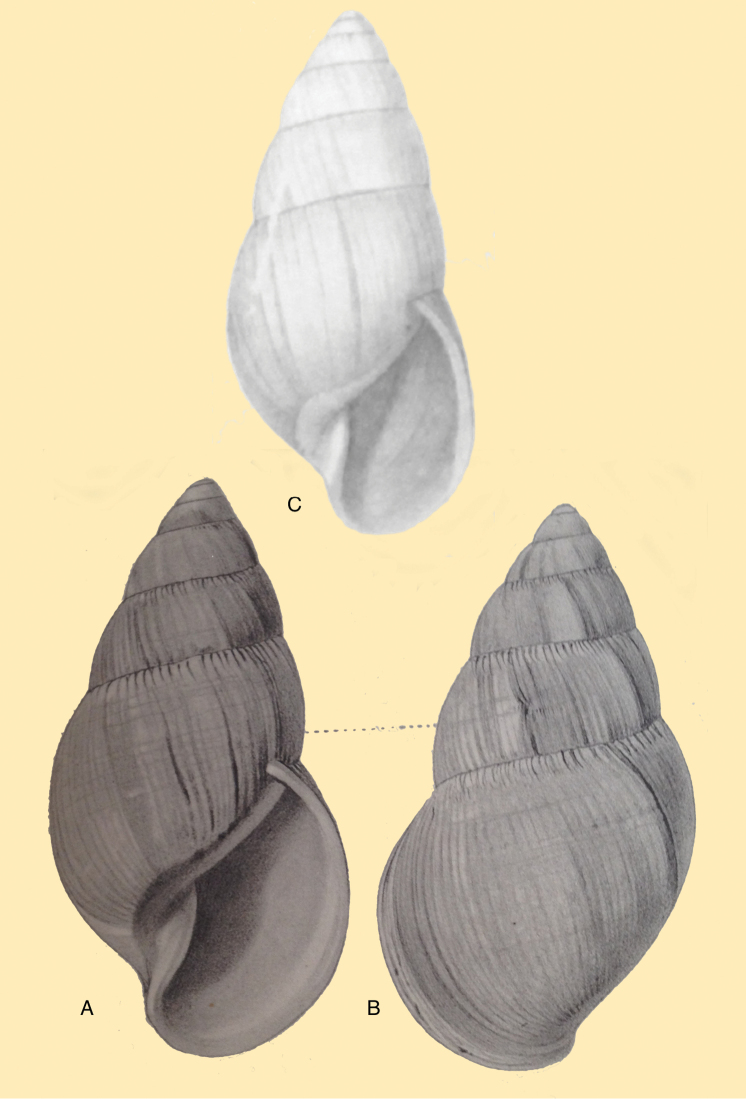
Thaumastus species. **A–B** Thaumastus (Thaumastus) integer (Pfeiffer, 1855); original figure of *Pachytholus pseudoiostomus* Strebel, 1909 [pl. 26 figs 397-398] (H = 73.0) **C** Thaumastus (Thaumastus) hartwegi (Pfeiffer in Philippi, 1846); original figure of *Zebra loxensis* Miller, 1879 [pl. 12 fig. 2] (H = 70).

**Figure 32.**
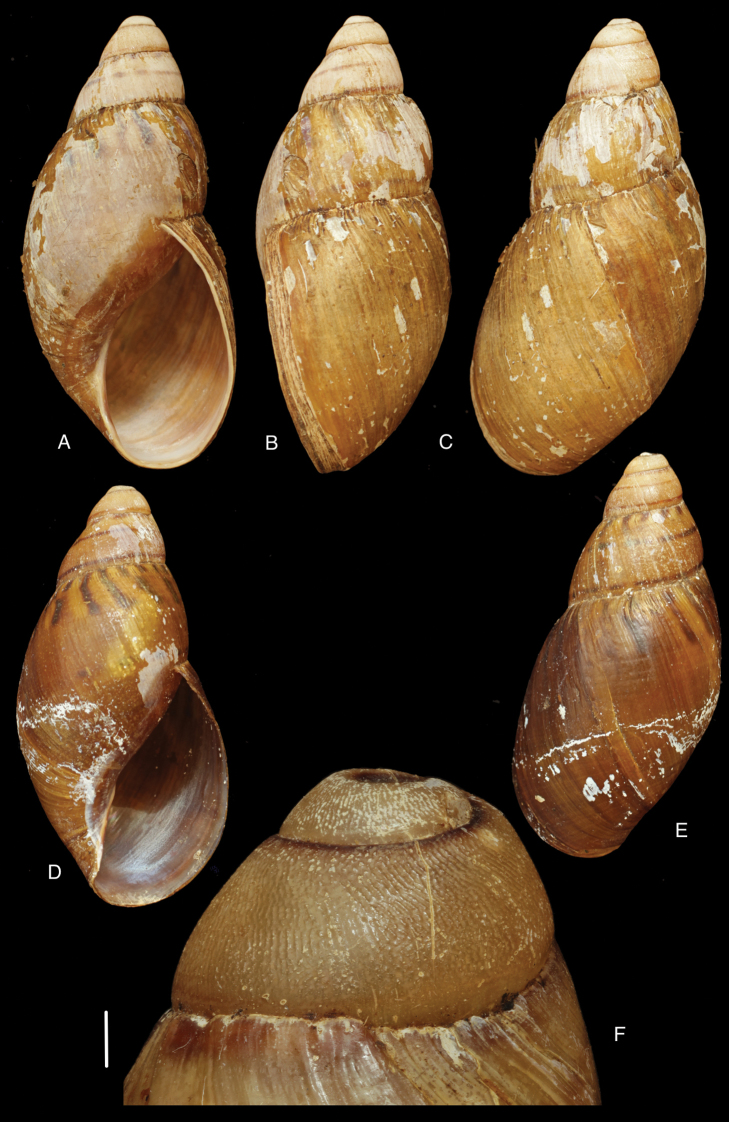
Thaumastus (Thaumastus) sumaqwayqu sp.n. **A–C** Holotype RMNH 201636 (H = 52.5) **D–E** Paratype VMA (H = 48.4) **F** Protoconch sculpture, paratype RMNH 201637; scale line = 1 mm.

**Figure 33.**
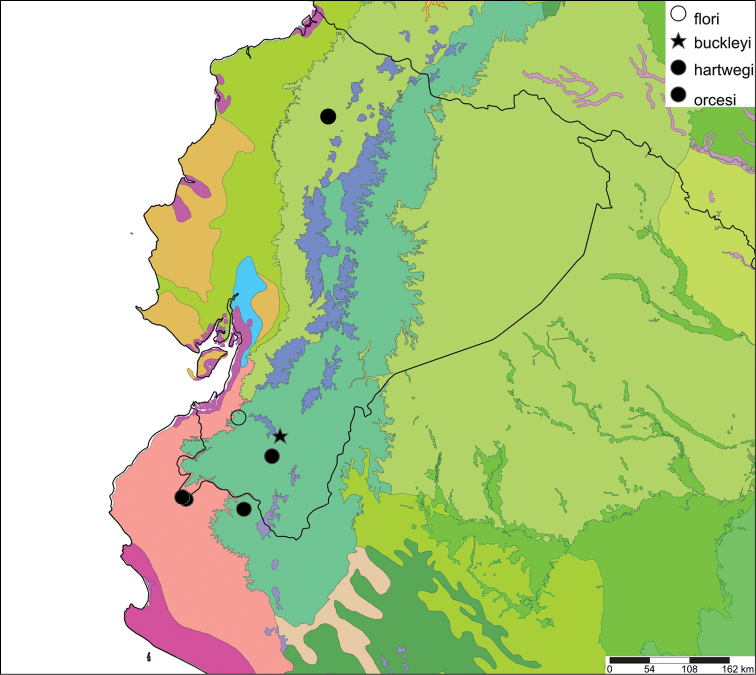
Distribution map of Thaumastus (Thaumastus) species. See Appendix 4 for explanation of ecoregions.

**Figure 34.**
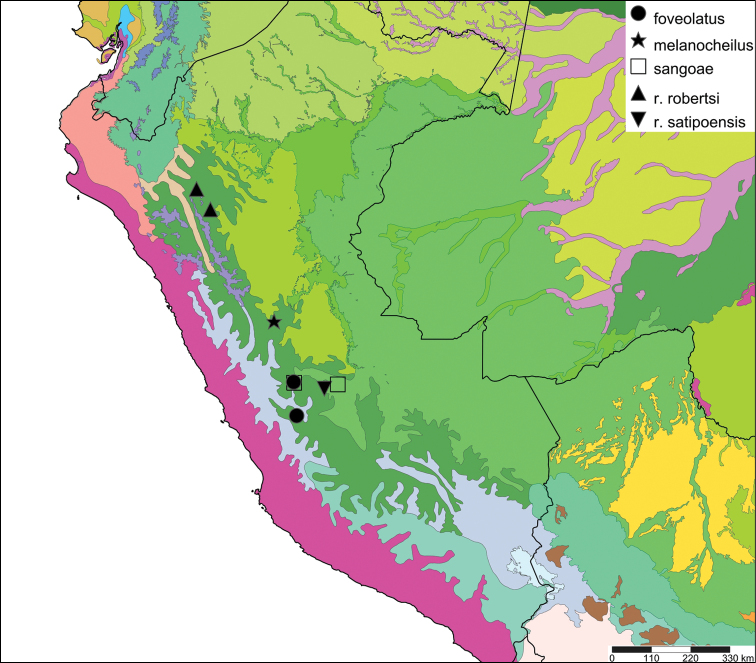
Distribution map of Thaumastus (Thaumastus) species (continued). See Figure 91 and Appendix 4 for explanation of ecoregions.

**Figure 35.**
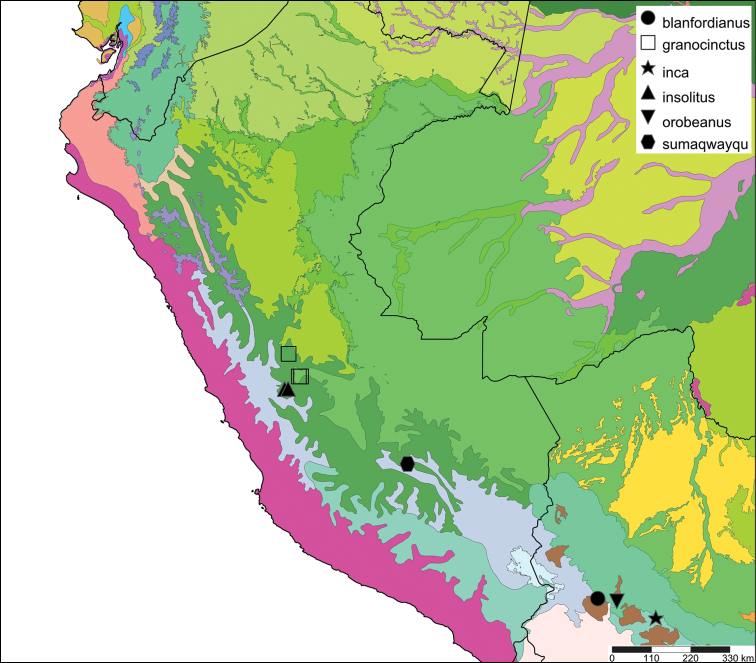
Distribution map of Thaumastus (Thaumastus) species (continued). See Figure 91 and Appendix 4 for explanation of ecoregions.

**Figure 36.**
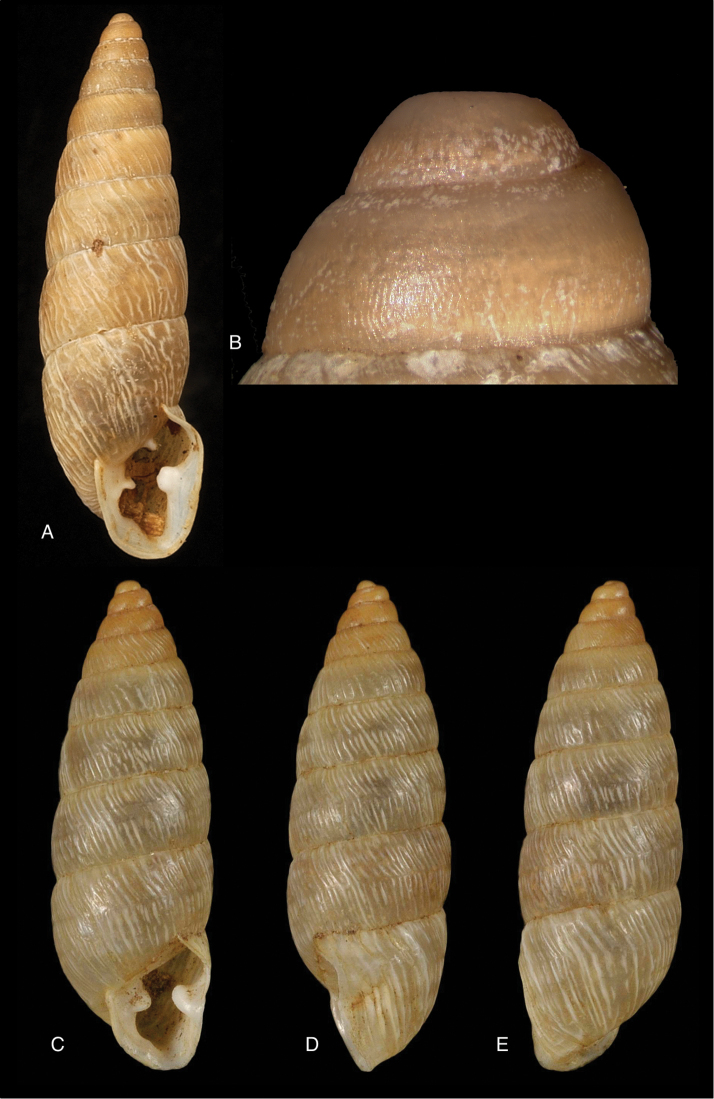
*Cyclondontina* and Spixia species. **A–B** *Cyclodontina lemoinei* (Ancey, 1892), possible syntype NMW 1955.158.24077 (H = 22.0) **B** detail with protoconch sculpture **C–E** *Spixia chuquisacana* (Marshall, 1930), holotype USNM 380700 (H = 17.0).

**Figure 37.**
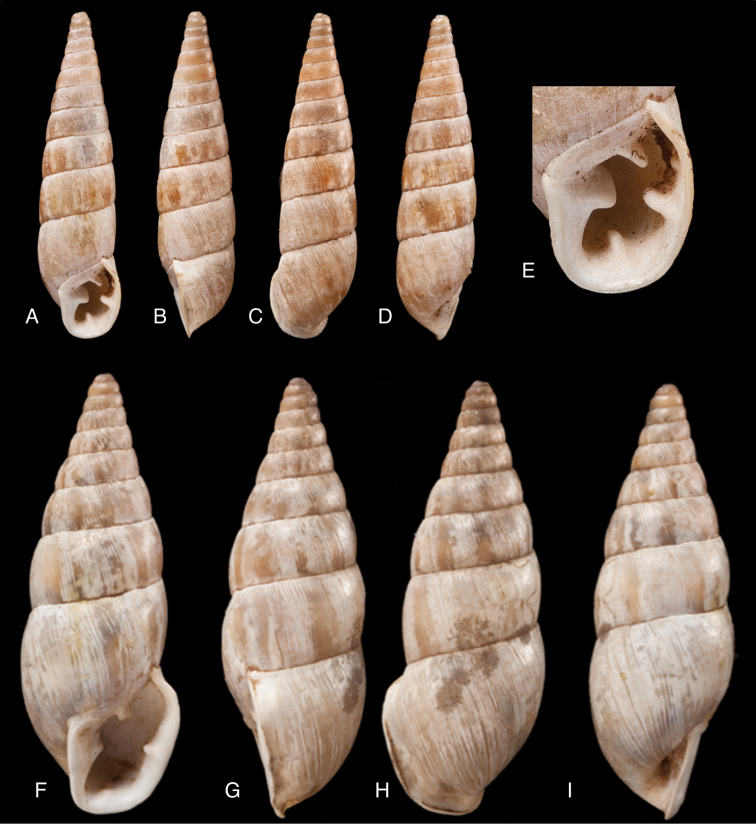
*Spixia* species. **A–E** Spixia minor (d’Orbigny, 1837), lectotype NHMUK 1854.12.4.231 (H = 29.2) **E** detail of aperture **F–I** Spixia striata (Wagner, 1827), lectotype of Pupa spixii var. major d’Orbigny, 1837, NHMUK 1854.12.4.232 (H = 34.8).

**Figure 38.**
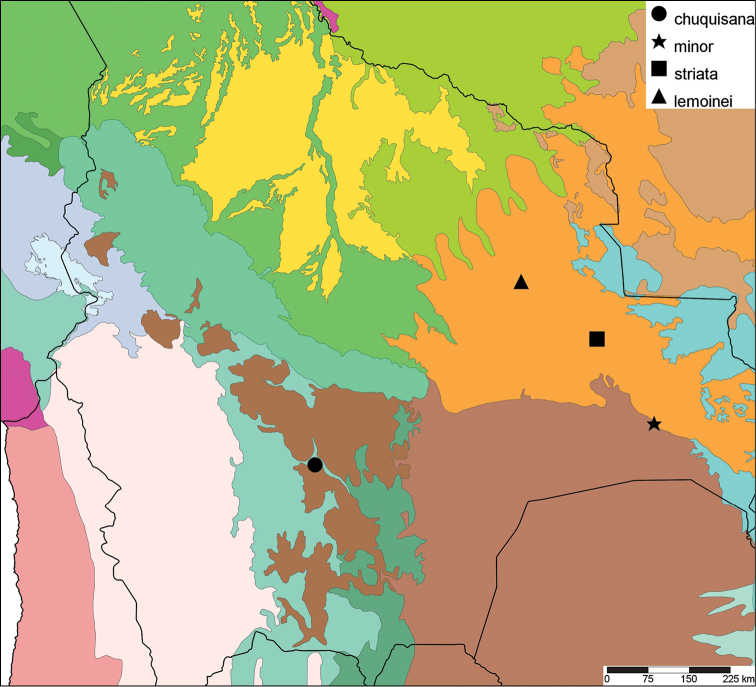
Distribution map of *Cyclondontina* and Spixia species. See Figure 91 and Appendix 4 for explanation of ecoregions.

**Figure 39.**
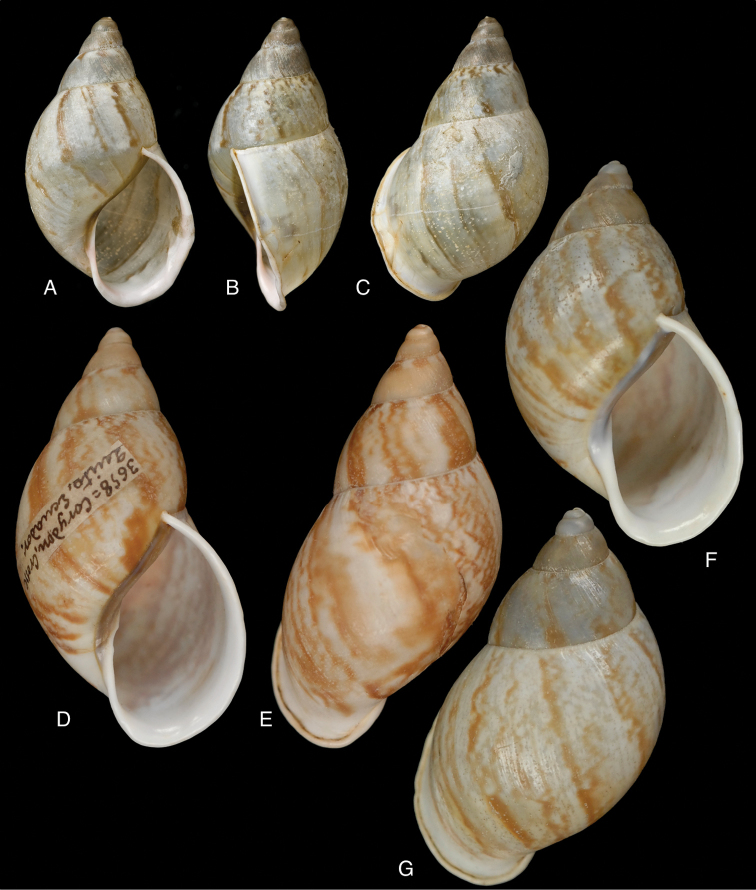
*Clathrorthalicus* species. **A–C** Clathrorthalicus phoebus (Pfeiffer, 1863), lectotype NHMUK 1975134 (H = 30.5) **D–G** Clathrorthalicus corydon (Crosse, 1869) **D–E** syntype MNCN 15.05/8077 (H = 42.1) **F–G** syntype MNCN 15.05/21868 (H = 38.2).

**Figure 40.**
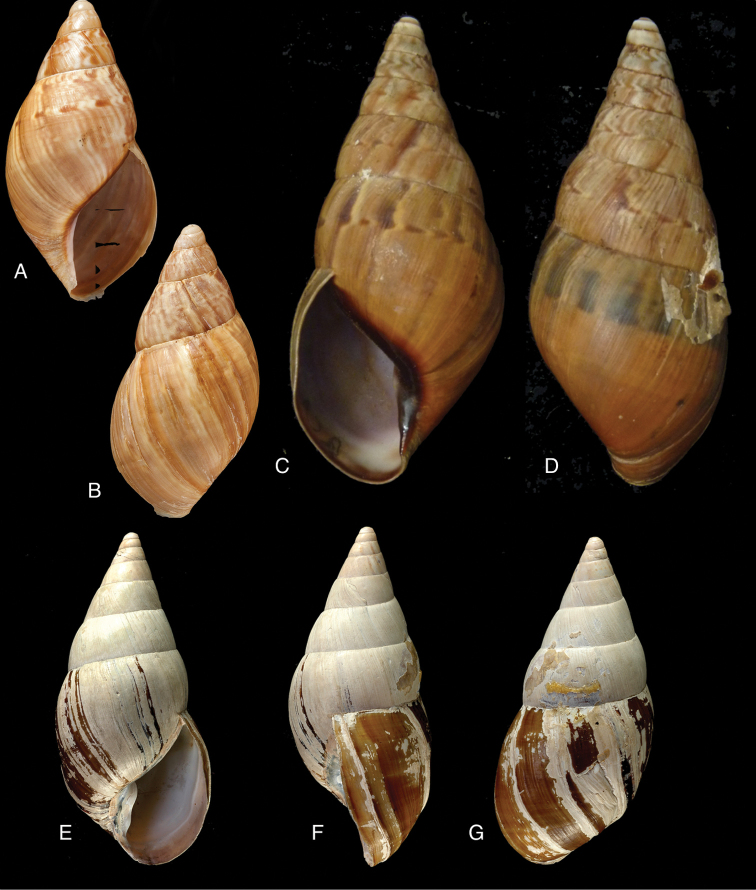
*Clathorthalicus* and *Corona* species. **A–B** *Clathorthalicus magnificus* (Pfeiffer, 1848), syntype NHMUK 20100508 (H = 46.6) **C–D** Corona incisa (Hupé, 1857), lectotype of *Bulimus incisus* Hupé MNHN 28068 (H = 73.8) **E–G** *Corona regina* (Férussac, 1823), lectotype MNHN 21881 (H = 45).

**Figure 41.**
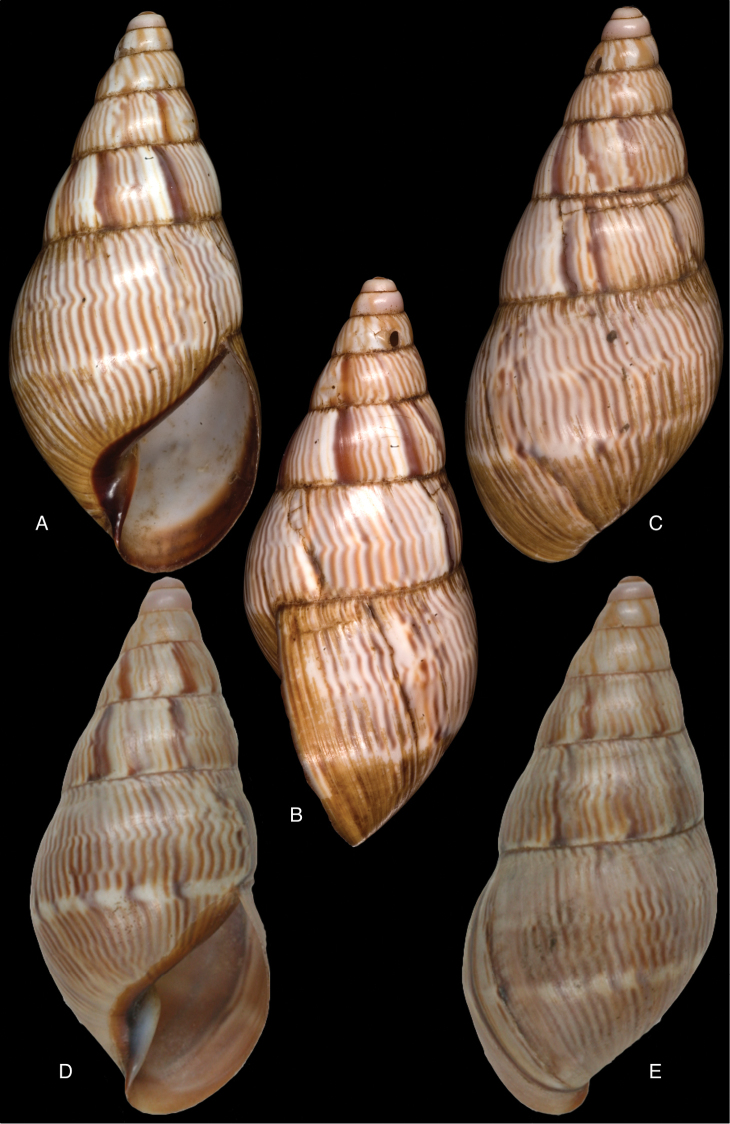
*Corona* species. **A–E** *Corona pfeifferi* (Hidalgo, 1869) **A–C** syntype MNCN 15.05/3280 (H = 56.3) **D–E** syntype of Corona pfeifferi cincta Strebel, 1909, ZMB 101836 (H = 55.0)

**Figure 42.**
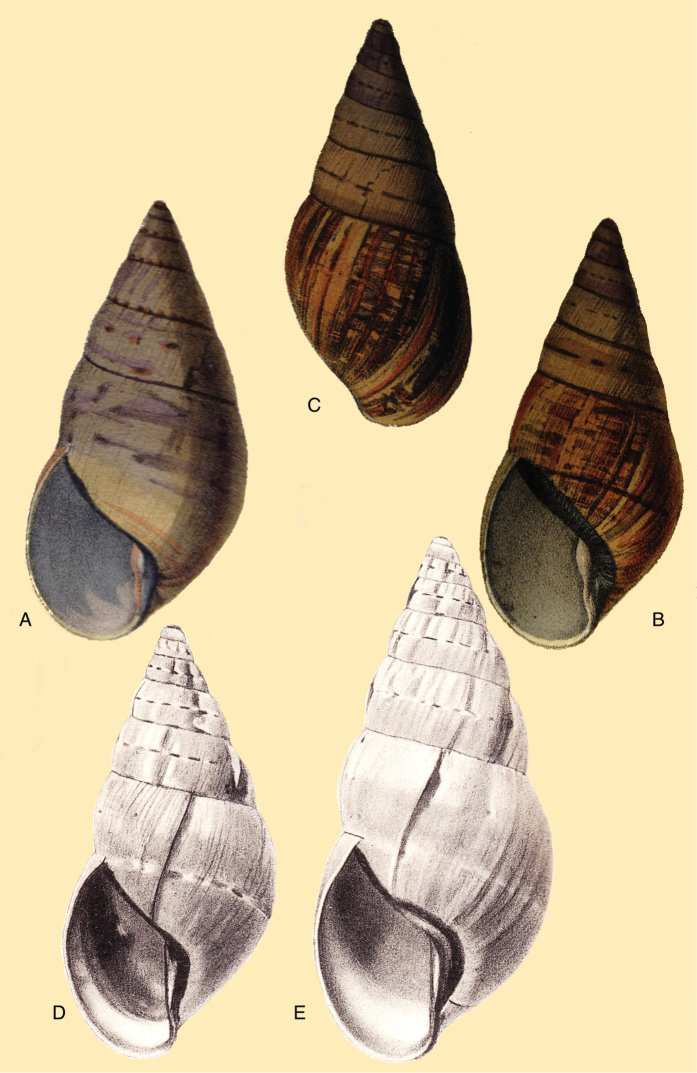
*Corona* species. **A–C** *Corona regalis* (Hupé, 1857) **A** original figure [Hupé 1857: pl. 10 fig. 3] (H = 70) **B–C** original figure of *Bulimus loroisianus* Hupé, 1857 [pl. 10 fig. 4] (H = 64) **D–E** *Corona incisa* (Hupé, 1857); original figure of Corona incisa var. machadoensis Strebel, 1909 [pl. 27 figs 412–413] (H = 66.3 respectively 81.5).

**Figure 43.**
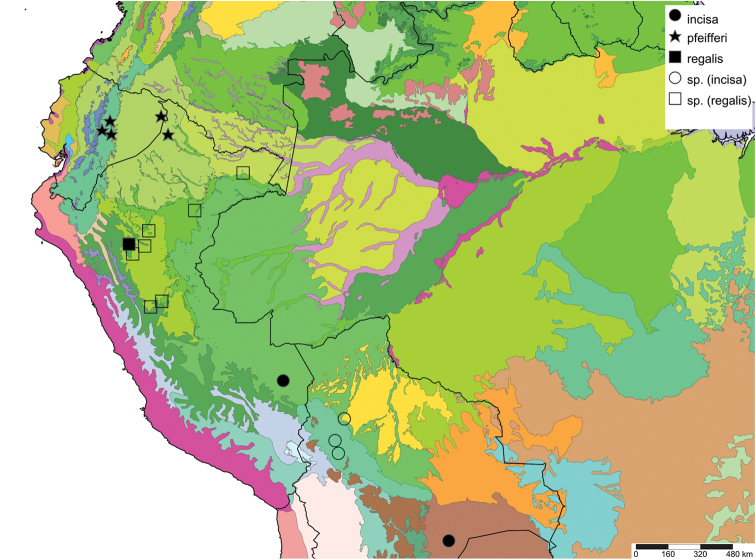
Distribution map of *Clathorthalicus* and *Corona* species. See Figure 91 and Appendix 4 for explanation of ecoregions.

**Figure 44.**
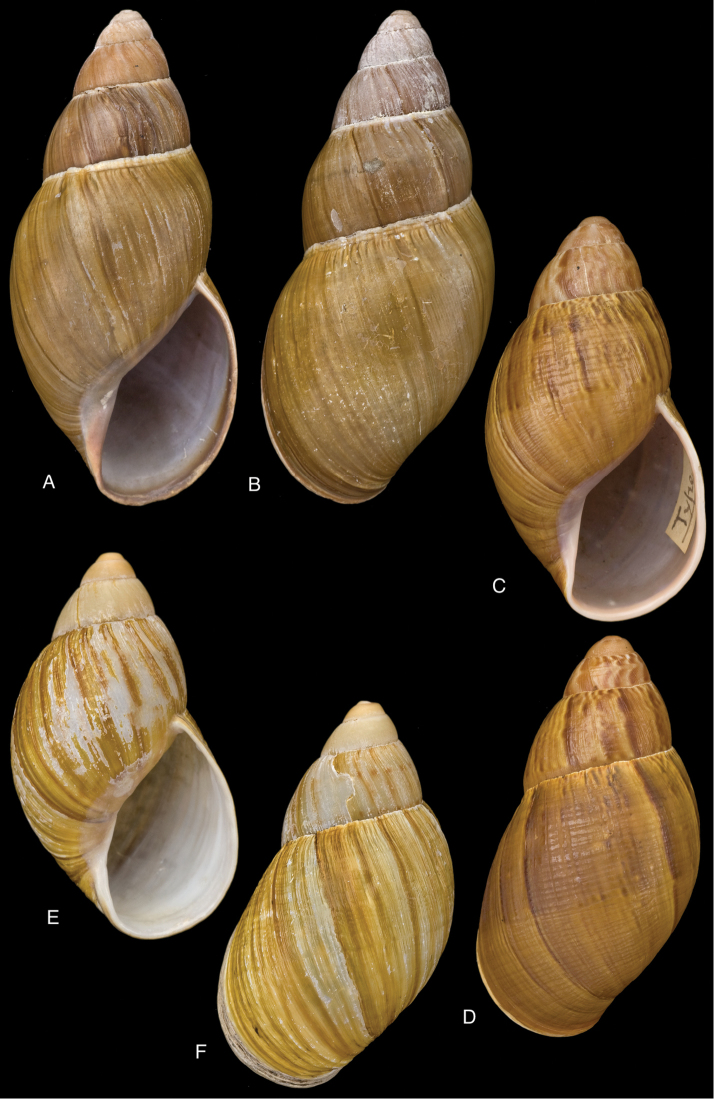
*Kara* species. **A–B** *Kara thompsonii* (Pfeiffer, 1848), lectotype NHMUK 1975464 (H = 71.0) **C–D** Kara indentatus (da Costa, 1901), lectotype NHMUK 1907.11.21.115 (H = 44.0) **E–F** *Kara yanamensis* (Morelet, 1863), paralectotype NHMUK 1893.2.4.167 (H = 48.6).

**Figure 45.**
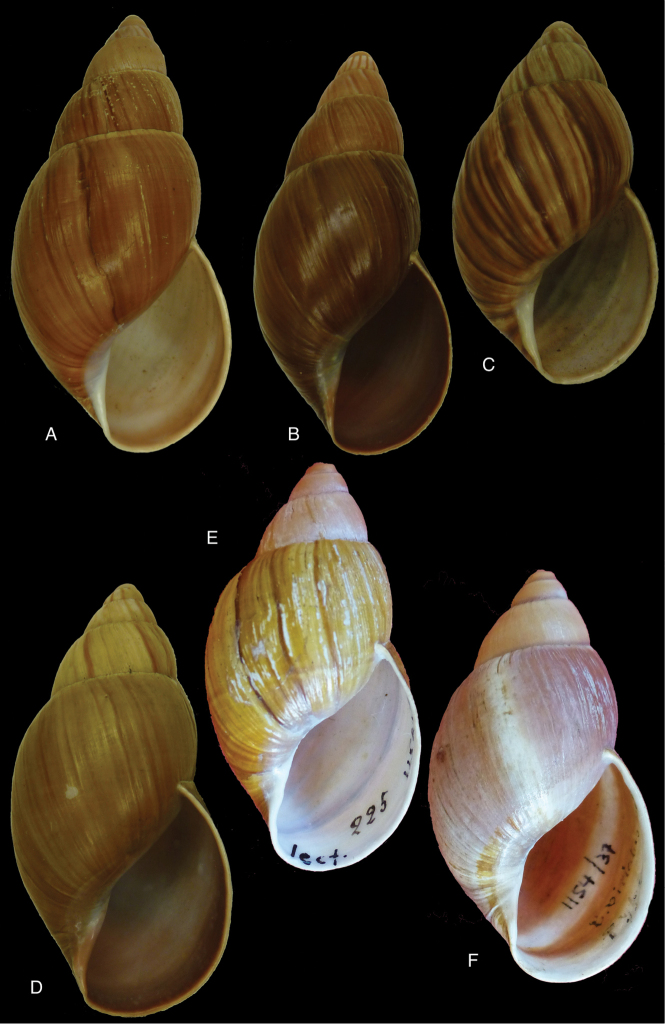
*Kara* species. **A–D** *Kara thompsonii* (Pfeiffer, 1848); **A** lectotype of Orphnus thompsoni var. lutea Cousin, 1887, RBINS/MT2358 (H=77.6) **B** lectotype of Orphnus thompsoni var. nigricans Cousin, 1887, RBINS/MT2363 (H=62.8) **C** lectotype of Orphnus thompsoni var. zebra Cousin, 1887, RBINS/MT2375 (H=46.4) **D** lectotype of Orphnus thompsoni var. olivacea Cousin, 1887, RBINS/MT2366 (H=64.5) **E** *Kara yanamensis* (Morelet, 1863), lectotype MHNG-INVE-60202 (H = 55.2) **F** *Kara viriatus* (Morelet, 1863), syntype MHNG-INVE-78772 (H = 58.7).

**Figure 46.**
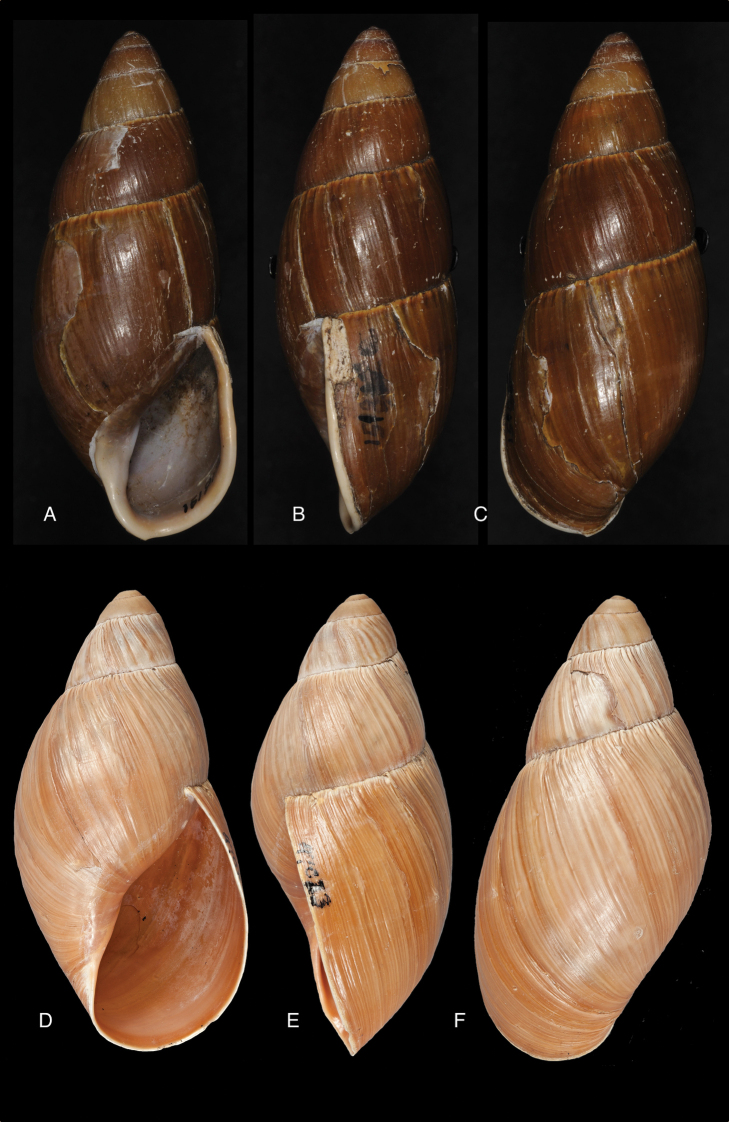
*Kara* species. **A–C** *Kara cadwaladeri* (Pilsbry, 1930), holotype ANSP 151812 (H = 70.2) **D–F** *Kara ortizianus* (Haas, 1951), holotype FMNH 47083 (H = 60.0).

**Figure 47.**
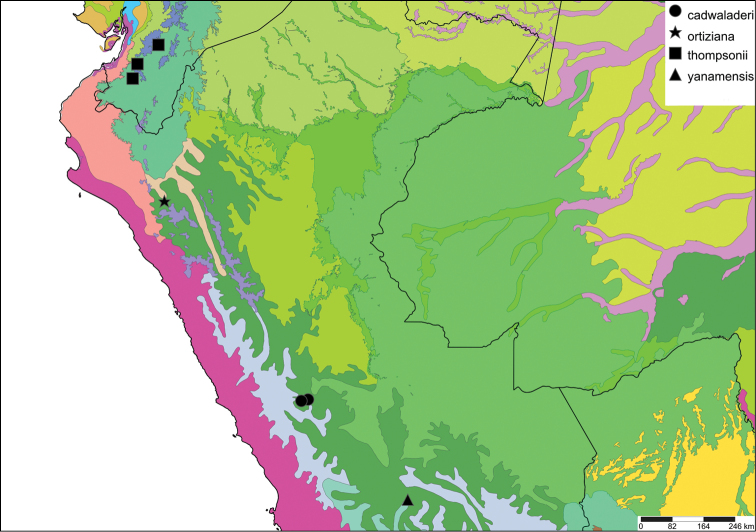
Distribution map of *Kara* species. See Figure 91 and Appendix 4 for explanation of ecoregions.

**Figure 48.**
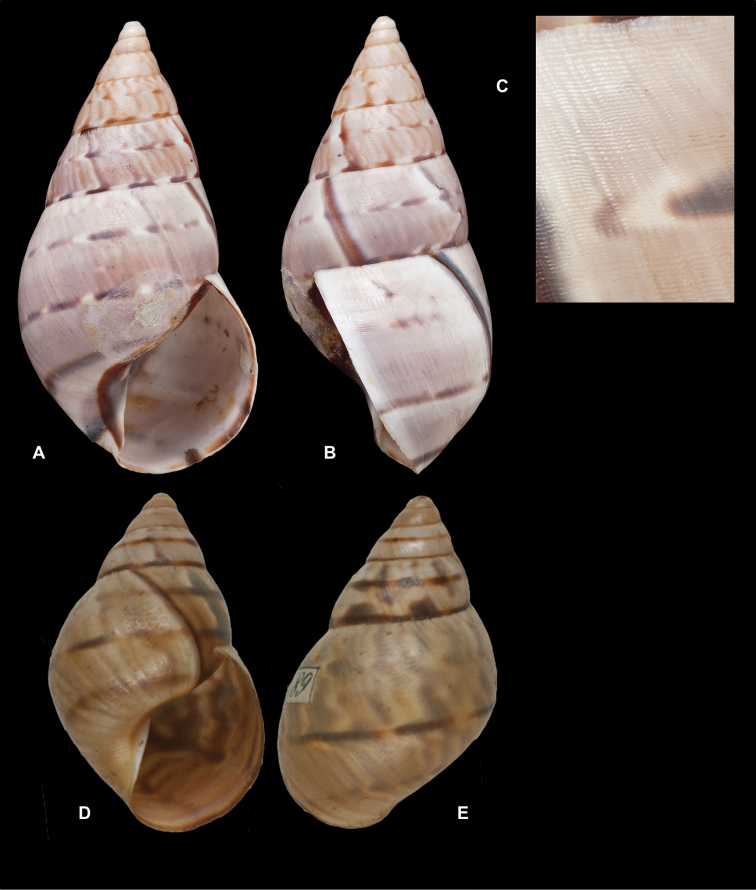
*Orthalicus* species. **A–E** Orthalicus bensoni (Reeve, 1849) **A–C** syntype of *Bulimus bensoni* Reeve NHMUK 1975582 (H = 66.6) **C** detail of sculpture on last whorl **D–E** syntype of *Orthalicus isabellinus* Martens ZMB 8876 (H = 37.0).

**Figure 49.**
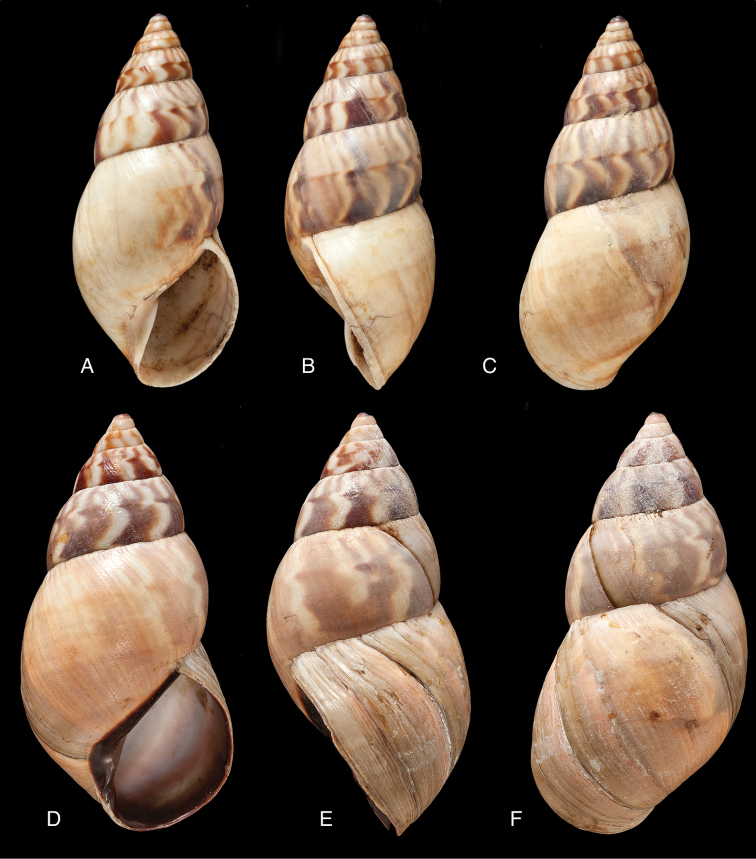
*Orthalicus* species. **A–C** *Orthalicus phlogerus* (d’Orbigny, 1835), syntype NHMUK 1854.12.4.86 (H = 59.8) **D–F** *Orthalicus mars* (Pfeiffer, 1861), syntype NHMUK 20100504 (H = 76.6).

**Figure 50.**
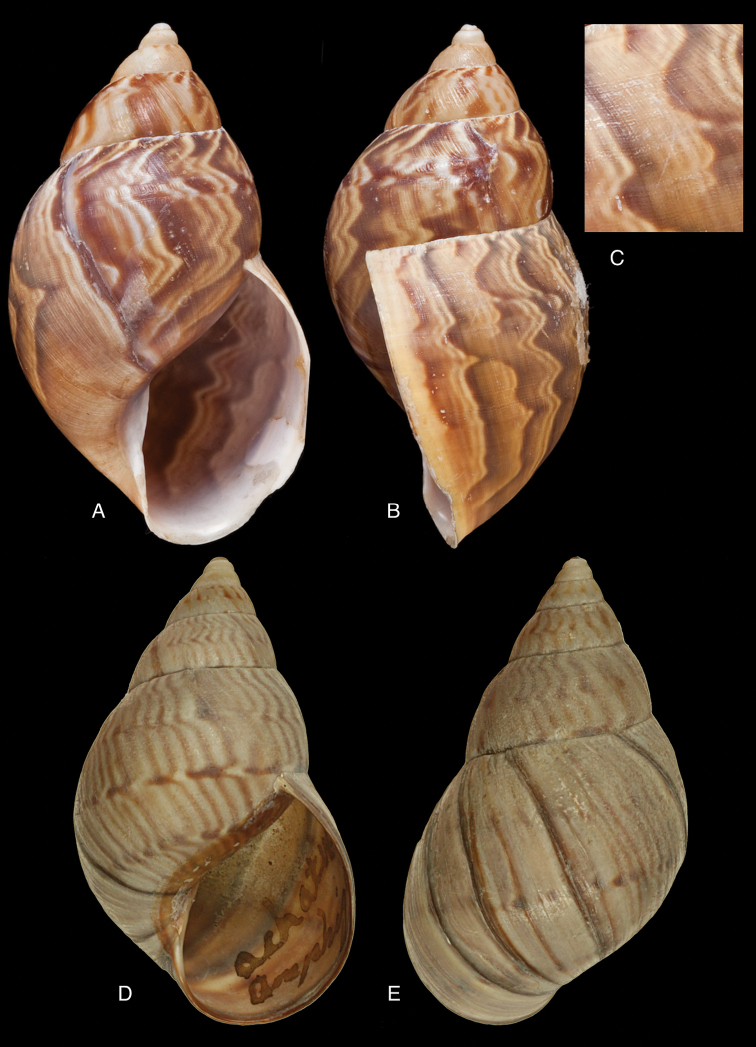
*Orthalicus* species. **A–C** *Orthalicus bifulguratus* (Reeve, 1849), lectotype NHMUK 20140082 (H = 56.9) **C** detail of sculpture on last whorl **D–E** *Orthalicus pulchellus* (Spix in Wagner, 1827), syntype ZSM 20020203 (H = 47.9).

**Figure 51.**
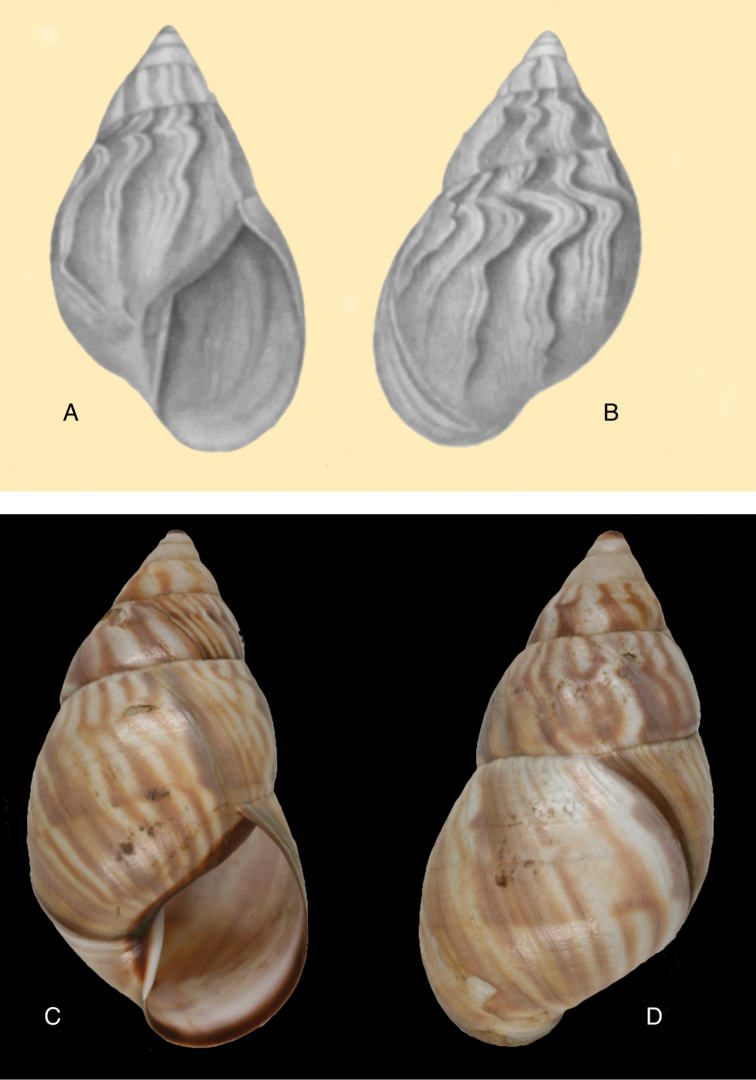
*Orthalicus* species. **A–B** *Orthalicus bifulguratus* (Reeve, 1849), original figure of *Zebra fulgur* Miller, 1878 [Miller 1879: pl. 6 fig. 1] (H = 50) **C–D** *Orthalicus maracaibensis* (Pfeiffer, 1856), holotype of *Zebra gruneri* Strebel, 1909 ZMB 117783 (H = 57.4).

**Figure 52.**
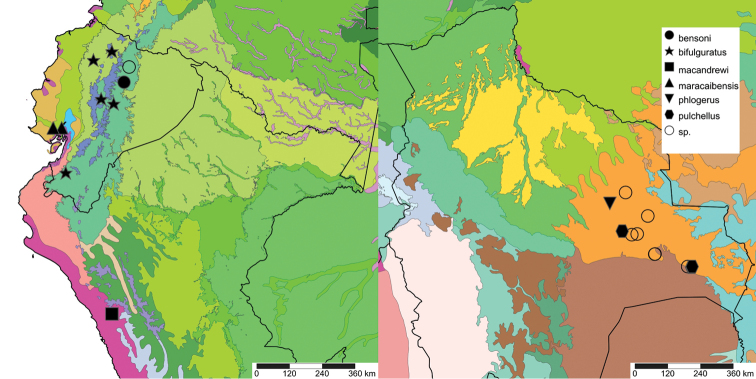
Distribution map of *Orthalicus* species. See Figure 91 and Appendix 4 for explanation of ecoregions.

**Figure 53.**
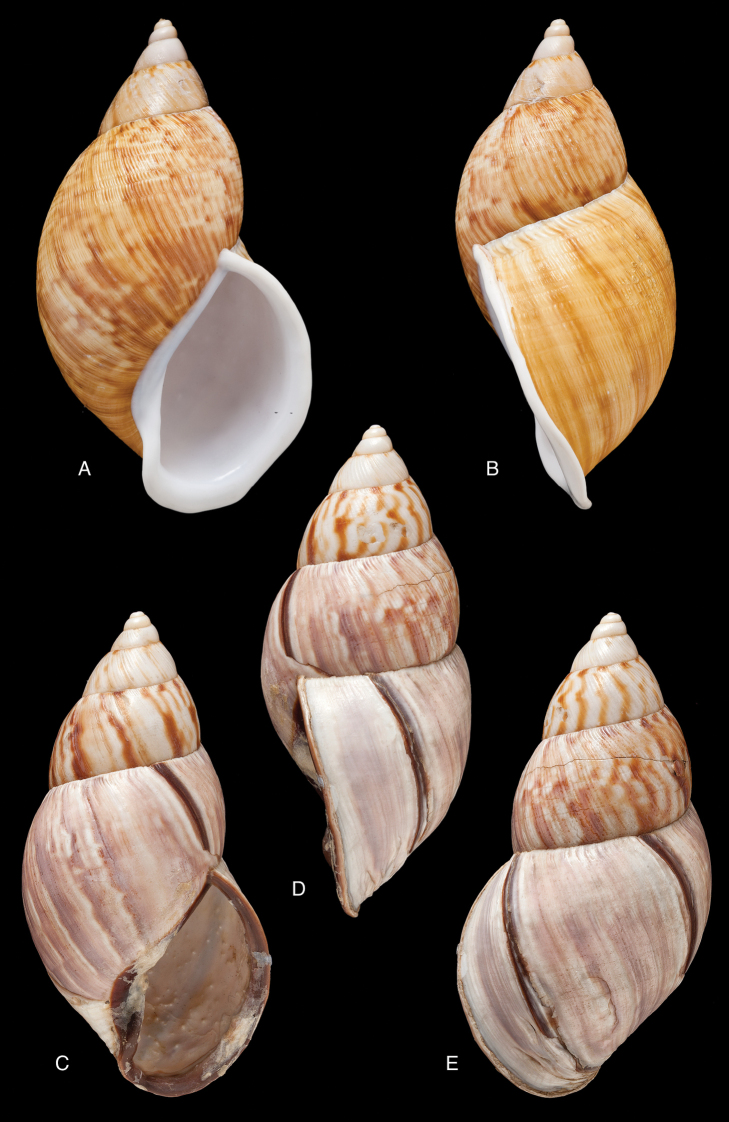
Porphyrobaphe species. **A–B** Porphyrobaphe (Oxyorthalicus) irrorata (Reeve, 1849), syntype NHMUK 1975248 (H = 77.0) **C–E** Porphyrobaphe (Porphyrobaphe) saturnus (Pfeiffer, 1860), syntype NHMUK 20140080 (H = 75.8).

**Figure 54.**
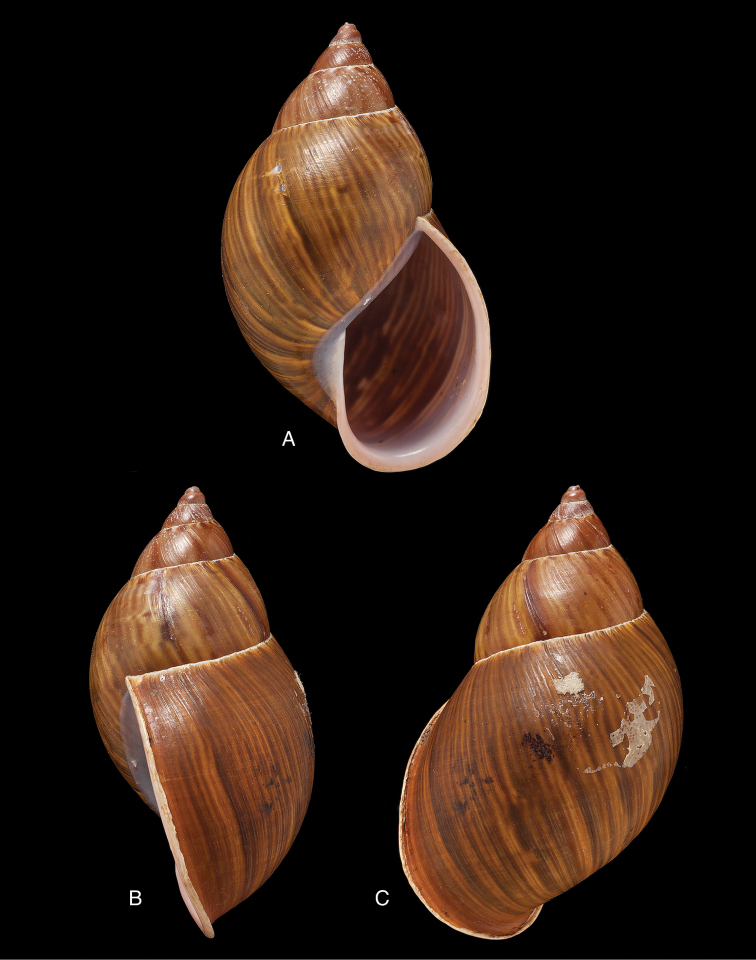
Porphyrobaphe species. **A–C** Porphyrobaphe (Oxyorthalicus) subirroratus (da Costa, 1898), lectotype NHMUK 1907.11.21.114 (H = 62.6).

**Figure 55.**
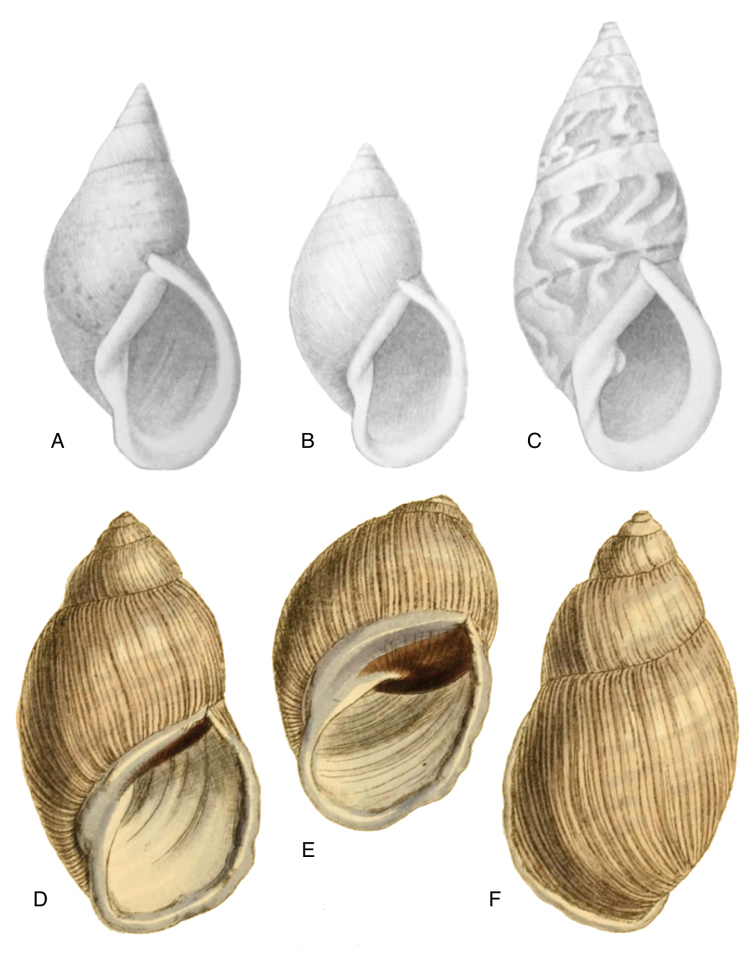
Porphyrobaphe and Sultana species. **A–B** Plekocheilus (Plekocheilus) irrorata (Reeve, 1849) **A** original figure of Dryptus irroratus var. elongatus Miller, 1878, Miller 1879: pl. 2 fig. 2a (H = 75) **B** original figure of Dryptus irroratus var. minor Miller, 1878, Miller1879: pl. 2 fig. 2b (H = 58) **C** Sultana (Metorthalicus) deburghiae (Reeve, 1859); original figure of Porphyrobaphe gloriosus var. elongatus Miller, 1878, Miller 1879: pl. 5 fig. 1 (H = 90) **D–F** Porphyrobaphe (Porphyrobaphe) iostoma (Sowerby I, 1824), original figure Sowerby I 1824: pl. 5 fig. 1 (H = 60.3).

**Figure 56.**
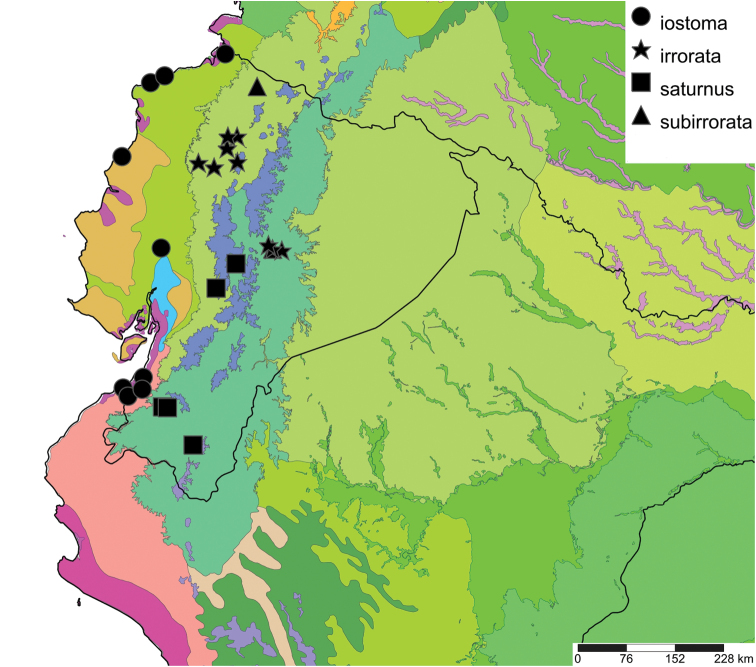
Distribution map of Porphyrobaphe species. See Figure 91 and Appendix 4 for explanation of ecoregions.

**Figure 57.**
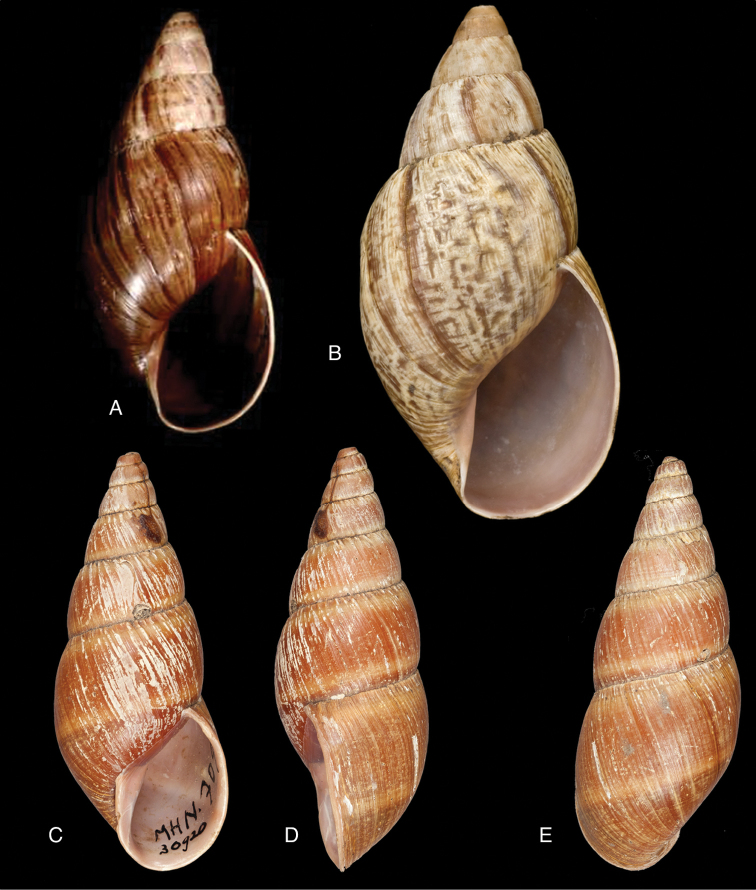
Quechua and Scholvienia species. **A** *Quechua taulisensis* Zilch, 1953, holotype SMF 111465 (H = 60.0) **B** *Quechua salteri* (Sowerby III, 1890), lectotype NHMUK 1907.11.21.118 (H = 69.9) **C–E** *Scholvienia bifasciata* (Philippi, 1845); holotype of Thaumastus (Quechua) tetricus Haas, 1951, FMNH 30920 (H = 52.6).

**Figure 58.**
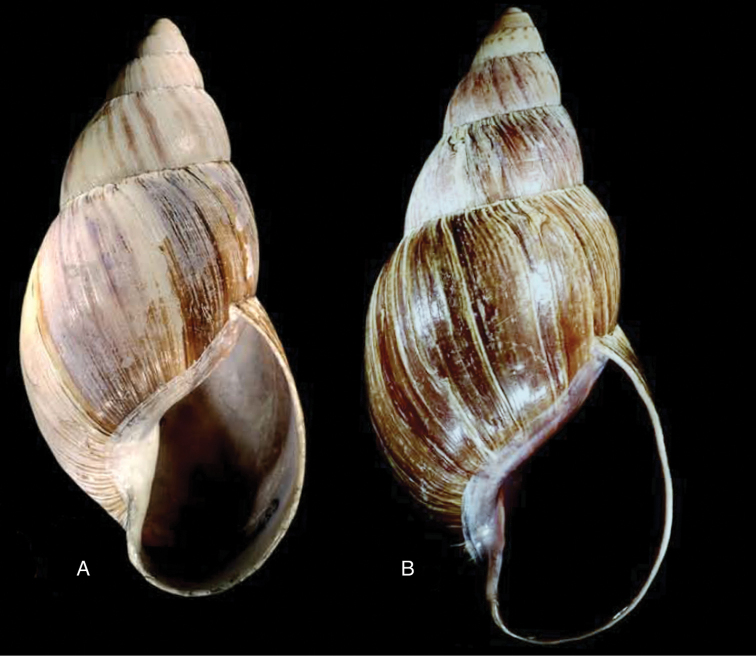
Quechua species. **A** *Quechua olmosensis* (Zilch, 1954), holotype SMF 123653 (H = 91.5) **B** *Quechua olmosensis maximus* (Weyrauch, 1967), holotype SMF 156381 (H = 99.4).

**Figure 59.**
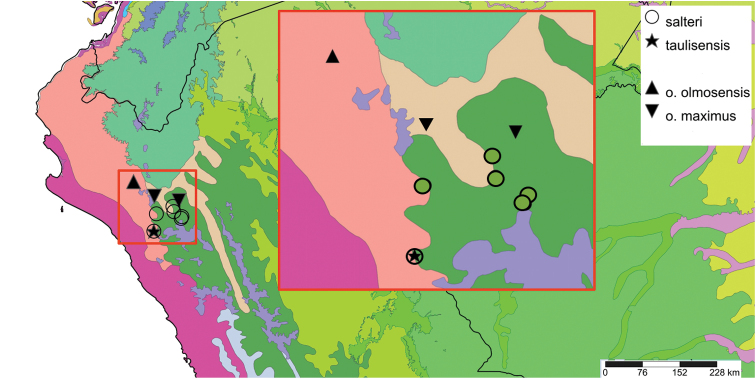
Distribution map of Quechua species. See Figure 91 and Appendix 4 for explanation of ecoregions.

**Figure 60.**
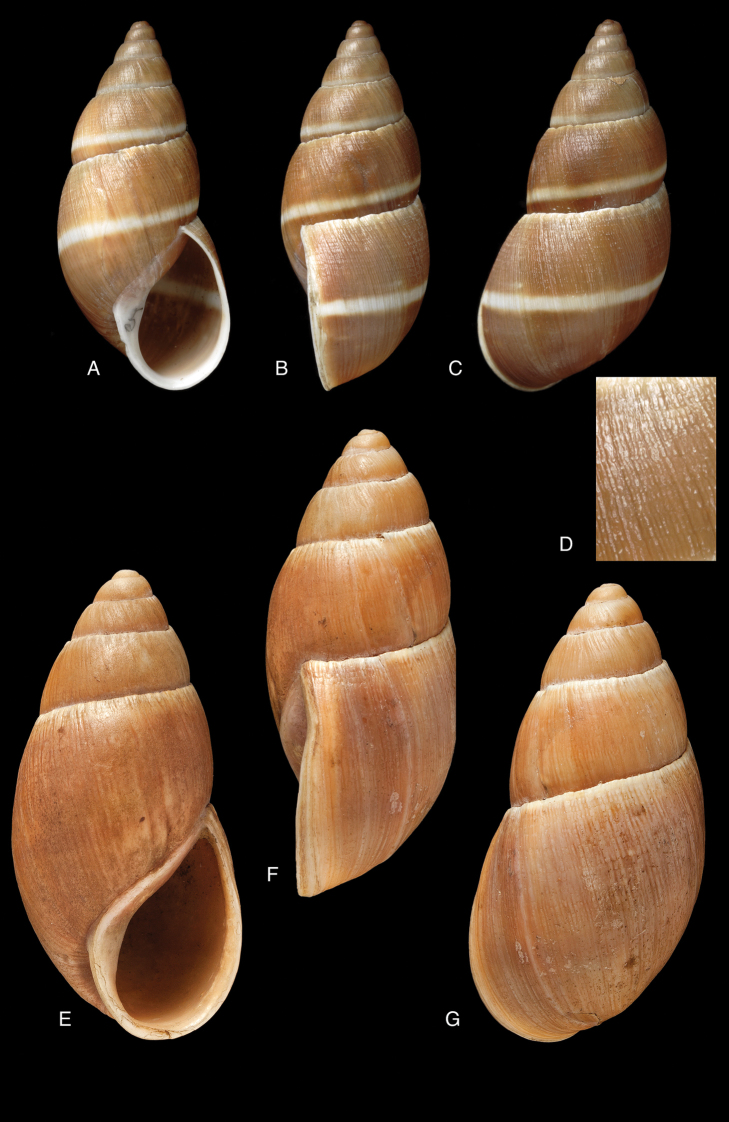
Scholvienia species. **A–D** *Scholvienia alutacea* (Reeve, 1849), lectotype NHMUK 1975148 (H = 35.5) **E–G** *Scholvienia brephoides* (d’Orbigny, 1835), lectotype NHMUK 1854.12.4.117 (H = 51.9).

**Figure 61.**
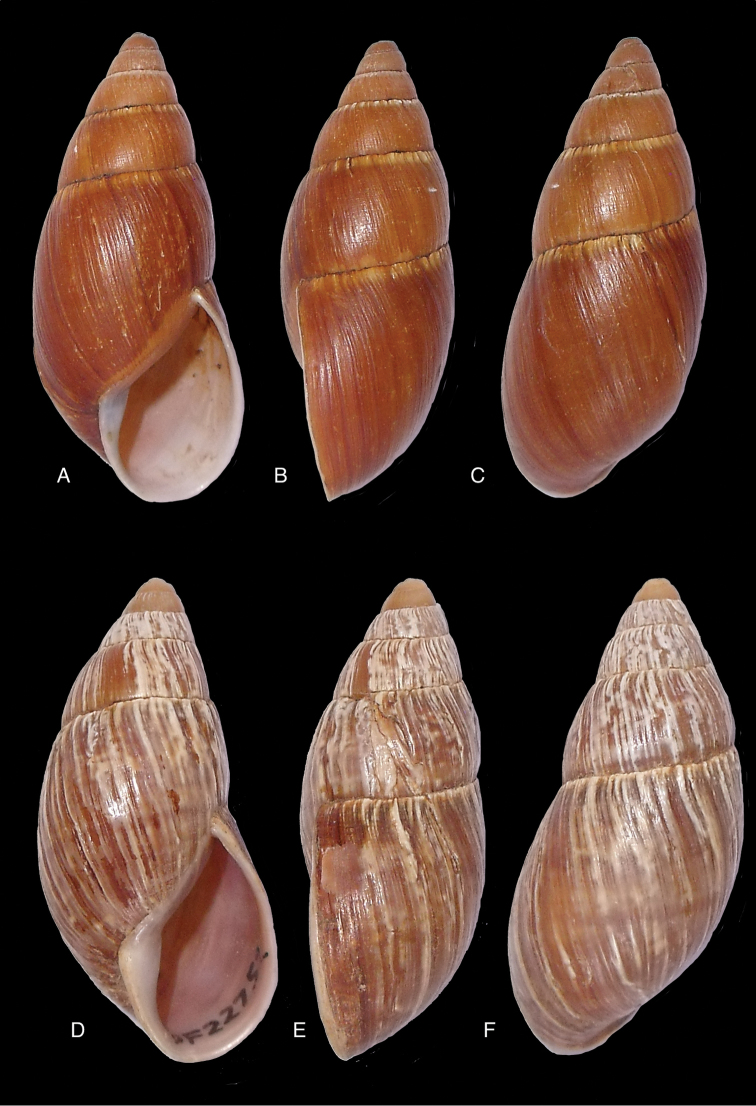
Scholvienia species. **A–C** *Scholvienia gittenbergerorum* (Breure, 1978), holotype UF 22119 (H = 41.0) **D–F** *Scholvienia bambamarcaensis* (Breure, 1978), holotype UF 22752 (H = 44.0).

**Figure 62.**
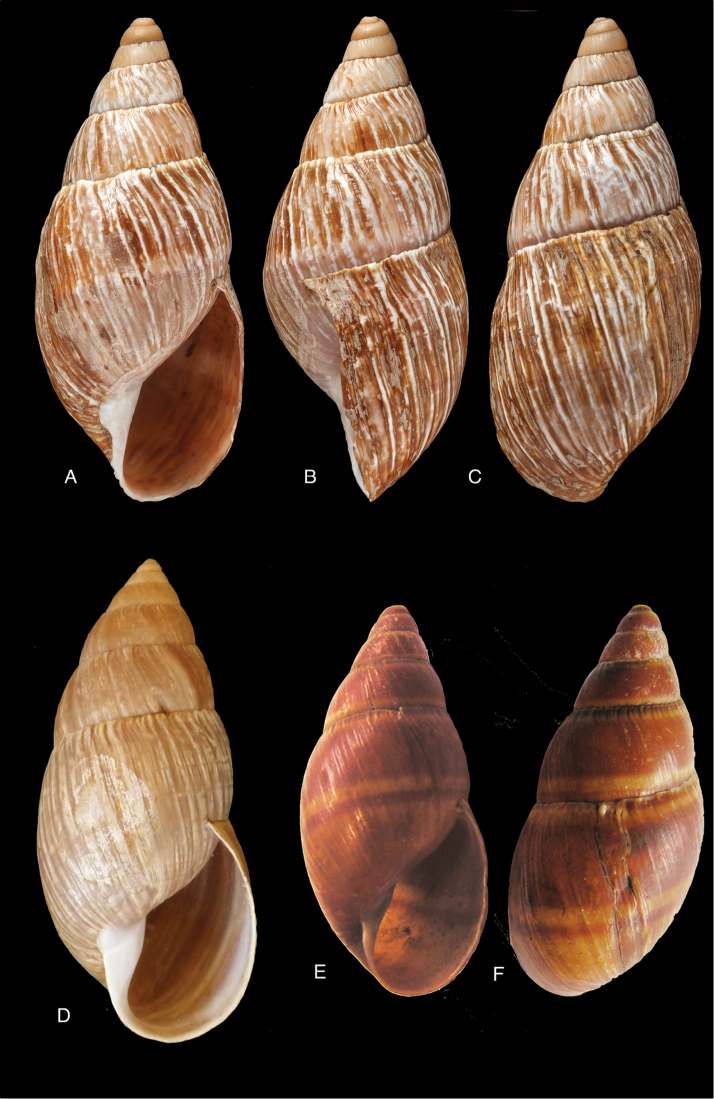
Scholvienia species. **A–C** *Scholvienia porphyria* (Pfeiffer, 1847), lectotype NHMUK 1975277 (H = 51.5) **D** *Scholvienia jaspidea* (Morelet, 1863), syntype MHNG-INVE-60211 (H = 47.2) **E–F** *Scholvienia jelskii* (Lubomirski, 1880), syntype MIZW (H = 35.0).

**Figure 63.**
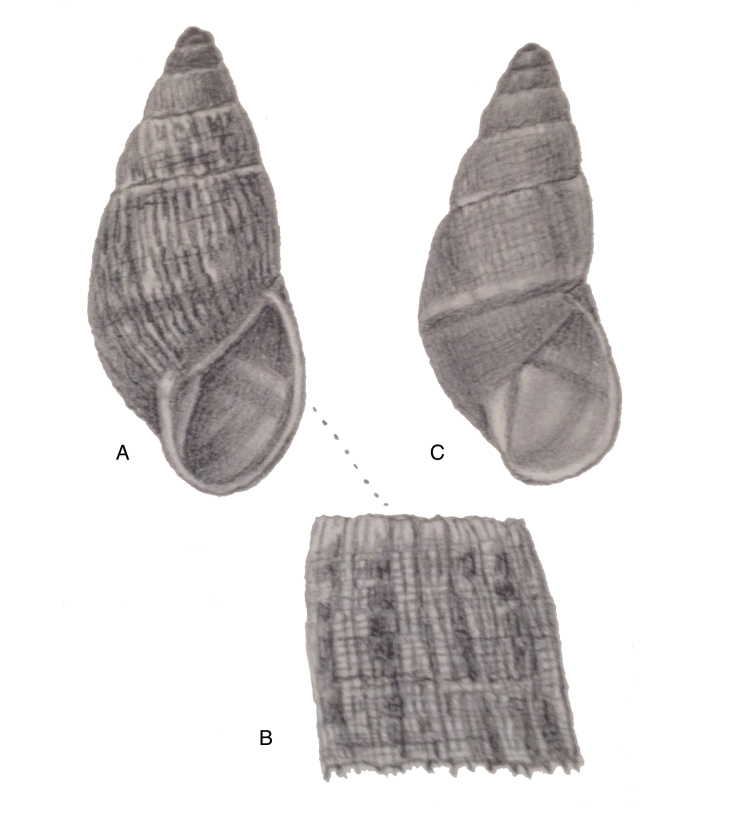
Scholvienia species. **A–C** *Scholvienia jaspidea* (Morelet, 1863), original figure of Scholvienia jaspidea forma minor Strebel, 1910: pl. 3 figs 31–32, 36 (H = 38.8 respectively 37.0).

**Figure 64.**
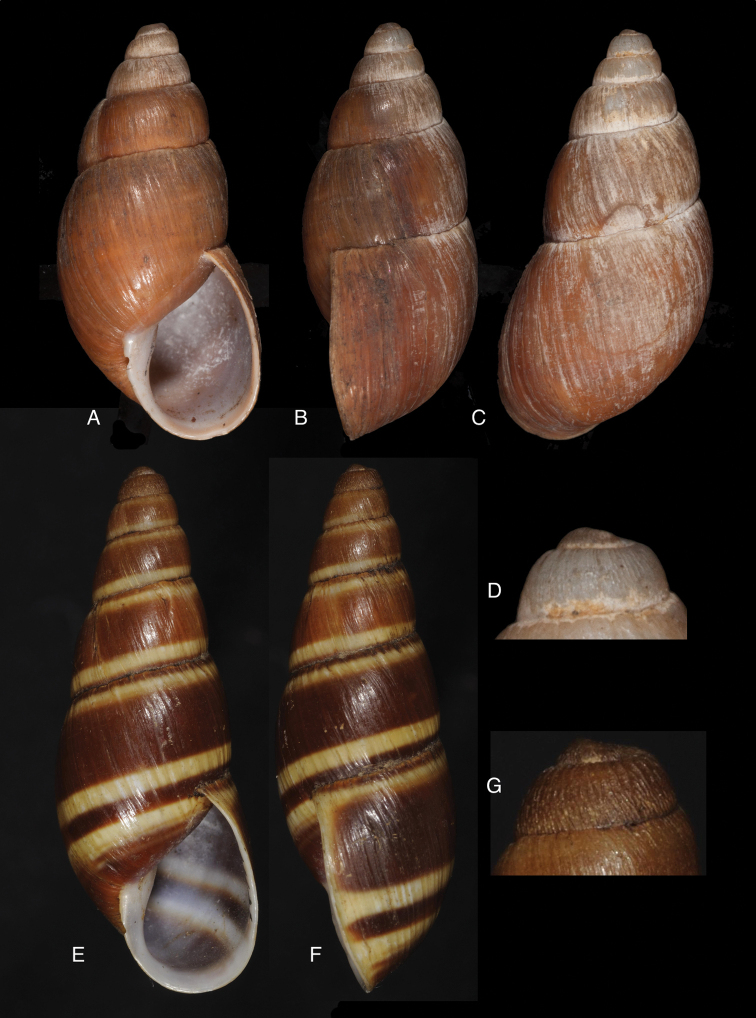
Scholvienia species. **A–D** *Scholvienia alutacea* (Reeve, 1849), holotype of Bulimulus (Protoglyptus) weeksi Pilsbry, 1930, ANSP 1402156 (H = 24.0) **D** protoconch **E–G** *Scholvienia weyrauchi* (Pilsbry, 1944), holotype 179996 (H = 39.5) **G** protoconch.

**Figure 65.**
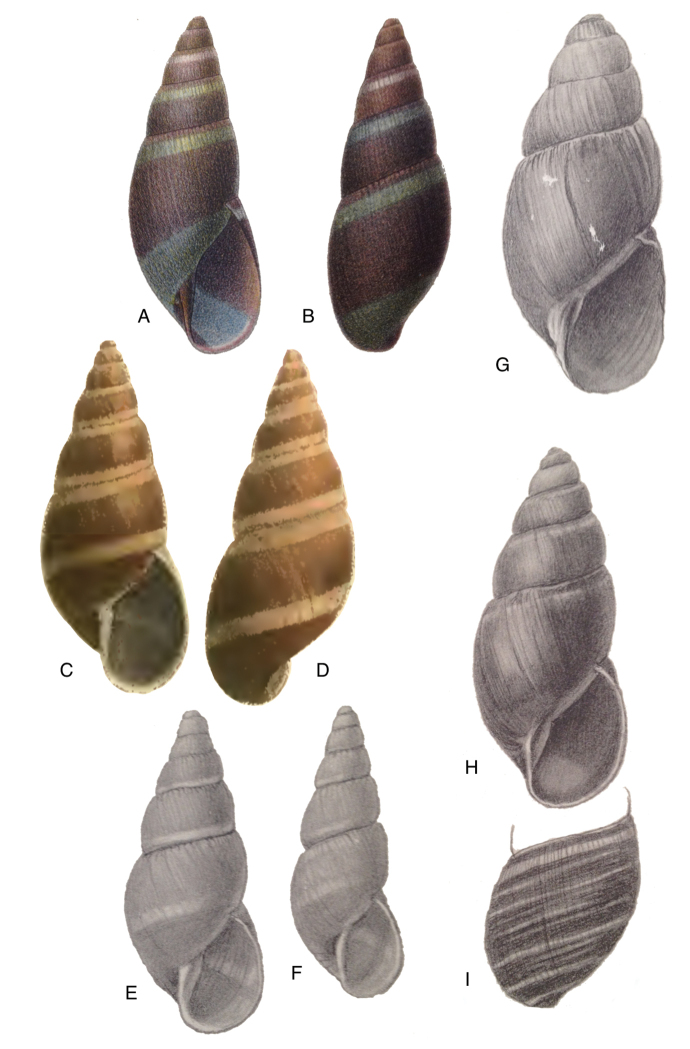
Scholvienia species. **A–B** *Scholvienia iserni* (Philippi, 1867), original figure [pl. 80 figs 16–17] (H = 53) **C–F** *Scholvienia bifasciata* (Philippi, 1845) **C–D** original figure [pl. 3 fig. 5] (H = 50) **E–F** original figure of Thaumastus (Scholvienia) bitaeniatus pallida Strebel, 1910 [pl. 3 figs 29–30] (H = 47.8 respectively 42.7) **G** *Scholvienia claritae* Strebel, 1910, original figure [pl. 2 fig. 16] (H = 61.2) **H–I** *Scholvienia huancabambensis* Strebel, 1910, original figure [pl. 2 figs 15, 19a] (H = 58.4).

**Figure 66.**
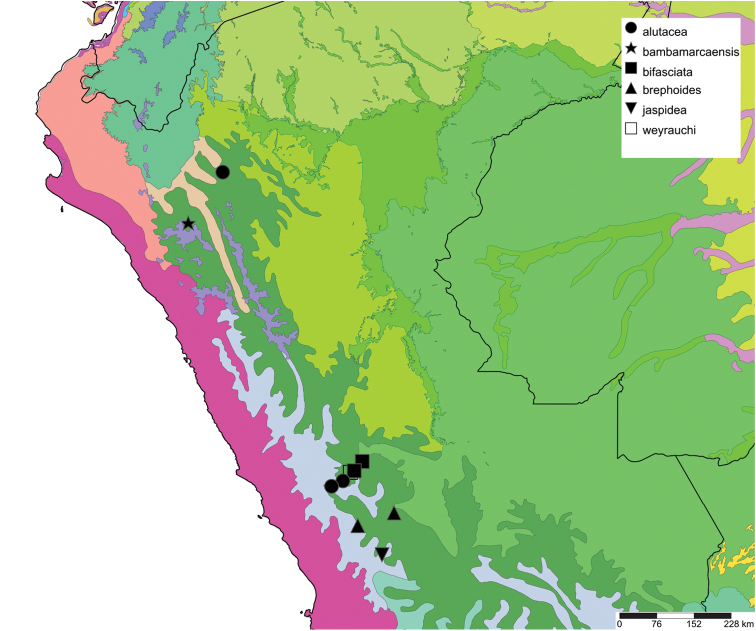
Distribution map of Scholvienia species. See Figure 91 and Appendix 4 for explanation of ecoregions.

**Figure 67.**
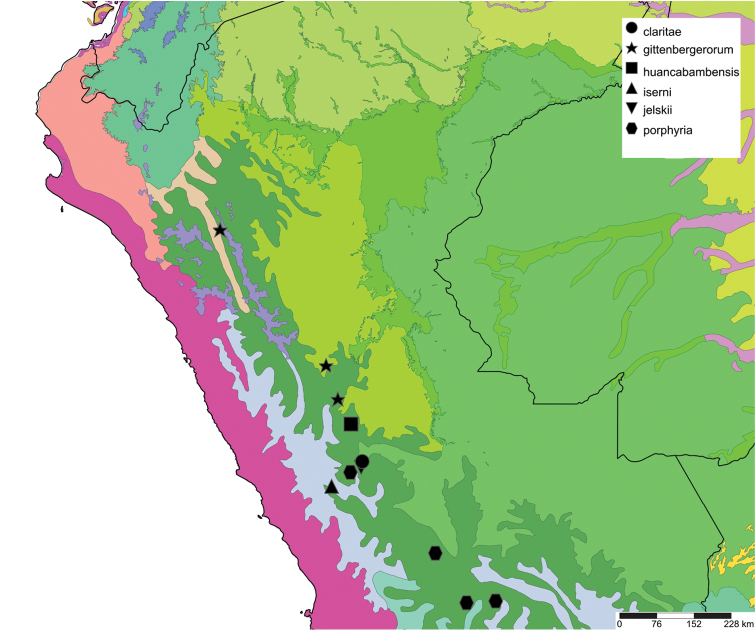
Distribution map of Scholvienia species. See Figure 91 and Appendix 4 for explanation of ecoregions.

**Figure 68.**
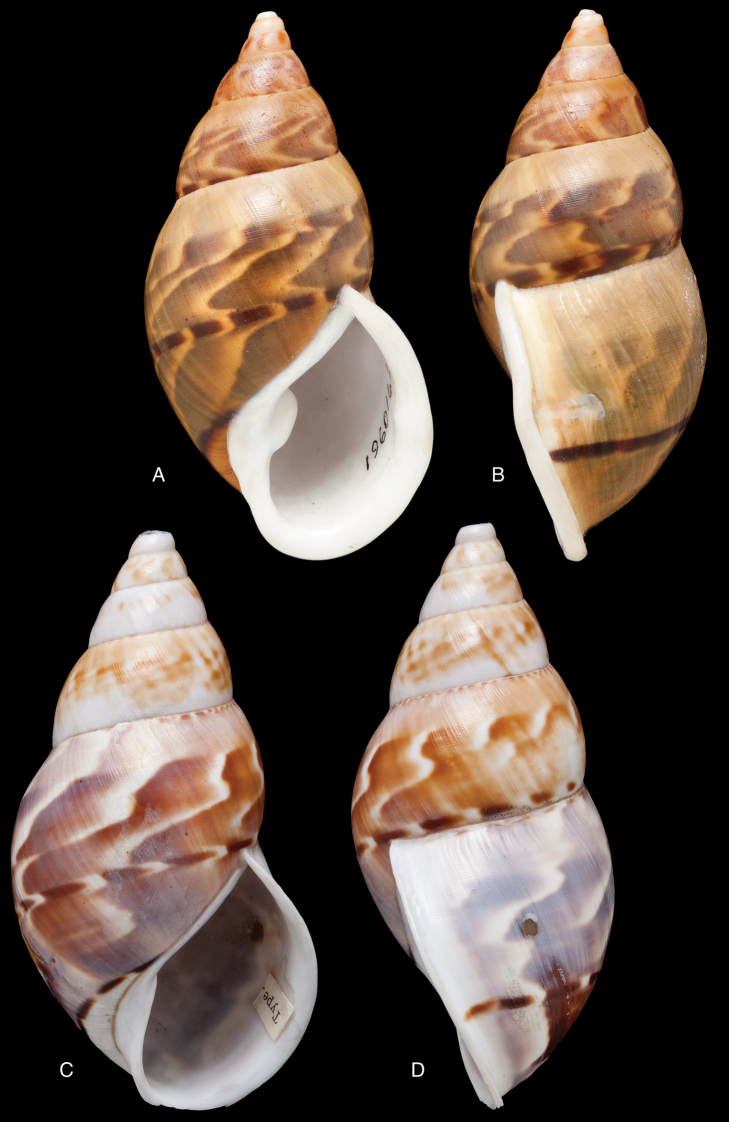
Sultana species. **A–D** Sultana (Metorthalicus) deburghiae (Reeve, 1859) **A–B** lectotype NHMUK 19601622 (H = 64.7) **C–D** lectotype of *Bulimus gloriosus* Pfeiffer, 1862 NHMUK 1975243 (H = 75.2).

**Figure 69.**
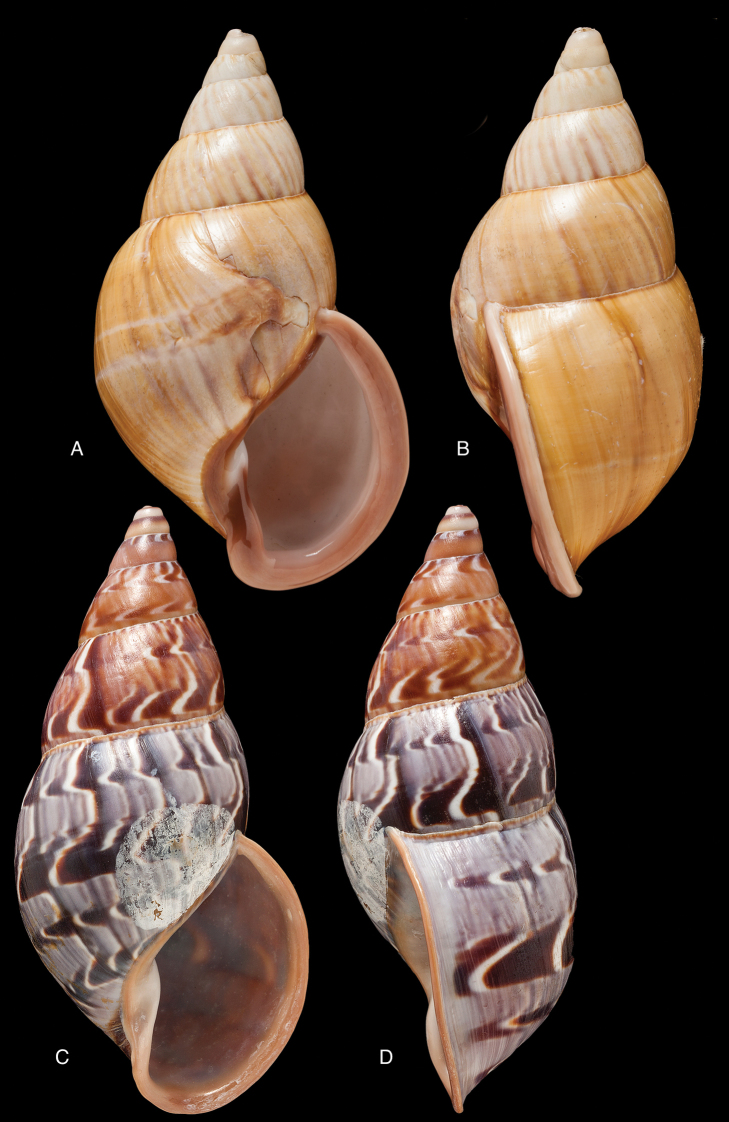
Sultana species. **A–D** Sultana (Metorthalicus) yatesi yatesi (Pfeiffer, 1855) **A–B** holotype of *Porphyrobaphe vicaria* Fulton, 1896 NHMUK 20100507 (H = 82.2) **C–D** lectotype NHMUK 1975239/1 (H 84.3).

**Figure 70.**
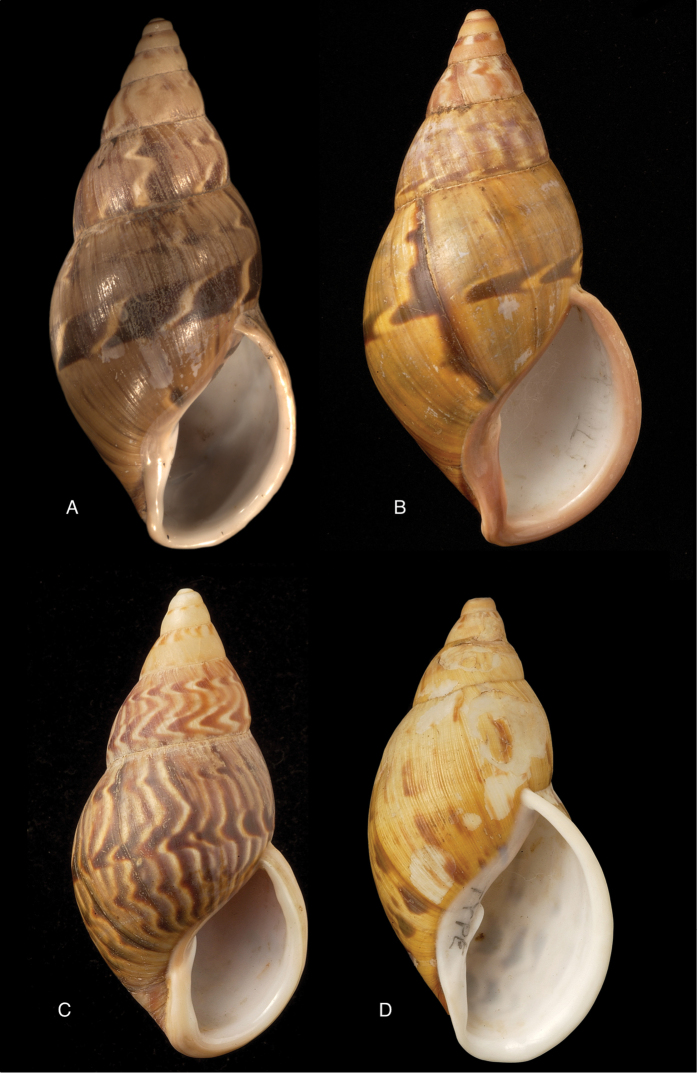
Sultana species. **A–C** Sultana (Metorthalicus) yatesi yatesi (Pfeiffer, 1855) **A** syntype of *Porphyrobaphe latevittata* Shuttleworth, 1856, NMBE 18965 (H = 80.0) **B** syntype of *Porphyrobaphe sublabeo* Ancey, 1890, NMW 1955.158.24080 (H = 82.5) **C** holotype of *Porphyrobaphe sarcostoma* Ancey, 1903, NMW 1955.158.24078 (H = 69.0) **D** Sultana (Metorthalicus) yatesi galactostoma (Ancey, 1890), syntype NMW 1955.158.24079 (H = 67.5).

**Figure 71.**
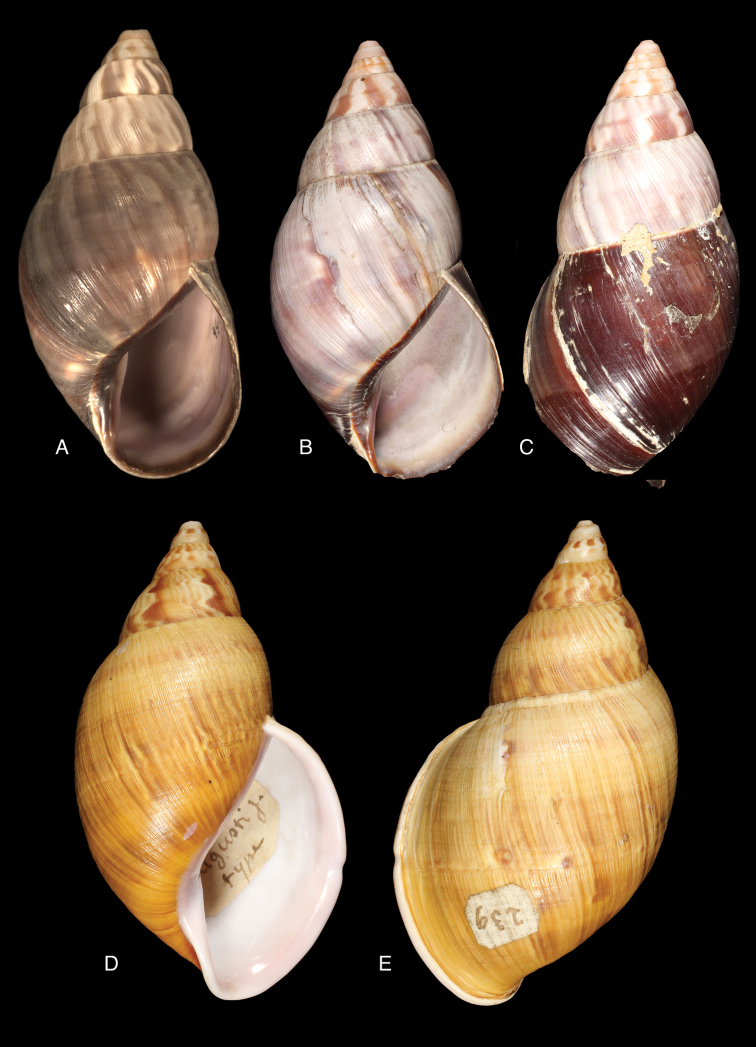
Sultana species. **A–C** Sultana (Metorthalicus) atramentaria (Pfeiffer, 1855) **A** syntype of *Orthalicus iodes* Shuttleworth, 1856, NMBE 19045 (H = 67.8) **B–C** syntype of *Bulimus boussingaultii* Hupé, 1857, MNHN 28025 (H = 65.3) **D–E** Sultana (Metorthalicus) augusti (Jousseaume, 1887), syntype MNHN 28014 (H = 68.4).

**Figure 72.**
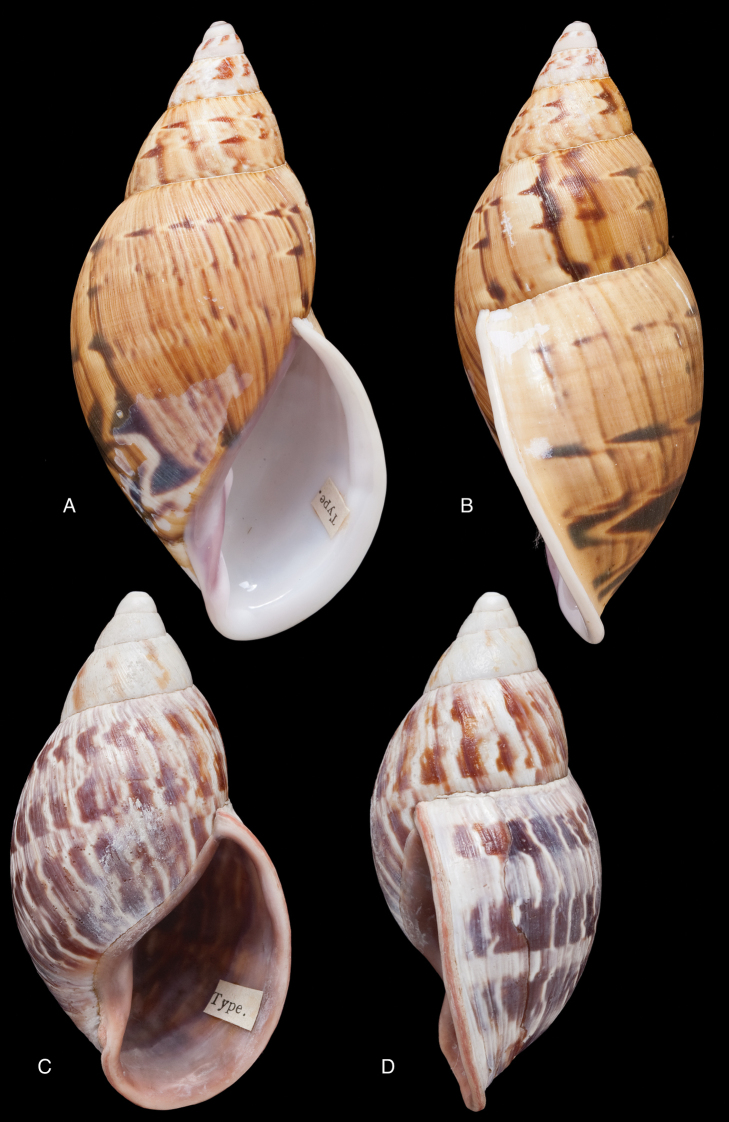
Sultana species. **A–B** Sultana (Metorthalicus) fraseri (Pfeiffer, 1858), lectotype NHMUK 20140083 (H = 88.9) **C–D** Sultana (Metorthalicus) kellettii (Reeve, 1850), lectotype NHMUK 1975241 (H = 61.2).

**Figure 73.**
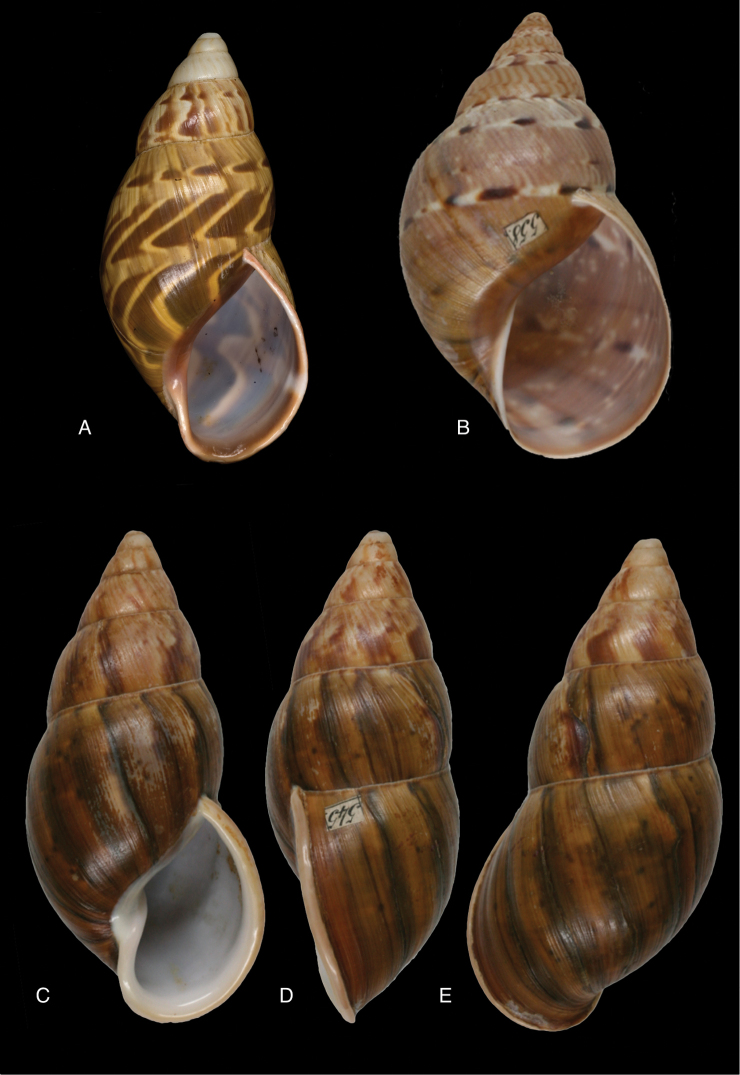
Sultana species. **A** Sultana (Metorthalicus) kellettii (Reeve, 1850), syntype of *Bulimus fungairinoi* Hidalgo, 1867, MNCN 15.05/3159 (H = 66.3) **B** Sultana (Sultana) meobambensis (Pfeiffer, 1855), holotype of *Orthalicus meobambensis carnea* Strebel, 1909 ZMB 101823 (H = 68.7) **C–E** Sultana (Metorthalicus) maranhonensis (Albers, 1854), lectotype ZMB 101825 (H = 75.6).

**Figure 74.**
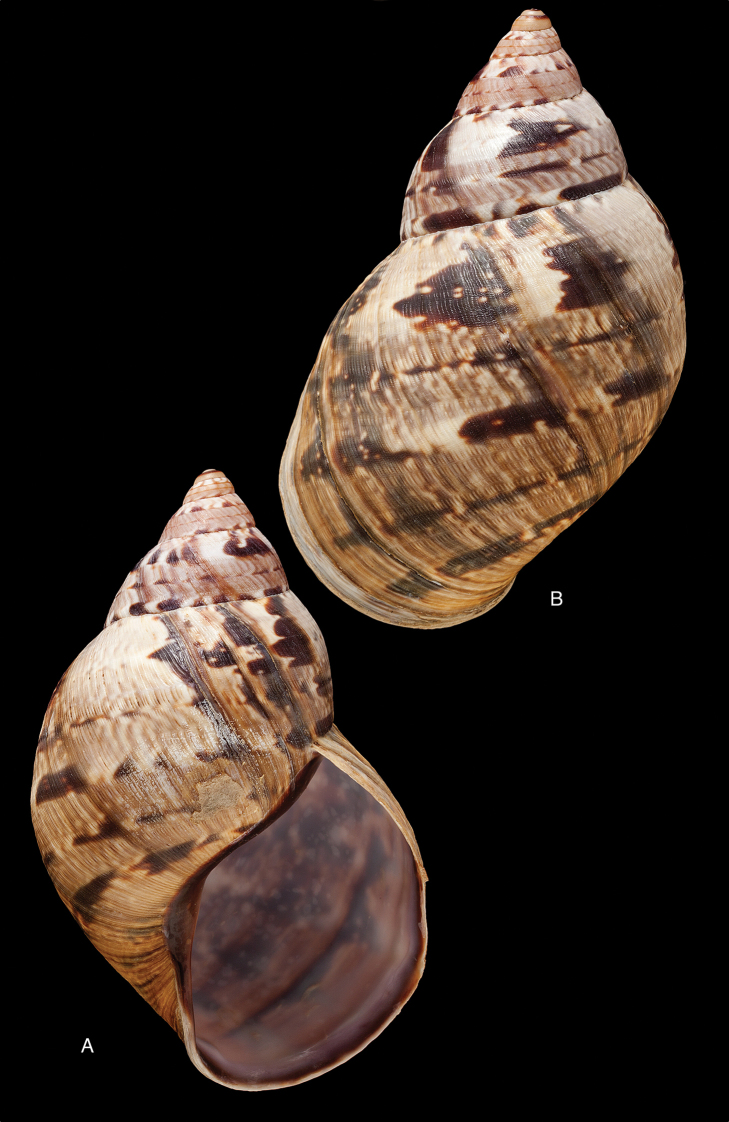
Sultana species. **A–B** Sultana (Sultana) meobambensis (Pfeiffer, 1855), syntype NHMUK 20100505 (H = 84.9).

**Figure 75.**
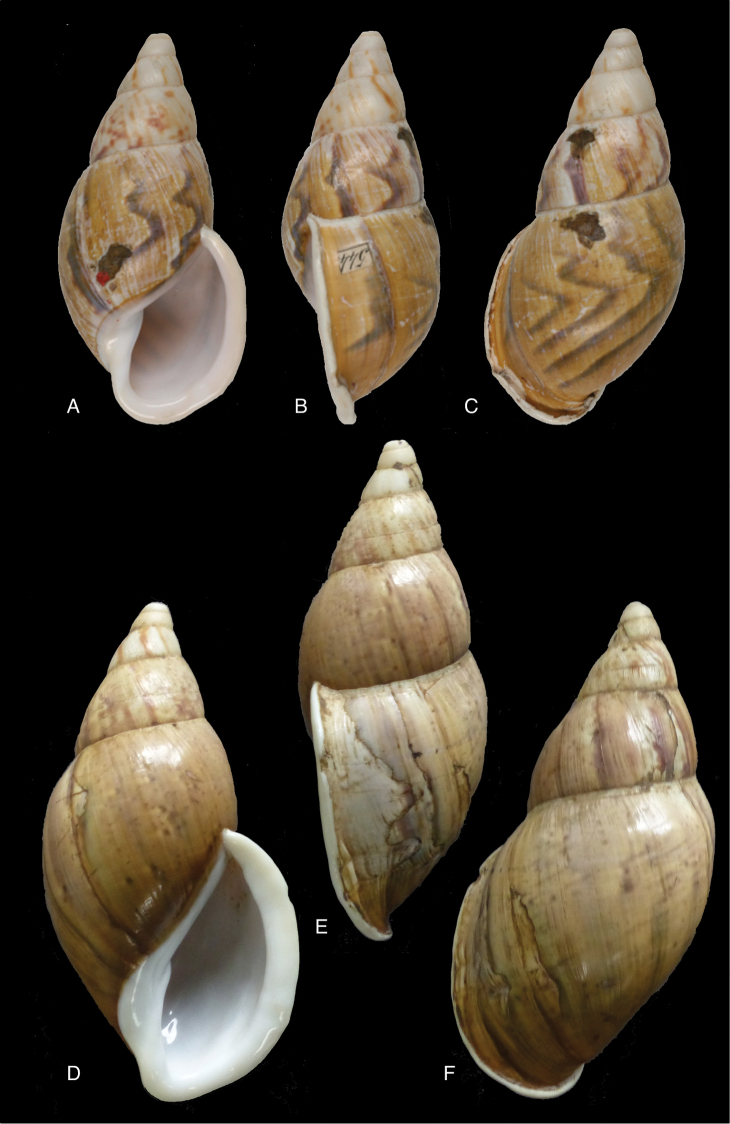
Sultana species. **A–C** Sultana (Metorthalicus) shuttleworthi (Albers, 1854), syntype ZMB 101827 (H = 70.3) **D–F** Sultana (Metorthalicus) wrzesniowskii (Lubomirski, 1880), holotype MIZW (H = 78.0).

**Figure 76.**
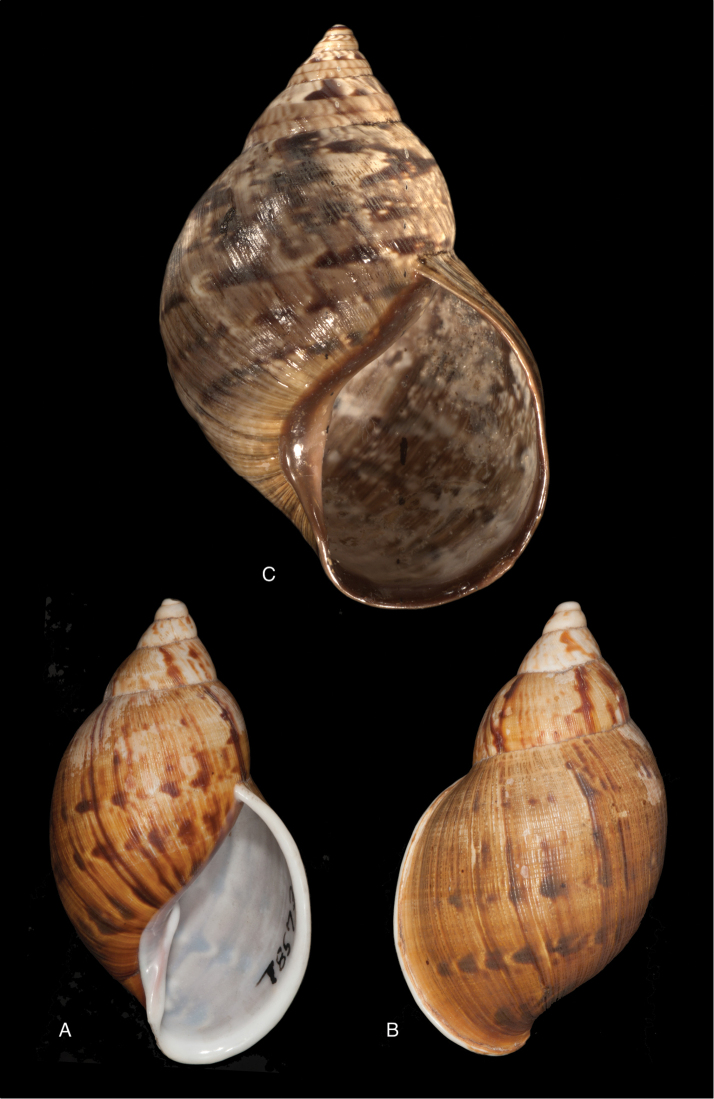
Sultana species. **A–B** Sultana (Metorthalicus) fraseri (Pfeiffer, 1858); holotype of *Orthalicus fraseri brevispira* Pilsbry, 1899, ANSP 78573 (H = 69) **C** Sultana (Sultana) sultana (Dillwyn, 1817), syntype of *Orthalicus trullisatus* Shuttleworth, 1956, NMBE 18962 (H = 87.4).

**Figure 77.**
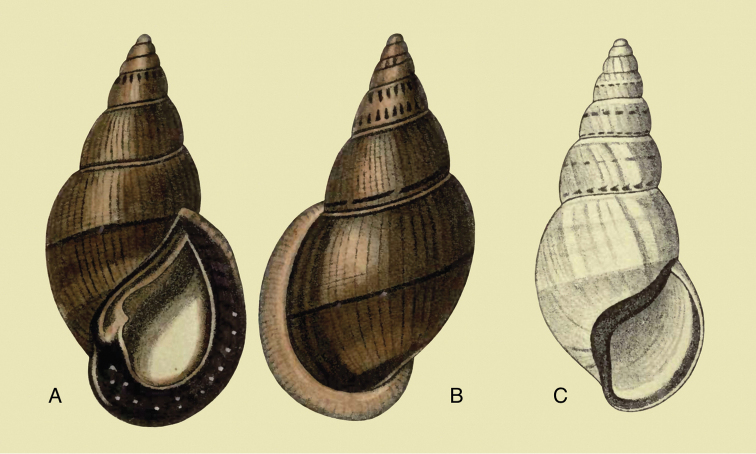
Sultana species. **A–B** Sultana (Metorthalicus) labeo (Broderip, 1828), original figure of *Bulinus labeo* Broderip 1828: suppl. 31 (H = 76.2) **C** Sultana (Metorthalicus) macandrewi (Sowerby III, 1889), original figure: pl. 25 fig. 18 (H = 70).

**Figure 78.**
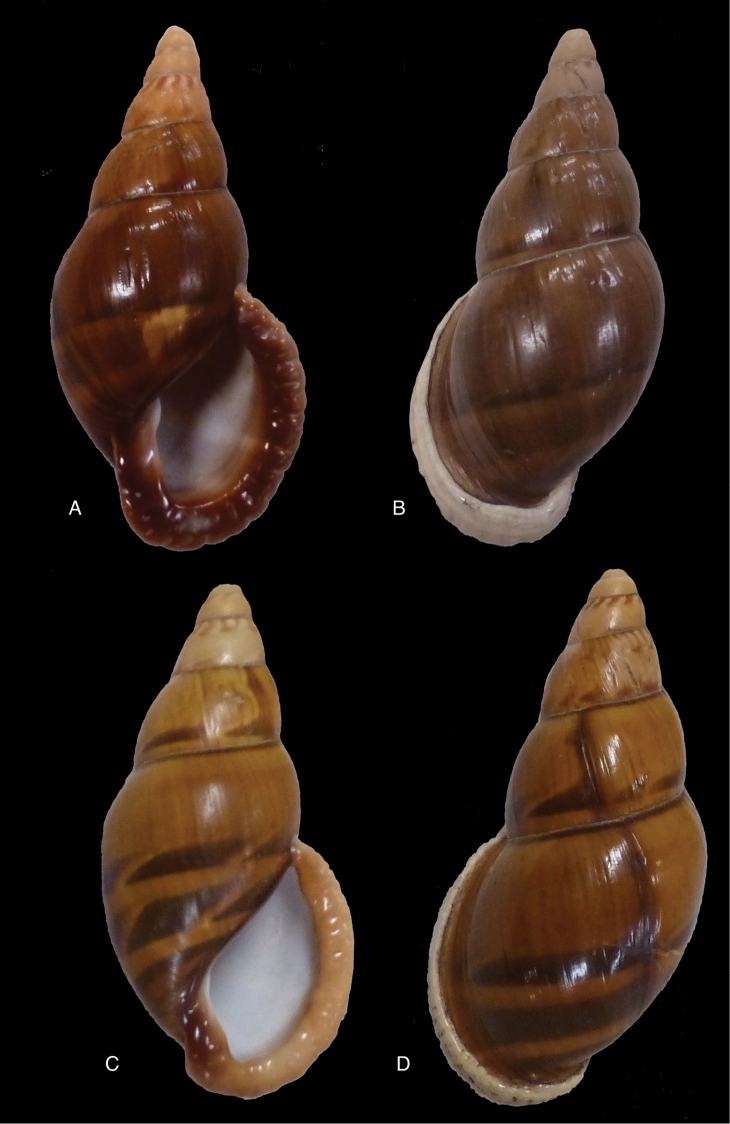
Sultana (Metorthalicus) labeo (Broderip, 1828). **A–B** NHMUK 1964473 (H = 80.2) **C–D** NHMUK 1908.6.13.101 (H = 80.7).

**Figure 79.**
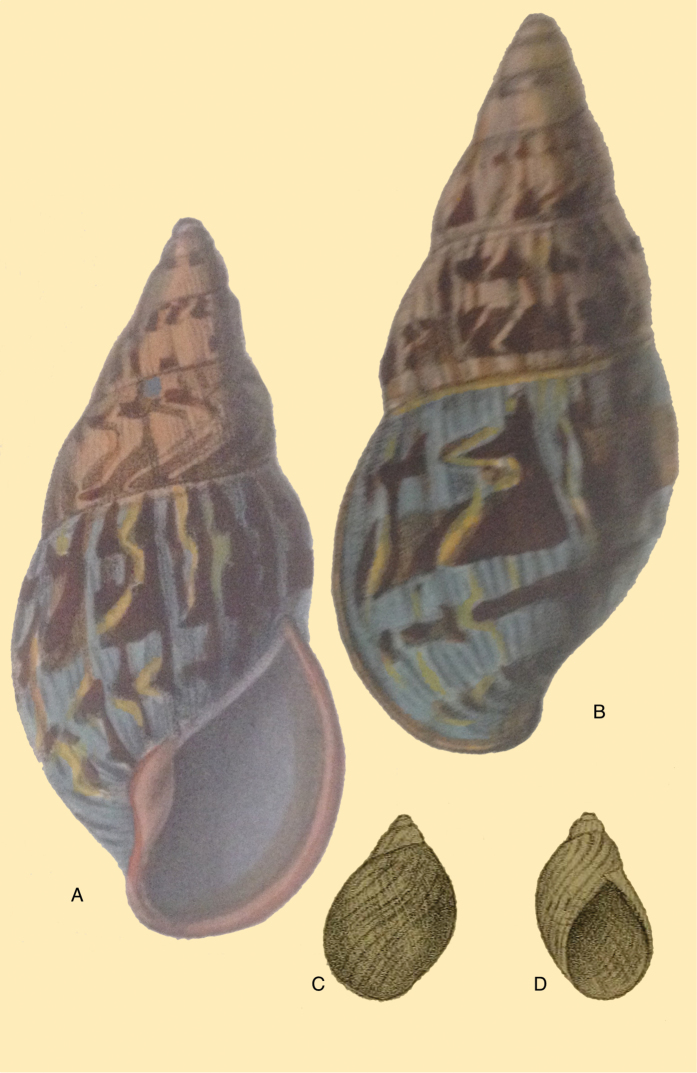
Sultana and Simpulopsis species. **A–B**Sultana (Metorthalicus) kellettii (Reeve, 1850); original figure of *Bulimus jatesi* ‘Shuttleworth’ Hupé, 1857 [pl. 8 figs 1–1a] (H = 70) **C–D**Simpulopsis (Eudioptus) citrinovitrea (S. Moricand, 1836); original figure of *Bulimus fulguratus* Miller, 1878 [Miller 1879: pl. 6 fig. 6a–b] (H = 18).

**Figure 80.**
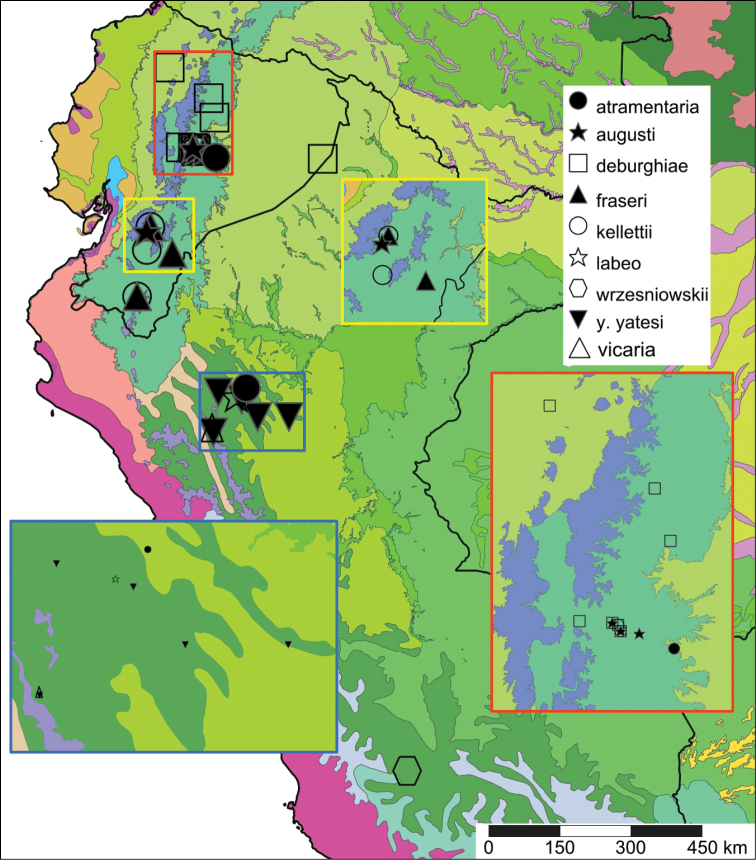
Distribution map of Sultana species. See Figure 91 and Appendix 4 for explanation of ecoregions.

**Figure 81.**
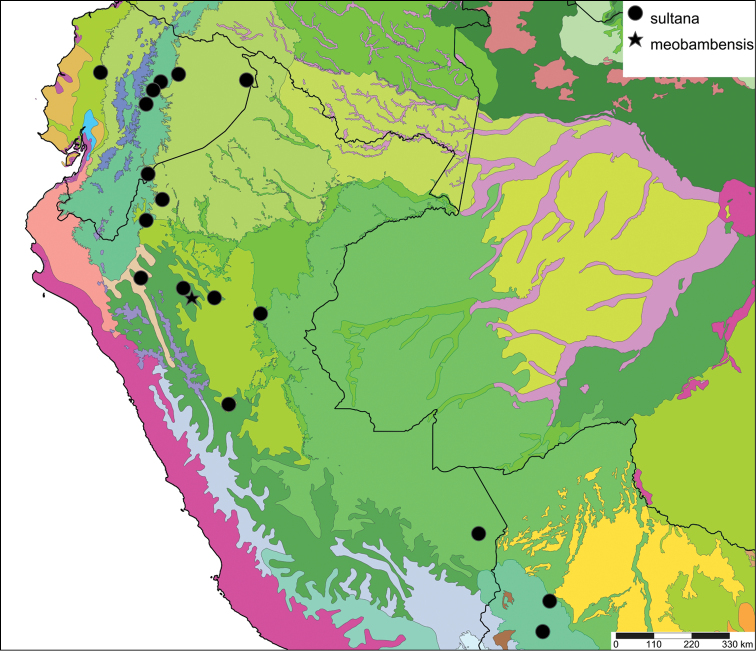
Distribution map of Sultana species. See Figure 91 and Appendix 4 for explanation of ecoregions.

**Figure 82.**
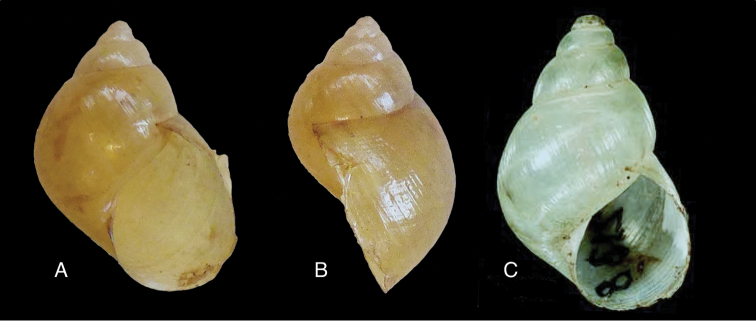
**A–C**Simpulopsis (Eudioptus) citrinovitrea (S. Moricand, 1836) **A–B** syntype MHNG-INVE-64617 (H = 16.0) **C** ‘Pseudoglandina’ agitata Weyrauch, 1967, paratype SMF 162138 (H = 16.5).

**Figure 83.**
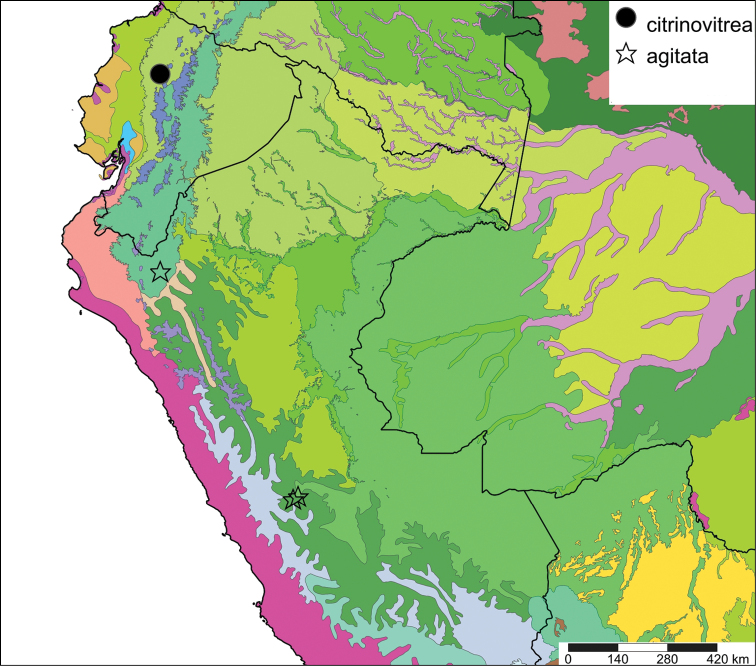
Distribution map of Simpulopsis species. See Figure 91 and Appendix 4 for explanation of ecoregions.

**Figure 84.**
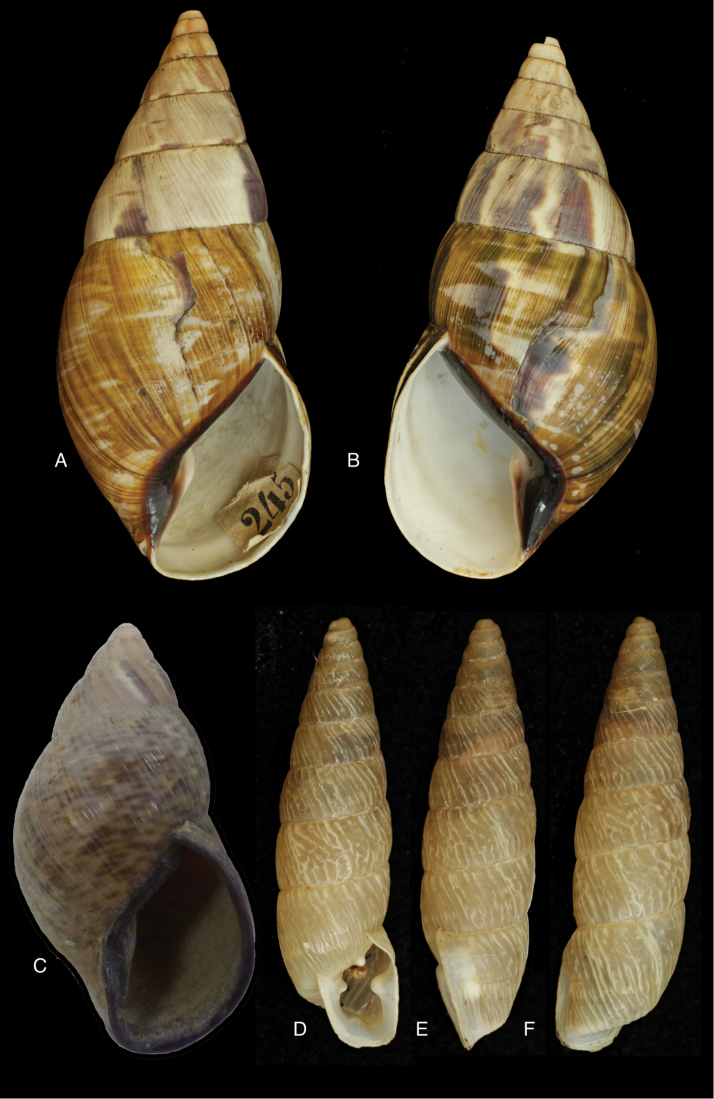
**A–B** Corona regalis (Hupé, 1857), RBINS (H = 92.0 resp. 81.9) **C**Porphyrobaphe (Porphyrobaphe) iostoma (Sowerby I, 1824), NHMUK 20150529 (H = 68.6) **D–F** Cyclodontina lemoinei (Ancey, 1892), possible syntype RBINS (H = 21.3).

**Figure 85.**
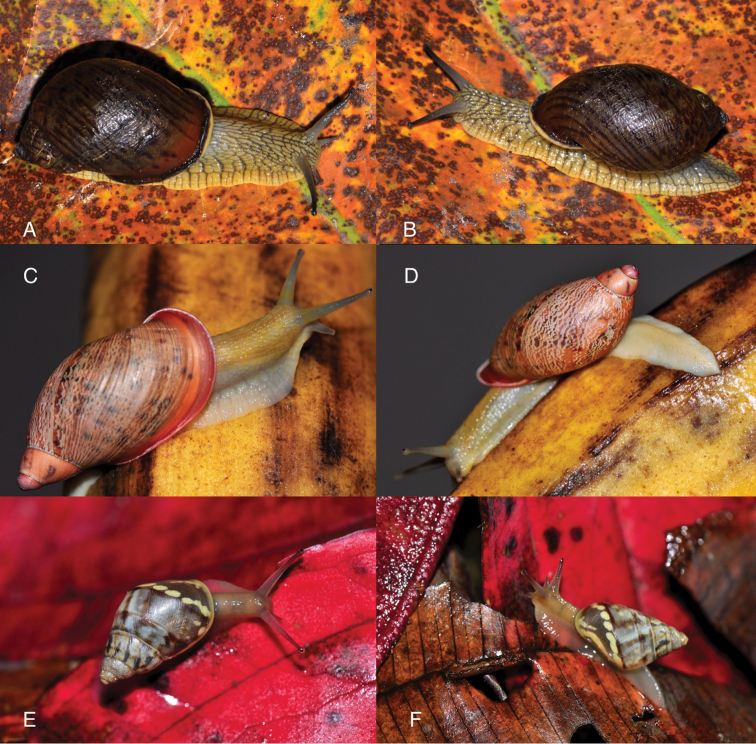
Living snails. **A–B** Plekocheilus sp., Ecuador, Prov. Carchi, near El Laurel **C–D**Plekocheilus (Aeropictus) tenuissimus Weyrauch, 1967, Ecuador, Prov. Carchi, near El Laurel **E–F** *Clathrorthalicus* sp., juvenile, Ecuador, Prov. Sucumbios, near La Bonita (all photos courtesy A. González).

**Figure 86.**
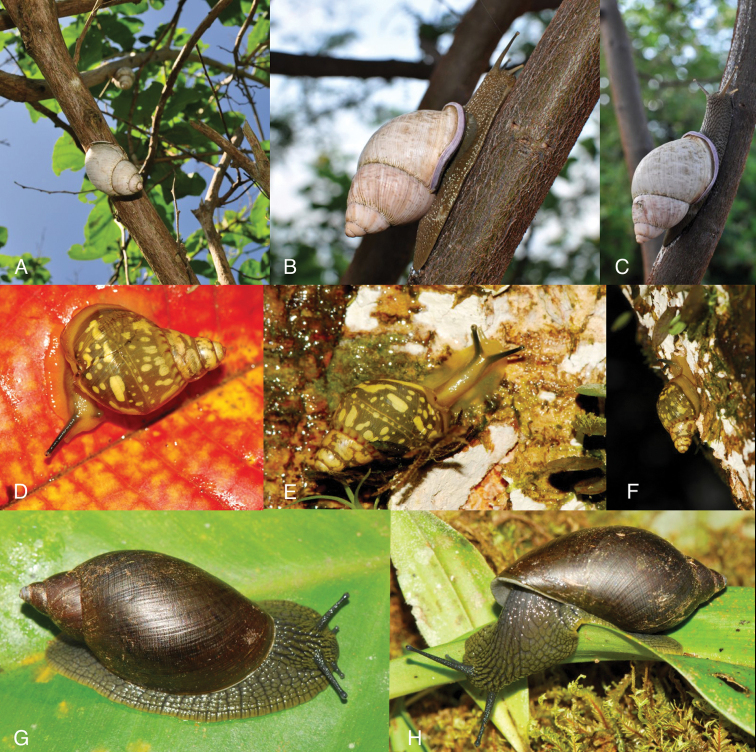
Living snails. **A–C**Porphyrobaphe (Porphyrobaphe) iostoma (Sowerby I, 1824), Ecuador, Prov. Manabí, Salango **D–F** *Clathrorthalicus* sp., juvenile, Ecuador, Prov. Imbabura, near Junín **G–H**Plekocheilus (Eurytus) sp., Ecuador, Prov. Imbabura, Chontal Alto (all photos courtesy A. González).

**Figure 87.**
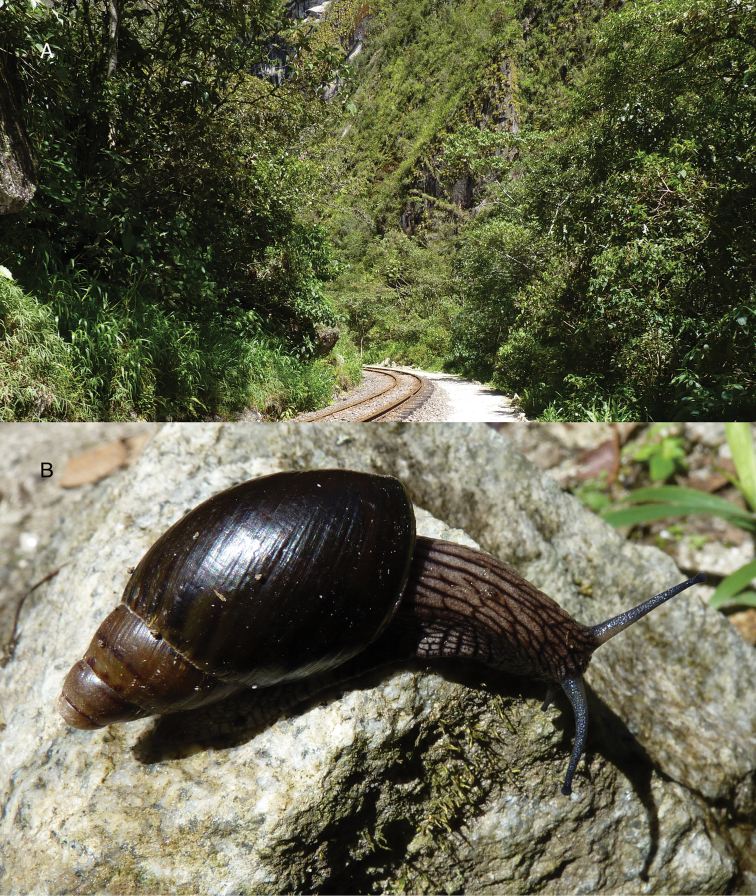
Living snails. **A–B**Thaumastus (Thaumastus) sumaqwayqu sp. n. **A** type locality **B** holotype living.

**Figure 88.**
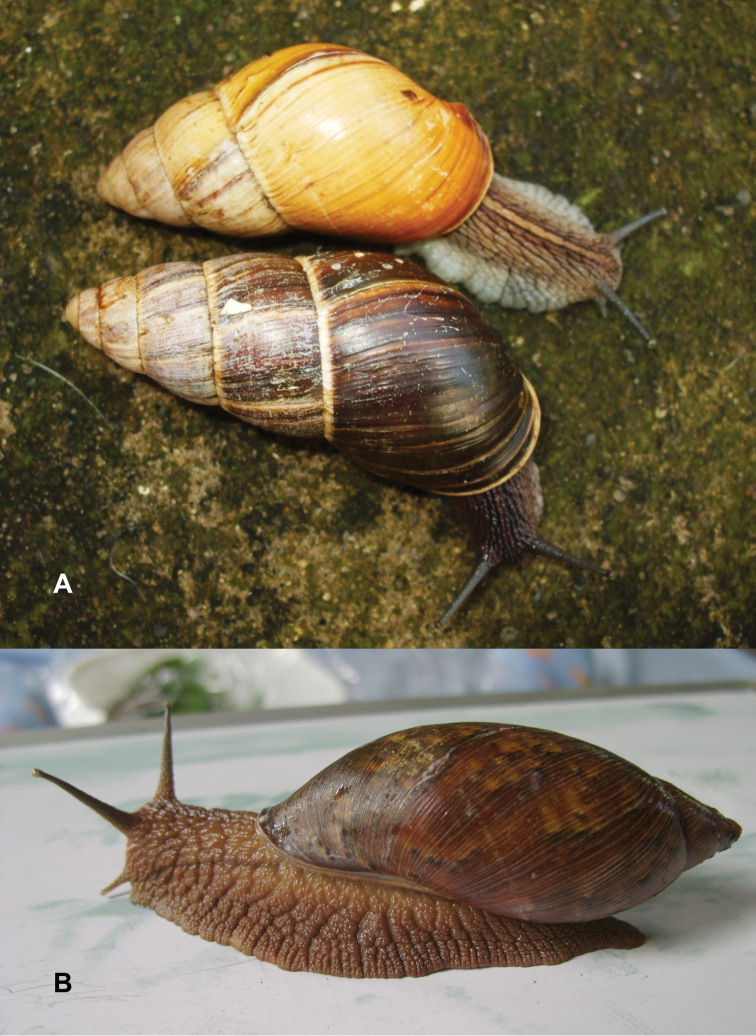
Living snails. **A** Kara thompsonii (Pfeiffer, 1848), yellow and dark colour form (courtesy M. Correoso) **B**Plekocheilus (Eurytus) floccosus (Spix in Wagner, 1827), Peru, Dept. Loreto, near río Arabela (courtesy G. Montalván).

**Figure 89.**
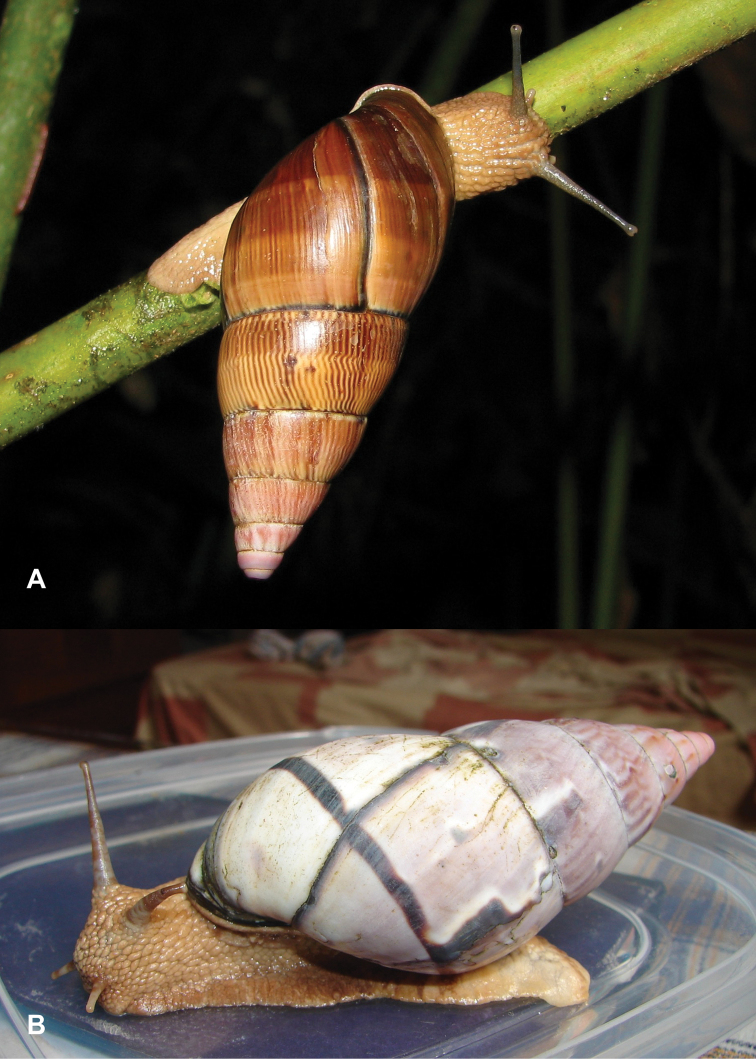
Living snails. **A** *Corona pfeifferi* (Hidalgo, 1869), Peru, Dept. Loreto, near río Curaray **B** *Corona incisa* (Hupé, 1857), Peru, Dept. Madre de Dios, Quebrada La Cachuela (all courtesy G. Montalván).

**Figure 90.**
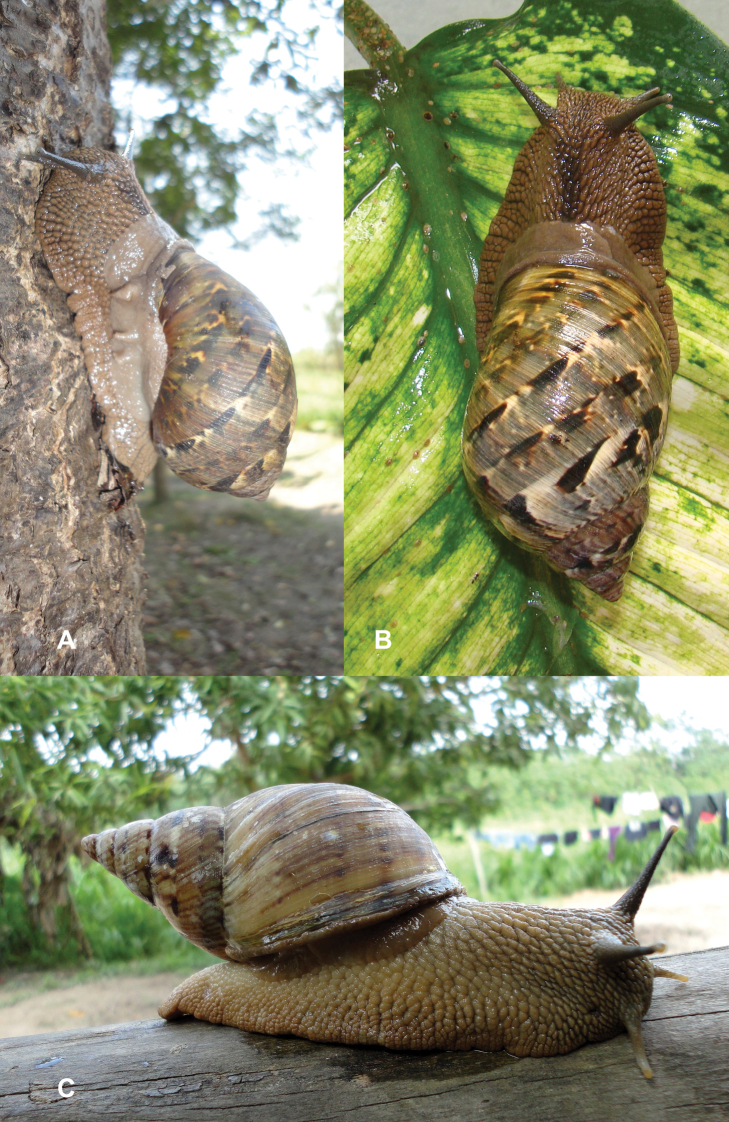
Living snails.Sultana (Sultana) meobambensis (Pfeiffer, 1855) **A–B** Peru, Dept. Loreto, San Miguel C Peru, Dept. Loreto, Santa Rita de Florida (all courtesy G. Montalván).

**Figure 91.**
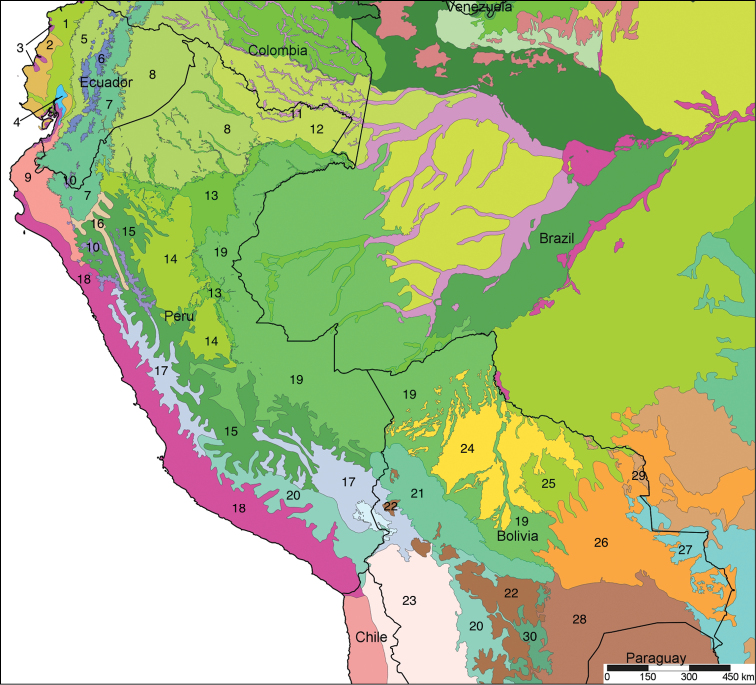
Ecoregions of Ecuador, Peru and Bolivia. **1** Western Ecuador moist forests **2** Ecuadorian dry forests **3** South American Pacific mangroves **4** Guayaquil flooded grasslands **5** Northwestern Andean montane forests **6** Northern Andean páramo **7** Eastern Cordillera real montane forests **8** Napo moist forests **9** Tumbes-Piura dry forests **10** Cordillera Central páramo **11** Purus varzea **12** Solimöes-Japurá moist forest **13** Iquitos varzea **14** Ucayalí moist forests **15** Peruvian Yungas **16** Marañon dry forests **17** Central Andean wet puna **18** Sechura desert **19** Southwest Amazon moist forests **20** Central Andean puna **21** Bolivian Yungas **22** Bolivian montane dry forests **23** Central Andean dry puna **24** Beni savanna **25** Madeira-Tapajós moist forests **26** Chiquitano dry forests **27** Pantanal **28** Dry Chaco **29** Cerrado **30** Southern Andean Yungas.

## Appendix 1

**Faunal list for Ecuador, based on this study**

**Remarks.** This list of taxa contains only the families treated herein, and is a revised version of Breure and Borrero (2008). For species marked with an asterisk (*) further evidence about their presence in this country are needed.

**Family Amphibulimidae**

1 Plekocheilus (Eurytus) aristaceus (Crosse, 1869)

2 Plekocheilus (Eurytus) aureonitens (Miller, 1878)

3 Plekocheilus (Eurytus) cardinalis (Pfeiffer, 1853)

4 Plekocheilus (Eurytus) eros (Angas, 1878)

5 Plekocheilus (Eurytus) jimenezi jimenezi (Hidalgo, 1872)

Plekocheilus (Eurytus) jimenezi oligostylus Pilsbry, 1939

6 Plekocheilus (Eurytus) lynciculus (Deville and Hupé, 1850)

[Plekocheilus (Eurytus) jacksoni Pilsbry 1939]

7 Plekocheilus (Eurytus) piperitus mcgintyi ‘Pilsbry’ H.B. Baker, 1963

8 Plekocheilus (Eurytus) nocturnus Pilsbry, 1939

9 Plekocheilus (Eurytus) roseolabrum (E.A. Smith, 1877)

10 Plekocheilus (Eurytus) taylorianus (Reeve, 1849)

[*Eurytus taylorioides minor* Miller, 1878]

11 Plekocheilus (Eurytus) tricolor (Pfeiffer, 1853)

[*Bulimus semipictus* Hidalgo, 1869]

12 Plekocheilus (Aeropictus) tenuissimus Weyrauch, 1967

13 Plekocheilus (Plekocheilus) cecepeus Breure & Araujo, 2015

**Family Megaspiridae**

14 Thaumastus (Thaumastus) buckleyi (Higgins, 1872)

15 Thaumastus (Thaumastus) flori (Jousseaume, 1897)

16 Thaumastus (Thaumastus) hartwegi (Pfeiffer in Philippi, 1846)

[*Bulimus loxensis* Miller, 1879]

[Plekocheilus (Eurytus) conspicuus Pilsbry, 1932]

17 Thaumastus (Thaumastus) integer (Pfeiffer, 1855)

[*Pachytholus pseudoiostomus* Strebel, 1909]

18 Thaumastus (Thaumastus) loxostomus (Pfeiffer, 1855)

19 Thaumastus (Thaumastus) orcesi Weyrauch, 1967

20 Thaumastus (Thaumastus) tatutor (Jousseaume, 1887)*

**Family Orthalicidae Martens in Albers, 1860**

21 *Clatrorthalicus corydon* (Crosse, 1869)

22 *Clatrorthalicus magnificus* (Pfeiffer, 1848)

23 *Clatrorthalicus phoebus* (Pfeiffer, 1863)

24 *Corona pfeifferi* (Hidalgo, 1869)

[*Corona pfeifferi cincta* Strebel, 1909]

25 Corona regalis (Hupé, 1857)

[*Bulimus loroisianus* Hupé 1857]

26 *Corona regina* (Férussac, 1823)*

27 Orthalicus mars (Pfeiffer, 1861)

28 Porphyrobaphe (Oxyorthalicus) irrorata (Reeve, 1849)

[*Dryptus irroratus elongata* Miller, 1878]

[*Dryptus irroratus minor* Miller, 1878]

29 Porphyrobaphe (Oxyorthalicus) subirroratus (da Costa, 1898)

30 Porphyrobaphe (Porphyrobaphe) iostoma (Sowerby I, 1824)

31 Porphyrobaphe (Porphyrobaphe) saturnus (Pfeiffer, 1860)

32 Sultana (Metorthalicus) atramentaria (Pfeiffer, 1855)

[*Orthalicus iodes* Shuttleworth, 1856]

[*Bulimus boussingaultii* Hupé, 1857]

33 Sultana (Metorthalicus) augusti (Jousseaume, 1887)

34 Sultana (Metorthalicus) deburghiae (Reeve, 1859)

[*Bulimus gloriosus* Pfeiffer, 1862]

[*Porphyrobaphe gloriosa elongata* Miller, 1878]

35 Sultana (Metorthalicus) kellettii (Reeve, 1850)

[*Bulimus jatesi* ‘Shuttleworth’ Hupé, 1857]

[*Bulimus fungairinoi* Hidalgo, 1867]

36 Sultana (Metorthalicus) yatesi galactostoma (Ancey, 1890)

37 Sultana (Trachyorthalicus) fraseri (Pfeiffer, 1858)

38 Kara thompsonii (Pfeiffer, 1845)

[Orphnus thompsoni var. lutea Cousin, 1887]

[Orphnus thompsoni var. nigricans Cousin, 1887]

[Orphnus thompsoni var. olivaceus Cousin, 1887]

[Orphnus thompsoni var. zebra Cousin, 1887]

**Family Simpulopsidae Schileyko, 1999**

39 Simpulopsis (Eudioptus) citrinovitrea (S. Moricand, 1836)

[*Bulimus fulguratus* Miller, 1878]

[Bulimulus (Paracochlea) willineri Hylton Scott, 1967]

## Appendix 2

**Faunal list for Peru, based on this study**

**Remarks.** This list of taxa contains only the families treated herein, and is a partially revised version of Ramírez et al. (2003). For species marked with an asterisk (*) further evidence about their presence in this country are needed.

**Family Amphibulimidae**

1 Plekocheilus (Eurytus) bruggeni Breure, 1978

2 Plekocheilus (Eurytus) floccosus (Spix, 1827)

[*Bulimus lacrimosus* Heimburg, 1884]

3 Plekocheilus (Eurytus) lynciculus (Deville and Hupé, 1850)

[Plekocheilus (Eurytus) jacksoni Pilsbry, 1939]

4 Plekocheilus (Eurytus) piperitus piperitus (Sowerby I, 1837)

[*Bulimus pseudopiperatus* J. Moricand, 1858]

5 Plekocheilus (Eurytus) prodeflexus Pilsbry, 1895

6 Plekocheilus (Eurytus) superstriatus (Sowerby III, 1890)

7 Plekocheilus (Eudolichotis) hauxwelli (Crosse, 1872)

**Family Megaspiridae**

8 Paeniscutalus crenellus (Philippi, 1867)

[Megalobulimus (Microborus) incarum Pilsbry, 1944]

[Strophocheilus (Microborus) tenuis Haas, 1955]

9 Quechua olmosensis olmosensis Zilch, 1954

Quechua olmosensis maxima Weyrauch, 1967

10 Quechua salteri (Sowerby III, 1890)

11 Quechua taulisensis Zilch, 1953

12 Thaumastus (Thaumastiella) glyptocephalus (Pilsbry, 1897)

13 Thaumastus (Thaumastiella) koepckei Zilch, 1953

14 Thaumastus (Thaumastiella) occidentalis occidentalis Weyrauch, 1960

Thaumastus (Thaumastiella) occidentalis debilisculptus Weyrauch, 1960

15 Thaumastus (Thaumastiella) sarcochrous (Pilsbry, 1897)

16 Thaumastus (Thaumastus) foveolatus (Reeve, 1849)

[*Bulimus impressus* Tschudi in Troschel, 1852]

17 Thaumastus (Thaumastus) granocinctus (Pilsbry, 1901)

[nom. n. for Bulimus (Dryptus) filocinctus Rolle, 1901]

18 Thaumastus (Thaumastus) hartwegi (Pfeiffer in Philippi, 1846)

[*Bulimus loxensis* Miller, 1879]

[Plekocheilus (Eurytus) conspicuus Pilsbry, 1932]

19 Thaumastus (Thaumastus) insolitus (Preston, 1909)

20 Thaumastus (Thaumastus) magnificus (Grateloup, 1839)*

21 Thaumastus (Thaumastus) melanocheilus (Nyst, 1845)

22 Thaumastus (Thaumastus) robertsi robertsi Pilsbry, 1932

Thaumastus (Thaumastus) robertsi satipoensis Pilsbry 1944

23 Thaumastus (Thaumastus) sangoae (Tschudi in Troschel, 1852)

24 Thaumastus (Thaumastus) taunaisii (Férussac, 1822)*

[*Bulimus achilles* Pfeiffer, 1853]

**Family Orthalicidae Martens in Albers, 1860**

25 *Corona pfeifferi* (Hidalgo, 1869)

[*Corona pfeifferi cincta* Strebel, 1909]

26 Corona regalis (Hupé, 1857)

[*Bulimus loroisianus* Hupé 1857]

27 Orthalicus bensoni (Reeve, 1849)

28 Sultana (Metorthalicus) atramentaria (Pfeiffer, 1855)

[*Orthalicus iodes* Shuttleworth, 1856]

[*Bulimus boussingaultii* Hupé, 1857]

29 Sultana (Metorthalicus) deburghiae (Reeve, 1859)*

[*Bulimus gloriosus* Pfeiffer, 1862]

[*Porphyrobaphe gloriosa elongata* Miller, 1878]

30 Sultana (Metorthalicus) kellettii (Reeve, 1850)*

[*Bulimus jatesi* ‘Shuttleworth’ Hupé, 1857]

[*Bulimus fungairinoi* Hidalgo, 1867]

31 Sultana (Metorthalicus) labeo (Broderip, 1828)

32 Sultana (Metorthalicus) macandrewi (Sowerby III, 1889)

33 Sultana (Metorthalicus) maranhonensis (Albers, 1854)

34 Sultana (Metorthalicus) meobambensis (Pfeiffer, 1855)

[*Orthalicus meobambensis carnea* Strebel, 1909]

35 Sultana (Metorthalicus) shuttleworthi (Albers, 1854)

36 Sultana (Metorthalicus) wrzesniowskii (Lubomirski, 1880)

37 Sultana (Metorthalicus) yatesi yatesi (Pfeiffer, 1855)

[*Porphyrobaphe latevittata* Shuttleworth, 1856]

[*Porphyrobaphe sublabeo* ‘Dohrn’ Ancey, 1890]

[*Porphyrobaphe vicaria* Fulton, 1896]

[*Porphyrobaphe sarcostoma* Ancey, 1903]

Sultana (Metorthalicus) yatesi galactostoma (Ancey, 1890)*

38 Sultana (Sultana) sultana (Dillwyn, 1817)

[*Orthalicus trullisatus* Shuttleworth, 1856]

[*Orthalicus sultana angustior* Preston, 1914]

39 Kara cadwaladeri (Pilsbry, 1930)

40 Kara ortiziana (Haas, 1955)

41 Kara viriata (Morelet, 1863)

42 Kara yanamensis (Morelet, 1863)

43 Scholvienia alutacea (Reeve, 1850)

[*Bulimus tarmensis* Philippi, 1867]

[*Scholvienia jaspidea minor* Strebel, 1910]

[*Bulimulus weeksi* Pilsbry, 1930]

44 Scholvienia bambamarcaensis (Breure, 1978)

45 Scholvienia bifasciata (Philippi, 1845)

[*Bulimus bitaeniatus* Nyst, 1845]

[Thaumastus (Scholvienia) bitaeniatus pallida Strebel, 1910]

[Thaumastus (Quechua) tetricus Haas, 1951]

46 Scholvienia brephoides (d’Orbigny, 1835)

[*Bulimus bifasciatus unicolor* Philippi, 1869]

47 Scholvienia claritae (Strebel, 1910)

48 Scholvienia gittenbergerorum (Breure, 1978)

49 Scholvienia huancabambensis Strebel, 1910

50 Scholvienia iserni (Philippi, 1867)

51 Scholvienia jaspidea (Morelet, 1863)

[Scholvienia jaspidea forma minor Strebel, 1910]

52 Scholvienia jelskii (Lubomirski, 1880)

53 Scholvienia porphyria (Pfeiffer, 1847)

54 Scholvienia weyrauchi (Pilsbry, 1944)

**Nomen inquirendum**

55 *Pseudoglandina agitata* Weyrauch, 1967

## Appendix 3

Faunal list for Bolivia, based on this study

**Remarks.** This list of taxa contains only the families treated herein, and is a partially revised version of Zischka (1953). For species marked with an asterisk (*) further evidence about their presence in this country are needed.

**Family Amphibulimidae**

1 Plekocheilus (Eurytus) floccosus (Spix, 1827)

[*Bulimus lacrimosus* Heimburg, 1884]

2 Plekocheilus (Eurytus) onca (d’Orbigny, 1835)

**Family Megaspiridae**

3 Thaumastus (Thaumastus) blanfordianus (Ancey, 1903)

4 Thaumastus (Thaumastus) inca (d’Orbigny, 1835)

[Thaumastus (Atahualpa) brunneus Strebel, 1910]

5 Thaumastus (Thaumastus) orobaenus (d’Orbigny, 1835)

**Family Odontostomidae**

6 Cyclodontina lemoinei (Ancey, 1892)

7 *Spixia chuquisacana* (Marshall, 1930)

8 Spixia minor (d’Orbigny, 1837)

9 Spixia striata (Wagner in Spix, 1827)

[*Pupa spixii major* d’Orbigny, 1837]

**Family Orthalicidae Martens in Albers, 1860**

10 Corona incisa (Hupé, 1857)

[*Corona incisa machadoensis* Strebel, 1909]

11 Orthalicus phlogerus (d’Orbigny, 1835)

12 Orthalicus pulchellus (Spix, 1827)

13 Sultana (Sultana) sultana (Dillwyn, 1817)

[*Orthalicus trullisatus* Shuttleworth, 1856]

[*Orthalicus sultana angustior* Preston, 1914]

14 Scholvienia porphyria (Pfeiffer, 1847)*

## Appendix 4

**Terrestrial ecoregions**

The following ecoregions (see Figure 91)—as currently defined by the World Wildlife Fund, and occurring in the study area—have been used in the text as far as distribution records can be assigned to any of them. It should be noted that the Ecuadorian ones as used by Breure and Borrero (2008) have been partly re-defined or re-named. Moreover, the following caveats apply (Olson et al. 2004): First, no single biogeographic framework is optimal for all taxa. Ecoregions reflect the best compromise for as many taxa as possible. Second, ecoregion boundaries rarely form abrupt edges; rather, ecotones and mosaic habitats bound them. Third, most ecoregions contain habitats that differ from their assigned biome (e.g., for example, rainforest ecoregions in Amazonia often contain small edaphic savannas). With these caveats in mind, ecoregions can form useful units for biological analysis and for conservation planning and action.

The numbers used in Figure 91 are given between brackets; between square brackets the corresponding WWF codes are given (see for more information WWF 2015).

**Tropical and subtropical moist broadleaf forests**

Bolivian Yungas (Bolivia, Peru) (21) [NT0105]

Eastern Cordillera real montane forests (Ecuador, Peru) (7) [NT0121]

Iquitos varzea (Peru) (13) [NT0128]

Madeira-Tapajós moist forests (Bolivia) (25) [NT0135]

Napo moist forests (Ecuador, Peru) (8) [NT0142]

Northwestern Andean montane forests (Ecuador) (5) [NT0145]

Peruvian Yungas (Peru) (15) [NT0153]

Purus varzea (Peru) (11) [NT0156]

Solimöes-Japurá moist forests (Peru) (12) [NT0163]

Southern Andean Yungas (Bolivia) (30) [NT0165]

Southwest Amazon moist forests (Bolivia, Peru) (19) [NT0166]

Ucayalí moist forests (Peru) (14) [NT0174]

Western Ecuador moist forests (Ecuador) (1) [NT0178]

**Tropical and subtropical dry broadleaf forests**

Bolivian montane dry forests (Bolivia) (22) [NT0206]

Dry Chaco (Bolivia) (28) [NT0210]

Chiquitano dry forests (Bolivia) (26) [NT0212]

Ecuadorian dry forests (Ecuador) (2) [NT0214]

Marañon dry forests (Peru) (16) [NT0223]

Tumbes-Piura dry forests (Ecuador, Peru) (9) [NT0232]

**Tropical and subtropical grasslands, savannas, and shrublands**

Beni savanna (Bolivia) (24) [NT0702]

Cerrado (Bolivia) (29) [NT0704]

**Flooded grassland and savannas**

Guayaquil flooded grasslands (Ecuador) (4) [NT0905]

Pantanal (Bolivia) (27) [NT0907]

**Montane grassland and shrublands**

Central Andean dry puna (Bolivia) (23) [NT1001]

Central Andean puna (Bolivia, Peru) (20) [NT1002]

Central Andean wet puna (Bolivia, Peru) (17) [NT1003]

Cordillera Central páramo (Ecuador, Peru) (10) [NT1004]

Northern Andean páramo (Ecuador) (6) [NT1006]

**Deserts and xeric shrublands**

Sechura desert (Peru) (18) [NT1315]

**Mangroves**

South American Pacific mangroves (3) [NT1405]

## Appendix 5

**Index to the taxa treated in this paper**

**Remarks.** A black diamond (❖) denotes a nomenclatoral act (new taxon, synonym, combination, or status), a black star (✦) indicates a *nomen inquirendum*, a reference mark (※) indicates taxa treated as junior synonyms or homonyms. (LT) is a lectotype designation. Supraspecific taxa are printed in bold.

*achilles* Pfeiffer, 1853※—41

***Aeropictus* Weyrauch, 1967**—10

*agitata* Weyrauch, 1967✦—83

*alutacea* Reeve, 1849—64

**Amphibulimidae P. Fischer, 1873**—9

*angulata* Wagner, 1827—86

*angustior* Preston, 1914—80

*aristaceus* Crosse, 1869—14

*atramentaria* Pfeiffer, 1855—73

*augusti* Jousseaume, 1887—73

*aureonitens* Miller, 1878—14

*bambamarcaensis* Breure, 1978—65

*bensoni* Reeve, 1849—55

*bifulguratus* Reeve, 1849—56

*bifasciatus* Philippi, 1845—65

*bitaeniatus* Nyst, 1845※—65

*bivittatus* Philippi, 1845※—65

*blanfordianus* Ancey, 1903—31

*boussingaultii* Hupé, 1857※—73

*bruggeni* Breure, 1978—15

*brephoides* d’Orbigny, 1835—67

*brunneus* Strebel, 1910※—34, 86

*buckleyi* Higgins, 1872—31

*cadwaladeri* Pilsbry, 1930—51

*cardinalis* Pfeiffer, 1853—15

*carnea* Strebel, 1909※—80

*cecepeus* Breure and Araujo, 2015—25

*chuquisacana* Marshall, 1930—42❖

*cincta* Strebel, 1909※—50

*citrinovitrea* S. Moricand, 1836—82

*claritae* Strebel, 1910—68

***Clathrorthalicus* Strebel, 1909—45**

*conspicuus* Pilsbry, 1932※—34 ❖

***Corona* Albers, 1850—47**

*corydon* Crosse, 1869—46❖

*crenellus* Philippi, 1867—26

***Cyclodontina* Beck, 1837—42**

*debilisculptus* Weyrauch, 1960—29

*deburghiae* Reeve, 1859—74

*doliarius* da Costa, 1898—16

*elongata* (*irroratus*) Miller, 1878※—59

*elongatus* (*gloriosus*) Miller, 1878※—74

*eros* Angas, 1878—16

***Eudioptus* Martens in Albers, 1860—82**

***Eudolichotis* Pilsbry, 1896—11**

**Eurytus Albers, 1850—12**

*excisus* Martens, 1885—86

*filocinctus* Rolle, 1901※—33

*floccosa* Spix in Wagner, 1827—17

*flori* Jousseaume, 1897—32

*foveolatus* Reeve, 1849—32

*fraseri* Pfeiffer, 1858—74

*fulgur* Miller, 1878※—56

*fulguratus* Miller, 1878※—82

*fungairinoi* Hidalgo, 1867※—75

*galactostoma* Ancey, 1890—79

*gibbonius* Hidalgo, 1870※—17

*gittenbergerorum* Breure, 1978—68

*gloriosus* Pfeiffer, 1862※—74

*glyptocephalus* Pilsbry, 1897—28

*granocinctus* Pilsbry, 1901—33

*granulosus* Broderip, 1832—86

*grevillei* Pfeiffer, 1876※—60

*gruneri* Strebel, 1909※—85❖

*hartwegi* Pfeiffer in Philippi, 1846—34

*hauxwelli* Crosse, 1872—11

*huancabambensis* Strebel, 1910—69

*impressus* Tschudi in Troschel, 1852※—32

*inca* d’Orbigny, 1835—34

*incarum* Pilsbry, 1944※—26

*incisa* Hupé, 1857—49 (LT)

*insolitus* Preston, 1909—35

*integer* Pfeiffer, 1855—35

*iodes* Shuttleworth, 1856※—73

*iostoma* Sowerby I, 1824—60

*irrorata* Reeve, 1849—59

*isabellinus* Martens, 1873※—55

*iserni* Philippi, 1867—69

*jacksoni* Pilsbry, 1939※—18

*jaspidea* Morelet, 1863—70

*jatesi* ‘Shuttleworth’ Hupé, 1857※—75

*jelskii* Lubomirski, 1880—70

*jimenezi* Hidalgo, 1872—17

***Kara* Strebel, 1910—51**

*kellettii* Reeve, 1850—75

*koepckei* Zilch, 1953—28

*labeo* Broderip, 1828—76

*labeo* Reeve, 1848※—78

*lacrimosus* Heimburg, 1884※—17

*latevittata* Shuttleworth, 1856※—78

*lemoinei* Ancey, 1892—43

*loroisianus* Hupé, 1857※—50

*loxensis* Miller, 1879※—34

*loxostomus* Pfeiffer, 1855—36

*lutea* Cousin, 1887※—53

*lynciculus* Deville and Hupé, 1850—18

*macandrewi* Sowerby III, 1889—76

*machadoensis* Strebel, 1909※—49

*magnificus* Pfeiffer, 1848—47

*magnificus* Grateloup, 1839—36

*mahogani* Pfeiffer, 1841※—32

*major* d’Orbigny, 1838※—45

*manco* Pilsbry, 1930—86

*maracaibensis* Pfeiffer, 1856—84, 85

*maranhonensis* Albers, 1854—77

*mars* Pfeiffer, 1861—57

*maximus* Weyrauch, 1967—62❖

*mcgintyi* ‘Pilsbry’ H.B. Baker, 1963—21❖

**Megaspiridae Pilsbry, 1904—25**

*melanocheilus* Nyst, 1845—37

*meobambensis* Pfeiffer, 1855—80

***Metorthalicus* Pilsbry, 1899—72**

*minor* (*irroratus*) Miller, 1878※—59

*minor* (*taylorioides*) Miller, 1878※—23

*minor* d’Orbigny 1837—44

*minor* Strebel, 1910※—64❖

*nigricans* Cousin, 1887※—53

*nocturnus* Pilsbry, 1939—19

*obductus* Shuttleworth, 1856—84

*occidentalis* Weyrauch, 1960—29

**Odontostomidae Pilsbry & Vanatta, 1898—42**

*oligostylus* Pilsbry, 1939—19

*olivaceus* Cousin, 1887※—53

*olmosensis* Zilch, 1954—62

*onca* d’Orbigny, 1835—20

*orcesi* Weyrauch, 1967—37

*orobaena* d’Orbigny, 1835—38

**Orthalicidae Martens in Albers, 1860—45**

***Orthalicus* Beck, 1837—54**

*ortizianus* Haas, 1955—52

***Oxyorthalicus* Strebel, 1909—59**

***Paeniscutalus* Wurtz, 1947—25**

*pentadina* d’Orbigny, 1835※—17

*pfeifferi* Hidalgo, 1869—50

*phlogera* d’Orbigny, 1835—57

*phoebus* Pfeiffer, 1863—47

*pilsbryi* Strebel, 1909—85

*piperitus* Sowerby I, 1837—20

**Plekocheilus Guilding, 1828—9, 24**

*ponderosus* Strebel in Strebel and Pfeffer, 1882—87

*porphyria* Pfeiffer, 1847—70

**Porphyrobaphe Shuttleworth, 1856—58, 60**

*princeps* Broderip in Sowerby I and II, 1833—85

*prodeflexus* Pilsbry, 1895—21❖

*pseudoiostomus* Strebel, 1909※—35

*pseudopiperatus* J. Moricand, 1858※—20

*pulchellus* Spix in Wagner, 1827—58

*pulicarius* Reeve, 1848—84

***Quechua* Strebel, 1910—61**

*regalis* Hupé, 1857—50

*regina* Férussac, 1823—84

*robertsi* Pilsbry, 1932—38

*roseolabrum* E.A. Smith, 1877—22

*salteri* Sowerby III, 1890—63

*sangoae* Tschudi in Troschel, 1852—39

*sarcochrous* Pilsbry, 1897—30

*sarcostoma* Ancey, 1903※—78

*satipoensis* Pilsbry, 1944—39

*saturnus* Pfeiffer, 1860—61

***Scholvienia* Strebel, 1910—64**

*semipictus* Hidalgo, 1869※—24

*shuttleworthi* Albers, 1854—77

**Simpulopsis Beck, 1837—81**

**Simpulopsidae Schileyko, 1999—81**

***Sparnotion* Pilsbry, 1944**※—**11**❖

***Spixia* Pilsbry & Vanatta, 1898—43**

*striata* Wagner, 1827—43

*subirroratus* da Costa, 1898—60

*sublabeo* ‘Dohrn’ Ancey, 1890※—78

*sultana* Dillwyn, 1817—80

**Sultana Shuttleworth, 1856—72, 79**

*sumaqwayqu* sp. n.—40❖

*superstriatus* Sowerby III, 1890—23

*taeniolus* Nyst, 1845✦—86

*tarmensis* Philippi, 1867※—64

*tatutor* Jousseaume, 1887—41

*taulisensis* Zilch, 1953—63

*taunaisii* Férussac, 1822—41

*taylorianus* Reeve, 1849—23

*tenuis* Haas, 1955※—26

*tenuissimus* Weyrauch, 1967—10

*tetricus* Haas, 1951※—66

***Thaumastiella* Weyrauch, 1956—27**

***Thaumastus* Martens in Albers, 1860—27, 30**

*thompsonii* Pfeiffer, 1845—53

***Thomsenia* Strebel, 1910**※—**64**❖

***Trachyorthalicus* Strebel, 1909**※—**72**

*tricolor* Pfeiffer, 1853—24

*trullisatus* Shuttleworth, 1856※—80

*unicolor* Philippi, 1869※—67❖

*vicaria* Fulton, 1896—77

*viriata* Morelet, 1863—53

*weeksi* Pilsbry, 1930※—64

*weyrauchi* Pilsbry, 1944—71

*willineri* Hylton Scott, 1967※—82

*wrzesniowskii* Lubomirski, 1880—78

*yanamensis* Morelet, 1863—54

*yatesi* Pfeiffer, 1855—78

*zebra* Cousin, 1887※—53

*zebra* Müller, 1774—87
